# Tracking the Fragile X Mental Retardation Protein in a Highly Ordered Neuronal RiboNucleoParticles Population: A Link between Stalled Polyribosomes and RNA Granules

**DOI:** 10.1371/journal.pgen.1006192

**Published:** 2016-07-27

**Authors:** Rachid El Fatimy, Laetitia Davidovic, Sandra Tremblay, Xavier Jaglin, Alain Dury, Claude Robert, Paul De Koninck, Edouard W. Khandjian

**Affiliations:** 1 Institut universitaire en santé mentale de Québec, Quebec, Canada; 2 Département de Psychiatrie et de Neurosciences, Faculté de Médecine, Université Laval, Québec, Quebec, Canada; 3 Institut de Pharmacologie Moléculaire et Cellulaire, UMR7275, Université de Nice-Sophia Antipolis, F-06560 Valbonne, France; 4 Neuroscience Institute, Department of Neuroscience and Physiology, New York University, New York, New York, United States of America; 5 Centre de recherche en biologie de la reproduction, Département des sciences animales, Faculté des sciences de l’agriculture et de l’alimentation, Université Laval, Québec, Quebec, Canada; 6 Département de Biochimie, Microbiologie et Bio-Informatique, Université Laval, Québec, Quebec, Canada; Rockefeller University, UNITED STATES

## Abstract

Local translation at the synapse plays key roles in neuron development and activity-dependent synaptic plasticity. mRNAs are translocated from the neuronal soma to the distant synapses as compacted ribonucleoparticles referred to as RNA granules. These contain many RNA-binding proteins, including the Fragile X Mental Retardation Protein (FMRP), the absence of which results in Fragile X Syndrome, the most common inherited form of intellectual disability and the leading genetic cause of autism. Using FMRP as a tracer, we purified a specific population of RNA granules from mouse brain homogenates. Protein composition analyses revealed a strong relationship between polyribosomes and RNA granules. However, the latter have distinct architectural and structural properties, since they are detected as close compact structures as observed by electron microscopy, and converging evidence point to the possibility that these structures emerge from stalled polyribosomes. Time-lapse video microscopy indicated that single granules merge to form cargoes that are transported from the soma to distal locations. Transcriptomic analyses showed that a subset of mRNAs involved in cytoskeleton remodelling and neural development is selectively enriched in RNA granules. One third of the putative mRNA targets described for FMRP appear to be transported in granules and FMRP is more abundant in granules than in polyribosomes. This observation supports a primary role for FMRP in granules biology. Our findings open new avenues for the study of RNA granule dysfunctions in animal models of nervous system disorders, such as Fragile X syndrome.

## Author Summary

Fragile X syndrome is the most common form of inherited mental retardation affecting approximately 1 female out of 7000 and 1 male out of 4000 worldwide. The syndrome is due to the silencing of a single gene, the *Fragile Mental Retardation 1* (*FMR1*), that codes for the Fragile X mental retardation protein (FMRP). This protein is highly expressed in brain and controls local protein synthesis essential for neuronal development and maturation as well as the formation of neural circuits. Several studies suggest a role for FMRP in the regulation of mRNA transport along axons and dendrites to distant synaptic locations in structures called RNA granules. Here we report the isolation of a particular subpopulation of these structures and the analysis of their architecture and composition in terms of RNA and protein. Also, using time-lapse video microscopy, we monitored granule transport and fusion throughout neuronal processes. These findings open new avenues for the study of RNA transport dysfunctions in animal models of nervous system disorders.

## Introduction

Neurons are remarkably diverse in shape [1]. They vary from simple unipolar to highly complex multipolar cells, decorated with complex projections of up several centimeters and even one meter in certain cases. Through billions of synaptic connections, these cell-to-cell interactions are the basis for neural circuits that are highly adaptable and functionally autonomous. Their remodelling and adaptation properties contribute to synaptic plasticity [2–5]. These changes rely on rapid local modulation of protein synthesis that is dependent on the presence of the translational machinery and mRNA at the synapse [6]. At the time, the discovery of ribosomal RNA in the axoplasm of the squid giant axon was considered odd and specific to this species [7]. Later, the observation of polyribosome aggregates beneath postsynaptic sites at the base of dendritic spines [8] and in the postsynaptic area of the squid giant synapse [9] convinced scientists that translation could also occur outside of the soma in an autonomous and rapid response to synaptic activity.

Supplying and maintaining subcellular compartments far away from the neuronal soma brings up complex conceptual and biological questions in terms of logistics. Most of protein synthesis takes place in the soma, as the bulk of mRNAs is translated into the cell body. However, a subset of mRNAs is delivered either at presynaptic axonal terminals or at postsynaptic dendritic spines [10,11] where they are further translated into proteins, the synthesis of which is specifically required for adaptation to the local needs of the synapse [12,13]. This extrasomatic targeting of mRNAs allows to rapidly control the synthesis and distribution of the corresponding protein, regulating its level at individual axonal terminals or dendritic spines, in response to external stimuli. The mechanisms of transport, targeting, and release of neurospecific mRNAs at the synapse are gradually being unveiled. While PeriAxoplasmic Ribosomal Plaques (PARPs) corresponding to the translation apparatus have been detected in squid axon [14], RNA granules were observed in the arborisation of neurons in culture [15]. When isolated using sucrose gradients, these granules exhibited sedimentation properties with S values higher than those of polyribosomes [16]. However, while these granules can be isolated from neurons in primary culture, attempts to purify them from total brain have not been conclusive. Preparations of granules were contaminated by co-sedimentation of other structures such as clathrin-coated vesicles [17] or polyribosomes [18]. Consequently, little is known about the protein and RNA species present in these structures. While mRNAs present in dendrites [11] may possibly be transported in travelling modules, there is no formal biochemical evidence yet for isolated granules. In the present study, we describe a method to isolate and purify RNA granules from mouse brain homogenates, using FMRP as a tracer. Furthermore, we provide a comprehensive proteomic and transcriptomic profiling of mouse brain RNA granules. Finally, we propose a definition of what we consider to be a single granule unit and what we conceive as a cargo of granules.

## Results

### Isolation of RNA granules from mouse brain

**Fig 1:**
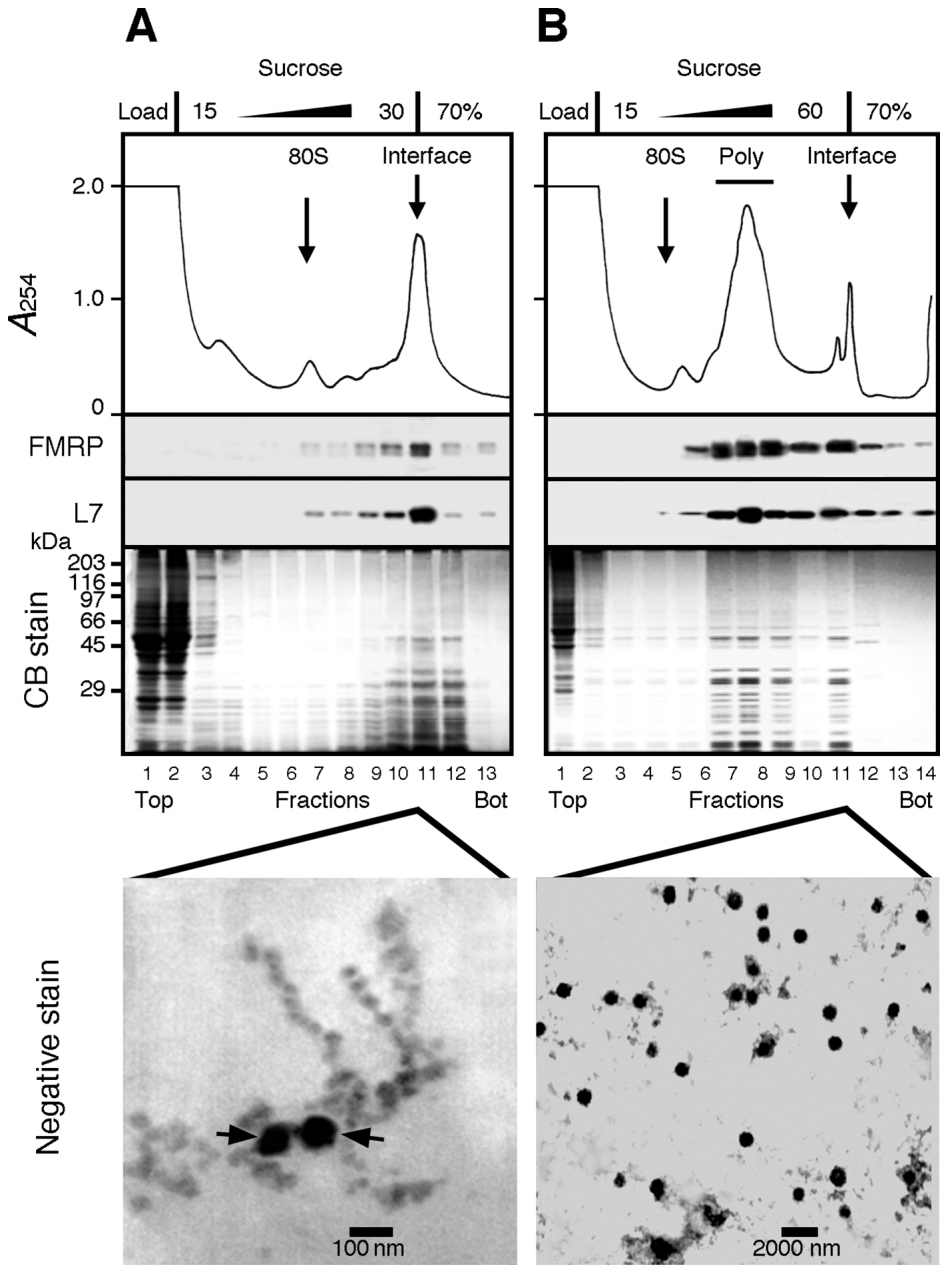
Attempts to separate polyribosomes from heavy sedimenting granules. **A)** Brain cytoplasmic extract prepared without detergent was analysed by sedimentation velocity throughout a 5–30% (w/v) sucrose density gradient layered over a 70% (w/v) sucrose cushion. The collected fractions were analysed by SDS-PAGE followed by Coomassie blue staining and immunoblotting to detect the ribosomal protein L7 and FMRP. A major UV-absorbing peak is observed at the 30–70% sucrose interface that contained L7 and FMRP. Electron micrographs of this fraction revealed that both beads on a string like structures of polyribosomes and dense amorphous granule-like structures (arrows) were recovered at the interface. **B**) Total polyribosomes from brain cytoplasmic extract were first concentrated by ultracentrifugation, resuspended, and then analysed by sedimentation velocity throughout a 15–60% (w/v) sucrose density gradient layered over a 70% (w/v) sucrose pad. All collected fractions were analysed by Coomassie blue staining after SDS-PAGE, and by immunoblotting to detect the ribosomal protein L7 and FMRP. While polyribosomes were detected in the middle of the gradient, electron micrographs revealed that the sucrose interface fraction contained 100–800 nm diameter granule-like structures. Note the extended scale in **B** as compared to that in panel **A**.

To study the diversity and complexity of neuronal RNA granules, we sought to obtain substantial amounts of these structures. Using mouse brain, we first applied the procedure described by Aschrafi et al. [18] which is based on different sedimentation rates of RNA granules and of polyribosomes [16,19]. Therefore, these two populations can be separated using isokinetic centrifugation through linear sucrose density gradients. Total brain cytoplasmic extracts were prepared without detergent from 10 days-old mice brain, loaded on a 15–30% w/v sucrose gradient over a 70% sucrose pad and centrifuged at 34 000 rpm for 2 hours. Continuous UV monitoring during the course of gradient unloading showed the presence of distinct peaks corresponding to two UV-absorbing populations with different sedimentation properties. A minor peak was observed in the middle of the gradient while a prominent second peak was at the 30–70% sucrose interphase (Fig 1A). According to Ashrafi et al. [18], the latter retains the granules fraction.

To determine the position of polyribosomes in such a shallow sucrose gradient (15–30% w/v), we fractioned the gradient and analysed the distribution of both the ribosomal protein L7 and FMRP, a well-known RNA-binding protein associated to polyribosomes [20,21]. This showed that the minor peak corresponded to monosomes sedimenting at 80S, while most of L7 and FMRP was recovered at the sucrose interface (Fig 1A). These observations strongly suggested that the fraction studied by Ashrafi et al. was unlikely to correspond to granules, as it contained also a high proportion of polyribosomes. Moreover, staining the gel with Coomassie blue showed that the great majority of the sedimenting material was concentrated at the 30–70% sucrose interface (Fig 1A). Finally, electron microscopy of the interface fraction revealed the coexistence of dense amorphous-looking granules and polyribosomes, assembled as beads on a string (Fig 1A). Altogether, this suggested that the method described by Ashrafi et al. was inadequate to separate RNA granules from polyribosomes in mouse brain.

**Fig 2:**
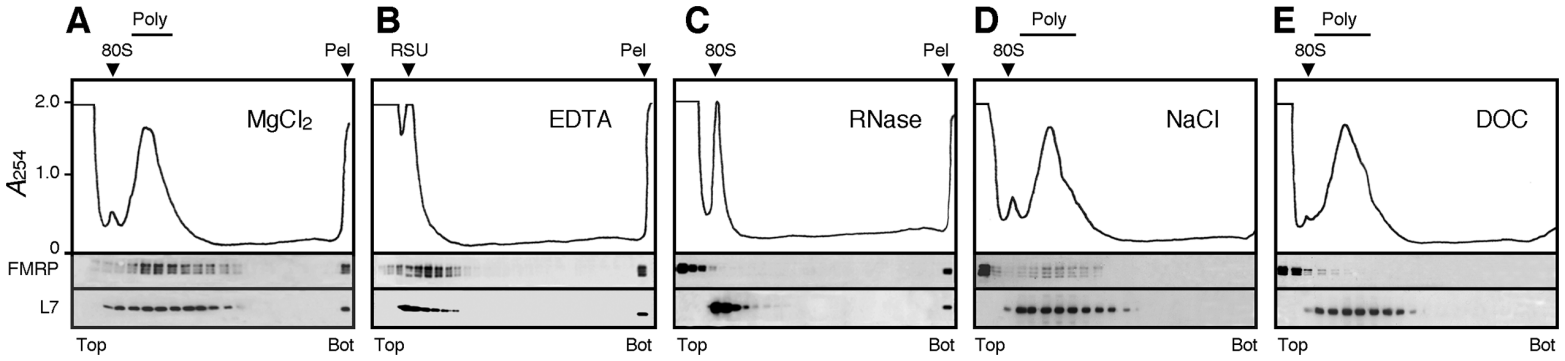
Heavy sedimenting structures are not *bone fide* polyribosomes. Aliquots of concentrated polyribosomes were analysed by sedimentation velocity through 15–60% (w/v) sucrose density gradients. **A)** In the presence of MgCl_2_, FMRP was detected at the level of polyribosomes (Poly) and the pellet fraction (Pel). After incubation with 30 mM EDTA (**B**) or treatment with 10 μg/ml RNase A (**C**), a clear displacement of the ribosomal L7 protein and FMRP towards the top of the gradient was observed, while both proteins were still detectable in the pellet (Pel). Conversely, in the presence of 0.4 M NaCl (**D**) or the anionic detergent deoxycholate (DOC, **E**), the majority of FMRP was found in the loading volume that did not penetrate the gradient, while the polyribosomal UV profile remained unchanged. In these conditions no UV-absorbing materials as well as no FMRP or L7 were detected in the pellet fraction.

Since discriminating polyribosomes from granules with a 15–30% w/v sucrose density gradient was not possible, we opted for a more standard 15–60% w/v linear gradient over a 70% w/v sucrose cushion. We used brains from P10 mice, since preparing a cytoplasmic fraction enriched in polyribosomes and heavy sedimenting structures is easier at this age [20]. The cytoplasmic sap was ultracentrifuged over a 60% w/v sucrose cushion. The resulting opalescent pellet was resuspended and further analysed by isokinetic centrifugation. Under these conditions, the ribosomal L7 protein and FMRP were detected at the level of polyribosomes (Fig 1B), as previously documented [20,21], indicating that polyribosomes could be separated from the 60–70% sucrose interface. Also, both proteins were present at the 60–70% w/v interface, but penetrated the sucrose cushion as they also appeared in lower fractions. To confirm that the material present at the interface did correspond to granules, samples were examined by electron microscopy following negative staining. Electron dense round shaped particles ranging from 100 to 800 nm were observed (note the extended scale in Fig 1B as compared to Fig 1A). Altogether, these data demonstrate that a fraction enriched in granules is obtained at the interface under the conditions described here. However, contamination by large sedimenting polyribosomes could not be ruled out because of the close vicinity of the collected fractions. We therefore modified three parameters: 1) we eliminated the sucrose cushion to allow rapidly sedimenting material to concentrate at the bottom of the centrifuge tube; 2) we reduced the centrifugation time down to 45 minutes to separate the polyribosome fractions from heavy sedimenting structures, and 3) we unloaded the sucrose gradient from the top using the Auto Densi-Flow (Buchler) rather than piercing the tube, thus protecting the integrity of the pellet. These adjustments, allowed the separation of polyribosomes from heavy sedimenting structures containing L7 and FMRP, which were recovered at the bottom of the tube (Fig 2A).

The presence of structures sedimenting faster than polyribosomes, suggested that this pellet fraction corresponded either to granules or polyribosomes aggregates. To discriminate between these two structures, polyribosome-enriched extracts were submitted to different treatments prior to velocity sedimentation through sucrose density gradients. An EDTA treatment, which dissociates polyribosomes into their ribosomal subunits (RSU), and an RNAse treatment, that completely destroys polyribosomes had no effect on the presence of FMRP and L7 in the pellet (Fig 2B and 2C). This suggested that these heavy-sedimenting structures did not correspond to classical *bone fide* polyribosomes. We then tested the effect of high-salt conditions (0.4 M NaCl) on the material contained in the pellet. We observed that approximately 50 to 70% of FMRP was removed from polyribosomes and was recovered at the top of the gradient, while the rest of FMRP remained associated with polyribosomes (Fig 2D), as previously reported [22]. Under these high-salt conditions, we neither detected a visible pellet, nor recovered UV absorbing material or FMRP and L7 at the bottom of the tubes (Fig 2D). Similarly, while treatment with the anionic detergent deoxycholate (DOC) did not affect the polyribosomal UV profile, it eliminated UV absorbing material from the pellet (Fig 2E). This is in agreement with previous studies showing that FMRP together with other proteins, is stripped off polyribosomes by DOC [20,21,23]. These observations strongly suggested that the material recovered at the bottom of the tubes present a tertiary structure different from classical polyribosomes.

**Fig 3:**
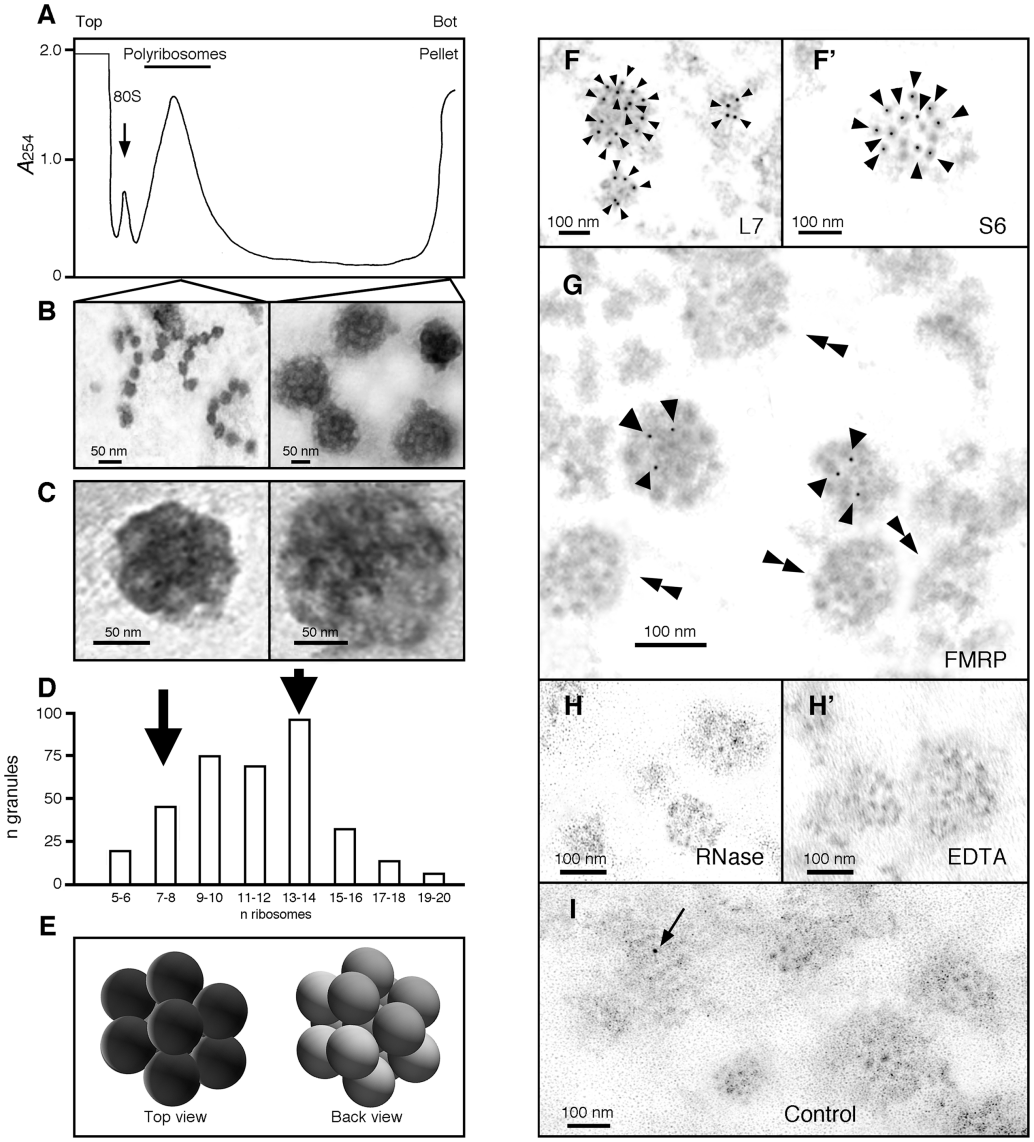
Ribosomes are the basic units of the granules. **A)** Concentrated samples of polyribosomes were analysed by centrifugation through linear 15–60% (w/v) sucrose gradient, and fractions were collected with continuous monitoring at 254 nm. Reducing the time of centrifugation to 45 min, allowed polyribosomes to be separated from granules that sediment at the bottom of the gradient. **B)** Isolated polyribosomes and granules were observed by electron microscopy after negative staining. While polyribosomes present an open structure similar to beads on a string, granules display a densely compacted morula-like structure. **C)** Shown are two granules of two different sizes. The diameter of each unit composing the granules is similar to the reported size of 25 nm for ribosomes. **D)** Size distribution of granules according to their number of visible units as revealed by negative staining. Quantification of ribosomes present in each granule shows that their number varies from 5 to 20, with a mean average of 9 to14 ribosomes. **E)** 3D model of granules from top and back views suggests that the number of ribosomes observed in flatten EM preparations is under estimated. **F** and **F’)** Immunogold labelling of ribosomal protein L7 and S6 (15 nm, arrow heads), and **G)** FMRP (5 nm, arrow heads) in granules; double arrow heads point to granules free of FMRP gold signals. **H** and **H’)** RNAse and EDTA treated granules show slightly altered structures. **I)** Control analysis without primary antibodies showing a single contaminant signal (arrow). Ultrathin sections were obtained on materials embedded in LR-White resin.

We then performed electron-microscopy analyses to visualize the components present in the pellet and in the polyribosomal fractions (Fig 3A). Electron micrographs of polyribosomal fractions revealed the typical appearance of polyribosomes with ribosomes assembled on mRNA as beads on a string, while clumps of amorphous structures were visible in the resuspended pellet fractions. We hypothesized that these aggregates were due to the high g forces generated during ultracentrifugation, compacting the particles against the tube bottom wall. We therefore added a step in which the pellet was dispersed by two short bursts of ultra-sonication. Following that treatment, the structures present in the pellet fraction appeared as a heterogeneous set of small granules with size ranging from 100 to 300 nm (Fig 3B). Higher magnification revealed a morula-like structure of granules, each formed of round units of 25 nm in diameter similar to ribosomes [24] (Fig 3C). Quantification in an array of 350 granules showed that the number of units present in a single granule range from 5 to 20 (Fig 3C and 3D). Because EM preparations tend to flatten structures, we hypothesized that the number of ribosomal units was underestimated. Indeed, 3D reconstruction models revealed the hidden face of the preparations. Thus, granules estimated to contain 7 ribosomes, might in fact accommodate 12 to 13 units (Fig 3E). Immunogold labelling on ultra-thin sections of LR-White resin embedded granules with antibodies against the large L7 and the small S6 ribosomal subunit proteins, confirmed that the units composing the granules corresponded to ribosomes (Fig 3F and 3F’). While we expected that all ribosomes contained in a granule would react to the antibodies, these granules were not uniformly labelled. This was probably due to the fact that the protein epitopes were localized above or below the levels of the ultrathin sections. Immunogold labelling of FMRP showed that the protein was not present in all granules (Fig 3G), as it was the case for its two homologues, FXR1P and FXR2P (S1 Fig). The average number of FMRP-gold signals detected in each granule was 3 as determined in n = 100 FMRP positive granules (S2 Fig). However, this number might be underestimated, because the FMRP epitopes might be missing, as is the case for L7 (see above). On the other hand, only 30% of the granules carried FMRP-gold signals. A more detailed quantitative study of the distribution of FMRP in granules, using immunofluorescence approach, is presented below. We observed the presence of granules in the pellets recovered after RNase and EDTA treatments of the cytoplasmic sap (see Fig 2B and 2C). However, we systematically noted that their morphology was slightly altered as less defined images were obtained (Fig 3H and 3H’). Finally, to serve as negative controls, sections were incubated in the presence of the sole secondary gold-labelled antibodies. Occasionally, single gold-particle was observed outside of the granules in different regions of the sections (Fig 3I).

These results collectively suggest that although granules are highly diverse in terms of size and composition, their basic unit remains the ribosome.

### Purification of the granules

**Fig 4:**
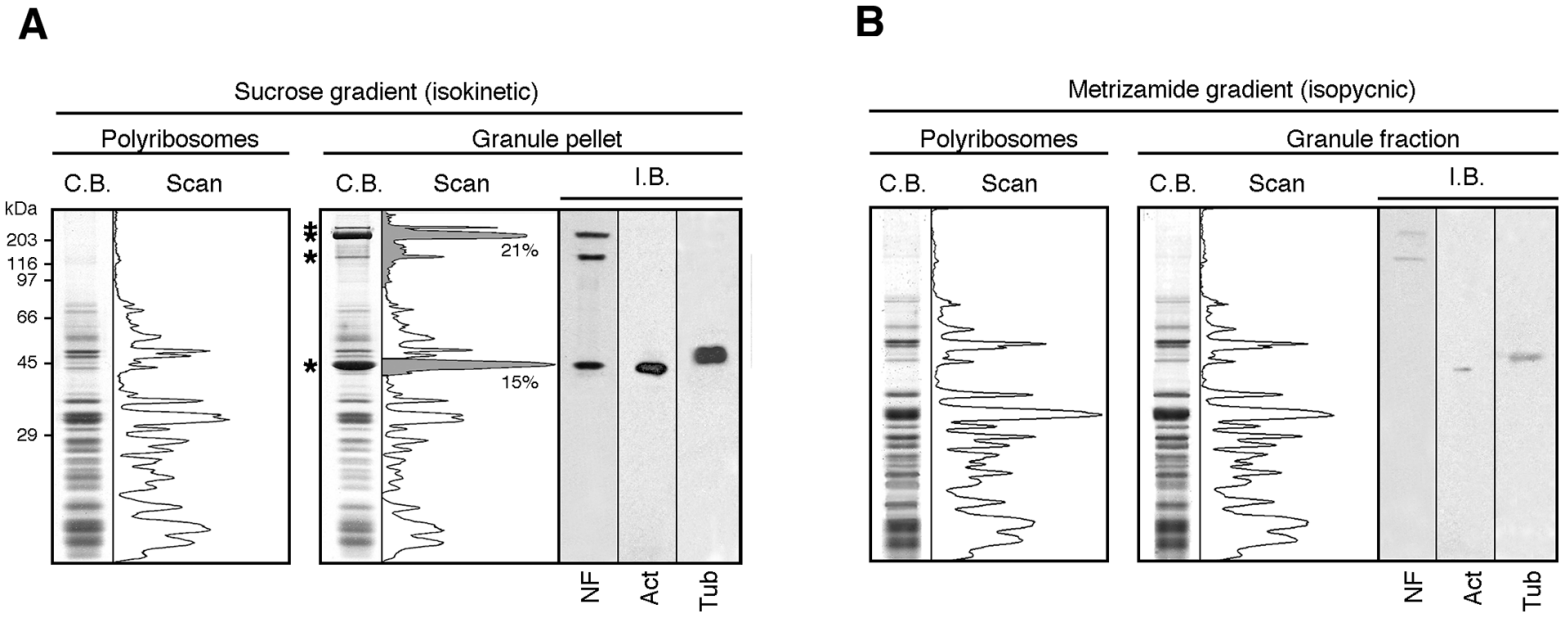
Protein analyses of the enriched and purified granules fractions. Distribution of proteins in polyribosomes (P) and granules (G) fractions: **A)** Following centrifugation in sucrose gradient and, **B)** After isopynic centrifugation in Metrizamide gradients. Coomassie blue staining of proteins separated by SDS-PAGE and their corresponding scans. Immunoblot analyses (I.B.) for both preparations were performed in parallel in the same conditions with identical exposure time to the X ray films.

While the procedures described above were appropriate to obtain fractions highly enriched with granules, we wondered whether they were purified sufficiently for further biochemical studies. Granule fractions obtained as pellets (see Fig 3A) were analysed by SDS-PAGE and their protein composition compared to that of purified polyribosomes. Coomassie blue staining revealed that granules contained a majority of ribosomal proteins. However additional bands were observed, in particular at around 230–100 and 55–40 kDa (Fig 4A, highlighted with stars and in grey area) accounting for 36% of the total protein content as determined after scanning of the stained gels. We hypothesized that these bands would correspond to components of the cytoskeleton framework that might have contaminated the granule preparations. Therefore, we tested for the presence of three main cytoskeletal proteins: the neurofilaments (NF), β-actin and β-tubulin. Immunoblot analyses using antibodies to these proteins confirmed their presence (Fig 4A). This evidenced that cytoskeleton components had contaminated the granule fraction during differential centrifugations. An additional purification step was thus necessary.

Granules and polyribosomes recovered after isokinetic ultracentrifugation in sucrose density gradients (S3A Fig) were subjected to equilibrium (isopycnic) ultracentrifugation in a 10 to 60% w/v Metrizamide linear gradient [25] (S3B Fig). Both structures were detected in fractions with a calculated density of 1.295 g/cm^3^, corresponding to the buoyant density of polyribosomes [26]. Centrifugation prolonged for 48 hours did not change the particle density indicating that they have reached their equilibrium density already by 18 hours. FMRP and L7 were systematically detected in fractions corresponding to this density. Coomassie blue staining highlighted that polyribosomes and granules shared common protein profile (Fig 4B), suggesting similar protein content. More importantly, the contaminant peaks highlighted in gray in Fig 4A were no longer detected after this additional purification step. To assess the level of purification, we also performed immunoblot analyses and observed a 8-fold decrease in the signals corresponding to neurofilament, actin and tubulin, indicating that the majority of cytoskeletal contaminants were removed by the Metrizamide step.

Altogether, these data showed that the procedure described here enables the isolation of granules from mouse brain with a minimum of contaminants.

### Proteomic analyses of granules preparations

**Fig 5:**
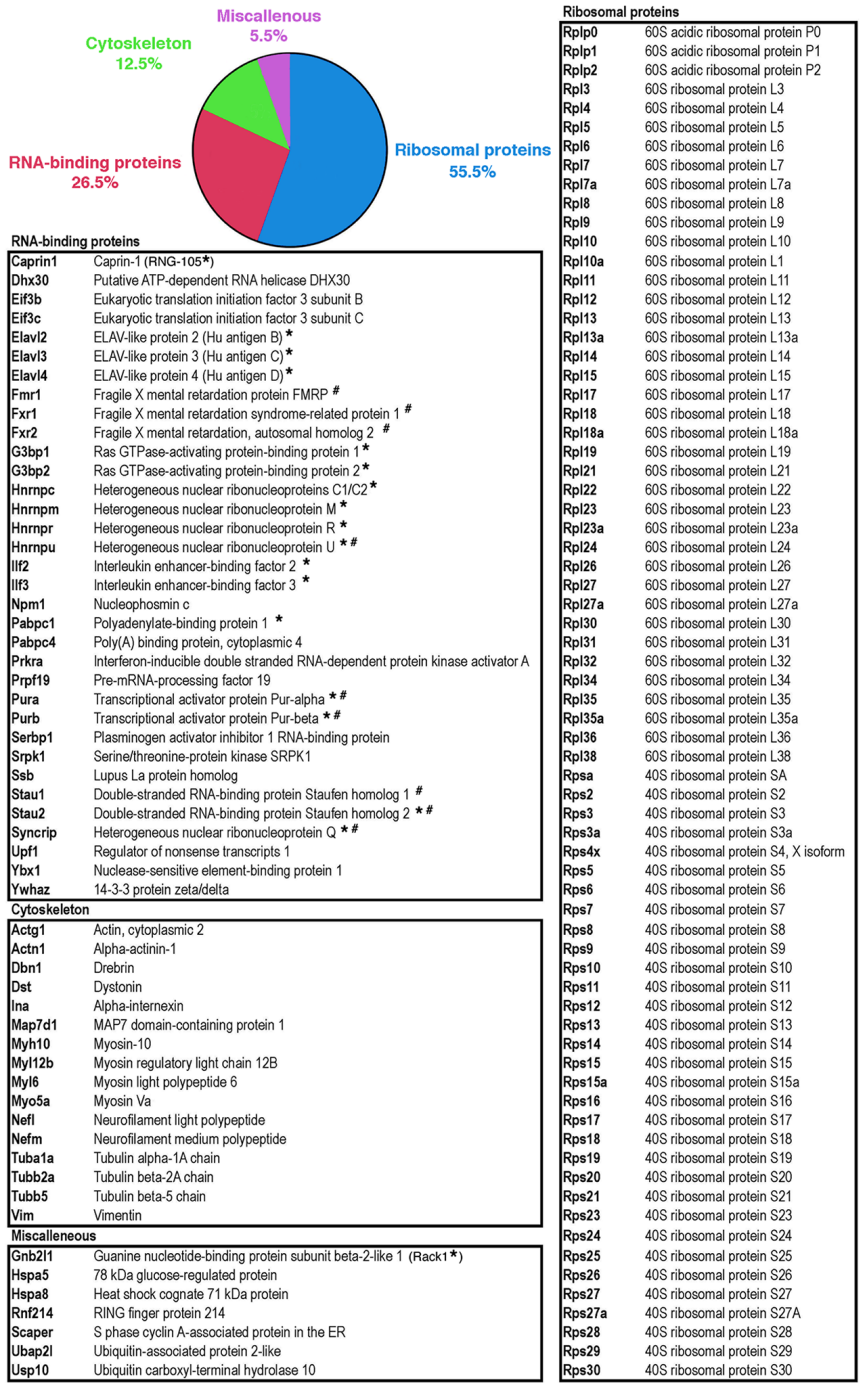
List of proteins detected in granules by mass-spectrometry. The pie chart reflects the main functional categories derived from the Gene Ontology analysis (S2 Table). Proteins labelled with * and # were previously identified in RNA granules [17,19 respectively].

To reveal the protein content of polyribosomes and granules purified by isopynic centrifugation in Metrizamide gradients, fractions were analysed by Mass Spectrometry (MS). A total of 128 proteins sharing at least 1 peptide with known proteins registered in databases were found in granules (Fig 5 and S1 Table), while 155 proteins were present in polyribosomes. Gene ontology-based pathway enrichment analyses revealed that the most significantly enriched biological processes in granules were notably ‘translational elongation’ (adjusted p-val = 2,67.10^−128^), ‘RNA processing’ (adjusted p-val = 1,38.10^−12^), ‘ribonucleoprotein complex biogenesis’ (adjusted p-val = 1,41.10^−12^) and ‘cytoskeleton-dependent intracellular transport’ (adjusted p-val = 3,40.10^−3^) (S2 Table). In addition, the most significantly enriched molecular functions in granules were ‘structural constituent of ribosome’ (adjusted p-val = 2,36.10^−98^), ‘RNA binding’ (adjusted p-val = 2,65.10^−64^), ‘translation regulator activity’ (adjusted p-val = 4,11.10^−2^) or ‘structural constituent of cytoskeleton’ (adjusted p-val = 1,42.10^−2^). The same analyses performed on the 155 proteins identified in polyribosomes highlights essentially the same classes of biological processes linked to ‘translation’, ‘ribosome’ or ‘RNA binding’ (S3 Table). However, cytoskeleton-related processes were not significantly enriched in the polyribosomal protein pool, suggesting that the main divergences observed with polyribosome and granule preparations expressed in terms of protein content, belong to this class of processes.

Based on the results of the gene ontology analysis results, we sorted the proteins detected in granules according to the following functional classes: ribosomal proteins, RNA-binding proteins and cytoskeleton-linked proteins (Fig 5). Fifty five percent of the identified proteins were core ribosomal proteins (Fig 5). RNA-binding proteins constituted the second major class of proteins in granules (26.5%). Some of them were already described as part of RNA granules, such as polyA Binding Protein 1 and 4 (PABP1), members of the ELAV family (HuB, HuC), Staufen1, Staufen2, Pur-α/β, series of heterogeneous ribonucleoproteins (hnRNPC1/C2; hnRNPR; hnRNPQ/SYNCRIP) and the Fragile X protein FMRP [16,17,19] (Fig 5). Also, several proteins known to interact directly with FMRP were detected, such as Caprin-1 [22] or the Fragile X-related proteins FXR1P and FXR2P [27]. Proteins, not previously described in RNA granules were also detected, such as the ATP-dependent helicase of the DEAD box family (DHX30) required for unwinding of mRNA during translation, and the translation initiation factors eIF3b and eIF3c. Other RNA-binding proteins were also present, for instance the splicing factors PRPF19, SRPK1 or UPF1. Finally, the presence of the axonal RNA-binding protein La suggested the presence of axonal granules in our preparations. Indeed, FMRP positive granules have been detected in axons [28,29]. Cytoskeleton-linked proteins represent 12.5% of the proteins identified in granules with a number of motor proteins (mostly myosins) and structural constituents of the cytoskeleton: actin, tubulin and neurofilaments (Fig 5).

### The Fragile X Related proteins are enriched in granules

**Fig 6:**
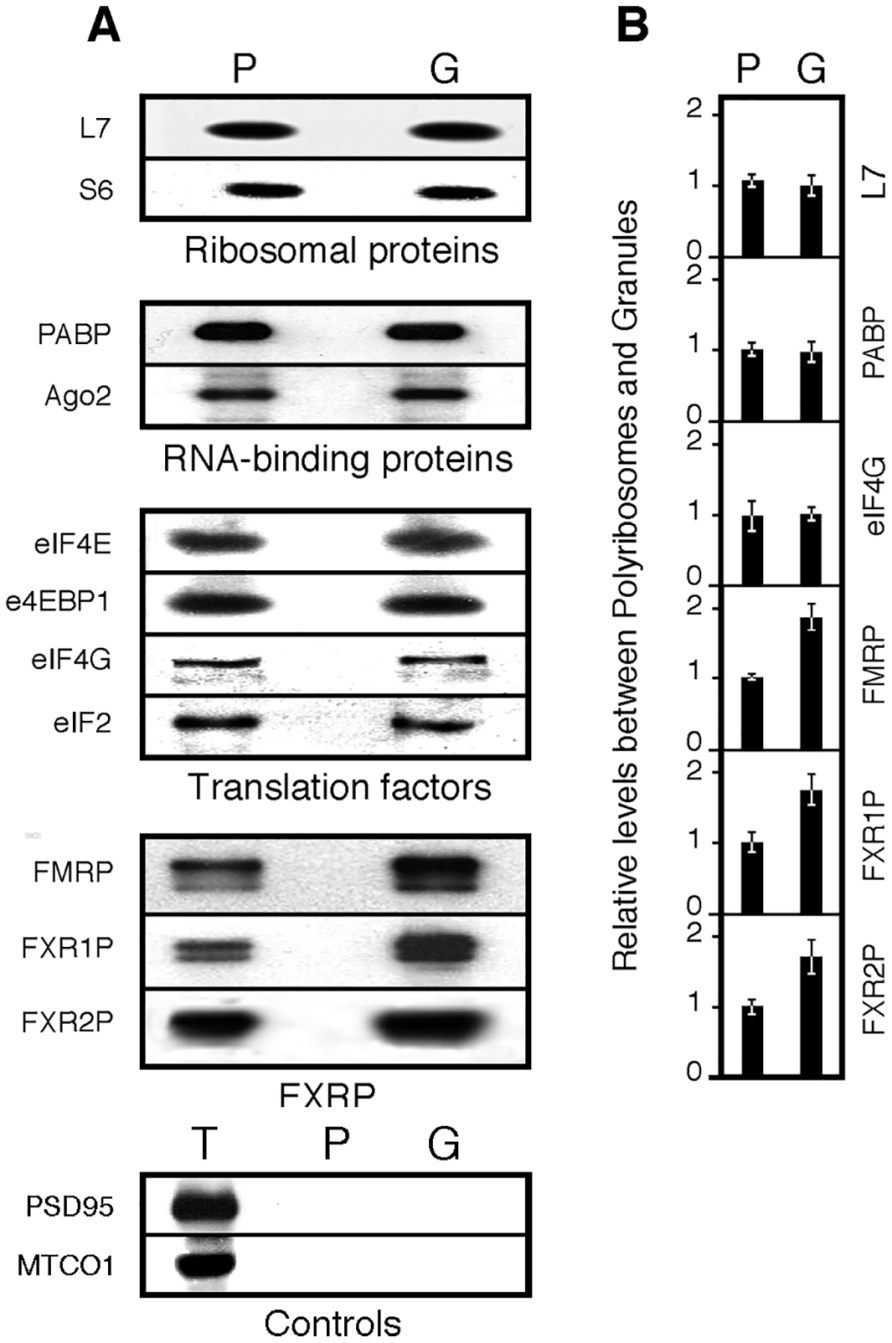
Comparative immunoblot analyses of selected proteins from polyribosomes and granules fractions. **A)** Immunoblot analyses of the steady state levels of selected proteins in polyribosomes (P) and in granules (G) and in total extract (T). **B)** Quantification of the signals. Mean values ± SEM of ratios calculated from 4 independent analyses.

Using immunoblot analyses, we further tested the presence of a series of proteins detected in granules or polyribosomes namely: ribosomal proteins, translation factors and RNA-binding proteins. Equal amounts of proteins from granules or polyribosomes were analysed; we then compared the intensities of immunoblot signals. Levels of the core ribosomal proteins L7 and S6 in preparations of polyribosomes and of granules were similar. Reports of the presence of translation factors in granules are contradictory. Krichevsky and Kosik [16] observed that the granule fraction contained trace amounts of the initiation factors eIF4E and eIF4G1. On the other hand, Kanai et al. [19] detected eIF2A, eIF2B and eIF2G while Elvira et al. [17] reported the presence of eIF4A. In view of the absence of a consensus, we tested the presence of eIF4E, eIF4EBP1/e4BP1, eIF4G1 and eIF2A. All these factors were detected with equal signal intensities in both polyribosome and granule preparations (Fig 6A).

We further tested a series of RNA-binding proteins. We confirmed that PABP1 and Ago2 were equally represented in granules and polyribosomes (Fig 6A). In addition, we quantified signal intensities for the FXR proteins, FMRP, FXR1P and FXR2P. Contrary to the other RNA-binding proteins tested, FXR proteins signals were higher in granules when compared to polyribosomes (Fig 6B). Since quantification of chemiluminescent signals is not linear [30], we first determined the optimum conditions to ascertain the increased signals of FMRP in granules. We therefore checked, using a titration assay for FMRP, that our analyses were performed in the linear signal range (S4 Fig). In the case of FMRP, we quantified by densitometry analyses an increase of 1.83 fold in granules. These results are supported by our proteomics data, as the normalized spectral counts for the Fragile X Proteins appear higher in granules than polyribosomes (S1 Table). Finally, as controls, the post-synaptic protein PSD-95 and the mitochondria encoded cytochrome C oxidase MTCO1 were not detected in either of the preparations (Fig 6A) in agreement with the proteomic analyses.

### Transcriptomic analyses of RNA granule preparations

**Fig 7:**
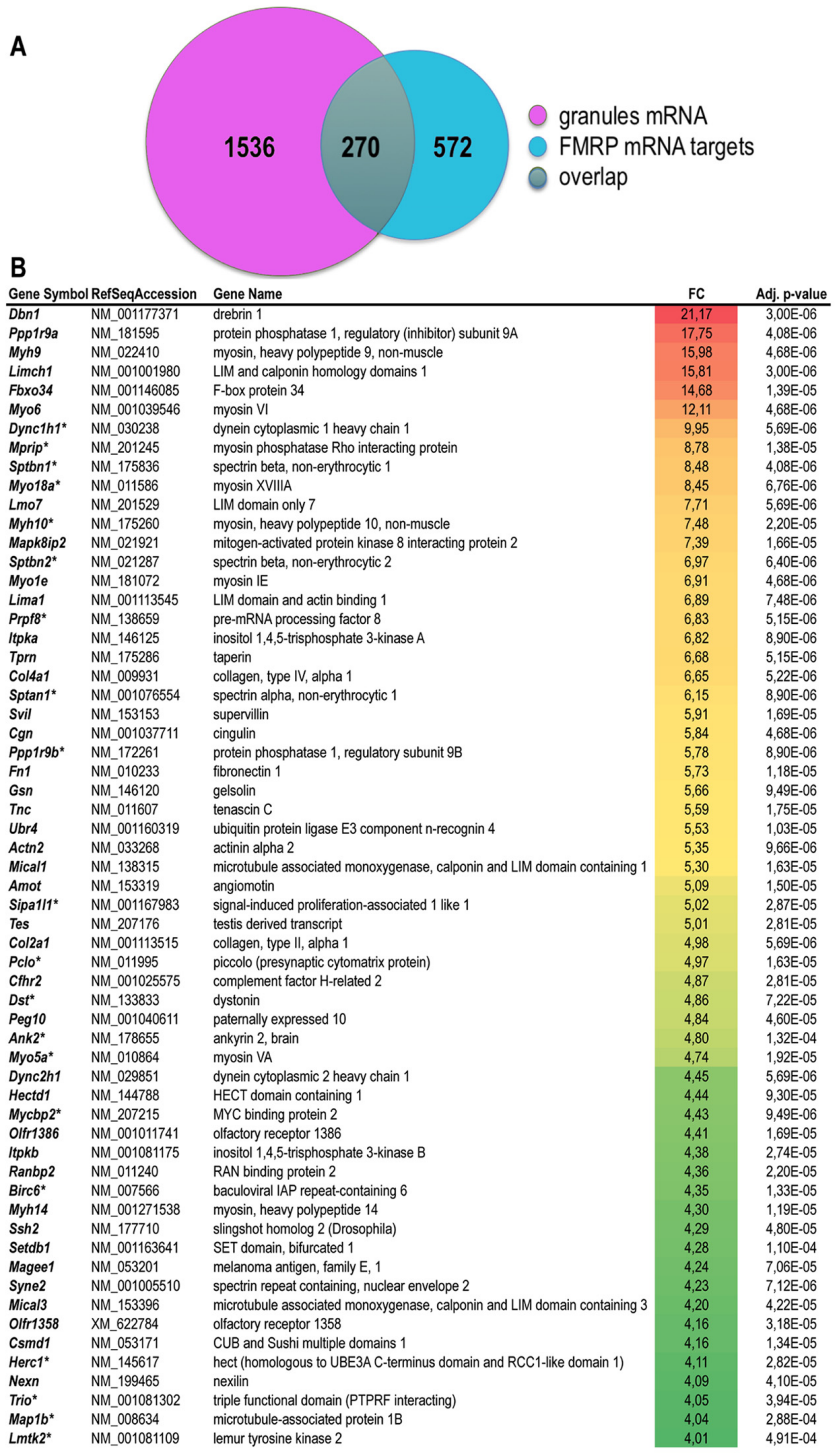
Overlap between mRNA present in granules and FMRP putative mRNA targets. **A)** Venn diagram presenting intersection between the list of mRNA present in granules listed in S4 Table (n = 1806) to the list of 842 putative FMRP mRNA targets identified by Darnell et al. [33]. **B)** List of mRNA selectively enriched in granules as compared to polyribosomes. Enrichment is calculated as the ratio in average probe intensity (fold-of-change, FC) in granules as compared to polyribosomes preparation. Only transcripts corresponding to probes displaying an enrichment above 4 (FC>2) with a significant adjusted pvalue (pval<0.002) are presented. In case of redundant probes targeting a single mRNA, data are provided for the probe providing the highest level of variation. The color code indicates the highest (red) to the lowest (green) folds of change detected. The asterisk (*) indicates putative FMRP mRNA targets identified by Darnell et al. [33].

Having studied the protein content of granules, we then wondered about their RNA content as compared to polyribosomes. To identify the RNA species present in granules, we compared RNA extracted from granules to polyribosomal RNA using whole transcriptome mouse microarrays. We focused on mRNA with signal intensities equal or higher than those from polyribosomes (Fold-of-Change (FC)>1, p-val<0.002). This corresponded to 1,806 annotated mRNAs (S4 Table) that can be assimilated to the mRNA species encountered in granules, corresponding to 7% of the total transcriptome. We performed gene ontology analysis to gain insights into the functional categories selectively over-represented in the granules (S5 Table). The biological processes enriched in granules mRNA were notably ‘actin cytoskeleton organization’ (adjusted pval = 6,16.10^−8^), ‘cytoskeleton-dependent intracellular transport’ (adjusted pval = 5,41.10^−4^), ‘neuron projection development’ (adjusted pval = 1,03.10^−5^), ‘synapse organization’ (adjusted pval = 7,54.10^−4^), ‘axonogenesis’ (adjusted pval = 5,5.10^−4^) or ‘ubiquitin-dependent protein process’ (adjusted pval = 2,16.10^−3^). In addition, the most enriched molecular functions concerned notably signal transduction pathways involving ‘GTPase regulator activity’ (adjusted pval = 7,47.10^−8^) or ‘calcium ion binding’ (adjusted pval = 8,62.10^−8^) or cytoskeleton-remodelling processes involving ‘cytoskeletal protein binding’ (adjusted pval = 1,81.10^−15^) or ‘motor activity’ (adjusted pval = 6,34.10^−4^). Finally, the most enriched cellular components included ‘cytoskeleton’ (adjusted pval = 7,21.10^−19^), ‘axon’ (adjusted pval = 1,87.10^−12^), ‘growth cone’ (adjusted pval = 3,48.10^−6^) ‘synapse’ (adjusted pval = 4,14.10^−9^) or ‘dendrite’ (adjusted pval = 7,26.10^−9^). All these processes are in line with the presumed functions of granules that are thought to transport mRNA dedicated to the regulation of synaptic development and plasticity [31,32]. Considering the crucial role of FMRP in all these processes, we sought for overlap between the list of mRNA granules, and the previously published list of putative FMRP mRNA targets [33]. Interestingly, 15% of the mRNA listed in granules has been described as putative mRNA targets of FMRP (270 out of 1806, Fig 7A and S3 Table). Also, 32% of FMRP mRNA targets (270 out of 842, Fig 7A and S4 Table) are detected in RNA granules. These data support the important role played by FMRP in these structures.

To identify the specific subset of RNA preferentially transported in RNA granules, we focused on highly enriched mRNA (FC>4) as compared to polyribosomes. Interestingly, the mRNA *Map1b* encoding the microtubule-associated protein MAP1b, a transcript known to be targeted to the dendritic arborization [32] is enriched by a factor of 4 in granules (Fig 7B). The mRNA encoding αCaMKII, a known dendritic mRNA was not detected in our analyses since it is not yet expressed in brain of young mice [34]. A series of mRNA is enriched above 10 folds in granules in particular mRNAs encoding the cytoskeleton regulator Drebrin1, the myosin motor proteins Myo6 and Myh9, and a LIM-domain containing protein Limch1 (Fig 6 and S4 Table).

### Granules and cargoes

While it has been reported that the granule size varies between 300 and 1000 nm [16], our electron microscopy results suggest that large granules might be composed of independent smaller granules of 100 to 300 nm in size (see Fig 3B to 3D). A plausible scenario would be that these independent granules fuse to form a “cargo of granules”. In the case of FMRP, transfection studies of neurons in culture have shown the presence of GFP-tagged FMRP in travelling granules [35–38]. However, vectors used previously to express RNA-binding proteins in neuron primary cultures contain strong promoters, either from the cytomegalo-(CMV) or SV40-viruses, which result in high expression levels of the GFP-tagged RNA-binding proteins. Excess expression of RNA-binding proteins, in particular of FMRP, leads to the formation of cytoplasmic *foci* corresponding to stress granules (SG) [39]. We wish to point that, morphologically speaking, SG cannot *a priori* be discriminated from neuronal granules.

**Fig 8:**
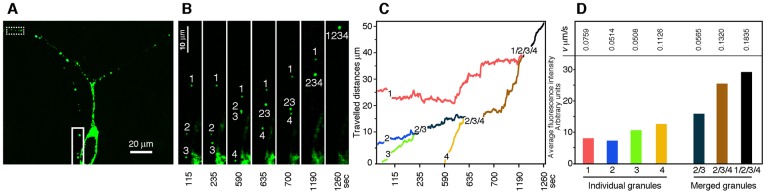
Traficking and merging of puncta into cargoes. **A**) GFP-FMRP is distributed in the somatodendritic compartment of a neuron in culture (DIV 7) transfected with the Syn-promoter driven GFP-FMRP expression vector. **B**) Insert at higher magnification, showing the movements of 4 independent puncta that finally merge into a large cargo. **C**) Trajectories and distance travelled by individual puncta across a 20 min period. **D**) Quantification of fluorescence intensity of each independent or merged puncta shown in **B**) and their respective speed.

To ensure a neurospecific and more physiological expression of FMRP, we used the pShuttle-*GFP*-*FMR1* vector under the synapsin promoter. To investigate in living neurons the dynamics and kinetics of granules, we performed time-lapse video-microscopy experiments to follow the movements of GFP-FMRP in transfected cultured rat hippocampal neurons. We observed high levels of GFP-FMRP in the soma and proximal dendritic compartments, while smaller GFP-FMRP containing puncta were distributed throughout the distal dendritic arborisation (Fig 8A). In neurons grown for 7 days in culture, we noted a high proportion (42.8%, see below) of granules showing dynamic movements. The mean speed of these moving puncta was 0.123 ± 0.005 μm/s (calculated on n = 100 granules disseminated along 4 dendritic segments from 5 neurons). Interestingly, we regularly observed coalescing of GFP-FMRP puncta into larger ones, a phenomenon that has not been reported yet. Fig 8A and 8B (and S5 Fig) illustrate this phenomenon observed in two dendritic segments. In that neuron, we followed the fate of 70 GFP-FMRP puncta, 30 of which were moving distally from the soma, while the others had oscillatory movements. Among these moving puncta, 14 merged to form larger cargo-like structures. As shown in Fig 8A and 8B, four puncta fused to form a single larger one (an additional sequence of events is shown in S5 Fig). Distances travelled by each puncta along the X and Y axes are presented in Fig 8C. We quantified the moving speed of the individual puncta over a period of 20 min. In this example (detailed in Fig 8B to 8D), we observed 3 slow puncta (number 1,2 and 3) and a faster one. Of high interest, when the slow puncta merged, they kept a slow speed (puncta 2 and 3), while when the fast puncta fused with slow one, it appeared that the former imposed its faster momentum (Fig 8D). These observations were reproduced when following a set of 5 other puncta in a more distal area of the same neuron (see dashed box in Fig 8A and details in S5 Fig). Interestingly, the intensity of the fluorescent puncta increased as they merged, although not linearly, likely because of fluorescence quenching [40]. Altogether, these results are consistent with the existence of dynamic dendritic granules that can coalesce into cargoes as they move through the dendritic arborization.

**Fig 9:**
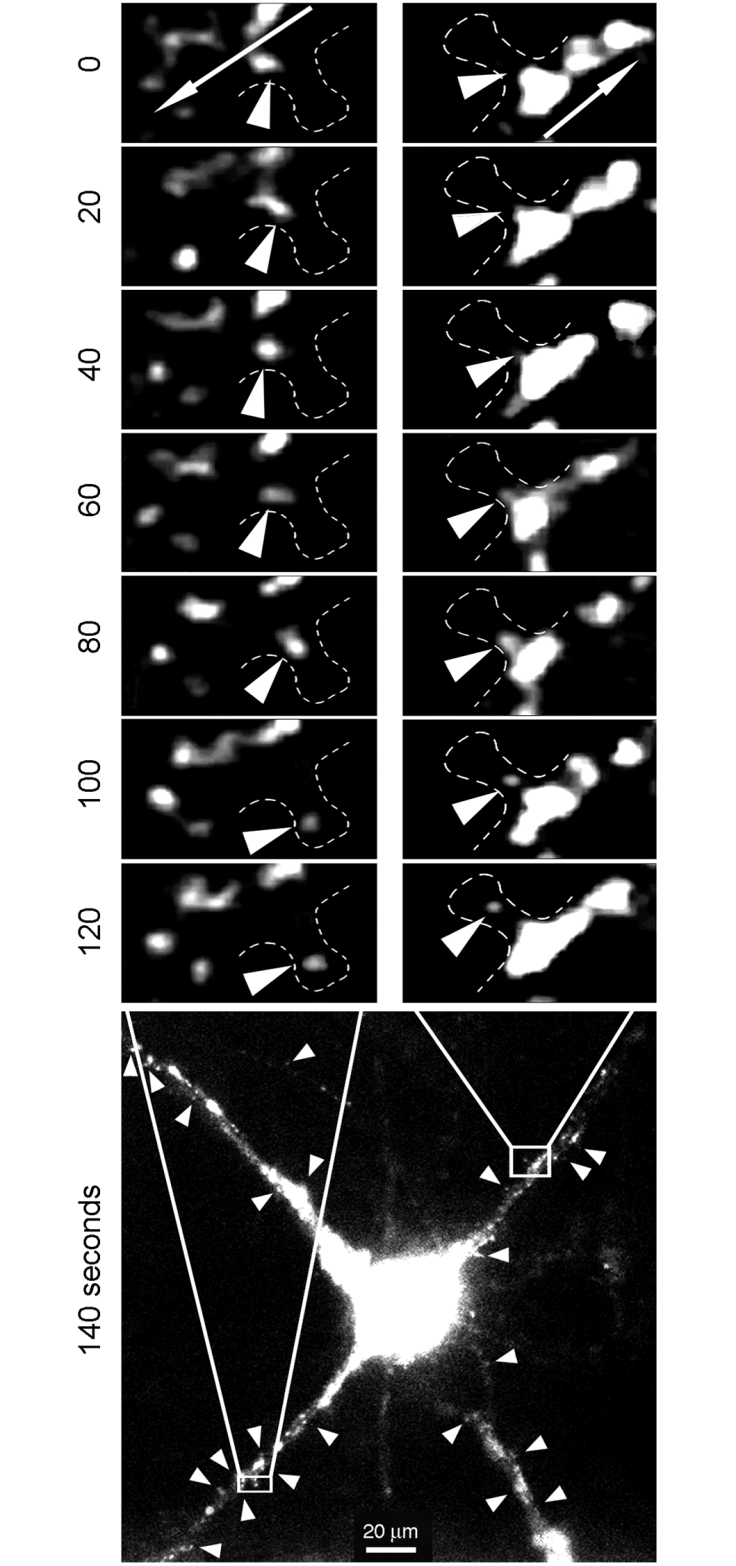
Translocation of small puncta into spines. Neurons (10–12 DIV) were transfected with the Syn-promoter driven GFP-FMRP expression vector. Time-lapse video microscopy showing small puncta emerging from large cargo-like structures and moving out of the dendrite main axis to reach the spine head. Arrows in the top insets indicate the anterograde flow movements. Arrow heads in the bottom image point to puncta emerging from the anterograde flow.

**Fig 10:**
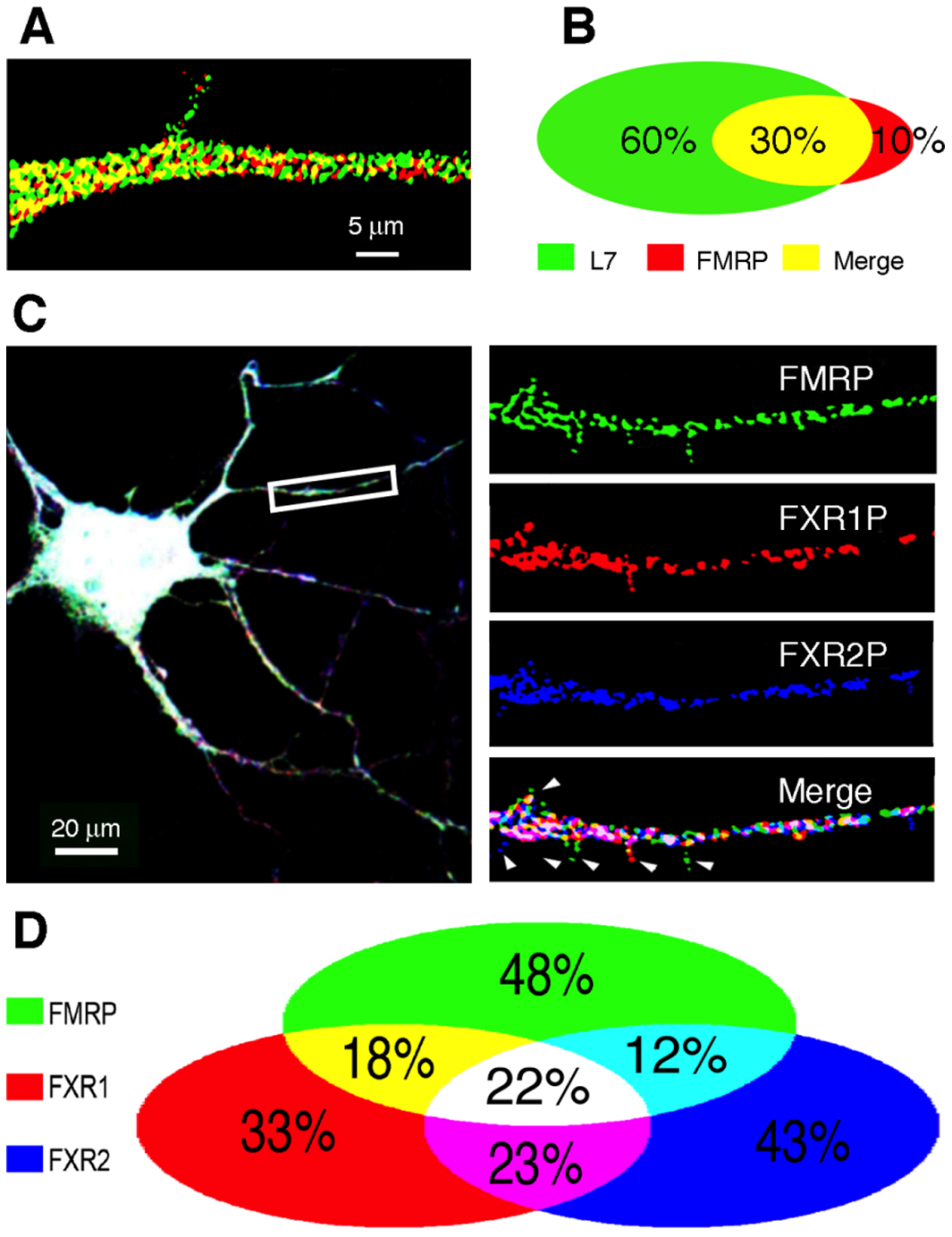
FMRP only partially co-localizes with the ribosomal protein L7 and members of the FXR family. **A)** Double-immunofluorescence of FMRP (red) and L7 (green) showing that the majority of L7 does not co-localize with FMRP, while a minority of the latter **(B)** is free of L7. **C)** Co-localization of the three FXR protein members in dendritic granules of primary hippocampal neurons. Triple immunofluorescence of FMRP, FXR1P, and FXR2P, showing that all three members are present in dendritic granules, but do not always co-localize. Arrowheads point to granules containing a single FXR protein, presumably at the spines. **D**) Quantification and distribution of the three FXR protein members in dendritic granules shown in **(C)**.

How dendritic mRNAs are delivered to the synapse remains poorly understood. For instance, how could the narrow aperture of the spine neck, less than 200 nm in the case of hippocampal neurons [41,42], allows for the passage of the large granules described by Krichevsky and Kosik [16]. A plausible scenario would be that small granules are released from cargoes enabling their passage through the spine neck to reach the synaptic compartment. Interestingly, while tracking the movements of 221 puncta in different dendrites, we found 24 that moved out of the directional flow (see examples in Fig 9 arrow heads). We noticed small puncta budding from large fluorescent clusters, translocating through the neck into the spine head. Whether these structures corresponded to FMRP associated with the whole translation apparatus could not be determined and needs further analyses that are beyond the scope of the present study. However, since Antar et al. [36] have reported that only approximately 50% of FMRP colocalizes with ribosomal RNA and as we observed that not all granules contain FMRP (Fig 3G), we decided to determine to which extent FMRP colocalizes with the ribosomal marker L7 in dendrites. We performed immunofluorescence analyses on rat hippocampal neurons in primary cultures using IgY**#**C10 anti-FMRP and anti-L7 antibodies. Deconvolution and binary merging analyses of images using the MetaMorph Software enabled us to study L7 and FMRP colocalization in 4534 granules spread over 15 dendritic segments (Fig 10A). Several observations could be made from these colocalization studies. Firstly, the population of puncta positive for L7 is 2.5 fold larger than the FMRP one. Secondly, L7 and FMRP co-localize in 30% of the puncta. Thirdly, 60% of L7 positive granules were free of FMRP. Fourthly, FMRP positive puncta free of L7 represent 10% of the total puncta (Fig 10B). This last population that contains no ribosomes may serve as supply cargoes required to replenish and feed the translation machinery at the synapse, such as the translationally silent mRNPs containing CYFIP and FMRP [43].

Immunofluorescence studies have shown the huge heterogeneity of RNA-binding proteins contained in granules. Since we did not aim at establishing an exhaustive list of the many RNA-binding protein combinations present in the granules, we limited our studies to the FXR family members. *In vitro*, all three members of this small family interact with each other to form homomers or heteromers [27] and have been detected in dendritic RNA granules after transfection of neurons with the respective expression vectors [44]. But so far, the coexistence of endogenous FMRP, FXR1P and FXR2P in the RNA granules has not been investigated. Therefore, we performed triple immunofluorescence labelling on hippocampal neurons in primary cultures (10 DIV) and studied the relative co-localization of the three endogenous FXR proteins (Fig 10C). We observed that 48% of FMRP-positive dendritic granules contain only FMRP, 43% of FXR2P-positive dendritic granules exhibit only FXR2P, while 33% of FXR1P-positive granules contain only FXR1P. Therefore, the FXR proteins are represented in various combinations, ranging from 12% to 23% of the granules that are double-labelled for two of the FXR proteins. Some granules exhibit only two members of the FXR family: 23% contain both FMRP and FXR2P or both FMRP and FXR1P and 12% show dual labelling for FXR1P and FXR2P. Interestingly, the three members of the family co-localize in only 22% of the granules (Fig 10D). These data are suggestive of the high level of diversity and heterogeneity in the composition of neuronal RNA granules.

## Discussion

We report in the present study that we have isolated and characterized RNA granules from mouse brain in their second postnatal week of life. This developmental period correspond to active synaptogenesis while FMRP is abundantly expressed in the brain [45,46]. Despite the fact that the results reported here represent only a snapshot of the granule content at a specific time of neurodevelopment, they nevertheless contribute to shed light on several points and raise several conceptual issues.

### Which proteins do make up granules?

Our high-resolution electron micrographs reveal an impressive and orderly morula-like architecture of granules resulting from densely packed polyribosomes. The proteomic analysis shows that 85.4% of the proteins identified in granules are also found in polyribosomes. While granules and polyribosomes share enriched pathways linked to ‘translation’, ‘ribosome’ and ‘RNA-binding’, cytoskeleton-linked terms appear only in RNA granule preparations. The latter data can be interpreted in two ways. On one hand, these proteins may represent trace contaminants from the cytoskeleton, which could remain in the fractions even after the Metrizamide purification step. On the other hand, they may be related to granule motility, as several myosin motors are represented. In particular, MyosinVa was previously described to transport major synaptic scaffolding proteins to dendritic spines [47].

Concerning the translation initiation and elongation factors in granules, our study documents the presence of eIF4E and eIF2a, in agreement with others [16,19]. We also report the identification a number of translation initiation factors IF3b, eIF3c, eIF4G as well as 4EBP1a and the RNA helicase DHX30. The presence in granules of the splicing regulators PRPF and SRPB1 seems a priory puzzling. However, the splicing factors and regulators, Nova-1 and Nova-2, are also present in the neuronal cytoplasm [48], where they control mRNA localization, stability and translation [49]. It is therefore plausible that PRPF and SRPB1 contribute to the localization and homeostasis of mature mRNA in neuronal cytoplasm and dendrites. Also intriguing is the presence of the argonaute family member Ago2, involved both in mRNA degradation and translation [50]. The diversity of the RNA-binding proteins identified in granules support the hypothesis that RNA granules might constitute a platform of local degradation or translation of mRNA transported at the synapse.

Using immunoblot analyses, we showed the presence of a series of proteins that were not detected by MS such as Ago2, eIF4E, e4EBP1, eIF4G, and eIF2A. The reason for this is not known. Interestingly, using MS, Elvira et al. [17] did not detect FMRP, or Pur α/β in granules while they were able to show the presence of these proteins by immunoblotting. It is possible that overrepresentation of ribosomal proteins may preclude adequate quantification or detection of other classes of proteins by MS. This might explain why some proteins are not detected. Alternatively, the relative abundances of some proteins may not be adequately reflected by normalized spectrum counts in proteomics versus immunoblotting analyses.

Proteomic analyses show that RNA granules contain both axonal and dendritic proteins. We believe that discrimination between dendritic and axonal granules is not possible when using total brain extract. The use of primary neurons grown in Campenot compartmentalized chambers [51] may allow such separation. It is not possible to conceive that all proteins described in the present study interact with each other in the same granule unit, neither it is envisioned that several mRNAs are targeted in the same granule. This is consistent with the fact that co-localization of different RNA-binding proteins in a single granule is not a general rule. Fig 10 shows that the FXRs only rarely colocalize *in vivo*, while the three members of the FXR family interact with each other *in vitro*. The same conclusions can be drawn for FMRP and Caprin1 as they interact physically *in vitro*, while little co-localization is observed in dendritic granules [22]. Therefore we propose that each granule contains a single mRNA species with its dedicated RNA-binding protein(s) ensuring translation repression.

### Which mRNAs are transported in granules?

Pathway enrichment analyses show that the transcripts present in granules are mainly associated with cytoskeleton-linked biological processes. In particular, the *Map1b* transcript, an identified FMRP target [33,52,53], is enriched at least 4-fold. Also, *Ppp1r9a* mRNA that codes the negative regulatory subunit of protein phosphatase-1 (PP1) is enriched over 16-fold in granules. The activation of PP1 is important for the induction of long-term depression (LTD), while its down regulation is required for the normal induction of long-term potentiation (LTP) of synaptic transmission [54]. Therefore, the fine-tuning of localized synthesis of PP1 regulatory subunit seems to modulate LTP and LTD locally at the synapse. LTP and LTD result from activity-dependent long-term adaptations of synaptic protein repertoire, in particular proteins involved in cytoskeleton remodelling. A strong enrichment (above 21-fold) of the F-actin and the microtubules modulator *Drebrin1* mRNAs suggests a crucial role for localized synthesis of the cognate protein. Indeed, Drebrin1 acts as a positive regulator of microtubule entry into spines [55], which plays a crucial role in synaptic function and plasticity. Also, mRNAs encoding LIM domain-containing proteins such as Limch1, Lmo7, Lima1, Mcal1 and Mcal3 are selectively enriched in granules preparations. LIM-domain containing proteins have been shown to play roles in cytoskeletal organisation, particularly at the synapse where their local synthesis would contribute to cytoskeleton remodelling, as described for LIM-domain containing kinase 1 [56].

### What can we learn about FMRP’s roles in granules?

In our granule preparations, we recovered approximately one third of the mRNA targets of FMRP described by Darnell et al. [33]. In addition, there is almost twice as much FMRP in granules than in polyribosomes. This speaks for a crucial role played by FMPR in these structures. Twenty putative mRNA targets of FMRP are enriched at least 4-fold in granules when compared to polyribosomes, indicating that these mRNAs are mainly targeted towards the dendritic compartment where they undergo localized synaptic translation regulated by FMRP, as is the case for *Map1b* [33,52,53]. The enrichment in granules of mRNA encoding motor proteins (*Dync1h1*, *Myo18a*, *Myh10*, *Myo5a*) described as targets of FMRP, raises the possibility that FMRP could address and modulate their local translation in dendrites and synapses, thereby controlling locally the movement of granules. In addition, the mRNA *Sptbn1*, *Sptbn2* and *Ank2*, the encoding members of the spectrin and ankyrin family that form a sub-membranous network involved in the regulation of synaptic stability and maintenance, are highly concentrated in granules and are putative mRNA targets of FMRP [33,57]. It is tempting to speculate that these mRNAs are addressed to the synapse and that dysregulation of their transport and local translation in the absence of FMRP contribute to the spine dysgenesis observed in Fragile X patients.

### Where and how are granules formed?

**Fig 11:**
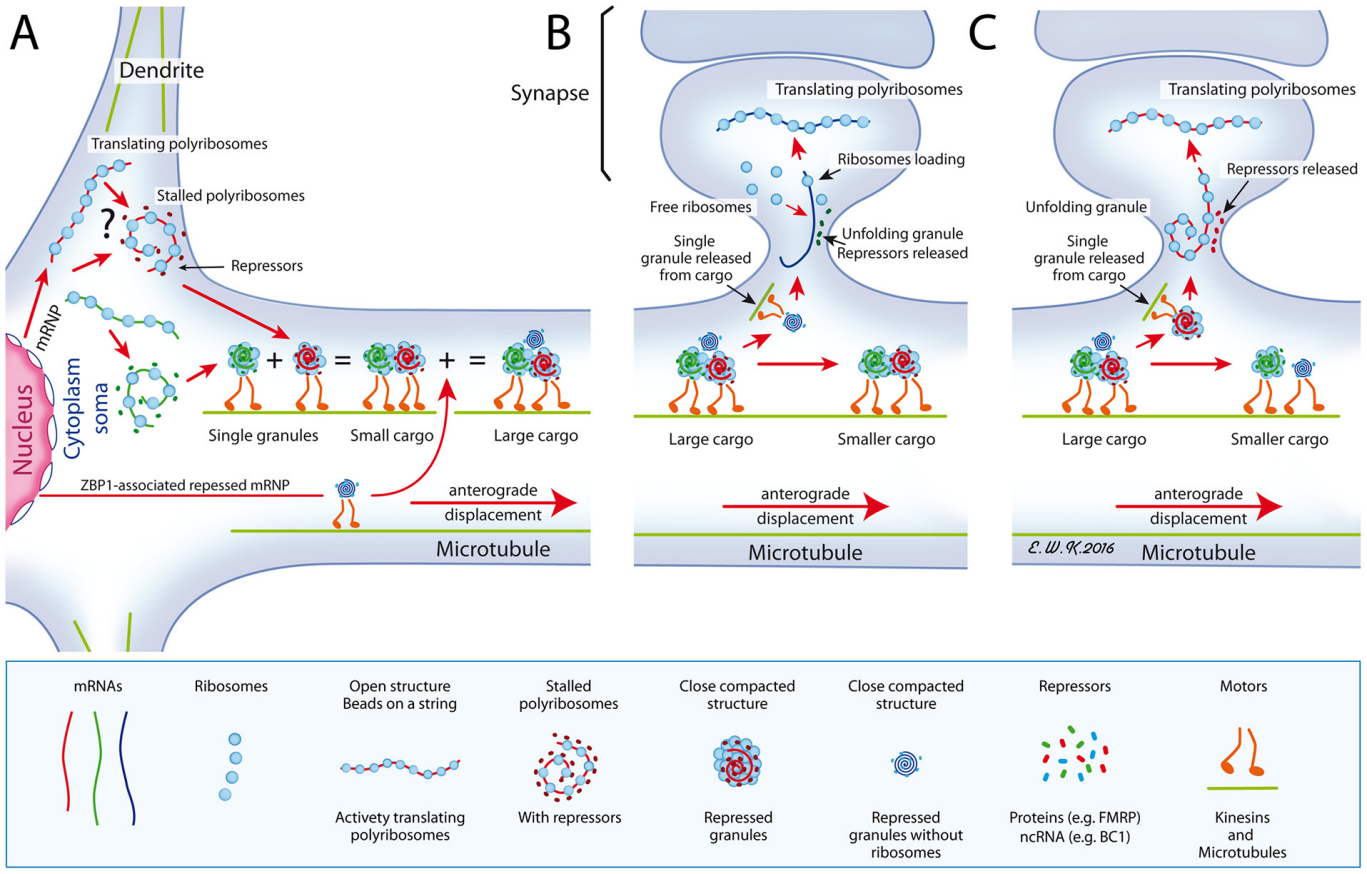
A proposed model for the formation of cargoes. **A)** Shown in the cytoplasm is the place of birth of granules that derive from stalled polyribosomes, in a close compacted structure. Alternatively (shown by an interrogation mark), polyribosomes might interact with yet unknown repressors (non coding RNA or proteins) to form granules that are transported to dendrites along microtubules, using motor proteins. Also, shown is a ZBP1-associated repressed RNP emerging from the nucleus. On their way, granules merge to form large cargoes. **B)** As large cargoes are too voluminous to cross the spine neck, individual small granules, in this instance the ZBP1-RNP devoid of ribosomes, emerge from cargoes and penetrate the narrow spine neck to join free ribosomes present in the post-synaptic area. **C)** A small granule containing the whole translation apparatus is translocated to the spine. In each instances, the close structure unfolds upon stimuli after releasing repressor molecules, either protein or RNA, to allow translation of its carried mRNA. Red lines with arrows indicate movements of the structures.

We postulate that a class of granules emerge from stalled somatic polyribosomes [33,58] to form independent small RNA granules that are transported on microtubules, as is the case for granules carrying FMRP [36,59]. These small granules merge to form larger granules through a mechanism that has yet to be uncovered. Therefore, we propose the concept of RNA cargoes in which individual granules are recruited en route (Fig 11). Using time-lapse video microscopy, we observed small puncta coalescing into larger ones, suggesting that the so-called RNA granules can fuse into large cargoes of RNA granule entities. Forward movements display an average velocity of 0.123 ± 0.005 μm/sec, compatible with the reported speeds for granules labelled for GFP-Staufen (0.1 μm/sec [60]) and GFP-Pur-α (0.10–0.12 μm/sec [19]). Our tracking observation suggest that fast granules merging with slower granules, can impose their kinetics to the latter. This implies that each granule is under the control of a motor that determines its speed, and that once granules have merged, one specific motor determines the speed of the cargo. Importantly, FMRP interacts physically with members of the kinesin family [38,61] and immunoprecipitation studies have shown that it is present in complex brain structures containing the actin-based motor protein myosin Va and dynein [62] as well as KIF5A [19]. Furthermore, we detected in granules enriched mRNAs encoding motor proteins: myosins (*Myo1e*, *5a*, *6*, *18a and Myh9*, *10*, *14*) and dynein *Dync1h1*. Myo5a is required for the transport of FMRP mRNP [63] and associates with mRNP present in peripheral axons [64]. Myosin 10 is a motor involved in the formation of filopodia and development of dendritic spines and synapses in hippocampal neurons [65]. These data suggest that individual granules may be carried by different motors cooperating for their transport in the neuronal arborization [66,67]. It is also possible that granules switch from microtubule to actin tracks to join other travelling granules, the nature of the motor of that switch being determined by the motor load [68].

### How is the mRNA maintained as translationally silent in granules?

The resistance of granules to EDTA and RNase treatments strongly argues for a role of protein-protein interactions, rather than by protein-RNA links in maintaining the granule structure. This network of protein-protein interactions is dissociated by high-salt conditions, that remove certain RNA-binding proteins from brain polyribosomes [22]. Also, the association of these RNA-binding proteins with polyribosomes is sensitive to the anionic detergent deoxycholate that preserves only core polyribosomal proteins [20,21,23]. These observations support the hypothesis that dissociation of granules is conditioned by the removal of RNA-binding proteins, such as FMRP that is engaged into protein-protein interactions. This leads to the unfolding of the densely packed granule structure, which in turn can evolve towards the polyribosomes open structure (see below). Importantly, the close and compact structures described in the present study, have not been described in isolated polyribosomes that appear either as ring-shaped forms collapsed into double row structures or as linear polyribosomes densely packed into 3D helices [69,70]. Also, they have not been observed in organelles containing polyribosomes such as the Vault particles [71].

The present study provides converging evidence that mRNA present in granules is blocked once the translation initiation complex is formed. Firstly, the EM micrographs of granule preparations present striking similarities with those illustrating complexes of stalled polyribosomes [33]. Secondly, the compact-close structure of stalled polyribosomes is maintained even after EDTA or micrococal nuclease treatments [33], as is the case for the granules described in the present study. Thirdly, we confirmed by proteomic and immunoblotting analyses the presence in granules of several members of the translation initiation complex. These results are in line with a recent study performed in primary neuronal cultures revealing the presence of ribosome-bound nascent polypeptide chains budding from neuronal RNA granules, together with the RNA-binding proteins Staufen 2 and FMRP [58]. This suggests that neuronal mRNAs are transported in granules in the form of packed polyribosomes stalled after the first round of translation elongation.

A number of studies have shown that high levels of exogenous FMRP induce translation repression of reporter transcripts [39,72,73]. In addition, for the wide majority of well-characterized FMRP mRNA targets, the levels of the cognate protein are increased in the absence of FMRP. Finally, a landmark study from Darnell *et al*. [33] unveiled ribosomal stalling as an unexpected mode for translation repression by FMRP. We envision that FMRP functions as a translational repressor in RNA granules to prevent ectopic translation of its target mRNA during transport. Similarly, the orthologs FXR1P and FXR2P are enriched in granules in which they can be detected by immuno-electron microscopy. However their specific roles in translation regulation have been neglected so far.

### How a large granule can cross the narrow neck of dendritic spines?

Because we showed that ribosomes are the basic unit of the granules, we postulate that the granule size is function of the number of ribosomes, presumably in relation itself to the length of the transported mRNA. Indeed, it has been hypothesized by Schuman et al. [74] that neuronal RNA granules exist as single entities and that each granule contains and transports a single mRNA. It results that, the longer the mRNA, the larger is the granule. The average size of a dendritic spine neck (200 nm [41,42]) represents a spatial constraint such that large RNA granules or cargoes cannot enter the synapse. In fact, as shown by our time-lapse video microscopy, small granules might bud from large cargoes and pass through the neck into the spine. These observations lead us to speculate that once located in the spine, the translation apparatus is reactivated following adequate stimulations; in turn, the RNA-binding proteins or other repressors are released from the complex and are degraded. In the case of FMRP, it has been reported that mGluR activation leads to FMRP loss at the synapse [36] due to rapid degradation by the ubiquitin-proteasome pathway [75,76].

While our model predicts that a specific class of granules emerges from stalled polyribosomes, it does not rule out that other granular structures are formed in distinct cellular compartments. Indeed, the non-coding BC1 RNA, a translation repressor [13], is predominantly detected in dendritic granules and does not transit in polyribosomes [77]. Also, some granules might correspond to travelling repressed mRNPs, such as the ZBP1-associated mRNA complexes, that are formed in the nucleus [78,79].

### Conclusions

The study of neuronal RNA granules have driven considerable attention since the discovery that the RNA-binding proteins FMRP, TDP-43, and SMN, respectively associated with Fragile X Syndrome [80], amyotrophic lateral sclerosis [81,82] and spinal muscular atrophy [83,84], are components of RNA granules. The primary morphological abnormality observed in the brain of Fragile X patients, is the presence of immature-looking dendritic spines [85] most likely resulting from alteration in the cytoskeleton architecture, as a consequence of defects in transport and translation of specific mRNA at the synapse. In the present study, we present evidence that travelling RNA granules are as heterogeneous as perhaps the whole extrasomatic transcriptome, and we hope that our approach will enable the in-depth study of dysregulations of RNA granules transport in neuropathologies. In the case of the Fragile X syndrome, we believe that the CLIP-RNAseq approach on granules preparations will enable to determine the precise nature of FMRP RNA targets addressed to the synapse, contributing to precisely reveal the defective mRNA involved in the syndrome.

## Materials and Methods

### Animals and neuronal primary cultures

C57BL/6J mice were bred in our animal facility and treated following the guidelines of the Canadian Council on Animal Care. The ethics committee of Université Laval has approved all procedures used in this study.

Hippocampal neuron cultures were prepared from neonatal rats as described [86]. Briefly, hippocampi were dissected out of postnatal day 1 rats. After dissociation, cells were washed, centrifuged and plated on poly-D-lysine-coated Aclar coverslips. Growth media consisted of Neurobasal supplemented with B27, penicillin/streptomycin (50 U/ml; 50 μg/ml), and 0.5 mM Glutamax (Thermo Fischer Scientific). Cytosine arabinofuranoside (Ara-C, 5 μM, Sigma) was added 2 days after plating to reduce the number of glial cells. After 4 days invitro, half of the growth medium was replaced with medium without Ara-C. Neurons were cultured between 7 and 13 DIV before use.

### Preparation of brain polyribosomes and granules

Total brain cytoplasmic extracts were prepared from 10 days old C57BL/6J mice, using two different methods.

#### Extraction in buffer without detergent

One brain (0.35 g) was homogenized in 3 ml of a buffer containing 20 mM Hepes (pH 7.4), 140 mM potassium acetate, 1 mM magnesium acetate, and 1 mM EGTA, supplemented with protease and RNase inhibitors [18,19]. A third of the volume of the post-mitochondrial supernatant was analysed by sedimentation velocity in sucrose gradients.

#### Extraction in buffer with detergent

Three brains were homogenized in 9 ml of a buffer containing 20 mM Tris-HCl (pH 7.4), 150 mM NaCl, 2.5 mM MgCl_2_, 1 mM DTT, 10 U/ml RNasine (Pharmacia), protease inhibitors (Mini Complete, Roche) and 50 μg/ml cycloheximide. A post-mitochondrial supernatant was obtained by centrifuging the homogenate at 9 000 g for 15 min. To concentrate polyribosomes, 1% Igepal CA-630 (Sigma Aldrich) was added to the post-mitochondrial supernatant and 8 ml of the solution were layered over a 2 ml pad made of 60% (w/v) sucrose in a 11 ml tube and centrifuged in a Beckman SW41 rotor at 34 000 rpm (avg 146 000g) for 2 hours at 4°C. The pellets were then resuspended in a 20 mM Tris-HCl pH 7.4, 150 mM NaCl, 2.5 mM MgCl_2_ buffer for isokinetic analyses on sucrose gradients [20].

### Isokinetic and isopycnic centrifugations

#### Sucrose density gradients

Linear sucrose density gradients were generated using the Gradient Mate (BioComp, Canada) according to the manufacturer’s instructions. Unless specified in the results section, polyribosomes were analysed on 15–60% (w/v) isokinetic sucrose gradients made up in 20 mM Tris-HCl pH 7.4, 150 mM NaCl, 2.5 mM MgCl2. After centrifugation in a Beckman SW41 rotor for 2 hours at 34 000 rpm (avg 146 000g) and 4°C, gradients were fractionated by unloading from the top using the Auto Densi-Flow (Buchler) connected to an ISCO UA-5 flow-through spectrophotometer set at 254 nm. Each fraction was precipitated overnight at –20°C after addition of 2 volumes of ethanol. The precipitated material was collected by centrifugation in a microfuge at 12 000 rpm for 20 min and solubilized in SDS-sample buffer before immunoblot analyses [20].

#### Metrizamide gradients

Fractions containing polyribosomes and granules obtained after isokinetic centrifugation in sucrose gradients were diluted and/or resuspended in 20 mM Tris-HCl pH 7.4, 150 mM NaCl, 2.5 mM MgCl2 and centrifuged in a SW60 Ti rotor at 54 000 rpm (avg 300 000 g) at 4°C. Pellets were resuspended in 20 mM Tris-HCl pH 7.4, 150 mM NaCl, 2.5 mM MgCl2 and 0.8 ml samples layered over 3.8 ml preformed, linear 20–60% (w/v) Metrizamide, a non-ionic radiopaque contrast agent (Nyegaard, Oslo) gradients and centrifuged to equilibrium for 18 hrs at 43 000 rpm (avg 190 000 g) in a Beckman SW60.1 rotor. The bottom of each tube was punctured and 0.3 ml fractions were collected. Densities were determined from refractive index measurements [87,88] using an Erma Refractometer (Tokyo, Japan).

### Protein analyses

Protein concentration was determined using the Bradford method after TCA precipitation of the extracted proteins and resolubilization in 0.2 N NaOH followed by neutralization with 0.2 N HCl. To adjust with accuracy the quantities of polyribosomal and granules protein loaded on SDS-PAGE, gels were stained with Coomassie Brilliant blue, scanned and total stained peaks integrated using the ImageJ program, and the loaded volumes consequently adjusted.

Protein were analysed by SDS-PAGE (10% acrylamide) and the resolved proteins stained with Coomassie brilliant blue. Resolved proteins were also transferred onto 0.45 μm nitrocellulose membranes (BioRad) and processed for immune-detection after blocking in 5% non-fat dry milk in PBS. The following primary antibodies were used: chicken anti-FMRP #C10 (dil 1:2000; [22]), mouse anti-FMRP mAb1C3 (1:2000; [89]), rabbit anti-FXR1P #ML13 (1:25000 [39]). Mouse anti-FXR2P mAb42 (1:2000), mouse anti-MTCO1 (1:2000, ab7291), rabbit anti-PSD95 (1:5000), mouse anti-α–Tubulin (1:5000, ab7291) were purchased from Abcam. Rabbit anti-L7 ribosomal protein (1:10000) was from Novus Biological; rabbit anti-S6 ribosomal protein (1:2000), rabbit anti-PABP1 (1:1000), rabbit anti-eIF4G (1:1000), rabbit anti-eIF2A (1:1000) from Cell Signaling. Rabbit polyclonal anti-NeuroFilaments (1:1000, NF18934-1-AP) was from Proteintech. Mouse anti-Ago (1:500) from Upstate, rabbit anti-eIF4E (1:1000), and rabbit anti-e4EBP1 (1:1000) from Assay BioTech, and mouse anti-actin JLA-20 (1:1000) was obtained from Developmental Studies Hybridoma Bank (Iowa City, IA). Detection of bound antibodies was performed with HRP-coupled goat secondary antibodies to mouse or chicken or rabbit (Immunoresearch) followed by ECL reaction (Perkin Elmer) and exposure to Fuji X-ray films. Quantitation of signals was performed after scanning the films and analyses using the ImageJ software.

### Immunofluorescence and live imaging

Hippocampal neurons grown on coverslips for 10-12DIV were processed for immunofluorescence. Rabbit anti-FXR1P #ML13, chicken anti-FMRP #C10, mouse anti-FXR2 and rabbit polyclonal anti-L7 primary antibodies were used at 10 times less than the dilutions used for immunoblot analyses (see above), followed by Alexa secondary antibodies (green, red, blue respectively). Samples were mounted in Prolong Gold medium (Invitrogen). Images were captured using a Zeiss LSM 510 confocal microscope and a 63x (1.4 NA) objective and analysed using the MetaMorph Software.

For time-lapse videomicroscopy experiments, hippocampal cultures were transfected using Lipofectamine as described [86] with the pShuttle/*GFP-FMR1* under the synapsin promotor, ensuring a neurospecific expression of FMRP. The vector was engineered by subcloning GFP-*FMR1* cDNA from pGFP-C2/FMRP construct [38] into the pShuttle (Stratagene) downstream of the synapsin promotor. Cells were imaged at 36°C–37°C in an open perfusion (0.2–0.5 ml/min) Qe-1 RC-41LP chamber (Warner Instruments) mounted onto a Zeiss Axiovert inverted microscope equipped with a 63x (1.4 NA) or 100x (1.3 NA) objectives. Images were captured with a cooled CCD camera (Cool Snap HQ, Roper Scientific) every 5 sec for 20 min. The intensities of fluorescence along the processes of each neuron were measured with a user-defined threshold with the MetaMorph software (Universal Imaging). The mean movements of granules were measured on a 20 minutes scale using the SpotTracker plugging of the ImageJ software (NIH, Bethesda).

### Electron microscopy

#### Negative staining

Ten μl of polyribosome fraction were deposited on discharged, collodion-coated gold grids for 5 min. After washing the sample with ddH_2_O, the grid was treated with 2% uranyl acetate for 2 seconds, the solution was removed, the grid washed with H_2_0 and air-dried.

#### Immunogold labelling

The pelleted material obtained after isokinetic centrifugation was resuspended and concentrated by ultracentrifugation at 54 000 rpm (avg 300 000g) for 2 hours in 0.8 ml polyallomer tubes fitted in the Beckman SW60 Ti rotor using a Delrin adapter (Seton, CA). The obtained pellets were fixed *in situ* with 4% paraformaldehyde in PBS for 18 h at 4°C, dispersed in 3% low-gelling temperature agarose (37°C) in 0.1 M cacodylate buffer, pH 7.3. After cooling the solidified pellets were cut into blocks, dehydrated in a series of increasing ethanol concentrations, and then embedded in LR-White resin (London Resin Comp. UK). Ultrathin sections (70–90 nm) on formvar coated gold grids were preblocked in PBS-0.1% Tween 20 (PBST) containing 5% dry milk and reacted for 2 hours with rabbit anti-L7 and anti-S6 polyclonal antibodies, mouse mAb1C3 against FMRP, rabbit polyclonal ML13 against FXR1P, or mouse mAb42 against FXR2P. After washes with PBST, the grids were incubated with gold-labeled anti rabbit (15 nm) or mouse (5 nm) IgGs (BBI, Cedarlane, ON, Canada) in PBST-5% dry milk for 1 hour at room temperature, washed with PBST, and postfixed with 1% glutaraldehyde. Sections were stained with uranyl acetate and lead nitrate and examined in a JEOL 1200EX electron microscope at 80 kV.

### Mass spectrometry and protein identification

Analyses were performed at the McGill University-Genome Québec Innovation Centre facility (Montréal, Canada). Fourty μg of proteins from granules or polyribosomes were run on an 11% acrylamide SDS-PAGE. Gels were stained with Coomassie blue and twenty gel slices per lane were excised. Proteins were digested *in situ* with trypsin, and the resulting tryptic peptides analysed by tandem mass spectrometry. All MS/MS spectra were analysed using Mascot [90] and X!Tandem [91]. Mascot was set up to search mouse proteome (Mus musculus released 2009/11/24) assuming non-specific digestion by trypsin. Mascot and X!Tandem were searched with a tolerance of 0.50 Da for both fragment and parent ion mass. Iodoacetamide derivatives of cysteine were specified in Mascot and X!Tandem as fixed modifications, while deamidation of asparagine and glutamine, methyl ester of aspartic acid and glutamic acid, methylation of cysteine and oxidation of methionine were specified in X! Tandem and Mascot as variable modifications. Scaffold software was used to validate MS/MS based peptide and protein identifications [92]. Peptide Prophet algorithm was used for peptide identification with a 95,0% confidence [93]. Protein identifications were accepted on the basis of at least 1 identified peptides. Protein probabilities were assigned by the Protein Prophet algorithm [94]. Proteins that contained similar peptides and could not be differentiated based on MS/MS analysis alone were grouped to satisfy the principles of parsimony. To provide a semi-quantitative appreciation of protein abundance, the dedicated function of Scaffold software was used to normalize individual protein spectral counts data to the total spectral counts for each MS samples. The data presented in S1 Table are scaled accordingly.

### Microarray analyses

Total RNA from polyribosomes and granules from wild-type mice (n = 3 biological replicates) were extracted using Trizol LS (Invitrogen) according to the manufacturer's instructions. After DNase I digestion (Qiagen), RNA was further purified using RNeasy Mini kit (Qiagen). Quality and concentration of extracted RNA was measured using the 2100-Bioanalyzer (Agilent Technologies, Palo Alto, CA, USA) with the RNA PicoLab Chip (Agilent Technologies). Only high-quality RNA (RIN over 8) was used for RNA amplification.

Thereafter, RNA was subjected to two rounds by T7 amplification using the RiboAmp HSPlus RNA Amplification Kit (Life Science, Foster City, CA, USA), purified and quantified using NanoDrop ND-1000 spectrophotometer (NanoDrop, Wilmington, DE, USA). Antisense-RNA (aRNA) samples were labelled with Cy3 or Cy5 using the Universal Linkage System (ULS) kit (Kreatech Diagnostic, Amsterdam, Netherlands) and 825 ng of labelled aRNA were hybridized on the SurePrint G3 Mouse GE 8x60K Microarray kit (Agilent) in a two-color dye-swap design in a hybridization oven for 17 h at 65°C. A simple direct comparison between treatments was done in full dye swap. Microarrays slides were then washed and scanned with the PowerScanner (Tecan, Männedorf, Switzerland) and analysed with the Array-Pro Analyzer software (MediaCybernetics, Bethesda, MD, USA).

Microarray data were pre-processed and analysed using the FlexArray 1.6.1 (http://genomequebec.mcgill.ca/FlexArray). Raw data correction consisted of a Lowess intra-array normalization and Quantile inter-array normalization. Statistically significant variations were detected using Limma (Bioconductor). Multiple hypotheses testing correction was done using the Benjamini-Hochberg procedure [95]. Differences in gene expression were evaluated by calculating the fold of change (FC) of signal intensity in granules preparations versus polyribosomal preparations. FC were considered significant when: i) net signal intensity is significantly over background in all arrays (technical and biological replicates); ii) the cut-off adjusted p-value <0.002 and iii) fold change reaches at least 1 (log2FC>0). Positive signal threshold was determined for each array from the average background value plus two standard deviations.

### Gene ontology analyses

Gene ontology-based pathway analyses and downstream exploitation of protein and gene lists were performed using the freely available DAVID bioinformatics resources [96,97].

## Supporting Information

S1 Fig FXR1P and FXR2P are also present in granules as detected by immunogold labelling (arrow heads for FXR1P; 15 nm and FXR2P; 5 nm) on LR-White resin embedded thin sections. (TIF)

S2 Fig Frequency distribution of gold-FMRP in 100 independent granules after immunogold-labelling on LR-White resin embedded sections. (TIF)

S3 Fig Isolation of polyribosome and RNA granule populations. **A**) Schematic diagram of the steps used for the purification of polyribosomes and granules. **B**) Isopycnic centrifugation on Metrizamide gradients reveals that granules and polyribosomes have identical density properties.
(TIF)

S4 Fig Titration assay to determine the steady state levels of FMRP in polyribosomes and granules. Ten and 20 μg of proteins from purified polyribosomes (P) and granules (G) were analysed in parallel by immunoblotting with IgY#C10, and the membrane exposed for different times to X ray films. Densitometric analyses enable to calculate that the ratio of 2 fold is observed with 10 μg loading and with a 20 sec exposure (underlined in the right panel). Quantitative analyses shown in Fig 6B in the main text, was performed under these conditions.
(TIF)

S5 Fig Cargoes are formed by progressive coalescence of smaller puncta as observed by time-lapse video microscopy. **A**) Presented here is the second boxed area in Fig 8A in the main text (Top left) showing the movements of 5 independent granules. **B**) Individual trajectories versus time of each of these granules.
(TIF)

S1 Table Normalized spectrum counts for proteins identified by mass-spectrometry in granules and polyribosomes-enriched preparations. For each protein, individual spectrum counts normalized to the total spectrum counts are presented and ordered from the highest (red) to the lowest (green) values in granules preparations. A value of 0 indicates that the protein was not detected. Official protein symbol and name, UniProt accession number and molecular weight (MW) are provided.
(PDF)

S2 Table Gene Ontology analysis to reveal functional categories enriched in proteins identified in granules (listed in Fig 5 and S1 Table). Analyses were performed using the DAVID bioinformatics resources with the granule list as input. GOTERM categories: biological processes, cellular components and molecular functions with a significant Benjamini’s adjusted pvalue (p<0.05) are presented.
(PDF)

S3 Table Gene Ontology analysis to reveal functional categories enriched in proteins identified in polyribosomes (listed in S1 Table). Analysis was performed using the DAVID bioinformatics resources with the granule list as input. GOTERM categories: biological processes, cellular components and molecular functions with a significant Benjamini’s adjusted pvalue (p<0.05) are presented.
(PDF)

S4 Table List of mRNA detected in granules. For each transcript, probe and gene ID are provided. Enrichment is calculated as the logarithm (base 2) of the ratio in average probe intensity (log2FC) in granules as compared to polyribosomes preparation. Transcripts corresponding to probes displaying an enrichment equals or above 1 (FC>1; log2(FC)>0) with a significant adjusted pvalue (pval<0.002) are presented. In case of redundant probes targeting a single mRNA, data are provided for the probe providing the highest level of variation. The colour code indicates the highest (red) to the lowest (green) folds of change detected. Putative FMRP mRNA targets identified in Darnell et al. [33] are highlighted in green.
(PDF)

S5 Table Gene Ontology analysis to reveal functional categories enriched in mRNA of the granules. Analysis was performed using DAVID bioinformatics resources with the list of transcripts with FC>1 and pval<0.002 (mRNA as abundant or more abundant in granules than polyribosomes, see S3 Table). GOTERM categories (biological processes, cellular components and molecular functions) with a significant Benjamini’s adjusted pvalue (p<0.05) are presented.
(PDF)
