# Supplementary figures and images for: Tracking the Fragile X Mental Retardation Protein in a Highly Ordered Neuronal RiboNucleoParticles Population: A Link between Stalled Polyribosomes and RNA Granules
Source: PLoS Genet. 2016 Jul 27;12(7):e1006192. doi: 10.1371/journal.pgen.1006192 (PMC4963131; doi:10.1371/journal.pgen.1006192)

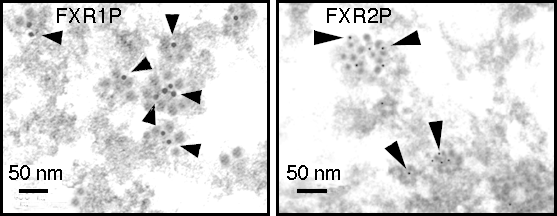

Supplement: S1 Fig — (TIF) [file pgen.1006192.s001.tif]

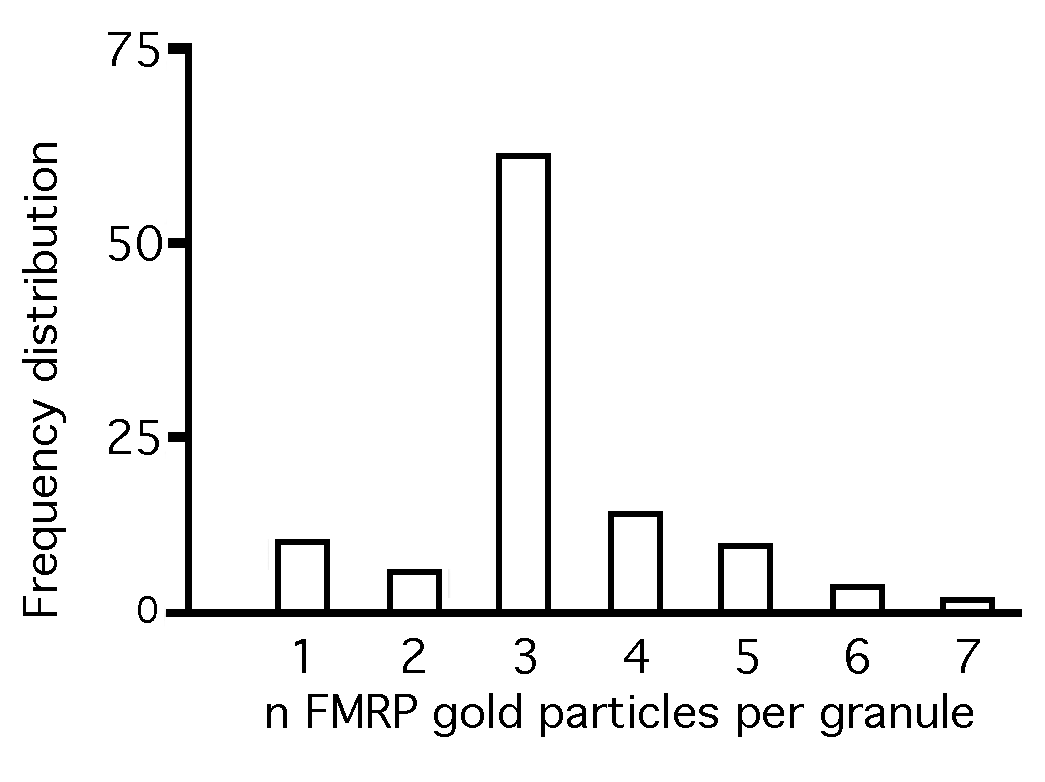

Supplement: S2 Fig — (TIF) [file pgen.1006192.s002.tif]

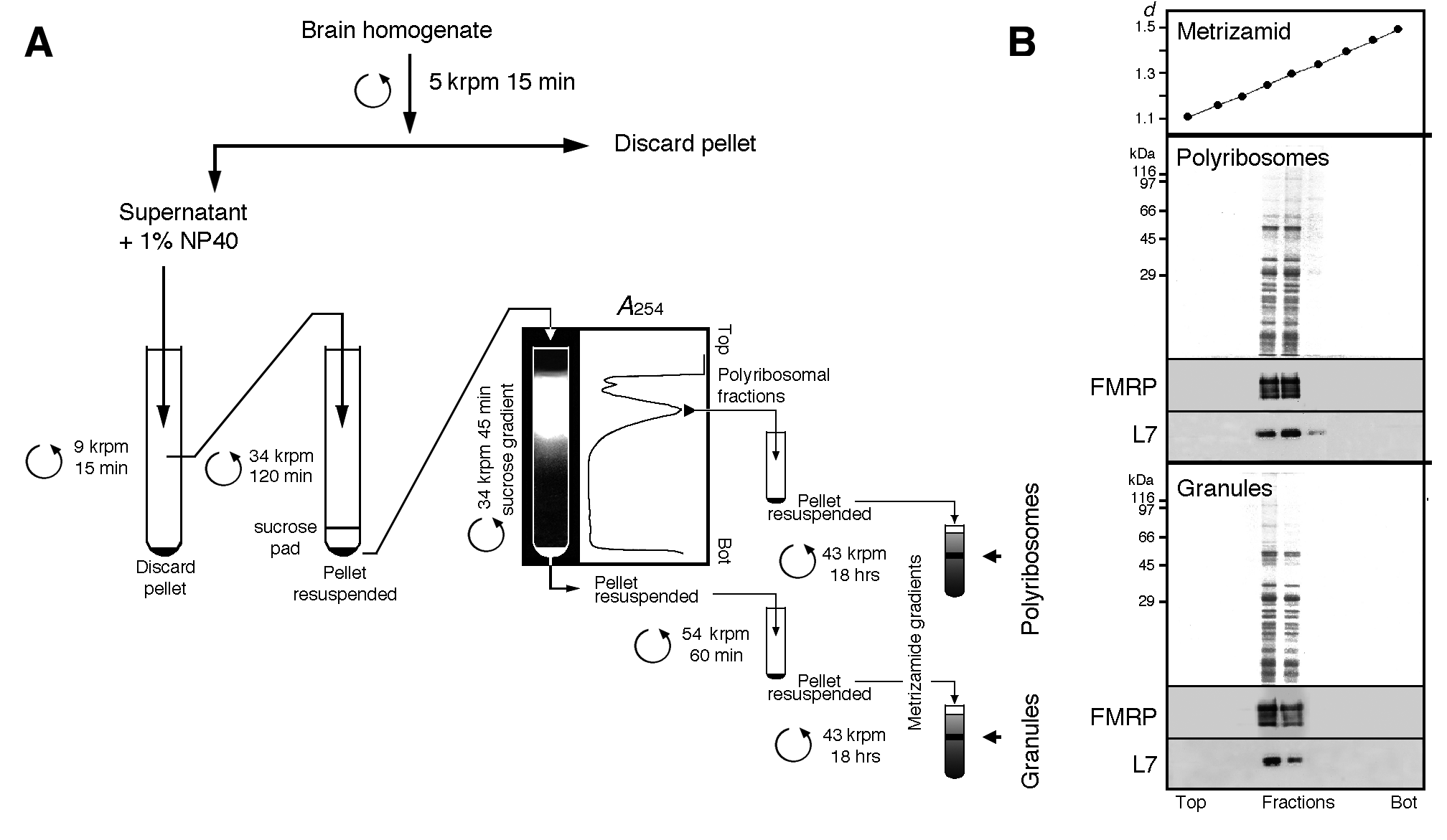

Supplement: S3 Fig — A) Schematic diagram of the steps used for the purification of polyribosomes and granules. B) Isopycnic centrifugation on Metrizamide gradients reveals that granules and polyribosomes have identical density properties. (TIF) [file pgen.1006192.s003.tif]

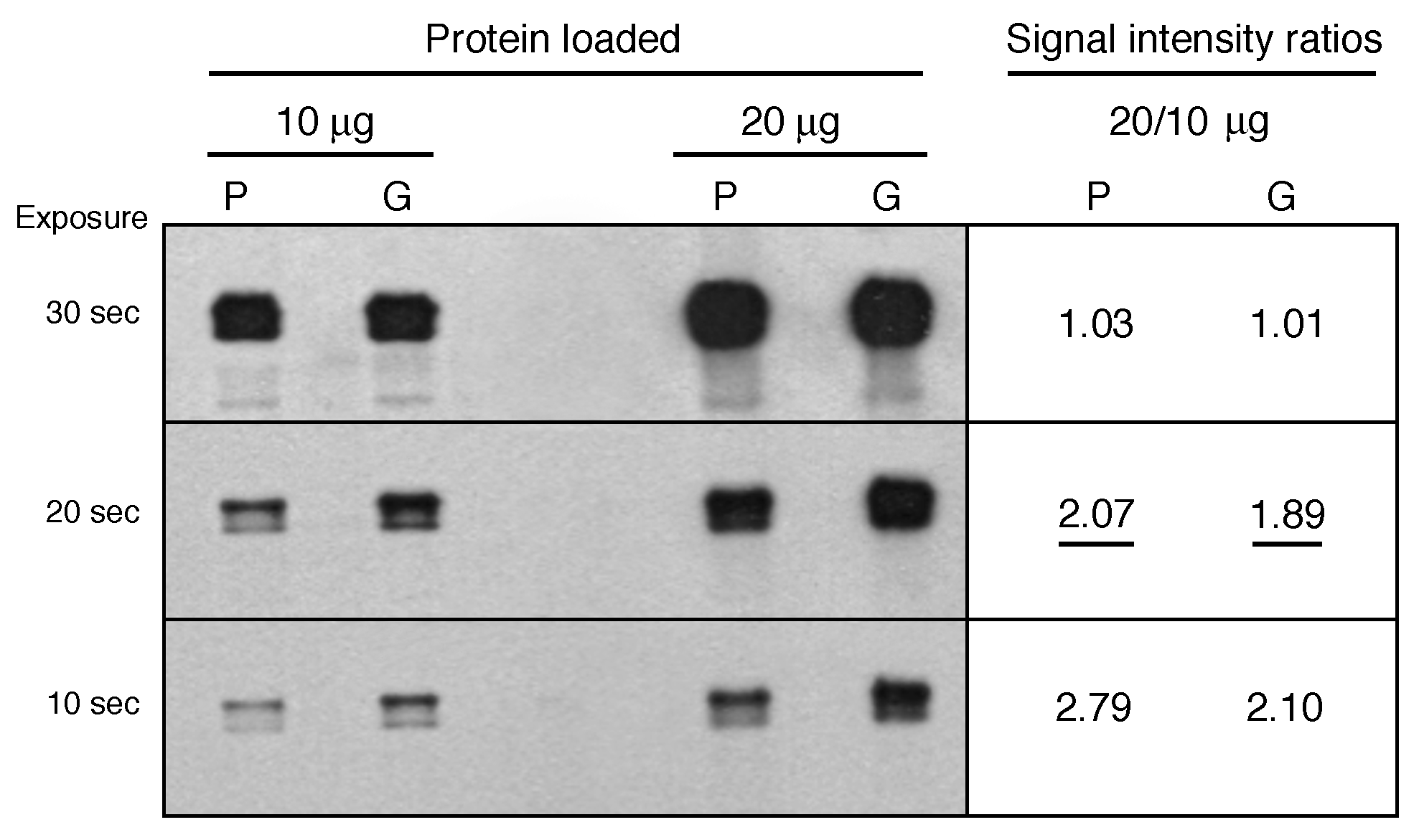

Supplement: S4 Fig — Ten and 20 μg of proteins from purified polyribosomes (P) and granules (G) were analysed in parallel by immunoblotting with IgY#C10, and the membrane exposed for different times to X ray films. Densitometric analyses enable to calculate that the ratio of 2 fold is observed with 10 μg loading and with a 20 sec exposure (underlined in the right panel). Quantitative analyses shown in Fig 6B in the main text, was performed under these conditions. (TIF) [file pgen.1006192.s004.tif]

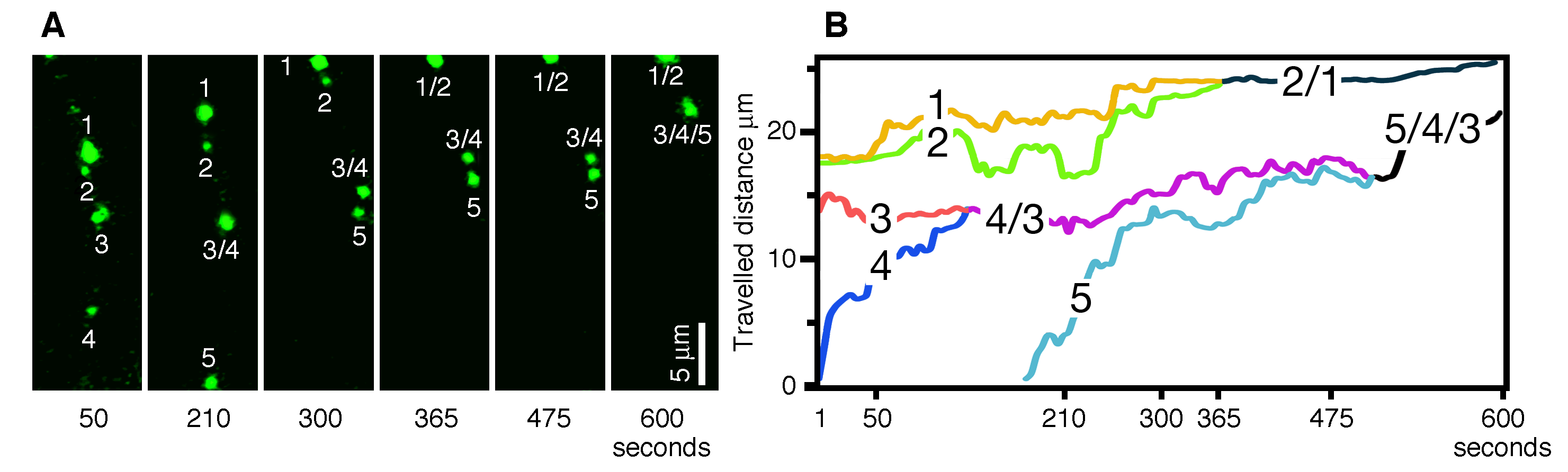

Supplement: S5 Fig — A) Presented here is the second boxed area in Fig 8A in the main text (Top left) showing the movements of 5 independent granules. B) Individual trajectories versus time of each of these granules. (TIF) [file pgen.1006192.s005.tif]
