# Supplementary material for: Tracking the Fragile X Mental Retardation Protein in a Highly Ordered Neuronal RiboNucleoParticles Population: A Link between Stalled Polyribosomes and RNA Granules
Source: PLoS Genet. 2016 Jul 27;12(7):e1006192. doi: 10.1371/journal.pgen.1006192 (PMC4963131; doi:10.1371/journal.pgen.1006192)
Supplement: S1 Table — For each protein, individual spectrum counts normalized to the total spectrum counts are presented and ordered from the highest (red) to the lowest (green) values in granules preparations. A value of 0 indicates that the protein was not detected. Official protein symbol and name, UniProt accession number and molecular weight (MW) are provided. (PDF) [file pgen.1006192.s006.pdf]

Entry name	Protein name	Alternate names	UniProt Accession	Gene name	MW (KDa)	Normalized spectrum counts		
						Granules	Polyribosomes	Ratio G/P
**CAPR1**	Caprin-1	**Caprin1** Gpiap,Gpiap1,Gpip137,M11s1,Rng105	Q60865	**Caprin1**	78	48	37	1,30
**RL4**	60S ribosomal protein L4	**Rpl4**	Q9D8E6	**Rpl4**	47	45	77	0,58
**G3BP2**	Ras GTPase-activating protein-binding protein 2	**G3bp2**	P97379	**G3bp2**	54	41	24	1,71
**HSP7C**	Heat shock cognate 71 kDa protein	**Hspa8** Hsc70,Hsc73	P63017	**Hspa8**	71	41	23	1,78
**MYH10**	Myosin-10	**Myh10**	Q61879	**Myh10**	229	41	0	only in G
**Q5FWB6**	60S acidic ribosomal protein P0	**Rplp0** mCG_17387	Q5FWB6	**Rplp0**	34	39	47	0,83
**PABP1**	Polyadenylate-binding protein 1	**Pabpc1** Pabp1	P29341	**Pabpc1**	71	38	40	0,95
**ACTG**	Actin, cytoplasmic 2	**Actg1** Actg	P63260	**Actg1**	42	35	3	11,67
**RS3**	40S ribosomal protein S3	**Rps3**	P62908	**Rps3**	27	32	31	1,03
**RSSA**	40S ribosomal protein SA	**Rpsa** Lamr1,P40-8	P14206	**Rpsa**	33	32	27	1,19
**GBLP**	Guanine nucleotide-binding protein ...	**Gnb2l1** Gnb2-rs1	P68040	**Gnb2l1**	35	29	26	1,12
**MYO5A**	Myosin-5a	**Myo5a**	B8JK04	**Myo5a**	215	29	7	4,14
**TBB2A**	Tubulin beta-2A chain	**Tubb2a** Tubb2	Q7TMM9	**Tubb2a**	50	29	10	2,90
**RS4X**	40S ribosomal protein S4, X isoform	**Rps4x** Rps4	P62702	**Rps4x**	30	25	27	0,93
**TBB5**	Tubulin beta-5 chain	**Tubb5**	P99024	**Tubb5**	50	25	0	only in G
**YBOX1**	Nuclease-sensitive element-binding protein 1	**Ybx1** Msy-1,Msy1,Nsep1,Yb1	P62960	**Ybx1**	36	25	21	1,19
**RL7A**	60S ribosomal protein L7a	**Rpl7a** Surf-3,Surf3	P12970	**Rpl7a**	30	22	31	0,71
**RL3**	60S ribosomal protein L3	**Rpl3**	P27659	**Rpl3**	46	20	43	0,47
**Q80Y51**	ELAV (Embryonic lethal, abnormal vision, Drosophila)-like 2 (Hu antigen B)	**Elavl2**	Q80Y51	**Elavl2**	41	19	18	1,06
**RL12**	60S ribosomal protein L12	**Rpl12**	P35979	**Rpl12**	18	19	17	1,12
**RL6**	60S ribosomal protein L6	**Rpl6**	P47911	**Rpl6**	34	19	43	0,44
**Q9CXQ0**	ELAV (Embryonic lethal, abnormal vision, Drosophila)-like 4 (Hu antigen D)	**Elavl4**	Q9CXQ0	**Elavl4**	41	17	13	1,31
**RL5**	60S ribosomal protein L5	**Rpl5**	P47962	**Rpl5**	34	17	19	0,89
**RENT1**	Regulator of nonsense transcripts 1	**Upf1** Rent1	Q9EPU0	**Upf1**	124	17	29	0,59
**GRP78**	78 kDa glucose-regulated protein	**Hspa5** Grp78	P20029	**Hspa5**	72	16	0	only in G
**RL10**	60S ribosomal protein L10	**Rpl10** Qm	Q6ZWV3	**Rpl10**	25	16	22	0,73
**RL23**	60S ribosomal protein L23	**Rpl23**	P62830	**Rpl23**	15	16	11	1,45
**RL30**	60S ribosomal protein L30	**Rpl30**	P62889	**Rpl30**	13	16	16	1,00
**RL38**	60S ribosomal protein L38	**Rpl38**	Q9JJI8	**Rpl38**	8	16	8	2,00
**RS27**	40S ribosomal protein S27	**Rps27**	Q6ZWU9	**Rps27**	9	16	10	1,60
**HNRPU**	Heterogeneous nuclear ribonucleoprotein U	**Hnrnpu** Hnrpu	Q8VEK3	**Hnrnpu**	88	15	25	0,60
**AINX**	Alpha-internexin	**Ina**	P46660	**Ina**	56	15	0	only in G
**RL14**	60S ribosomal protein L14	**Rpl14**	Q9CR57	**Rpl14**	24	15	27	0,56
**RS21**	40S ribosomal protein S21	**Rps21**	Q9CQR2	**Rps21**	9	15	6	2,50
**STAU2**	Double-stranded RNA-binding protein Staufen homolog 2	**Stau2**	Q8CJ67	**Stau2**	63	15	14	1,07
**Q6P5B5**	Fragile X mental retardation, autosomal homolog 2	**Fxr2** Fxr2h	Q6P5B5	**Fxr2**	74	13	10	1,30
**RL23A**	60S ribosomal protein L23a	**Rpl23a**	P62751	**Rpl23a**	18	13	12	1,08
**Q6ZWZ4**	60S ribosomal protein L36	**Rpl36** mCG_20352	Q6ZWZ4	**Rpl36**	12	13	14	0,93
**RL7**	60S ribosomal protein L7	**Rpl7**	P14148	**Rpl7**	31	13	27	0,48
**RS14**	40S ribosomal protein S14	**Rps14**	P62264	**Rps14**	16	13	11	1,18
**RS15**	40S ribosomal protein S15	**Rps15** Rig	P62843	**Rps15**	17	13	12	1,08
**RS18**	40S ribosomal protein S18	**Rps18**	P62270	**Rps18**	18	13	13	1,00
**RS3A**	40S ribosomal protein S3a	**Rps3a** Rps3a1	P97351	**Rps3a**	30	13	18	0,72
**TBA1A**	Tubulin alpha-1A chain	**Tuba1a** Tuba1	P68369	**Tuba1a**	50	13	4	3,25
**ELAV3**	ELAV (Embryonic lethal, abnormal vision, Drosophila)-like 3 (Hu antigen C)	**Elavl3** Huc	Q60900	**Elavl3**	40	12	13	0,92
**Q8VHM5**	Heterogeneous nuclear ribonucleoprotein R	**Hnrnpr** Hnrpr	Q8VHM5	**Hnrnpr**	71	12	14	0,86

**ILF3**	Interleukin enhancer-binding factor 3	**Ilf3**	Q9Z1X4	**Ilf3**	96	12	16	0,75
**Q99LF8**	Polyadenylate-binding protein, cytoplasmic 4	**Pabpc4**	Q99LF8	**Pabpc4**	72	12	12	1,00
**RL17**	60S ribosomal protein L17	**Rpl17**	Q9CPR4	**Rpl17**	21	12	20	0,60
**RLA1**	60S acidic ribosomal protein P1	**Rplp1**	P47955	**Rplp1**	11	12	8	1,50
**Q642L7**	40S ribosomal protein S27A	**Rps27a** mCG_13441,mCG_15222	Q642L7	**Rps27a**	18	12	6	2,00
**RS6**	40S ribosomal protein S6	**Rps6**	P62754	**Rps6**	29	12	21	0,57
**RS8**	40S ribosomal protein S8	**Rps8**	P62242	**Rps8**	24	12	34	0,35
**Q69ZJ3**	S phase cyclin A-associated protein in the ER	**Scaper** mKIAA1454,Zfp291	Q69ZJ3	**Scaper**	157	12	21	0,57
**HNRPQ**	Heterogeneous nuclear ribonucleoprotein Q	**Syncrip** Hnrpq,Nsap1,Nsap1l	Q7TMK9	**Syncrip**	70	12	15	0,80
**DHX30**	Putative ATP-dependent RNA helicase DHX30	**Dhx30** Helg	Q99PU8	**Dhx30**	137	10	22	0,45
**Q547R0**	Fragile X mental retardation protein FMRP	**Fmr1**	Q547R0	**Fmr1**	66	10	8	1,25
**FXR1**	Fragile X mental retardation syndrome-related protein 1	**Fxr1** Fxr1h	Q61584	**Fxr1**	76	10	4	2,50
**RL13**	60S ribosomal protein L13	**Rpl13**	P47963	**Rpl13**	24	10	16	0,63
**RL27A**	60S ribosomal protein L27a	**Rpl27a**	Q9CQ16	**Rpl27a**	17	10	14	0,71
**RL32**	60S ribosomal protein L32	**Rpl32**	P62911	**Rpl32**	16	10	7	1,43
**RLA2**	60S acidic ribosomal protein P2	**Rplp2**	P99027	**Rplp2**	12	10	8	1,25
**RS13**	40S ribosomal protein S13	**Rps13**	P62301	**Rps13**	17	10	14	0,71
**RS16**	40S ribosomal protein S16	**Rps16**	P14131	**Rps16**	16	10	21	0,48
**RS9**	40S ribosomal protein S9	**Rps9**	Q6ZWN5	**Rps9**	23	10	11	0,91
**VIME**	Vimentin	**Vim**	P20152	**Vim**	54	10	0	only in G
**G3BP1**	Ras GTPase-activating protein-binding protein 1	**G3bp1** G3bp	P97855	**G3bp1**	52	9	2	4,50
**Q3UXI9**	Interleukin enhancer-binding factor 2	**Ilf2**	Q3UXI9	**Ilf2**	43	9	10	0,90
**Q5XJF6**	Ribosomal protein L1	**Rpl10a** mCG_123122,mCG_18533	Q5XJF6	**Rpl10a**	25	9	12	0,75
**RL18**	60S ribosomal protein L18	**Rpl18**	P35980	**Rpl18**	22	9	19	0,47
**RL21**	60S ribosomal protein L21	**Rpl21**	O09167	**Rpl21**	19	9	12	0,75
**RL8**	60S ribosomal protein L8	**Rpl8**	P62918	**Rpl8**	28	9	9	1,00
**RL9**	60S ribosomal protein L9	**Rpl9**	P51410	**Rpl9**	22	9	14	0,64
**RS10**	40S ribosomal protein S10	**Rps10**	P63325	**Rps10**	19	9	7	1,29
**RS11**	40S ribosomal protein S11	**Rps11**	P62281	**Rps11**	18	9	11	0,82
**RS2**	40S ribosomal protein S2	**Rps2** Llrep3,Rps4	P25444	**Rps2**	31	9	18	0,50
**RS20**	40S ribosomal protein S20	**Rps20**	P60867	**Rps20**	13	9	11	0,82
**RS24**	40S ribosomal protein S24	**Rps24**	P62849	**Rps24**	15	9	16	0,56
**RS28**	40S ribosomal protein S28	**Rps28**	P62858	**Rps28**	8	9	2	4,50
**RS7**	40S ribosomal protein S7	**Rps7**	P62082	**Rps7**	22	9	15	0,60
**Q9DBE7**	Double-stranded RNA-binding protein Staufen homolog 1	**Stau1** mCG_14603	Q9DBE7	**Stau1**	54	9	10	0,90
**RS30**	40S ribosomal protein S30	**Fau**	P62862	**Rps30**	7	7	4	1,75
**PRKRA**	Interferon-inducible double stranded RNA-dependent protein kinase activator A	**Prkra** Rax	Q9WTX2	**Prkra**	34	7	8	0,88
**RN214**	RING finger protein 214	**Rnf214**	Q8BFU3	**Rnf214**	74	7	3	2,33
**RL13A**	60S ribosomal protein L13a	**Rpl13a** P198,Tstap198-7	P19253	**Rpl13a**	23	7	14	0,50
**RS19**	40S ribosomal protein S19	**Rps19**	Q9CZX8	**Rps19**	16	7	10	0,70
**RS23**	40S ribosomal protein S23	**Rps23**	P62267	**Rps23**	16	7	11	0,64
**RS25**	40S ribosomal protein S25	**Rps25**	P62852	**Rps25**	14	7	10	0,70
**Q91V55**	40S ribosomal protein S5	**Rps5** mCG_22552	Q91V55	**Rps5**	23	7	12	0,58
**EIF3B**	Eukaryotic translation initiation factor 3 subunit B	**Eif3b** Eif3s9	Q8JZQ9	**Eif3b**	91	6	4	1,50
**NFL**	Neurofilament light polypeptide	**Nefl** Nf68,Nfl	P08551	**Nefl**	62	6	0	only in G
**PURA**	Transcriptional activator protein Pur-alpha	**Pura**	P42669	**Pura**	35	6	11	0,55
**RL11**	60S ribosomal protein L11	**Rpl11**	Q9CXW4	**Rpl11**	20	6	9	0,67
**RL22**	60S ribosomal protein L22	**Rpl22**	P67984	**Rpl22**	15	6	6	1,00

**RL24**	60S ribosomal protein L24	**Rpl24**	Q8BP67	**Rpl24**	18	6	17	0,35
**RL26**	60S ribosomal protein L26	**Rpl26**	P61255	**Rpl26**	17	6	5	1,20
**RL31**	60S ribosomal protein L31	**Rpl31**	P62900	**Rpl31**	14	6	11	0,55
**RL35**	60S ribosomal protein L35	**Rpl35**	Q6ZWV7	**Rpl35**	15	6	5	1,20
**Q6ZWZ6**	40S ribosomal protein S12	**Rps12** Rps12-ps3,mCG_132913,mCG_6749	Q6ZWZ6	**Rps12**	15	6	5	1,20
**RS15A**	40S ribosomal protein S15a	**Rps15a**	P62245	**Rps15a**	15	6	10	0,60
**RS17**	40S ribosomal protein S17	**Rps17**	P63276	**Rps17**	16	6	12	0,50
**PAIRB**	Plasminogen activator inhibitor 1 RNA-binding protein	**Serbp1** Pairbp1	Q9CY58	**Serbp1**	45	6	7	0,86
**ACTN1**	Alpha-actinin-1	**Actn1**	Q7TPR4	**Actn1**	103	4	0	only in G
**DREB**	Drebrin	**Dbn1** Drba	Q9QXS6	**Dbn1**	77	4	0	only in G
**HNRPC**	Heterogeneous nuclear ribonucleoproteins C1/C2	**Hnrnpc** Hnrpc	Q9Z204	**Hnrnpc**	34	4	8	0,50
**NPM**	Nucleophosmin	**Npm1**	Q61937	**Npm1**	33	4	9	0,44
**RL15**	60S ribosomal protein L15	**Rpl15**	Q9CZM2	**Rpl15**	24	4	5	0,80
**RL27**	60S ribosomal protein L27	**Rpl27**	P61358	**Rpl27**	16	4	8	0,50
**SRPK1**	Serine/threonine-protein kinase SRPK1	**Srpk1**	O70551	**Srpk1**	73	4	6	0,67
**UBP10**	Ubiquitin carboxyl-terminal hydrolase 10	**Usp10** Kiaa0190,Ode-1,Uchrp	P52479	**Usp10**	87	4	8	0,50
**DYST**	Dystonin	**Dst** Bpag1,Macf2	Q91ZU6	**Dst**	834	3	0	only in G
**MA7D1**	MAP7 domain-containing protein 1	**Map7d1** Kiaa1187,Mtap7d1	A2AJI0	**Map7d1**	93	3	4	0,75
**ML12B**	Myosin regulatory light chain 12B	**Myl12b** Mrlc2,Mylc2b	Q3THE2	**Myl12b**	20	3	0	only in G
**MYL6**	Myosin light polypeptide 6	**Myl6** Myln	Q60605	**Myl6**	17	3	0	only in G
**NFM**	Neurofilament medium polypeptide	**Nefm** Nef3,Nfm	P08553	**Nefm**	96	3	0	only in G
**PRP19**	Pre-mRNA-processing factor 19	**Prpf19** Prp19,Snev	Q99KP6	**Prpf19**	55	3	2	1,50
**PURB**	Transcriptional activator protein Pur-beta	**Purb**	O35295	**Purb**	34	3	11	0,27
**RL19**	60S ribosomal protein L19	**Rpl19**	P84099	**Rpl19**	23	3	0	only in G
**RL35A**	60S ribosomal protein L35a	**Rpl35a**	O55142	**Rpl35a**	13	3	2	1,50
**RS26**	40S ribosomal protein S26	**Rps26**	P62855	**Rps26**	13	3	12	0,25
**LA**	Lupus La protein homolog	**Ssb** Ss-b	P32067	**Ssb**	48	3	3	1,00
**UBP2L**	Ubiquitin-associated protein 2-like	**Ubap2l**	Q80X50	**Ubap2l**	117	3	0	only in G
**1433Z**	14-3-3 protein zeta/delta	**Ywhaz**	P63101	**Ywhaz**	28	3	0	only in G
**EIF3C**	Eukaryotic translation initiation factor 3 subunit C	**Eif3c** Eif3s8	Q8R1B4	**Eif3c**	106	1	2	0,50
**HNRPM**	Heterogeneous nuclear ribonucleoprotein M	**Hnrnpm** Hnrpm	Q9D0E1	**Hnrnpm**	78	1	2	0,50
**RL18A**	60S ribosomal protein L18a	**Rpl18a**	P62717	**Rpl18a**	21	1	8	0,13
**RL34**	60S ribosomal protein L34	**Rpl34**	Q9D1R9	**Rpl34**	13	1	0	only in G
**RS29**	40S ribosomal protein S29	**Rps29**	P62274	**Rps29**	7	1	2	0,50
**AGAP2**	Arf-GAP with GTPase, ANK repeat and PH domain-containing protein 2	**Agap2** Centg1,Kiaa0167	Q3UHD9	**Agap2**	125	0	3	only in P
**ARHG2**	Rho guanine nucleotide exchange factor 2	**Arhgef2** Kiaa0651,Lbcl1,Lfc	Q60875	**Arhgef2**	112	0	3	only in P
**ATX2L**	Ataxin-2-like protein	**Atxn2l** A2lp	Q7TQH0	**Atxn2l**	111	0	2	only in P
**SPF27**	Pre-mRNA-splicing factor SPF27	**Bcas2** Dam1	Q9D287	**Bcas2**	26	0	2	only in P
**CDC5L**	Cell division cycle 5-like protein	**Cdc5l** Kiaa0432	Q6A068	**Cdc5l**	92	0	7	only in P
**CKAP4**	Cytoskeleton-associated protein 4	**Ckap4**	Q8BMK4	**Ckap4**	64	0	2	only in P
**YBOX3**	Y-box-binding protein 3	**Ybx3** Csda,Msy4	Q9JKB3	**Csda**	39	0	11	only in P
**DHX29**	ATP-dependent RNA helicase DHX29	**Dhx29**	Q6PGC1	**Dhx29**	154	0	2	only in P
**DHX36**	ATP-dependent RNA helicase DHX36	**Dhx36** Ddx36,Kiaa1488,Mlel1	Q8VHK9	**Dhx36**	114	0	8	only in P
**DHX9**	ATP-dependent RNA helicase A	**Dhx9** Ddx9	O70133	**Dhx9**	149	0	8	only in P
**EIF3A**	Eukaryotic translation initiation factor 3 subunit A	**Eif3a** Csma,Eif3,Eif3s10	P23116	**Eif3a**	162	0	13	only in P
**EIF3L**	Eukaryotic translation initiation factor 3 subunit L	**Eif3l** Eif3eip,Eif3s6ip,Paf67	Q8QZY1	**Eif3l**	67	0	2	only in P
**F120A**	Constitutive coactivator of PPAR-gamma-like protein 1	**FAM120A** Kiaa0183,Ossa	Q6A0A9	**FAM120A**	122	0	8	only in P
**F120C**	Constitutive coactivator of PPAR-gamma-like protein 2	**Fam120c** ORF34	Q8C3F2	**Fam120c**	120	0	6	only in P

**Q8K2R0**	Farp1 protein	**Farp1**	Q8K2R0	**Farp1**	95	0	2	only in P
**HNRH1**	Heterogeneous nuclear ribonucleoprotein H	**Hnrnph1** Hnrph,Hnrph1	O35737	**Hnrnph1**	49	0	4	only in P
**HNRPK**	Heterogeneous nuclear ribonucleoprotein K	**Hnrnpk** Hnrpk	P61979	**Hnrnpk**	51	0	4	only in P
**LARP1**	La-related protein 1	**Larp1** Kiaa0731,Larp	Q6ZQ58	**Larp1**	121	0	2	only in P
**LARP4**	La-related protein 4	**Larp4**	Q8BWW4	**Larp4**	80	0	2	only in P
**LAR4B**	La-related protein 4B	**Larp4b** D13Wsu64e,Kiaa0217,Larp5	Q6A0A2	**Larp4b**	82	0	9	only in P
**LAS1L**	Ribosomal biogenesis protein LAS1L	**Las1l**	A2BE28	**Las1l**	89	0	3	only in P
**MATR3**	Matrin-3	**Matr3**	Q8K310	**Matr3**	95	0	4	only in P
**NCBP1**	Nuclear cap-binding protein subunit 1	**Ncbp1** Cbp80	Q3UYV9	**Ncbp1**	92	0	2	only in P
**NUCL**	Nucleolin	**Ncl** Nuc	P09405	**Ncl**	77	0	4	only in P
**NOL9**	Polynucleotide 5'-hydroxyl-kinase nucleolar protein 9	**Nol9**	Q3TZX8	**Nol9**	81	0	2	only in P
**NUFP2**	Nuclear fragile X mental retardation-interacting protein 2	**Nufip2** Kiaa1321	Q5F2E7	**Nufip2**	76	0	6	only in P
**PRC2A**	Large proline-rich protein BAT2	**Prrc2a** Bat2	Q7TSC1	**Prrc2a**	229	0	5	only in P
**PRC2C**	BAT2 domain-containing protein 1	**Prrc2c** Bat2d,Bat2d1,Bat2l2,Kiaa1096	Q3TLH4	**Prrc2c**	309	0	6	only in P
**PURG**	Purine-rich element-binding protein gamma	**Purg**	Q8R4E6	**Purg**	40	0	6	only in P
**RBMX**	Heterogeneous nuclear ribonucleoprotein G	**Rbmx** Hnrnpg,Hnrpg,Rbmxp1,Rbmxrt	Q9R0Y0	**Rbmx**	31	0	4	only in P
**RL28**	60S ribosomal protein L28	**Rpl28**	P41105	**Rpl28**	16	0	1	only in P
**RL37A**	60S ribosomal protein L37a	**Rpl37a**	P61514	**Rpl37a**	10	0	2	only in P
**RRBP1**	Ribosome-binding protein 1	**Rrbp1**	Q99PL5	**Rrbp1**	173	0	3	only in P
**SUGP2**	Putative splicing factor, arginine/serine-rich 14	**Sugp2** Sfrs14,Srsf14	Q8CH09	**Sfrs14**	118	0	2	only in P
**SPAS2**	Spermatogenesis-associated serine-rich protein 2	**Spats2** Scr59	Q8K1N4	**Spats2**	59	0	2	only in P
**SPS2L**	SPATS2-like protein	**Spats2l**	Q91WJ7	**Spats2l**	62	0	3	only in P
**STRBP**	Spermatid perinuclear RNA-binding protein	**Strbp** Spnr	Q91WM1	**Strbp**	74	0	4	only in P
**TEX10**	Testis-expressed sequence 10 protein	**Tex10**	Q3URQ0	**Tex10**	105	0	2	only in P
**THOC4**	THO complex subunit 4	**Alyref** Aly,Ref1,Refbp1,THOC4	O08583	**Thoc4**	27	0	2	only in P
**TTC3**	E3 ubiquitin-protein ligase, tetratricopeptide repeat domain 3	**Ttc3** Kiaa4119	O88196	**Ttc3**	224	0	2	only in P
**WDR18**	WD repeat-containing protein 18	**Wdr18**	Q4VBE8	**Wdr18**	47	0	2	only in P
**A2A7S7**	Tyrosyl-tRNA synthetase	**Yars** mCG_141662	A2A7S7	**Yars**	63	0	6	only in P
**ZFR**	Zinc finger RNA-binding protein	**Zfr**	O88532	**Zfr**	117	0	4	only in P
		**Total proteins detected**				**128**	**155**	
		**Total normalized spectrum counts**				**1579**	**1789**	
		**Ribosomal proteins normalized spectrum counts**				**765**	**1011**	
		**% normalized spectrum counts corresponding to ribosomal proteins**				**48,4%**	**56,5%**	
