# Supplementary material for: Tracking the Fragile X Mental Retardation Protein in a Highly Ordered Neuronal RiboNucleoParticles Population: A Link between Stalled Polyribosomes and RNA Granules
Source: PLoS Genet. 2016 Jul 27;12(7):e1006192. doi: 10.1371/journal.pgen.1006192 (PMC4963131; doi:10.1371/journal.pgen.1006192)
Supplement: S2 Table — Analyses were performed using the DAVID bioinformatics resources with the granule list as input. GOTERM categories: biological processes, cellular components and molecular functions with a significant Benjamini’s adjusted pvalue (p<0.05) are presented. (PDF) [file pgen.1006192.s007.pdf]

GOTERM category	Term	PValue	Fold Enrichment	Bonferroni	Benjamini	FDR
**Biological Process**	**GO:0006414~translational elongation**	4,13E-131	81,28	2,67E-128	2,67E-128	6,16E-128
	**GO:0006412~translation**	2,87E-90	25,85	1,85E-87	9,26E-88	4,28E-87
	**GO:0042274~ribosomal small subunit biogenesis**	9,90E-18	105,11	6,39E-15	2,13E-15	1,48E-14
	**GO:0042254~ribosome biogenesis**	1,11E-17	18,01	7,15E-15	1,79E-15	1,65E-14
	**GO:0006396~RNA processing**	1,07E-14	6,13	6,89E-12	1,38E-12	1,59E-11
	**GO:0022613~ribonucleoprotein complex biogenesis**	1,31E-14	12,20	8,46E-12	1,41E-12	1,95E-11
	**GO:0006364~rRNA processing**	3,74E-14	18,85	2,42E-11	3,45E-12	5,58E-11
	**GO:0016072~rRNA metabolic process**	6,92E-14	18,07	4,47E-11	5,59E-12	1,03E-10
	**GO:0042273~ribosomal large subunit biogenesis**	3,33E-13	92,50	2,15E-10	2,39E-11	4,98E-10
	**GO:0034470~ncRNA processing**	5,95E-11	9,89	3,85E-08	3,85E-09	8,88E-08
	**GO:0034660~ncRNA metabolic process**	1,09E-09	8,04	7,06E-07	6,42E-08	1,63E-06
	**GO:0043488~regulation of mRNA stability**	3,33E-05	26,28	2,13E-02	1,79E-03	4,97E-02
	**GO:0045103~intermediate filament-based process**	3,33E-05	26,28	2,13E-02	1,79E-03	4,97E-02
	**GO:0006413~translational initiation**	3,95E-05	15,42	2,52E-02	1,96E-03	5,89E-02
	**GO:0043487~regulation of RNA stability**	4,78E-05	24,09	3,04E-02	2,20E-03	7,12E-02
	**GO:0042255~ribosome assembly**	7,06E-05	46,25	4,46E-02	3,03E-03	1,05E-01
	**GO:0016071~mRNA metabolic process**	7,81E-05	4,06	4,92E-02	3,15E-03	1,16E-01
	**GO:0030705~cytoskeleton-dependent intracellular transport**	8,01E-05	13,34	5,04E-02	3,04E-03	1,19E-01
	**GO:0010608~posttranscriptional regulation of gene expression**	8,30E-05	5,48	5,22E-02	2,98E-03	1,24E-01
	**GO:0042257~ribosomal subunit assembly**	2,17E-04	115,62	1,31E-01	7,37E-03	3,24E-01
	**GO:0043489~RNA stabilization**	2,59E-04	30,83	1,54E-01	8,34E-03	3,86E-01
	**GO:0048255~mRNA stabilization**	2,59E-04	30,83	1,54E-01	8,34E-03	3,86E-01
	**GO:0045104~intermediate filament cytoskeleton organization**	6,30E-04	23,12	3,34E-01	1,92E-02	9,35E-01
	**GO:0033119~negative regulation of RNA splicing**	7,17E-04	69,37	3,71E-01	2,08E-02	1,06E+00
	**GO:0006397~mRNA processing**	1,76E-03	3,60	6,79E-01	4,83E-02	2,59E+00
**Cellular Component**	**GO:0022626~cytosolic ribosome**	5,46E-117	82,85	9,17E-115	9,17E-115	6,64E-114
	**GO:0005840~ribosome**	4,72E-103	36,66	7,93E-101	3,97E-101	5,75E-100
	**GO:0033279~ribosomal subunit**	1,40E-102	54,09	2,36E-100	7,86E-101	1,71E-99
	**GO:0030529~ribonucleoprotein complex**	1,40E-100	18,61	2,36E-98	5,89E-99	1,71E-97
	**GO:0044445~cytosolic part**	4,75E-94	44,85	7,98E-92	1,60E-92	5,78E-91
	**GO:0022627~cytosolic small ribosomal subunit**	3,97E-57	85,21	6,67E-55	1,11E-55	4,84E-54
	**GO:0005829~cytosol**	1,11E-55	6,81	1,86E-53	2,66E-54	1,35E-52
	**GO:0022625~cytosolic large ribosomal subunit**	4,80E-53	84,09	8,06E-51	1,01E-51	5,84E-50
	**GO:0043232~intracellular non-membrane-bounded organelle**	4,43E-52	4,19	7,43E-50	8,26E-51	5,39E-49
	**GO:0043228~non-membrane-bounded organelle**	4,43E-52	4,19	7,43E-50	8,26E-51	5,39E-49
	**GO:0015935~small ribosomal subunit**	7,90E-50	55,79	1,33E-47	1,33E-48	9,62E-47
	**GO:0015934~large ribosomal subunit**	1,13E-48	52,46	1,89E-46	1,72E-47	1,37E-45
	**GO:0005730~nucleolus**	4,45E-09	3,97	7,47E-07	6,23E-08	5,42E-06
	**GO:0031981~nuclear lumen**	1,74E-05	2,28	2,93E-03	2,25E-04	2,12E-02
	**GO:0060053~neurofilament cytoskeleton**	9,00E-05	42,61	1,50E-02	1,08E-03	1,10E-01
	**GO:0070013~intracellular organelle lumen**	1,36E-04	1,98	2,26E-02	1,52E-03	1,65E-01
	**GO:0043233~organelle lumen**	2,08E-04	1,93	3,44E-02	2,19E-03	2,53E-01
	**GO:0031974~membrane-enclosed lumen**	2,99E-04	1,89	4,90E-02	2,95E-03	3,64E-01
	**GO:0005844~polysome**	5,80E-04	23,67	9,28E-02	5,40E-03	7,04E-01
	**GO:0005883~neurofilament**	1,26E-03	53,26	1,91E-01	1,11E-02	1,52E+00
	**GO:0005681~spliceosome**	1,45E-03	5,65	2,16E-01	1,21E-02	1,75E+00
**Molecular Function**	**GO:0003735~structural constituent of ribosome**	1,58E-100	43,00	2,36E-98	2,36E-98	1,89E-97
	**GO:0005198~structural molecule activity**	1,50E-73	13,21	2,23E-71	1,11E-71	1,78E-70
	**GO:0003723~RNA binding**	4,13E-66	11,37	6,15E-64	2,05E-64	4,93E-63
	**GO:0003729~mRNA binding**	8,66E-19	26,97	1,29E-16	3,23E-17	1,03E-15
	**GO:0019843~rRNA binding**	3,90E-12	36,10	5,82E-10	1,16E-10	4,66E-09
	**GO:0003725~double-stranded RNA binding**	2,54E-04	15,86	3,71E-02	6,28E-03	3,02E-01
	**GO:0005200~structural constituent of cytoskeleton**	6,72E-04	8,49	9,54E-02	1,42E-02	7,99E-01
	**GO:0008143~poly(A) RNA binding**	2,40E-03	39,26	3,01E-01	4,38E-02	2,83E+00
	**GO:0045182~translation regulator activity**	2,53E-03	14,44	3,15E-01	4,11E-02	2,98E+00
	**GO:0070717~poly-purine tract binding**	3,07E-03	34,90	3,67E-01	4,48E-02	3,60E+00
