# Supplementary material for: Tracking the Fragile X Mental Retardation Protein in a Highly Ordered Neuronal RiboNucleoParticles Population: A Link between Stalled Polyribosomes and RNA Granules
Source: PLoS Genet. 2016 Jul 27;12(7):e1006192. doi: 10.1371/journal.pgen.1006192 (PMC4963131; doi:10.1371/journal.pgen.1006192)
Supplement: S3 Table — Analysis was performed using the DAVID bioinformatics resources with the granule list as input. GOTERM categories: biological processes, cellular components and molecular functions with a significant Benjamini’s adjusted pvalue (p<0.05) are presented. (PDF) [file pgen.1006192.s008.pdf]

| GOTERM category | Term | PValue | Fold Enrichment |
| --- | --- | --- | --- |
| Biological process | GO:0006414~translational elongation | 9,73E-128 | 75,47 |
| | GO:0006412~translation | 3,44E-96 | 25,62 |
| | GO:0006396~RNA processing | 2,09E-22 | 7,46 |
| | GO:0042274~ribosomal small subunit biogenesis | 1,98E-17 | 97,60 |
| | GO:0042254~ribosome biogenesis | 4,40E-17 | 16,72 |
| | GO:0022613~ribonucleoprotein complex biogenesis | 2,09E-16 | 12,53 |
| | GO:0006364~rRNA processing | 1,08E-13 | 17,51 |
| | GO:0016072~rRNA metabolic process | 1,99E-13 | 16,78 |
| | GO:0042273~ribosomal large subunit biogenesis | 5,69E-13 | 85,89 |
| | GO:0016071~mRNA metabolic process | 2,27E-11 | 6,38 |
| | GO:0034470~ncRNA processing | 1,76E-10 | 9,19 |
| | GO:0034660~ncRNA metabolic process | 3,49E-10 | 7,94 |
| | GO:0006397~mRNA processing | 8,52E-10 | 6,35 |
| | GO:0008380~RNA splicing | 9,67E-10 | 6,80 |
| | GO:0006413~translational initiation | 1,65E-07 | 19,09 |
| | GO:0043488~regulation of mRNA stability | 1,44E-06 | 29,28 |
| | GO:0000377~RNA splicing, via transesterification reactions with bulged adenosine as nucleophile | 1,49E-06 | 7,72 |
| | GO:0000375~RNA splicing, via transesterification reactions | 1,49E-06 | 7,72 |
| | GO:0000398~nuclear mRNA splicing, via spliceosome | 1,49E-06 | 7,72 |
| | GO:0043487~regulation of RNA stability | 2,30E-06 | 26,84 |
| | GO:0010608~posttranscriptional regulation of gene expression | 4,01E-06 | 6,11 |
| | GO:0043489~RNA stabilization | 8,76E-06 | 35,79 |
| | GO:0048255~mRNA stabilization | 8,76E-06 | 35,79 |
| | GO:0042255~ribosome assembly | 8,81E-05 | 42,95 |
| | GO:0042257~ribosomal subunit assembly | 2,53E-04 | 107,37 |
| | GO:0006403~RNA localization | 3,24E-04 | 7,52 |
| | GO:0022618~ribonucleoprotein complex assembly | 4,35E-04 | 9,34 |
| | GO:0033119~negative regulation of RNA splicing | 8,32E-04 | 64,42 |
| | GO:0050657~nucleic acid transport | 2,03E-03 | 6,64 |
| | GO:0051236~establishment of RNA localization | 2,03E-03 | 6,64 |
| | GO:0050658~RNA transport | 2,03E-03 | 6,64 |
| | GO:0051168~nuclear export | 2,27E-03 | 8,95 |
| Cellular component | GO:0030529~ribonucleoprotein complex | 1,12E-115 | 18,89 |
| | GO:0022626~cytosolic ribosome | 3,69E-110 | 73,01 |
| | GO:0005840~ribosome | 5,80E-102 | 33,72 |
| | GO:0033279~ribosomal subunit | 4,58E-96 | 47,69 |
| | GO:0044445~cytosolic part | 1,14E-83 | 38,28 |
| | GO:0022627~cytosolic small ribosomal subunit | 2,02E-55 | 76,31 |
| | GO:0022625~cytosolic large ribosomal subunit | 6,39E-49 | 72,80 |
| | GO:0005829~cytosol | 1,81E-48 | 6,02 |
| | GO:0043228~non-membrane-bounded organelle | 3,63E-48 | 3,89 |
| | GO:0043232~intracellular non-membrane-bounded organelle | 3,63E-48 | 3,89 |
| | GO:0015935~small ribosomal subunit | 4,56E-48 | 49,97 |
| | GO:0015934~large ribosomal subunit | 7,10E-45 | 45,56 |
| | GO:0005730~nucleolus | 1,69E-14 | 4,78 |
| | GO:0031981~nuclear lumen | 2,81E-10 | 2,83 |
| | GO:0005681~spliceosome | 1,18E-08 | 9,39 |
| | GO:0070013~intracellular organelle lumen | 4,18E-08 | 2,36 |
| | GO:0043233~organelle lumen | 8,15E-08 | 2,31 |
| | GO:0031974~membrane-enclosed lumen | 1,44E-07 | 2,26 |
| | GO:0030530~heterogeneous nuclear ribonucleoprotein complex | 2,40E-05 | 28,06 |
| | GO:0005844~polysome | 3,06E-05 | 26,50 |
| | GO:0005852~eukaryotic translation initiation factor 3 complex | 4,57E-04 | 25,44 |
| | GO:0042788~polysomal ribosome | 6,36E-04 | 71,54 |
| Molecular function | GO:0003735~structural constituent of ribosome | 1,65E-95 | 38,09 |
| | GO:0003723~RNA binding | 1,25E-85 | 12,27 |
| | GO:0005198~structural molecule activity | 1,04E-61 | 11,12 |
| | GO:0003729~mRNA binding | 6,51E-18 | 23,89 |
| | GO:0019843~rRNA binding | 1,19E-11 | 31,98 |
| | GO:0003725~double-stranded RNA binding | 1,18E-06 | 19,67 |
| | GO:0003727~single-stranded RNA binding | 1,82E-04 | 17,17 |
| | GO:0008143~poly(A) RNA binding | 3,05E-03 | 34,78 |
| | GO:0045182~translation regulator activity | 3,58E-03 | 12,79 |
| | GO:0070717~poly-purine tract binding | 3,90E-03 | 30,91 |
| | GO:0008026~ATP-dependent helicase activity | 4,01E-03 | 5,68 |
| | GO:0070035~purine NTP-dependent helicase activity | 4,01E-03 | 5,68 |
| | GO:0003743~translation initiation factor activity | 4,10E-03 | 7,60 |
| | GO:0003730~mRNA 3'-UTR binding | 5,88E-03 | 25,29 |

| Bonferroni | Benjamini | FDR |
|---|---|---|
| 5,32E-125 | 5,32E-125 | 1,42E-124 |
| 1,88E-93 | 9,42E-94 | 5,02E-93 |
| 1,14E-19 | 3,81E-20 | 3,05E-19 |
| 1,08E-14 | 2,71E-15 | 2,89E-14 |
| 2,41E-14 | 4,82E-15 | 6,42E-14 |
| 1,21E-13 | 2,02E-14 | 3,22E-13 |
| 5,91E-11 | 8,44E-12 | 1,58E-10 |
| 1,09E-10 | 1,36E-11 | 2,90E-10 |
| 3,11E-10 | 3,46E-11 | 8,30E-10 |
| 1,24E-08 | 1,24E-09 | 3,32E-08 |
| 9,62E-08 | 8,75E-09 | 2,57E-07 |
| 1,91E-07 | 1,59E-08 | 5,09E-07 |
| 4,66E-07 | 3,58E-08 | 1,24E-06 |
| 5,29E-07 | 3,78E-08 | 1,41E-06 |
| 9,01E-05 | 6,01E-06 | 2,40E-04 |
| 7,89E-04 | 4,93E-05 | 2,10E-03 |
| 8,13E-04 | 4,78E-05 | 2,17E-03 |
| 8,13E-04 | 4,78E-05 | 2,17E-03 |
| 8,13E-04 | 4,78E-05 | 2,17E-03 |
| 1,25E-03 | 6,97E-05 | 3,35E-03 |
| 2,19E-03 | 1,16E-04 | 5,85E-03 |
| 4,78E-03 | 2,40E-04 | 1,28E-02 |
| 4,78E-03 | 2,40E-04 | 1,28E-02 |
| 4,71E-02 | 2,29E-03 | 1,28E-01 |
| 1,29E-01 | 6,26E-03 | 3,68E-01 |
| 1,62E-01 | 7,67E-03 | 4,71E-01 |
| 2,12E-01 | 9,88E-03 | 6,33E-01 |
| 3,66E-01 | 1,80E-02 | 1,21E+00 |
| 6,72E-01 | 4,19E-02 | 2,93E+00 |
| 6,72E-01 | 4,19E-02 | 2,93E+00 |
| 6,72E-01 | 4,19E-02 | 2,93E+00 |
| 7,12E-01 | 4,50E-02 | 3,26E+00 |
| 1,55E-113 | 1,55E-113 | 1,32E-112 |
| 5,13E-108 | 2,57E-108 | 4,35E-107 |
| 8,07E-100 | 2,69E-100 | 6,84E-99 |
| 6,37E-94 | 1,59E-94 | 5,40E-93 |
| 1,59E-81 | 3,18E-82 | 1,35E-80 |
| 2,81E-53 | 4,69E-54 | 2,38E-52 |
| 8,88E-47 | 1,27E-47 | 7,52E-46 |
| 2,51E-46 | 3,14E-47 | 2,13E-45 |
| 5,05E-46 | 5,61E-47 | 4,28E-45 |
| 5,05E-46 | 5,61E-47 | 4,28E-45 |
| 6,33E-46 | 6,33E-47 | 5,37E-45 |
| 9,87E-43 | 8,97E-44 | 8,37E-42 |
| 2,35E-12 | 1,96E-13 | 1,99E-11 |
| 3,91E-08 | 3,01E-09 | 3,31E-07 |
| 1,64E-06 | 1,17E-07 | 1,39E-05 |
| 5,81E-06 | 3,88E-07 | 4,93E-05 |
| 1,13E-05 | 7,08E-07 | 9,60E-05 |
| 2,00E-05 | 1,17E-06 | 1,69E-04 |
| 3,33E-03 | 1,85E-04 | 2,83E-02 |
| 4,25E-03 | 2,24E-04 | 3,61E-02 |
| 6,16E-02 | 3,17E-03 | 5,38E-01 |
| 8,46E-02 | 4,20E-03 | 7,47E-01 |
| 2,69E-93 | 2,69E-93 | 2,00E-92 |
| 2,03E-83 | 1,02E-83 | 1,51E-82 |
| 1,70E-59 | 5,66E-60 | 1,26E-58 |
| 1,06E-15 | 2,65E-16 | 7,88E-15 |
| 1,94E-09 | 3,88E-10 | 1,44E-08 |
| 1,92E-04 | 3,21E-05 | 1,43E-03 |
| 2,93E-02 | 4,24E-03 | 2,21E-01 |
| 3,93E-01 | 6,04E-02 | 3,64E+00 |
| 4,43E-01 | 6,29E-02 | 4,25E+00 |
| 4,71E-01 | 6,17E-02 | 4,62E+00 |
| 4,80E-01 | 5,78E-02 | 4,75E+00 |
| 4,80E-01 | 5,78E-02 | 4,75E+00 |
| 4,88E-01 | 5,42E-02 | 4,85E+00 |
| 6,17E-01 | 7,12E-02 | 6,89E+00 |
