# Supplementary material for: Tracking the Fragile X Mental Retardation Protein in a Highly Ordered Neuronal RiboNucleoParticles Population: A Link between Stalled Polyribosomes and RNA Granules
Source: PLoS Genet. 2016 Jul 27;12(7):e1006192. doi: 10.1371/journal.pgen.1006192 (PMC4963131; doi:10.1371/journal.pgen.1006192)
Supplement: S4 Table — For each transcript, probe and gene ID are provided. Enrichment is calculated as the logarithm (base 2) of the ratio in average probe intensity (log2FC) in granules as compared to polyribosomes preparation. Transcripts corresponding to probes displaying an enrichment equals or above 1 (FC>1; log2(FC)>0) with a significant adjusted pvalue (pval<0.002) are presented. In case of redundant probes targeting a single mRNA, data are provided for the probe providing the highest level of variation. The colour code indicates the highest (red) to the lowest (green) folds of change detected. Putative FMRP mRNA targets identified in Darnell et al. [33] are highlighted in green. (PDF) [file pgen.1006192.s009.pdf]

Probe ID	GeneSymbol	GeneName	log2FC	FC	P-value	Adj p-value	PrimaryAcces	RefSeqAcces	GenbankAcce	UniGeneID	EntrezGeneID	EnsemblID
A_55_P2459897	***A2m***	alpha-2-macroglobulin	1,05	2,07	2,22E-06	1,50E-04	NM_175628	NM_175628	NM_175628	Mm.30151	232345	ENSMUST00000032203
A_55_P2052056	***A3galt2***	alpha 1,3-galactosyltransferase 2 (isoglobotriaosylceramide s	0,85	1,80	2,13E-05	5,72E-04	NM_00100981	NM_00100981	NM_00100981	Mm.300900	215493	ENSMUST00000030585
A_51_P453043	***Aacs***	acetoacetyl-CoA synthetase	0,83	1,77	2,46E-06	1,59E-04	NM_030210	NM_030210	NM_030210	Mm.431573	78894	ENSMUST00000031445
A_55_P2015495	***Abat***	4-aminobutyrate aminotransferase	0,82	1,77	1,37E-05	4,41E-04	NM_172961	NM_172961	NM_172961	Mm.259315	268860	ENSMUST00000065987
A_52_P665675	***Abca1***	ATP-binding cassette, sub-family A (ABC1), member 1	1,15	2,22	1,53E-05	4,70E-04	NM_013454	NM_013454	NM_013454	Mm.277376	11303	ENSMUST00000030010
A_52_P519653	***Abca5***	ATP-binding cassette, sub-family A (ABC1), member 5	1,28	2,44	8,35E-07	9,30E-05	NM_147219	NM_147219	NM_147219	Mm.41942	217265	ENSMUST00000043961
A_51_P363556	***Abi2***	abl-interactor 2	0,95	1,94	2,05E-05	5,59E-04	NM_198127	NM_198127	NM_198127	Mm.212066	329165	ENSMUST00000052332
A_51_P155196	***Abtb2***	ankyrin repeat and BTB (POZ) domain containing 2	1,00	2,00	1,48E-05	4,62E-04	NM_178890	NM_178890	NM_178890	Mm.35850	99382	ENSMUST00000076212
A_55_P2082914	***Acly***	ATP citrate lyase	1,30	2,46	1,28E-06	1,12E-04	NM_134037	NM_134037	NM_134037	Mm.282039	104112	ENSMUST00000107385
A_55_P2096043	***Acot11***	acyl-CoA thioesterase 11	0,82	1,76	3,81E-05	8,05E-04	NM_025590	NM_025590	NM_025590	Mm.222956	329910	ENSMUST00000102762
A_51_P414305	***Acot12***	acyl-CoA thioesterase 12	1,06	2,08	1,23E-05	4,11E-04	NM_028790	NM_028790	NM_028790	Mm.275963	74156	ENSMUST00000022120
A_55_P2186615	***Acp1***	acid phosphatase 1, soluble	0,95	1,93	1,07E-05	3,81E-04	NM_00111023	NM_00111023	NM_00111023	Mm.359831	11431	ENSMUST00000062740
A_52_P496956	***Acsbg1***	acyl-CoA synthetase bubblegum family member 1	0,90	1,87	1,16E-05	3,98E-04	NM_053178	NM_053178	NM_053178	Mm.20592	94180	ENSMUST00000034822
A_55_P1985984	***Actg1***	actin, gamma, cytoplasmic 1	0,90	1,87	2,86E-05	6,77E-04	NM_009609	NM_009609	NM_009609	Mm.426706	11465	ENSMUST00000131103
A_51_P347862	***Actn1***	actinin, alpha 1	1,84	3,59	1,39E-07	4,10E-05	NM_134156	NM_134156	NM_134156	Mm.253564	109711	ENSMUST00000167327
A_51_P108266	***Actn2***	actinin alpha 2	2,42	5,35	7,82E-09	9,66E-06	NM_033268	NM_033268	NM_033268	Mm.37638	11472	ENSMUST00000168193
A_51_P394788	***Actn4***	actinin alpha 4	1,80	3,49	4,32E-08	2,20E-05	NM_021895	NM_021895	NM_021895	Mm.81144	60595	ENSMUST00000068045
A_51_P378825	***Actr8***	ARP8 actin-related protein 8	0,54	1,45	1,39E-04	1,88E-03	NM_027493	NM_027493	NM_027493	Mm.215110	56249	ENSMUST00000016115
A_55_P2025760	***Adam9***	a disintegrin and metallopeptidase domain 9 (meltrin gamma)	0,92	1,89	6,46E-05	1,14E-03	NM_00127099	NM_00127099	NM_00127099	Mm.28908	11502	ENSMUST00000084032
A_52_P489295	***Adamts1***	disintegrin-like metallopeptidase with thrombospondin type 1 r	1,01	2,01	3,65E-06	2,03E-04	NM_009621	NM_009621	NM_009621	Mm.1421	11504	ENSMUST00000023610
A_52_P233305	***Adamts12***	a disintegrin-like and metallopeptidase (reprolysin type) with tl	0,64	1,56	5,72E-05	1,05E-03	NM_175501	NM_175501	NM_175501	Mm.482727	239337	ENSMUST00000061318
A_51_P311038	***Adamts15***	a disintegrin-like and metallopeptidase (reprolysin type) with tl	0,88	1,84	8,38E-05	1,34E-03	NM_00102413	NM_00102413	NM_00102413	Mm.65867	235130	ENSMUST00000065112
A_66_P124114	***Adamts17***	disintegrin-like metallopeptidase with thrombospondin type 1 r	1,13	2,20	1,16E-06	1,08E-04	NM_00103387	NM_00103387	NM_00103387	Mm.445763	233332	ENSMUST00000148538
A_55_P1955778	***Adamts4***	disintegrin-likemetallopeptidase with thrombospondin type 1 n	1,72	3,30	5,71E-07	7,87E-05	NM_172845	NM_172845	NM_172845	Mm.23156	240913	ENSMUST00000006570
A_52_P183181	***Adar***	adenosine deaminase, RNA-specific	0,70	1,63	1,05E-04	1,56E-03	NM_00103858	NM_00103858	NM_00103858	Mm.316628	56417	ENSMUST00000107405
A_55_P2106645	***Adcy5***	adenylate cyclase 5	0,72	1,64	2,73E-05	6,61E-04	NM_00101276	NM_00101276	NM_00101276	Mm.41137	224129	ENSMUST00000114913
A_55_P1990336	***Add1***	adducin 1 (alpha)	0,75	1,69	1,47E-04	1,95E-03	NM_00110244	NM_00110244	NM_00110244	Mm.289106	11518	ENSMUST00000114338
A_55_P1988623	***Add3***	adducin 3 (gamma)	1,26	2,39	4,56E-07	7,05E-05	NM_00116409	NM_00116409	NM_00116409	Mm.426080	27360	ENSMUST00000111741
A_66_P105307	***Adnp***	activity-dependent neuroprotective protein	1,42	2,67	1,68E-05	4,96E-04	NM_009628	NM_009628	NM_009628	Mm.201322	11538	ENSMUST00000057793
A_52_P179583	***Adnp2***	ADNP homeobox 2	0,71	1,64	1,71E-05	5,01E-04	NM_175028	NM_175028	NM_175028	Mm.26594	240442	ENSMUST00000066743
A_55_P2046938	***Adpgk***	ADP-dependent glucokinase	0,83	1,78	1,33E-04	1,83E-03	NM_028121	NM_028121	NM_028121	Mm.22456	72141	ENSMUST00000026266
A_52_P424778	***Adra1a***	adrenergic receptor, alpha 1a	1,17	2,25	1,82E-05	5,20E-04	NM_013461	NM_013461	NM_013461	Mm.57064	11549	ENSMUST00000054661
A_55_P2019954	***Adra1d***	adrenergic receptor, alpha 1d	1,08	2,12	9,53E-07	9,82E-05	NM_013460	NM_013460	NM_013460	Mm.389380	11550	0
A_51_P269687	***Adsl***	adenylosuccinate lyase	0,79	1,73	3,07E-05	7,09E-04	NM_009634	NM_009634	NM_009634	Mm.38151	11564	ENSMUST00000166711
A_52_P206613	***Adss***	adenylosuccinate synthetase, non muscle	0,80	1,74	4,49E-05	8,99E-04	NM_007422	NM_007422	NM_007422	Mm.338021	11566	ENSMUST00000159184
A_55_P2030030	***Adssl1***	adenylosuccinate synthetase like 1	0,49	1,40	1,27E-04	1,78E-03	NM_007421	NM_007421	NM_007421	Mm.3440	11565	ENSMUST00000180015
A_51_P183283	***Aff2***	AF4/FMR2 family, member 2	1,77	3,41	3,27E-07	6,07E-05	NM_008032	NM_008032	NM_008032	Mm.466576	14266	ENSMUST00000033532
A_51_P486121	***Aff3***	AF4/FMR2 family, member 3	1,16	2,24	3,11E-06	1,84E-04	NM_010678	NM_010678	NM_010678	Mm.336679	16764	ENSMUST00000039827
A_52_P551743	***Aff4***	AF4/FMR2 family, member 4	0,99	1,99	7,64E-05	1,26E-03	NM_033565	NM_033565	NM_033565	Mm.395281	93736	ENSMUST00000060945
A_51_P119544	***Aga***	aspartylglucosaminidase	1,14	2,20	9,54E-06	3,58E-04	NM_00100584	NM_00100584	NM_00100584	Mm.334535	11593	ENSMUST00000033920
A_55_P2427685	***Agl***	amylo-1,6-glucosidase, 4-alpha-glucanotransferase	0,88	1,83	5,90E-05	1,07E-03	NM_00108132	NM_00108132	NM_00108132	Mm.237099	77559	ENSMUST00000162792
A_55_P2009127	***Agrn***	agrin	1,25	2,38	9,04E-05	1,42E-03	NM_021604	NM_021604	NM_021604	Mm.273098	11603	ENSMUST00000071248
A_51_P118763	***Ahctf1***	AT hook containing transcription factor 1	0,76	1,70	5,14E-05	9,82E-04	NM_026375	NM_026375	NM_026375	Mm.128165	226747	ENSMUST00000151734
A_51_P206268	***Ahcyl1***	S-adenosylhomocysteine hydrolase-like 1	0,78	1,72	3,11E-05	7,11E-04	NM_145542	NM_145542	NM_145542	Mm.220328	229709	ENSMUST00000029490
A_52_P162509	***Ahcyl2***	S-adenosylhomocysteine hydrolase-like 2	1,01	2,01	4,89E-05	9,52E-04	NM_021414	NM_021414	NM_021414	Mm.210899	74340	ENSMUST00000141304
A_52_P263095	***Ahnak***	AHNAK nucleoprotein (desmoyokin)	0,92	1,89	1,48E-04	1,96E-03	NM_009643	NM_009643	NM_009643	Mm.203866	66395	ENSMUST00000092956
A_65_P06603	***Ahr***	aryl-hydrocarbon receptor	0,80	1,74	1,08E-04	1,59E-03	NM_013464	NM_013464	NM_013464	Mm.341377	11622	ENSMUST00000116436
A_52_P517289	***Akap1***	A kinase (PRKA) anchor protein 1	0,99	1,99	6,98E-05	1,19E-03	NM_00104254	NM_00104254	NM_00104254	Mm.2969	11640	ENSMUST00000153787
A_52_P244964	***Akap11***	A kinase (PRKA) anchor protein 11	1,54	2,91	1,63E-07	4,35E-05	NM_00116450	NM_00116450	NM_00116450	Mm.89413	219181	ENSMUST00000022593
A_55_P2099810	***Akap12***	A kinase (PRKA) anchor protein (gravin) 12	1,28	2,42	1,68E-07	4,37E-05	NM_031185	NM_031185	NM_031185	Mm.27481	83397	ENSMUST00000045730
A_55_P2444515	***Akap2***	A kinase (PRKA) anchor protein 2	1,14	2,20	7,45E-06	3,10E-04	NM_00103553	NM_00103553	NM_00103553	Mm.467752	11641	ENSMUST00000098066
A_55_P2350032	***Akap6***	A kinase (PRKA) anchor protein 6	1,71	3,26	1,36E-08	1,33E-05	NM_198111	NM_198111	NM_198111	Mm.310822	238161	ENSMUST00000095737
A_66_P106716	***Akap8***	A kinase (PRKA) anchor protein 8	0,77	1,71	5,99E-05	1,08E-03	NM_019774	NM_019774	NM_019774	Mm.328945	56399	ENSMUST00000002699
A_55_P2060369	***Akap9***	A kinase (PRKA) anchor protein (yotiao) 9	1,07	2,09	2,24E-05	5,90E-04	NM_194462	NM_194462	NM_194462	Mm.46044	100986	ENSMUST00000044492
A_55_P2083649	***Alas1***	aminolevulinic acid synthase 1	1,35	2,55	1,54E-07	4,22E-05	NM_020559	NM_020559	NM_020559	Mm.290578	11655	ENSMUST00000134053
A_51_P359272	***Alcam***	activated leukocyte cell adhesion molecule	1,14	2,20	1,17E-05	4,00E-04	NM_009655	NM_009655	NM_009655	Mm.288282	11658	ENSMUST00000164888
A_65_P19286	***Alg10b***	asparagine-linked glycosylation 10B (alpha-1,2-glucosyltransf	0,92	1,90	2,35E-05	6,03E-04	NM_00103344	NM_00103344	NM_00103344	Mm.17853	380959	ENSMUST00000100309
A_52_P93902	***Alg5***	asparagine-linked glycosylation 5 (dolichyl-phosphate beta-glu	0,81	1,75	1,73E-05	5,04E-04	NM_025442	NM_025442	NM_025442	Mm.144218	66248	ENSMUST00000044567
A_55_P2007663	***Alkbh8***	alkB, alkylation repair homolog 8 (E. coli)	0,75	1,69	3,09E-05	7,10E-04	NM_026303	NM_026303	NM_026303	Mm.116968	67667	ENSMUST00000165105
A_55_P2454521	***Alms1***	Alstrom syndrome 1	0,69	1,62	3,51E-05	7,66E-04	NM_145223	NM_145223	NM_145223	Mm.246967	236266	ENSMUST00000072018
A_51_P247249	***Alox5***	arachidonate 5-lipoxygenase	0,86	1,81	4,39E-06	2,24E-04	NM_009662	NM_009662	NM_009662	Mm.41072	11689	ENSMUST00000169625
A_52_P336585	***Als2***	amyotrophic lateral sclerosis 2 (juvenile)	0,90	1,86	6,34E-05	1,12E-03	NM_028717	NM_028717	NM_028717	Mm.272078	74018	ENSMUST00000159166
A_55_P2168431	***Amer1***	APC membrane recruitment 1	1,26	2,39	2,52E-06	1,61E-04	NM_175179	NM_175179	NM_175179	Mm.182867	72345	ENSMUST00000084535
A_55_P2046509	***Amot***	angiomotin	2,35	5,09	1,81E-08	1,50E-05	NM_153319	NM_153319	NM_153319	Mm.100068	27494	ENSMUST00000112835
A_55_P2244112	***Amotl1***	angiomotin-like 1	0,87	1,83	8,16E-05	1,32E-03	NM_00108139	NM_00108139	NM_00108139	Mm.159552	75723	ENSMUST00000160770
A_51_P348804	***Amotl2***	angiomotin-like 2	1,67	3,19	9,64E-07	9,86E-05	NM_019764	NM_019764	NM_019764	Mm.21145	56332	ENSMUST00000142011
A_51_P141390	***Anapc1***	anaphase promoting complex subunit 1	1,03	2,04	1,75E-05	5,07E-04	NM_008569	NM_008569	NM_008569	Mm.277408	17222	ENSMUST00000014499
A_51_P112355	***Angel1***	angel homolog 1 (Drosophila)	0,46	1,38	1,16E-04	1,67E-03	NM_144524	NM_144524	NM_144524	Mm.63979	68737	ENSMUST00000021682
A_55_P2063785	***Ank1***	ankyrin 1, erythroid	0,49	1,41	1,41E-04	1,90E-03	NM_00111078	NM_00111078	NM_00111078	Mm.334444	11733	ENSMUST00000033947
A_55_P2131288	***Ank2***	ankyrin 2, brain	2,26	4,80	1,76E-06	1,32E-04	NM_178655	NM_178655	NM_178655	Mm.220242	109676	ENSMUST00000044443
A_65_P13713	***Ank3***	ankyrin 3, epithelial	1,81	3,50	4,86E-08	2,30E-05	NM_146005	NM_146005	NM_146005	Mm.235960	11735	ENSMUST00000092433
A_55_P2121563	***Ankfy1***	ankyrin repeat and FYVE domain containing 1	1,46	2,75	5,76E-07	7,91E-05	NM_009671	NM_009671	NM_009671	Mm.10313	11736	ENSMUST00000132889
A_55_P2034390	***Ankhd1***	ankyrin repeat and KH domain containing 1	1,03	2,04	6,82E-07	8,57E-05	NM_175375	NM_175375	NM_175375	Mm.482287	108857	ENSMUST00000006205
A_52_P504330	***Ankib1***	ankyrin repeat and IBR domain containing 1	1,30	2,47	1,32E-05	4,29E-04	NM_00100390	NM_00100390	NM_00100390	Mm.130148	70797	ENSMUST00000043551
A_55_P2391227	***Ankle2***	ankyrin repeat and LEM domain containing 2	0,93	1,91	2,98E-06	1,80E-04	NM_027922	NM_027922	NM_027922	Mm.288442	71782	ENSMUST00000086674
A_52_P24428	***Ankrd11***	ankyrin repeat domain 11	0,67	1,59	1,15E-04	1,66E-03	ENSMUST000	0	BC059876	Mm.31567	77087	ENSMUST00000098334
A_52_P277139	***Ankrd17***	ankyrin repeat domain 17	1,19	2,29	1,96E-07	4,65E-05	NM_030886	NM_030886	NM_030886	Mm.245522	81702	ENSMUST00000014421
A_55_P2134690	***Ankrd26***	ankyrin repeat domain 26	0,89	1,85	1,98E-06	1,41E-04	NM_00108111	NM_00108111	NM_00108111	Mm.276116	232339	ENSMUST00000112830
A_51_P207412	***Ankrd32***	ankyrin repeat domain 32	0,57	1,49	1,38E-04	1,87E-03	NM_134071	NM_134071	NM_134071	Mm.209730	105377	ENSMUST00000151524
A_55_P1971672	***Ankrd50***	ankyrin repeat domain 50	1,24	2,36	1,55E-05	4,74E-04	NM_00116788	NM_00116788	NM_00116788	Mm.290937	99696	ENSMUST00000120875
A_55_P2040455	***Ano10***	anoctamin 10	0,93	1,90	1,21E-06	1,10E-04	NM_133979	NM_133979	NM_133979	Mm.87676	102566	ENSMUST00000042546
A_52_P662711	***Anxa13***	annexin A13	1,24	2,35	7,00E-06	2,98E-04	NM_027211	NM_027211	NM_027211	Mm.237985	69787	ENSMUST00000068515
A_55_P2076792	***Anxa6***	annexin A6	0,84	1,79	4,71E-06	2,34E-04	NM_013472	NM_013472	NM_013472	Mm.265347	11749	ENSMUST00000108883
A_51_P486681	***Ap3b2***	adaptor-related protein complex 3, beta 2 subunit	1,20	2,30	1,86E-06	1,36E-04	NM_021492	NM_021492	NM_021492	Mm.322894	11775	ENSMUST00000082090
A_51_P344249	***Apbb1***	amyloid beta (A4) precursor protein-binding, family B, membe	0,74	1,67	2,08E-05	5,63E-04	NM_009685	NM_009685	NM_009685	Mm.38469	11785	ENSMUST00000081165
A_55_P2010891	***Apc***	adenomatosis polyposis coli	1,49	2,81	1,74E-05	5,05E-04	NM_007462	NM_007462	NM_007462	Mm.384171	11789	ENSMUST00000115781
A_55_P2075627	***Apc2***	adenomatosis polyposis coli 2	1,30	2,47	4,85E-05	9,48E-04	NM_011789	NM_011789	NM_011789	Mm.57247	23805	ENSMUST00000020349
A_51_P473576	***Apex1***	apurinic/apyrimidinic endonuclease 1	0,54	1,46	1,11E-04	1,62E-03	NM_009687	NM_009687	NM_009687	Mm.203	11792	ENSMUST00000049411
A_52_P142191	***Aph1b***	anterior pharynx defective 1b homolog (C. elegans)	1,06	2,09	9,35E-06	3,54E-04	NM_177583	NM_177583	NM_177583	Mm.41291	208117	ENSMUST00000168309
A_51_P277295	***Apmap***	adipocyte plasma membrane associated protein	0,49	1,40	1,31E-04	1,82E-03	NM_027977	NM_027977	NM_027977	Mm.26599	71881	ENSMUST00000046399
A_51_P488510	***Appl1***	adaptor protein, phosphotyrosine interaction, PH domain and	0,66	1,58	6,18E-05	1,10E-03	NM_145221	NM_145221	NM_145221	Mm.202322	72993	ENSMUST00000036570
A_51_P179907	***Appl2***	adaptor protein, phosphotyrosine interaction, PH domain and	1,15	2,22	3,56E-06	1,99E-04	NM_145220	NM_145220	NM_145220	Mm.282985	216190	ENSMUST00000020500
A_55_P2177953	***Aqp11***	aquaporin 11	0,80	1,74	1,56E-05	4,75E-04	NM_175105	NM_175105	NM_175105	Mm.29756	66333	ENSMUST00000084986
A_55_P2000369	***Aqp4***	aquaporin 4	0,96	1,95	9,00E-05	1,41E-03	NM_009700	NM_009700	NM_009700	Mm.250786	11829	ENSMUST00000079081
A_52_P157240	***Aqr***	aquarius	1,29	2,44	4,61E-06	2,31E-04	NM_009702	NM_009702	NM_009702	Mm.2545	11834	ENSMUST00000102543
A_52_P478629	***Arcn1***	archain 1	0,80	1,74	6,65E-05	1,16E-03	NM_145985	NM_145985	NM_145985	Mm.371682	213827	ENSMUST00000034607
A_52_P83557	***Arfgef2***	ADP-ribosylation factor guanine nucleotide-exchange factor 2	0,59	1,51	3,18E-05	7,23E-04	NM_00108549	NM_00108549	NM_00108549	Mm.297192	99371	ENSMUST00000099078
A_52_P527106	***Arhgap12***	Rho GTPase activating protein 12	0,68	1,61	5,10E-05	9,77E-04	NM_00103969	NM_00103969	NM_00103969	Mm.239675	75415	ENSMUST00000077128
A_55_P2400691	***Arhgap21***	Rho GTPase activating protein 21	1,66	3,16	1,34E-07	4,05E-05	NM_00112808	NM_00112808	NM_00112808	Mm.28507	71435	ENSMUST00000173194
A_55_P2039566	***Arhgap23***	Rho GTPase activating protein 23	1,56	2,94	4,09E-07	6,72E-05	NM_021493	NM_021493	NM_021493	Mm.394118	58996	ENSMUST00000107601
A_52_P559808	***Arhgef12***	Rho guanine nucleotide exchange factor (GEF) 12	0,91	1,88	1,21E-05	4,06E-04	NM_027144	NM_027144	NM_027144	Mm.275266	69632	ENSMUST00000072767
A_55_P1963863	***Arhgef17***	Rho guanine nucleotide exchange factor (GEF) 17	1,16	2,24	3,18E-07	6,03E-05	NM_00108111	NM_00108111	NM_00108111	Mm.29954	207212	ENSMUST00000107032
A_51_P394735	***Arhgef18***	rho/rac guanine nucleotide exchange factor (GEF) 18	0,98	1,97	9,14E-06	3,50E-04	NM_133962	NM_133962	NM_133962	Mm.170461	102098	ENSMUST00000156085
A_51_P419925	***Arhgef40***	Rho guanine nucleotide exchange factor (GEF) 40	1,07	2,10	1,12E-05	3,91E-04	NM_00114592	NM_00114592	NM_00114592	Mm.9239	268739	ENSMUST00000100639
A_51_P159565	***Arhgef9***	CDC42 guanine nucleotide exchange factor (GEF) 9	0,80	1,74	2,04E-05	5,58E-04	NM_00103332	NM_00103332	NM_00103332	Mm.44841	236915	ENSMUST00000128565
A_66_P116888	***Arid1a***	AT rich interactive domain 1A (SWI-like)	1,43	2,69	7,79E-05	1,28E-03	NM_00108081	NM_00108081	NM_00108081	Mm.22478	93760	ENSMUST00000105897
A_52_P289256	***Arid1b***	AT rich interactive domain 1B (SWI-like)	1,16	2,23	1,04E-06	1,02E-04	NM_00108535	NM_00108535	NM_00108535	Mm.133401	239985	ENSMUST00000115797
A_52_P432580	***Arid5b***	AT rich interactive domain 5B (MRF1-like)	0,56	1,47	4,83E-05	9,47E-04	BC082312	0	BC082312	Mm.490355	71371	0
A_51_P396752	***Arl2bp***	ADP-ribosylation factor-like 2 binding protein	0,78	1,72	6,19E-06	2,79E-04	NM_024191	NM_024191	NM_024191	Mm.393247	107566	ENSMUST00000034228

A_52_P302463	*Arl5a*	ADP-ribosylation factor-like 5A	0,95	1,94	1,06E-05	3,81E-04	NM_182994	NM_182994	NM_182994	Mm.314378	75423	ENSMUST00000036541
A_51_P301964	*Arl6ip1*	ADP-ribosylation factor-like 6 interacting protein 1	0,77	1,71	3,79E-05	8,02E-04	NM_019419	NM_019419	NM_019419	Mm.29924	54208	ENSMUST00000032888
A_55_P2120469	*Armc2*	armadillo repeat containing 2	0,61	1,53	5,81E-05	1,06E-03	NM_00103485	NM_00103485	NM_00103485	Mm.211320	213402	ENSMUST00000160262
A_52_P215418	*Armc9*	armadillo repeat containing 9	0,71	1,64	4,46E-05	8,96E-04	NM_027456	NM_027456	NM_027456	Mm.379264	78795	ENSMUST00000155168
A_51_P346641	*Armcx4*	armadillo repeat containing, X-linked 4	1,46	2,75	2,63E-05	6,47E-04	NM_0012025C	NM_0012025C	NM_0012025C	Mm.31961	100503043	0
A_51_P191586	*Arpc1a*	actin related protein 2/3 complex, subunit 1A	0,58	1,49	1,36E-04	1,85E-03	NM_019767	NM_019767	NM_019767	Mm.371610	56443	ENSMUST00000142276
A_55_P2057587	*Arx*	aristaless related homeobox	0,88	1,84	4,27E-05	8,69E-04	NM_007492	NM_007492	NM_007492	Mm.275547	11878	ENSMUST00000046565
A_55_P1964154	*Asah1*	N-acylsphingosine amidohydrolase 1	1,38	2,61	3,10E-06	1,84E-04	NM_019734	NM_019734	NM_019734	Mm.22547	11886	ENSMUST00000126561
A_55_P2071137	*Asap1*	ArfGAP with SH3 domain, ankyrin repeat and PH domain1	1,13	2,19	3,02E-06	1,81E-04	NM_010026	NM_010026	NM_010026	Mm.277236	13196	ENSMUST00000023008
A_55_P1957918	*Asap2*	ArfGAP with SH3 domain, ankyrin repeat and PH domain 2	0,67	1,59	7,52E-05	1,25E-03	NM_00113519	NM_00113519	NM_00113519	Mm.358946	211914	ENSMUST00000064595
A_51_P421094	*Ascc3*	activating signal cointegrator 1 complex subunit 3	1,40	2,63	1,98E-07	4,67E-05	NM_198007	NM_198007	NM_198007	Mm.222497	77987	ENSMUST00000035606
A_51_P223929	*Ash1l*	ash1 (absent, small, or homeotic)-like (Drosophila)	1,71	3,28	8,68E-07	9,54E-05	NM_138679	NM_138679	NM_138679	Mm.130752	192195	ENSMUST00000090933
A_51_P174645	*Asl*	argininosuccinate lyase	0,68	1,60	2,75E-05	6,63E-04	NM_133768	NM_133768	NM_133768	Mm.23869	109900	ENSMUST00000160129
A_55_P1988228	*Aspm*	asp (abnormal spindle)-like, microcephaly associated (Drosop	1,98	3,95	1,09E-08	1,18E-05	NM_009791	NM_009791	NM_009791	Mm.168523	12316	ENSMUST00000053364
A_51_P143951	*Astn1*	astrotactin 1	1,19	2,28	7,41E-07	8,79E-05	NM_007495	NM_007495	NM_007495	Mm.329586	11899	ENSMUST00000046110
A_55_P2398399	*Astn2*	astrotactin 2	0,60	1,51	2,83E-05	6,73E-04	NM_207109	NM_207109	NM_207109	Mm.445312	56079	ENSMUST00000068214
A_51_P140607	*Asun*	asunder, spermatogenesis regulator	0,75	1,68	9,52E-05	1,47E-03	NM_138757	NM_138757	NM_138757	Mm.271774	71177	ENSMUST00000032427
A_52_P262219	*Atcay*	ataxia, cerebellar, Cayman type homolog (human)	0,56	1,47	8,30E-05	1,33E-03	NM_178662	NM_178662	NM_178662	Mm.127681	16467	ENSMUST00000047408
A_52_P478729	*Atg2a*	autophagy related 2A	0,82	1,76	3,05E-05	7,08E-04	NM_194348	NM_194348	NM_194348	Mm.277384	329015	ENSMUST00000145600
A_55_P1987988	*Atg2b*	autophagy related 2B	0,83	1,78	2,59E-06	1,64E-04	NM_029654	NM_029654	NM_029654	Mm.114362	76559	ENSMUST00000041055
A_55_P2151762	*Atg7*	autophagy related 7	0,82	1,76	1,47E-04	1,95E-03	NM_00125371	NM_00125371	NM_00125371	Mm.275332	74244	ENSMUST00000169310
A_55_P2424459	*Atg9a*	autophagy related 9A	1,17	2,25	2,71E-06	1,68E-04	NM_00100391	NM_00100391	NM_00100391	Mm.358931	245860	ENSMUST00000040689
A_66_P134534	*Atl2*	atlastin GTPase 2	0,82	1,76	1,14E-04	1,65E-03	NM_019717	NM_019717	NM_019717	Mm.175403	56298	ENSMUST00000068282
A_52_P369581	*Atm*	ataxia telangiectasia mutated homolog (human)	1,24	2,36	9,50E-06	3,58E-04	NM_007499	NM_007499	NM_007499	Mm.5088	11920	ENSMUST00000118282
A_55_P1971076	*Atp11a*	ATPase, class VI, type 11A	0,96	1,94	2,30E-05	5,96E-04	NM_015804	NM_015804	NM_015804	Mm.257837	50770	ENSMUST00000091237
A_55_P2083839	*Atp13a1*	ATPase type 13A1	1,01	2,02	6,32E-07	8,24E-05	BC138721	0	BC138721	Mm.186066	170759	0
A_55_P2083841	*Atp13a1*	ATPase type 13A1	0,80	1,75	3,06E-05	7,08E-04	NM_133224	NM_133224	NM_133224	Mm.186066	170759	ENSMUST00000034326
A_52_P227267	*Atp1a2*	ATPase, Na+/K+ transporting, alpha 2 polypeptide	1,23	2,34	8,86E-05	1,40E-03	NM_178405	NM_178405	NM_178405	Mm.207432	98660	ENSMUST00000131751
A_51_P151484	*Atp1b1*	ATPase, Na+/K+ transporting, beta 1 polypeptide	1,03	2,05	6,06E-05	1,09E-03	NM_009721	NM_009721	NM_009721	Mm.4550	11931	ENSMUST00000027863
A_51_P520384	*Atp1b3*	ATPase, Na+/K+ transporting, beta 3 polypeptide	0,74	1,67	1,41E-04	1,90E-03	NM_007502	NM_007502	NM_007502	Mm.424	11933	ENSMUST00000034983
A_51_P244856	*Atp2a1*	ATPase, Ca++ transporting, cardiac muscle, fast twitch 1	1,86	3,63	9,03E-07	9,68E-05	NM_007504	NM_007504	NM_007504	Mm.35134	11937	ENSMUST00000032974
A_51_P451075	*Atp2a2*	ATPase, Ca++ transporting, cardiac muscle, slow twitch 2	1,67	3,19	3,59E-07	6,23E-05	NM_009722	NM_009722	NM_009722	Mm.227583	11938	ENSMUST00000177974
A_66_P138001	*Atp2b1*	ATPase, Ca++ transporting, plasma membrane 1	1,51	2,84	1,55E-05	4,74E-04	NM_026482	NM_026482	NM_026482	Mm.166944	67972	ENSMUST00000020107
A_51_P256384	*Atp2b2*	ATPase, Ca++ transporting, plasma membrane 2	0,93	1,90	6,09E-06	2,76E-04	NM_009723	NM_009723	NM_009723	Mm.321755	11941	ENSMUST00000101044
A_55_P2013178	*Atp2b3*	ATPase, Ca++ transporting, plasma membrane 3	0,93	1,90	4,23E-06	2,20E-04	AK032322	0	AK032322	Mm.210095	320707	ENSMUST00000033744
A_55_P1985265	*Atp2b4*	ATPase, Ca++ transporting, plasma membrane 4	1,22	2,33	1,85E-05	5,26E-04	NM_00116794	NM_00116794	NM_00116794	Mm.188617	381290	ENSMUST00000048953
A_55_P2051899	*Atp2c1*	ATPase, Ca++-sequestering	0,77	1,71	1,18E-04	1,69E-03	NM_175025	NM_175025	NM_175025	Mm.326247	235574	ENSMUST00000038118
A_55_P2110978	*Atp6ap1*	ATPase, H+ transporting, lysosomal accessory protein 1	1,47	2,78	8,36E-07	9,30E-05	NM_018794	NM_018794	NM_018794	Mm.489665	54411	ENSMUST00000144506
A_52_P428125	*Atr*	ataxia telangiectasia and Rad3 related	1,07	2,10	2,36E-05	6,03E-04	NM_019864	NM_019864	NM_019864	Mm.212462	245000	ENSMUST00000034980
A_52_P500244	*Atrn*	attractin	0,68	1,61	1,94E-05	5,41E-04	NM_009730	NM_009730	NM_009730	Mm.119936	11990	ENSMUST00000028781
A_51_P413366	*Atrx*	alpha thalassemia/mental retardation syndrome X-linked hom	1,76	3,39	4,26E-08	2,20E-05	NM_009530	NM_009530	NM_009530	Mm.10141	22589	ENSMUST00000113573
A_66_P120901	*Atxn10*	ataxin 10	1,08	2,12	7,52E-05	1,25E-03	NM_016843	NM_016843	NM_016843	Mm.248906	54138	ENSMUST00000163242
A_51_P129866	*Atxn1l*	ataxin 1-like	0,84	1,79	8,04E-05	1,30E-03	NM_00108093	NM_00108093	NM_00108093	Mm.276770	52335	ENSMUST00000093162
A_55_P2086983	*Atxn2*	ataxin 2	0,72	1,64	2,35E-05	6,03E-04	NM_009125	NM_009125	NM_009125	Mm.260900	20239	ENSMUST00000051950
A_51_P166339	*Avil*	advillin	1,39	2,61	2,13E-06	1,46E-04	NM_009635	NM_009635	NM_009635	Mm.10739	11567	ENSMUST00000129173
A_55_P2168692	*Azi1*	5-azacytidine induced gene 1	0,64	1,56	5,29E-05	1,00E-03	NM_009734	NM_009734	NM_009734	Mm.2556	12009	ENSMUST00000145641
A_52_P24631	*Azin1*	antizyme inhibitor 1	1,31	2,48	1,72E-05	5,03E-04	NM_018745	NM_018745	NM_018745	Mm.250214	54375	ENSMUST00000129589
A_52_P236755	*B3gat2*	beta-1,3-glucuronyltransferase 2 (glucuronosyltransferase S)	0,90	1,86	1,48E-05	4,60E-04	NM_172124	NM_172124	NM_172124	Mm.471661	280645	ENSMUST00000140583
A_65_P16680	*B4galt5*	UDP-Gal:betaGlcNAc beta 1,4-galactosyltransferase, polypep	0,57	1,48	4,27E-05	8,70E-04	NM_019835	NM_019835	NM_019835	Mm.200886	56336	ENSMUST00000109221
A_51_P283649	*Baat1*	BRCA1-associated ATM activator 1	0,87	1,82	9,14E-06	3,50E-04	NM_172724	NM_172724	NM_172724	Mm.271932	231841	ENSMUST00000110806
A_51_P133761	*Bai3*	brain-specific angiogenesis inhibitor 3	1,22	2,32	5,00E-06	2,43E-04	NM_175642	NM_175642	NM_175642	Mm.336569	210933	ENSMUST00000153568
A_55_P2139326	*Baiap3*	BAI1-associated protein 3	0,91	1,88	1,70E-05	4,99E-04	NM_00116327	NM_00116327	NM_00116327	Mm.328859	545192	ENSMUST00000169109
A_51_P463828	*Baz1b*	bromodomain adjacent to zinc finger domain, 1B	0,90	1,87	2,30E-05	5,96E-04	NM_011714	NM_011714	NM_011714	Mm.40331	22385	ENSMUST00000002825
A_55_P1996837	*Baz2b*	bromodomain adjacent to zinc finger domain, 2B	1,00	2,01	8,32E-06	3,30E-04	NM_00100118	NM_00100118	NM_00100118	Mm.228071	407823	ENSMUST00000112550
A_55_P2005868	*Bbs7*	Bardet-Biedl syndrome 7 (human)	0,86	1,81	1,54E-05	4,71E-04	NM_027810	NM_027810	NM_027810	Mm.286187	71492	ENSMUST00000040148
A_55_P2121491	*Bbs9*	Bardet-Biedl syndrome 9 (human)	0,52	1,43	1,41E-04	1,99E-03	NM_178415	NM_178415	NM_178415	Mm.176725	319845	ENSMUST00000147712
A_55_P1971604	*Bcan*	brevican	0,71	1,64	1,44E-04	1,93E-03	NM_00110975	NM_00110975	NM_00110975	Mm.4598	12032	0
A_55_P2127844	*Bcas3*	breast carcinoma amplified sequence 3	1,41	2,65	6,20E-07	8,24E-05	NM_00116664	NM_00116664	NM_00116664	Mm.287663	192197	ENSMUST00000144276
A_52_P161495	*Bcl6*	B cell leukemia/lymphoma 6	0,53	1,44	1,10E-04	1,61E-03	NM_009744	NM_009744	NM_009744	Mm.347398	12053	ENSMUST00000023151
A_51_P183630	*Bcor*	BCL6 interacting corepressor	1,14	2,20	1,98E-06	1,41E-04	NM_029510	NM_029510	NM_029510	Mm.196328	71458	ENSMUST00000043441
A_51_P162437	*Bcr*	breakpoint cluster region	0,58	1,49	1,15E-04	1,66E-03	NM_00108141	NM_00108141	NM_00108141	Mm.485801	110279	ENSMUST00000164107
A_55_P2099232	*Bdp1*	B double prime 1, subunit of RNA polymerase III transcription	0,95	1,93	1,71E-05	5,01E-04	NM_00108106	NM_00108106	NM_00108106	Mm.288546	544971	ENSMUST00000099262
A_51_P365378	*Best2*	bestrophin 2	1,23	2,35	8,29E-06	3,29E-04	NM_00113019	NM_00113019	NM_00113019	Mm.215154	212989	ENSMUST00000059072
A_51_P297586	*Birc6*	baculoviral IAP repeat-containing 6	2,12	4,35	1,37E-08	1,33E-05	NM_007566	NM_007566	NM_007566	Mm.290908	12211	ENSMUST00000024879
A_51_P480904	*Blmh*	bleomycin hydrolase	0,66	1,58	1,99E-05	5,48E-04	NM_178645	NM_178645	NM_178645	Mm.399785	104184	ENSMUST00000021197
A_51_P488196	*Bmper*	BMP-binding endothelial regulator	1,09	2,13	2,49E-06	1,60E-04	NM_028472	NM_028472	NM_028472	Mm.335020	73230	ENSMUST00000071982
A_52_P208600	*Bms1*	BMS1 homolog, ribosome assembly protein (yeast)	0,94	1,91	1,50E-05	4,65E-04	NM_194339	NM_194339	NM_194339	Mm.25269	213895	ENSMUST00000032237
A_55_P2097244	*Bptf*	bromodomain PHD finger transcription factor	1,11	2,17	3,04E-07	5,93E-05	NM_176850	NM_176850	NM_176850	Mm.343986	207165	ENSMUST00000147816
A_52_P18267	*Brca1*	breast cancer 1	1,41	2,66	1,52E-06	1,22E-04	NM_009764	NM_009764	NM_009764	Mm.244975	12189	ENSMUST00000017290
A_55_P1986993	*Brd8*	bromodomain containing 8	0,89	1,85	1,30E-05	4,27E-04	NM_030147	NM_030147	NM_030147	Mm.45602	78656	ENSMUST00000003876
A_55_P2168466	*Brox*	BRO1 domain and CAAX motif containing	0,82	1,77	3,32E-05	7,41E-04	NM_027861	NM_027861	NM_027861	Mm.392270	71678	ENSMUST00000163528
A_51_P181538	*Brwd1*	bromodomain and WD repeat domain containing 1	0,90	1,87	4,23E-05	8,64E-04	NM_145125	NM_145125	NM_145125	Mm.240871	93871	ENSMUST00000023631
A_52_P442169	*Bsn*	bassoon	1,36	2,57	3,70E-05	7,91E-04	NM_007567	NM_007567	NM_007567	Mm.20425	12217	ENSMUST00000035208
A_55_P2082434	*Btbd11*	BTB (POZ) domain containing 11	0,50	1,42	6,50E-05	1,14E-03	NM_028709	NM_028709	NM_028709	Mm.36544	74007	ENSMUST00000128338
A_52_P507214	*Btbd7*	BTB (POZ) domain containing 7	0,57	1,49	3,55E-05	7,70E-04	NM_172806	NM_172806	NM_172806	Mm.440255	238386	ENSMUST00000045652
A_55_P2040539	*Btbd9*	BTB (POZ) domain containing 9	1,37	2,58	3,48E-06	1,96E-04	NM_172618	NM_172618	NM_172618	Mm.41220	224671	ENSMUST00000079924
A_55_P2004026	*Btrc*	beta-transducin repeat containing protein	0,73	1,66	2,28E-05	5,93E-04	NM_00103775	NM_00103775	NM_00103775	Mm.119717	12234	ENSMUST00000065601
A_51_P123405	*Bub1*	budding uninhibited by benzimidazoles 1 homolog (S. cerevisi	0,78	1,71	1,16E-04	1,67E-03	NM_009772	NM_009772	NM_009772	Mm.2185	12235	ENSMUST00000028858
A_51_P503303	*C1galt1c1*	C1GALT1-specific chaperone 1	0,48	1,40	1,30E-04	1,35E-03	NM_021550	NM_021550	NM_021550	Mm.441410	59048	ENSMUST00000058265
A_51_P351923	*C1qb*	complement component 1, q subcomponent, beta polypeptide	0,72	1,65	1,03E-04	1,54E-03	NM_009777	NM_009777	NM_009777	Mm.2570	12260	ENSMUST00000046384
A_55_P1985259	*C2cd3*	C2 calcium-dependent domain containing 3	0,93	1,90	1,00E-05	3,68E-04	NM_00101796	NM_00101796	NM_00101796	Mm.276374	277939	ENSMUST00000098259
A_55_P2078633	*C4b*	complement component 4B (Chido blood group)	0,68	1,60	3,48E-05	7,63E-04	NM_009780	NM_009780	NM_009780	Mm.477109	12268	ENSMUST00000069507
A_52_P93422	*Cabin1*	calcineurin binding protein 1	1,08	2,11	3,76E-06	2,06E-04	NM_172549	NM_172549	NM_172549	Mm.257404	104248	ENSMUST00000001712
A_52_P175028	*Cachd1*	cache domain containing 1	0,67	1,59	2,63E-05	6,47E-04	ENSMUST000	0	AK046180	Mm.151962	320508	ENSMUST00000030237
A_52_P1180358	*Cacna1e*	calcium channel, voltage-dependent, R type, alpha 1E subuni	0,98	1,98	6,13E-05	1,10E-03	NM_009782	NM_009782	NM_009782	Mm.267517	12290	ENSMUST00000004214
A_51_P498720	*Cacna2d1*	calcium channel, voltage-dependent, alpha2/delta subunit 1	0,87	1,83	1,49E-05	4,62E-04	NM_009784	NM_009784	NM_009784	Mm.159842	12293	ENSMUST00000039370
A_51_P502437	*Cacna2d3*	calcium channel, voltage-dependent, alpha2/delta subunit 3	0,98	1,97	1,91E-05	5,35E-04	NM_009785	NM_009785	NM_009785	Mm.386754	12294	ENSMUST00000022567
A_55_P2076749	*Cad*	carbamoyl-phosphate synthetase 2, aspartate transcarbamyla	1,59	3,00	6,01E-08	2,64E-05	NM_023525	NM_023525	NM_023525	Mm.305535	69719	ENSMUST00000013773
A_55_P2114118	*Cadm1*	cell adhesion molecule 1	0,92	1,90	2,56E-05	6,36E-04	NM_207675	NM_207675	NM_207675	Mm.234832	54725	ENSMUST00000085909
A_55_P2142928	*Cadm2*	cell adhesion molecule 2	1,15	2,22	1,08E-05	3,82E-04	NM_178721	NM_178721	NM_178721	Mm.394300	239857	ENSMUST00000120898
A_51_P503937	*Calm1*	calmodulin 1	0,88	1,84	1,39E-04	1,88E-03	NM_009790	NM_009790	NM_009790	Mm.285993	12313	ENSMUST00000110082
A_55_P2065385	*Camsap1*	calmodulin regulated spectrin-associated protein 1	0,92	1,90	4,67E-06	2,33E-04	NM_00111507	NM_00111507	NM_00111507	Mm.36834	227634	ENSMUST00000091268
A_55_P2139905	*Camsap3*	calmodulin regulated spectrin-associated protein family, meml	1,03	2,05	1,26E-05	4,16E-04	NM_00116374	NM_00116374	NM_00116374	Mm.390010	69697	ENSMUST00000171962
A_55_P2023707	*Camta1*	calmodulin binding transcription activator 1	1,00	2,00	1,41E-05	4,48E-04	NM_00108155	NM_00108155	NM_00108155	Mm.318846	100072	ENSMUST00000097774
A_52_P57308	*Cand1*	cullin associated and neddylation disassociated 1	0,87	1,83	3,24E-05	7,30E-04	NM_027994	NM_027994	NM_027994	Mm.203965	71902	ENSMUST00000020315
A_52_P644264	*Capn12*	calpain 12	1,81	3,50	6,69E-07	8,47E-05	NM_00111080	NM_00111080	NM_00111080	Mm.476990	60594	ENSMUST00000066880
A_51_P184100	*Capn7*	calpain 7	1,14	2,21	4,00E-06	2,13E-04	NM_009796	NM_009796	NM_009796	Mm.201535	12339	ENSMUST00000022451
A_55_P2070194	*Caprin1*	cell cycle associated protein 1	0,75	1,69	3,10E-05	7,11E-04	NM_016739	NM_016739	NM_016739	Mm.427589	53872	ENSMUST00000028607
A_66_P117495	*Capza1*	capping protein (actin filament) muscle Z-line, alpha 1	0,74	1,67	7,87E-05	1,29E-03	NM_009797	NM_009797	NM_009797	Mm.19142	12340	ENSMUST00000094028
A_51_P496845	*Capza2*	capping protein (actin filament) muscle Z-line, alpha 2	0,89	1,86	5,51E-05	1,03E-03	NM_007604	NM_007604	NM_007604	Mm.392504	12343	ENSMUST00000015877
A_52_P208649	*Cartpt*	CART prepropeptide	1,54	2,90	5,57E-08	2,53E-05	NM_013732	NM_013732	NM_013732	Mm.75498	27220	ENSMUST00000022150
A_55_P2052789	*Cask*	calcium/calmodulin-dependent serine protein kinase (MAGUK	1,19	2,28	3,21E-05	7,26E-04	NM_009806	NM_009806	NM_009806	Mm.327591	12361	ENSMUST00000152358
A_51_P191611	*Cat*	catalase	0,79	1,72	1,43E-05	4,52E-04	NM_009804	NM_009804	NM_009804	Mm.4215	12359	ENSMUST00000028610
A_51_P443364	*Cbll1*	Casitas B-lineage lymphoma-like 1	0,56	1,47	6,23E-05	1,11E-03	NM_134048	NM_134048	NM_134048	Mm.273270	104836	ENSMUST00000101499
A_52_P249856	*Cc2d2a*	coiled-coil and C2 domain containing 2A	1,29	2,44	3,26E-06	1,90E-04	NM_172274	NM_172274	NM_172274	Mm.44434	231214	ENSMUST00000048150
A_55_P1981599	*Ccdc125*	coiled-coil domain containing 125	0,80	1,74	3,11E-05	7,12E-04	NM_183115	NM_183115	NM_183115	Mm.23977	76041	ENSMUST00000057325
A_51_P181002	*Ccdc132*	coiled-coil domain containing 132	0,96	1,95	1,49E-04	1,96E-03	NM_024260	NM_024260	NM_024260	Mm.45539	73288	ENSMUST00000171375
A_55_P2329180	*Ccdc148*	coiled-coil domain containing 148	0,78	1,72	1,19E-04	1,70E-03	NM_00100117	NM_00100117	NM_00100117	Mm.330234	227933	ENSMUST00000134708
A_66_P124958	*Ccdc47*	coiled-coil domain containing 47	1,14	2,20	3,06E-05	7,08E-04	NM_026009	NM_026009	NM_026009	Mm.22109	67163	ENSMUST00000106865
A_55_P2169586	*Ccdc80*	coiled-coil domain containing 80	1,25	2,38	5,04E-06	2,44E-04	NM_026439	NM_026439	NM_026439	Mm.181074	67896	ENSMUST00000099498

A_52_P600946	***Ccdc88c***	coiled-coil domain containing 88C	1,28	2,43	4,19E-06	2,18E-04	NM_026681	NM_026681	NM_026681	Mm.156217	68339	ENSMUST00000068411
A_51_P506201	***Cckbr***	cholecystokinin B receptor	0,89	1,85	3,61E-05	7,80E-04	NM_007627	NM_007627	NM_007627	Mm.44513	12426	ENSMUST00000181339
A_55_P1968723	***Ccrn4l***	CCR4 carbon catabolite repression 4-like (S. cerevisiae)	0,64	1,56	5,73E-05	1,05E-03	NM_009834	NM_009834	NM_009834	Mm.86541	12457	ENSMUST00000023849
A_66_P106478	***Cct3***	chaperonin containing Tcp1, subunit 3 (gamma)	1,21	2,31	2,32E-05	6,00E-04	NM_009836	NM_009836	NM_009836	Mm.256034	12462	ENSMUST00000167000
A_55_P2125912	***Cct6a***	chaperonin containing Tcp1, subunit 6a (zeta)	0,78	1,71	9,58E-05	1,47E-03	NM_009838	NM_009838	NM_009838	Mm.360232	12466	ENSMUST00000031402
A_52_P456279	***Cct8***	chaperonin containing Tcp1, subunit 8 (theta)	1,08	2,11	7,58E-06	3,12E-04	NM_009840	NM_009840	NM_009840	Mm.328673	12469	ENSMUST00000176241
A_55_P2174490	***Cd37***	CD37 antigen	0,87	1,83	3,74E-05	7,95E-04	NM_007645	NM_007645	NM_007645	Mm.3689	12493	ENSMUST00000128823
A_52_P383089	***Cd4***	CD4 antigen	0,76	1,69	4,00E-05	8,32E-04	NM_013488	NM_013488	NM_013488	Mm.2209	12504	ENSMUST00000145977
A_55_P2182483	***Cd47***	CD47 antigen (Rh-related antigen, integrin-associated signal t	1,68	3,20	1,34E-07	4,05E-05	NM_010581	NM_010581	NM_010581	Mm.390865	16423	ENSMUST00000114496
A_51_P337675	***Cd53***	CD53 antigen	1,08	2,12	5,83E-05	1,06E-03	NM_007651	NM_007651	NM_007651	Mm.316861	12508	ENSMUST00000038845
A_51_P320852	***Cd9***	CD9 antigen	0,83	1,78	1,50E-05	4,65E-04	NM_007657	NM_007657	NM_007657	Mm.210676	12527	ENSMUST00000032492
A_52_P263870	***Cdc23***	CDC23 cell division cycle 23	1,09	2,12	4,56E-05	9,10E-04	NM_178347	NM_178347	NM_178347	Mm.196638	52563	ENSMUST00000155307
A_55_P2129092	***Cdc42bpa***	CDC42 binding protein kinase alpha	0,78	1,71	8,92E-06	3,45E-04	NM_00103328	NM_00103328	NM_00103328	Mm.259655	226751	ENSMUST00000143350
A_52_P211737	***Cdca2***	cell division cycle associated 2	0,95	1,93	2,00E-05	5,50E-04	NM_175384	NM_175384	NM_175384	Mm.33831	108912	ENSMUST00000163100
A_52_P384718	***Cdh10***	cadherin 10	0,88	1,84	1,12E-05	3,90E-04	NM_009865	NM_009865	NM_009865	Mm.117794	320873	ENSMUST00000176146
A_55_P2067478	***Cdh6***	cadherin 6	0,85	1,81	7,47E-05	1,24E-03	NM_007666	NM_007666	NM_007666	Mm.57048	12563	ENSMUST00000036439
A_51_P518919	***Cdh7***	cadherin 7, type 2	0,90	1,86	6,58E-06	2,88E-04	NM_172853	NM_172853	NM_172853	Mm.487119	241201	ENSMUST00000172005
A_55_P1970621	***Cdh8***	cadherin 8	0,85	1,80	3,76E-05	7,98E-04	NM_007667	NM_007667	NM_007667	Mm.441131	12564	ENSMUST00000128860
A_55_P2112235	***Cdk5rap2***	CDK5 regulatory subunit associated protein 2	0,73	1,66	1,51E-04	1,98E-03	NM_145990	NM_145990	NM_145990	Mm.379344	214444	ENSMUST00000138561
A_52_P253915	***Cds2***	CDP-diacylglycerol synthase (phosphatidate cytidylyltransfera	1,01	2,01	7,52E-06	3,10E-04	NM_138651	NM_138651	NM_138651	Mm.284503	110911	ENSMUST00000103181
A_51_P202971	***Cecr6***	cat eye syndrome chromosome region, candidate 6	1,02	2,03	2,87E-06	1,75E-04	NM_033567	NM_033567	NM_033567	Mm.23088	94047	ENSMUST00000178687
A_52_P350148	***Celsr2***	cadherin, EGF LAG seven-pass G-type receptor 2 (flamingo h	0,95	1,93	5,87E-05	1,07E-03	NM_00100417	NM_00100417	NM_00100417	Mm.39728	53883	ENSMUST00000126349
A_55_P2017418	***Cfh***	complement component factor h	1,69	3,23	1,43E-07	4,10E-05	NM_009888	NM_009888	NM_009888	Mm.8655	12628	ENSMUST00000123238
A_55_P2098995	***Cfhr2***	complement factor H-related 2	2,29	4,87	6,73E-08	2,81E-05	NM_00102557	NM_00102557	NM_00102557	Mm.439660	545366	ENSMUST00000094489
A_55_P2169415	***Cgn***	cingulin	2,55	5,84	1,02E-09	4,68E-06	NM_00103771	NM_00103771	NM_00103771	Mm.87634	70737	ENSMUST00000107273
A_52_P163201	***Chadl***	chondroadherin-like	0,56	1,48	2,60E-05	6,44E-04	NM_00116432	NM_00116432	NM_00116432	Mm.393287	214685	ENSMUST00000072910
A_51_P519837	***Cherp***	calcium homeostasis endoplasmic reticulum protein	0,65	1,57	8,47E-05	1,35E-03	NM_138585	NM_138585	NM_138585	Mm.30136	27967	ENSMUST00000079510
A_51_P358316	***Chga***	chromogranin A	0,67	1,59	1,08E-04	1,59E-03	NM_007693	NM_007693	NM_007693	Mm.4137	12652	ENSMUST00000021610
A_51_P191669	***Chgb***	chromogranin B	1,06	2,09	7,30E-05	1,23E-03	NM_007694	NM_007694	NM_007694	Mm.255241	12653	ENSMUST00000028826
A_52_P441294	***Chl1***	cell adhesion molecule with homology to L1CAM	0,87	1,83	3,54E-05	7,69E-04	NM_007697	NM_007697	NM_007697	Mm.251288	12661	ENSMUST00000066905
A_55_P1989782	***Chml***	choroideremia-like	1,15	2,21	5,59E-06	2,61E-04	NM_021350	NM_021350	NM_021350	Mm.440490	12663	ENSMUST00000104984
A_51_P335758	***Chn1***	chimerin (chimaerin) 1	0,94	1,92	1,27E-04	1,77E-03	NM_029716	NM_029716	NM_029716	Mm.489675	108699	ENSMUST00000124450
A_55_P2123683	***Chrdl1***	chordin-like 1	0,62	1,54	1,45E-04	1,94E-03	NM_00111438	NM_00111438	NM_00111438	Mm.157697	83453	ENSMUST00000112878
A_55_P2055284	***Chrm2***	cholinergic receptor, muscarinic 2, cardiac	1,12	2,17	8,95E-05	1,41E-03	NM_203491	NM_203491	NM_203491	Mm.448632	243764	ENSMUST00000172278
A_52_P222026	***Chrnb2***	cholinergic receptor, nicotinic, beta polypeptide 2 (neuronal)	0,57	1,48	1,18E-04	1,68E-03	NM_009602	NM_009602	NM_009602	Mm.35088	11444	ENSMUST00000029562
A_55_P1952950	***Cic***	capicua homolog (Drosophila)	1,03	2,04	5,96E-05	1,08E-03	NM_00111013	NM_00111013	NM_00111013	Mm.28833	71722	ENSMUST00000167379
A_55_P1999633	***Cit***	citron	0,97	1,96	3,33E-05	7,41E-04	NM_007708	NM_007708	NM_007708	Mm.8321	12704	ENSMUST00000123736
A_55_P2110215	***Ckap5***	cytoskeleton associated protein 5	1,19	2,28	5,04E-07	7,31E-05	NM_00116598	NM_00116598	NM_00116598	Mm.168478	75786	ENSMUST00000099716
A_52_P385606	***Ckb***	creatine kinase, brain	0,96	1,94	1,06E-04	1,57E-03	NM_021273	NM_021273	NM_021273	Mm.16831	12709	ENSMUST00000001304
A_55_P1999197	***Clasp1***	CLIP associating protein 1	0,77	1,71	5,41E-05	1,02E-03	NM_00108127	NM_00108127	NM_00108127	Mm.138740	76707	ENSMUST00000070989
A_55_P2363983	***Clasp2***	CLIP associating protein 2	1,22	2,34	7,07E-06	3,00E-04	NM_00111434	NM_00111434	NM_00111434	Mm.222272	76499	ENSMUST00000163895
A_55_P2121222	***Clcc1***	chloride channel CLIC-like 1	0,58	1,50	5,64E-05	1,04E-03	NM_00117777	NM_00117777	NM_00117777	Mm.214545	229725	ENSMUST00000029483
A_55_P2081214	***Clcn6***	chloride channel 6	1,11	2,16	2,43E-06	1,58E-04	NM_011929	NM_011929	NM_011929	Mm.89987	26372	ENSMUST00000030879
A_51_P456465	***Cldn10***	claudin 10	0,69	1,61	1,85E-05	5,26E-04	NM_021386	NM_021386	NM_021386	Mm.390755	58187	ENSMUST00000047761
A_51_P409452	***Cldn11***	claudin 11	0,84	1,79	1,00E-05	3,68E-04	NM_008770	NM_008770	NM_008770	Mm.4425	18417	ENSMUST00000046174
A_51_P288906	***Clip1***	CAP-GLY domain containing linker protein 1	1,81	3,51	4,74E-07	7,18E-05	NM_019765	NM_019765	NM_019765	Mm.241109	56430	ENSMUST00000137602
A_51_P200667	***Clmn***	calmin	1,20	2,30	3,36E-07	6,08E-05	NM_053155	NM_053155	NM_053155	Mm.244078	94040	ENSMUST00000109937
A_52_P164136	***Cln6***	ceroid-lipofuscinosis, neuronal 6	0,69	1,62	4,29E-05	8,72E-04	NM_00103317	NM_00103317	NM_00103317	Mm.283636	76524	ENSMUST00000034776
A_51_P407193	***Clp1***	CLP1, cleavage and polyadenylation factor I subunit	1,35	2,54	1,24E-04	1,74E-03	NM_133840	NM_133840	NM_133840	Mm.21583	98985	ENSMUST00000138231
A_66_P134785	***Clpp***	ClpP caseinolytic peptidase, ATP-dependent, proteolytic subu	0,71	1,63	1,05E-04	1,56E-03	NM_017393	NM_017393	NM_017393	Mm.287892	53895	ENSMUST00000002735
A_51_P304683	***Clpx***	caseinolytic peptidase X (E.coli)	1,04	2,05	1,04E-04	1,55E-03	NM_011802	NM_011802	NM_011802	Mm.30088	270166	ENSMUST00000015501
A_52_P424784	***Clstn2***	calsyntenin 2	1,06	2,09	1,24E-06	1,10E-04	NM_022319	NM_022319	NM_022319	Mm.440903	64085	ENSMUST00000035027
A_66_P119174	***Cmtm6***	CKLF-like MARVEL transmembrane domain containing 6	0,66	1,58	5,84E-05	1,07E-03	NM_026036	NM_026036	NM_026036	Mm.28858	67213	ENSMUST00000035007
A_55_P2072816	***Cnksr2***	connector enhancer of kinase suppressor of Ras 2	0,89	1,85	1,38E-04	1,87E-03	NM_177751	NM_177751	NM_177751	Mm.197074	245684	ENSMUST00000026750
A_55_P1953753	***Cnr1***	cannabinoid receptor 1 (brain)	0,67	1,59	3,43E-05	7,54E-04	NM_007726	NM_007726	NM_007726	Mm.7992	12801	ENSMUST00000084736
A_66_P125925	***Cntn2***	contactin 2	1,19	2,28	1,93E-05	5,40E-04	NM_177129	NM_177129	NM_177129	Mm.485420	21367	ENSMUST00000086521
A_55_P2157978	***Cntn4***	contactin 4	1,21	2,31	1,42E-04	1,91E-03	NM_00110975	NM_00110975	NM_00110975	Mm.321683	269784	ENSMUST00000113261
A_52_P412452	***Cntn6***	contactin 6	1,08	2,11	5,60E-05	1,04E-03	NM_017383	NM_017383	NM_017383	Mm.321671	53870	ENSMUST00000162872
A_55_P2022128	***Cntnap1***	contactin associated protein-like 1	1,00	1,99	7,92E-07	9,12E-05	NM_016782	NM_016782	NM_016782	Mm.474527	53321	ENSMUST00000103109
A_52_P215750	***Cntnap2***	contactin associated protein-like 2	1,51	2,85	8,26E-06	3,29E-04	NM_00100435	NM_00100435	NM_00100435	Mm.440084	66797	ENSMUST00000114641
A_55_P1964653	***Cntnap4***	contactin associated protein-like 4	1,09	2,13	6,35E-06	2,83E-04	NM_130457	NM_130457	NM_130457	Mm.209232	170571	ENSMUST00000118171
A_52_P150651	***Cog5***	component of oligomeric golgi complex 5	0,88	1,85	2,85E-05	6,76E-04	NM_00116312	NM_00116312	NM_00116312	Mm.173068	238123	ENSMUST00000036862
A_51_P130824	***Cog8***	component of oligomeric golgi complex 8	0,65	1,57	6,18E-05	1,10E-03	NM_139229	NM_139229	NM_139229	Mm.288641	97484	ENSMUST00000034391
A_55_P2062469	***Col12a1***	collagen, type XII, alpha 1	0,99	1,99	1,30E-05	4,25E-04	NM_007730	NM_007730	NM_007730	Mm.3819	12816	ENSMUST00000071750
A_52_P170882	***Col15a1***	collagen, type XV, alpha 1	0,63	1,55	1,31E-04	1,81E-03	NM_009928	NM_009928	NM_009928	Mm.233547	12819	ENSMUST00000102917
A_55_P2124791	***Col18a1***	collagen, type XVIII, alpha 1	1,04	2,05	4,10E-05	8,45E-04	NM_00110999	NM_00110999	NM_00110999	Mm.4352	12822	ENSMUST00000072755
A_51_P213676	***Col19a1***	collagen, type XIX, alpha 1	0,96	1,94	1,46E-04	1,94E-03	NM_007733	NM_007733	NM_007733	Mm.329196	12823	0
A_55_P2118520	***Col1a1***	collagen, type I, alpha 1	1,51	2,84	1,54E-07	4,22E-05	NM_007742	NM_007742	NM_007742	Mm.277735	12842	ENSMUST00000001547
A_51_P182303	***Col1a2***	collagen, type I, alpha 2	1,50	2,83	1,70E-06	1,30E-04	NM_007743	NM_007743	NM_007743	Mm.277792	12843	ENSMUST00000031668
A_55_P2107715	***Col25a1***	collagen, type XXV, alpha 1	1,36	2,56	5,10E-06	2,45E-04	NM_029838	NM_029838	NM_029838	Mm.91728	77018	ENSMUST00000106353
A_55_P2004179	***Col2a1***	collagen, type II, alpha 1	2,32	4,98	2,12E-09	5,69E-06	NM_00111351	NM_00111351	NM_00111351	Mm.2423	12824	ENSMUST00000023123
A_51_P124254	***Col4a1***	collagen, type IV, alpha 1	2,73	6,65	1,49E-09	5,22E-06	NM_009931	NM_009931	NM_009931	Mm.738	12826	ENSMUST00000033898
A_51_P491350	***Col4a2***	collagen, type IV, alpha 2	2,09	4,24	3,15E-08	1,84E-05	NM_009932	NM_009932	NM_009932	Mm.181021	12827	ENSMUST00000033899
A_55_P1969367	***Col4a5***	collagen, type IV, alpha 5	1,16	2,24	2,07E-06	1,44E-04	NM_00116315	NM_00116315	NM_00116315	Mm.286892	12830	ENSMUST00000112930
A_52_P516409	***Col4a6***	collagen, type IV, alpha 6	1,39	2,62	1,13E-07	3,70E-05	NM_053185	NM_053185	NM_053185	Mm.155586	94216	ENSMUST00000101205
A_51_P414637	***Col5a1***	collagen, type V, alpha 1	1,26	2,39	3,57E-06	1,99E-04	NM_015734	NM_015734	NM_015734	Mm.7281	12831	ENSMUST00000028280
A_55_P2104894	***Col5a2***	collagen, type V, alpha 2	1,43	2,69	3,82E-07	6,44E-05	NM_007737	NM_007737	NM_007737	Mm.10299	12832	ENSMUST00000086430
A_55_P2128153	***Col6a1***	collagen, type VI, alpha 1	1,11	2,17	6,19E-07	8,24E-05	NM_009933	NM_009933	NM_009933	Mm.2509	12833	ENSMUST00000001147
A_51_P279639	***Col6a2***	collagen, type VI, alpha 2	1,23	2,35	2,64E-06	1,66E-04	NM_146007	NM_146007	NM_146007	Mm.1949	12834	ENSMUST00000001181
A_52_P479262	***Col6a3***	collagen, type VI, alpha 3	1,32	2,49	2,35E-06	1,56E-04	NM_00124300	NM_00124300	NM_00124300	Mm.7562	12835	ENSMUST00000097653
A_55_P2064292	***Col7a1***	collagen, type VII, alpha 1	1,01	2,02	6,24E-07	8,24E-05	NM_007738	NM_007738	NM_007738	Mm.6200	12836	ENSMUST00000026740
A_52_P48777	***Copg2***	coatomer protein complex, subunit gamma 2	0,91	1,88	2,28E-05	5,93E-04	NM_017478	NM_017478	NM_017478	Mm.381860	54160	ENSMUST00000048774
A_51_P328014	***Cops3***	COP9 (constitutive photomorphogenic) homolog, subunit 3 (A	0,72	1,65	1,04E-04	1,55E-03	NM_011991	NM_011991	NM_011991	Mm.40	26572	ENSMUST00000019517
A_51_P371152	***Cops4***	COP9 (constitutive photomorphogenic) homolog, subunit 4 (A	0,76	1,70	5,03E-05	9,67E-04	NM_012001	NM_012001	NM_012001	Mm.957	26891	ENSMUST00000123559
A_55_P2111302	***Cp***	ceruloplasmin	0,84	1,79	1,81E-05	5,19E-04	NM_007752	NM_007752	NM_007752	Mm.13787	12870	ENSMUST00000129135
A_52_P359965	***Cpd***	carboxypeptidase D	0,73	1,66	1,07E-04	1,58E-03	NM_007754	NM_007754	NM_007754	Mm.276736	12874	ENSMUST00000021201
A_55_P2152248	***Cpe***	carboxypeptidase E	1,20	2,29	1,47E-04	1,95E-03	NM_013494	NM_013494	NM_013494	Mm.31395	12876	ENSMUST00000048967
A_52_P466090	***Cplx1***	complexin 1	0,65	1,57	1,19E-04	1,70E-03	NM_007756	NM_007756	NM_007756	Mm.5195	12889	ENSMUST00000046892
A_52_P565051	***Cpne3***	copine III	0,97	1,95	3,17E-05	7,21E-04	NM_027769	NM_027769	NM_027769	Mm.38390	70568	ENSMUST00000108254
A_51_P506045	***Cpq***	carboxypeptidase Q	0,95	1,93	1,16E-05	3,98E-04	NM_176073	NM_176073	NM_176073	Mm.158251	54381	ENSMUST00000042167
A_52_P272811	***Cpsf1***	cleavage and polyadenylation specific factor 1	1,44	2,71	1,55E-07	4,22E-05	NM_053193	NM_053193	NM_053193	Mm.45141	94230	ENSMUST00000071898
A_51_P479894	***Cpsf3***	cleavage and polyadenylation specificity factor 3	0,71	1,64	2,19E-05	5,83E-04	NM_018813	NM_018813	NM_018813	Mm.356778	54451	ENSMUST00000067284
A_52_P126825	***Cramp1l***	Crm, cramped-like (Drosophila)	0,73	1,66	1,44E-05	4,53E-04	NM_020608	NM_020608	NM_020608	Mm.328854	57354	ENSMUST00000073337
A_55_P2137979	***Crb3***	crumbs homolog 3 (Drosophila)	1,06	2,08	2,94E-05	6,90E-04	NM_177638	NM_177638	NM_177638	Mm.391027	224912	ENSMUST00000097299
A_55_P2054540	***Crebbp***	CREB binding protein	0,96	1,94	3,73E-05	7,95E-04	AK048818	0	AK048818	Mm.392384	12914	0
A_51_P314418	***Crip2***	cysteine rich protein 2	0,81	1,75	3,21E-05	7,27E-04	NM_024223	NM_024223	NM_024223	Mm.133825	68337	ENSMUST00000084882
A_55_P2017636	***Crmp1***	collapsin response mediator protein 1	0,69	1,62	1,04E-04	1,55E-03	NM_00113605	NM_00113605	NM_00113605	Mm.290995	12933	ENSMUST00000114158
A_55_P2066007	***Crocc***	ciliary rootlet coiled-coil, rootletin	1,70	3,26	6,25E-08	2,68E-05	NM_172122	NM_172122	NM_172122	Mm.80685	230872	ENSMUST00000040222
A_51_P489153	***Crot***	carnitine O-octanoyltransferase	0,96	1,95	1,48E-04	1,95E-03	NM_023733	NM_023733	NM_023733	Mm.28197	74114	ENSMUST00000003720
A_51_P160439	***Crybg3***	beta-gamma crystallin domain containing 3	0,77	1,71	3,47E-05	7,61E-04	NM_174848	NM_174848	NM_174848	Mm.246424	224273	ENSMUST00000044604
A_55_P2068330	***Cryzl1***	crystallin, zeta (quinone reductase)-like 1	0,79	1,73	4,82E-06	2,37E-04	NM_026994	NM_026994	NM_026994	Mm.109823	66609	ENSMUST00000114023
A_52_P182659	***Cs***	citrate synthase	0,95	1,93	8,87E-06	3,44E-04	NM_026444	NM_026444	NM_026444	Mm.58836	12974	ENSMUST00000005826
A_51_P268529	***Csad***	cysteine sulfinic acid decarboxylase	0,66	1,58	1,36E-04	1,85E-03	NM_144942	NM_144942	NM_144942	Mm.296382	246277	ENSMUST00000023805
A_51_P248705	***Cse1l***	chromosome segregation 1-like (S. cerevisiae)	0,93	1,91	2,19E-05	5,83E-04	NM_023565	NM_023565	NM_023565	Mm.22417	110750	ENSMUST00000171410
A_52_P602091	***Csf1r***	colony stimulating factor 1 receptor	1,39	2,61	1,09E-06	1,04E-04	NM_00103785	NM_00103785	NM_00103785	Mm.22574	12978	ENSMUST00000025523
A_51_P341465	***Csf2ra***	colony stimulating factor 2 receptor, alpha, low-affinity (granulo	0,70	1,62	5,27E-05	9,98E-04	NM_009970	NM_009970	NM_009970	0	12982	ENSMUST00000076046
A_55_P2063422	***Csmd1***	CUB and Sushi multiple domains 1	2,06	4,16	1,48E-08	1,34E-05	NM_053171	NM_053171	NM_053171	Mm.436988	94109	ENSMUST00000125551
A_51_P476960	***Csmd3***	CUB and Sushi multiple domains 3	1,88	3,68	5,83E-08	2,60E-05	NM_00108139	NM_00108139	NM_00108139	Mm.309363	239420	ENSMUST00000110261
A_51_P397768	***Csnk2a1***	casein kinase 2, alpha 1 polypeptide	0,81	1,75	8,36E-06	3,31E-04	NM_007788	NM_007788	NM_007788	Mm.23692	12995	ENSMUST00000099224
A_55_P2080703	***Cstf2***	cleavage stimulation factor, 3' pre-RNA subunit 2	1,20	2,29	1,96E-06	1,40E-04	ENSMUST000	0	BC036719	Mm.67938	108062	ENSMUST00000113286

A_55_P2032760	Ctif	CBP80/20-dependent translation initiation factor	0,67	1,59	1,48E-04	1,95E-03	NM_201354	NM_201354	NM_201354	Mm.36745	269037	ENSMUST00000165559
A_51_P324572	Ctnna1	catenin (cadherin associated protein), alpha 1	0,64	1,56	1,45E-04	1,93E-03	NM_009818	NM_009818	NM_009818	Mm.18962	12385	ENSMUST00000042345
A_51_P438841	Ctnna2	catenin (cadherin associated protein), alpha 2	1,21	2,32	8,88E-07	9,65E-05	NM_009819	NM_009819	NM_009819	Mm.34637	12386	ENSMUST00000159626
A_52_P424585	Ctnnb1	catenin (cadherin associated protein), beta 1	1,58	2,99	1,68E-07	4,37E-05	NM_007614	NM_007614	NM_007614	Mm.291928	12387	ENSMUST00000154356
A_55_P2024215	Ctnnbl1	catenin, beta like 1	0,94	1,92	1,28E-05	4,22E-04	NM_025680	NM_025680	NM_025680	Mm.45193	66642	ENSMUST00000029178
A_55_P1953252	Ctnnd1	catenin (cadherin associated protein), delta 1	0,94	1,91	4,24E-05	8,65E-04	NM_007615	NM_007615	NM_007615	Mm.35738	12388	ENSMUST00000111694
A_52_P195839	Ctsc	cathepsin C	0,91	1,88	3,21E-05	7,27E-04	NM_009982	NM_009982	NM_009982	Mm.322945	13032	ENSMUST00000032779
A_65_P13209	Ctsd	cathepsin D	1,67	3,19	1,34E-05	4,35E-04	NM_009983	NM_009983	NM_009983	Mm.231395	13033	ENSMUST00000066401
A_51_P253547	Ctsl	cathepsin L	0,73	1,66	7,57E-05	1,25E-03	NM_009984	NM_009984	NM_009984	Mm.930	13039	ENSMUST00000021933
A_52_P326214	Cttn	cortactin	1,41	2,65	5,88E-06	2,70E-04	NM_007803	NM_007803	NM_007803	Mm.205601	13043	ENSMUST00000103079
A_52_P481423	Cttnbp2nl	CTTNBP2 N-terminal like	1,04	2,06	5,52E-05	1,03E-03	NM_030249	NM_030249	NM_030249	Mm.200327	80281	ENSMUST00000077548
A_52_P664699	Cul1	cullin 1	0,78	1,71	1,03E-04	1,55E-03	NM_012042	NM_012042	NM_012042	Mm.87611	26965	ENSMUST00000146200
A_55_P2121081	Cul2	cullin 2	0,97	1,97	5,11E-05	9,78E-04	NM_029402	NM_029402	NM_029402	Mm.291707	71745	ENSMUST00000080089
A_52_P47645	Cul3	cullin 3	1,29	2,45	4,78E-06	2,36E-04	NM_016716	NM_016716	NM_016716	Mm.12665	26554	ENSMUST00000167794
A_55_P2056958	Cul9	cullin 9	1,54	2,92	3,36E-07	6,08E-05	NM_00108133	NM_00108133	NM_00108133	Mm.329076	78309	ENSMUST00000066026
A_52_P99810	Cx3cr1	chemokine (C-X3-C) receptor 1	0,63	1,55	9,20E-05	1,43E-03	NM_009987	NM_009987	NM_009987	Mm.44065	13051	ENSMUST00000177637
A_52_P585124	Cxcr4	chemokine (C-X-C motif) receptor 4	0,85	1,81	8,10E-05	1,31E-03	NM_009911	NM_009911	NM_009911	Mm.1401	12767	ENSMUST00000052172
A_55_P1956667	Cyfip1	cytoplasmic FMR1 interacting protein 1	0,83	1,77	5,70E-05	1,05E-03	NM_00116466	NM_00116466	NM_00116466	Mm.37249	20430	ENSMUST00000032629
A_55_P2102515	Daam1	dishevelled associated activator of morphogenesis 1	0,84	1,79	2,63E-05	6,47E-04	NM_026102	NM_026102	NM_026102	Mm.87417	208846	ENSMUST00000085299
A_52_P392216	Dab1	disabled 1	1,53	2,88	1,51E-06	1,22E-04	NM_177259	NM_177259	NM_177259	Mm.289682	13131	ENSMUST00000106830
A_55_P1959763	Dach1	dachshund 1 (Drosophila)	0,87	1,83	1,52E-06	1,22E-04	NM_007826	NM_007826	NM_007826	Mm.320593	13134	ENSMUST00000071533
A_52_P127682	Dagla	diacylglycerol lipase, alpha	0,69	1,61	3,17E-05	7,21E-04	NM_198114	NM_198114	NM_198114	Mm.329718	269060	ENSMUST00000125567
A_55_P2195157	Dbn1	drebrin 1	4,40	21,17	2,38E-10	3,00E-06	NM_00117737	NM_00117737	NM_00117737	Mm.19016	56320	ENSMUST00000109921
A_52_P569348	Dbt	dihydrolipoamide branched chain transacylase E2	0,63	1,55	7,50E-05	1,25E-03	NM_010022	NM_010022	NM_010022	Mm.3636	13171	ENSMUST00000000349
A_55_P2020497	Dcaf12l1	DDB1 and CUL4 associated factor 12-like 1	0,94	1,92	7,75E-06	3,16E-04	NM_178739	NM_178739	NM_178739	Mm.55685	245404	ENSMUST00000060481
A_52_P471502	Dcaf13	DDB1 and CUL4 associated factor 13	0,95	1,93	1,17E-04	1,68E-03	NM_198606	NM_198606	NM_198606	Mm.321937	223499	ENSMUST00000022909
A_66_P130906	Dcaf7	DDB1 and CUL4 associated factor 7	1,09	2,12	2,87E-05	6,78E-04	NM_027946	NM_027946	NM_027946	Mm.307455	71833	ENSMUST00000106891
A_52_P46310	Dcaf7	DDB1 and CUL4 associated factor 7	0,74	1,67	7,12E-05	1,21E-03	NM_027946	NM_027946	NM_027946	Mm.307455	71833	ENSMUST00000058438
A_65_P07978	Dcc	deleted in colorectal carcinoma	1,03	2,05	1,09E-05	3,84E-04	NM_007831	NM_007831	NM_007831	Mm.167882	13176	0
A_51_P270355	Dclk1	doublecortin-like kinase 1	0,92	1,89	2,85E-06	1,75E-04	NM_019978	NM_019978	NM_019978	Mm.393242	13175	ENSMUST00000054237
A_55_P1975385	Dctn1	dynactin 1	0,95	1,94	1,27E-05	4,20E-04	NM_007835	NM_007835	NM_007835	Mm.6919	13191	ENSMUST00000077407
A_55_P2046084	Dcun1d4	DCN1, defective in cullin neddylation 1, domain containing 4 (	0,69	1,61	2,81E-05	6,72E-04	NM_178896	NM_178896	NM_178896	Mm.220312	100737	ENSMUST00000113558
A_51_P110471	Ddah1	dimethylarginine dimethylaminohydrolase 1	0,74	1,67	4,97E-05	9,60E-04	NM_026993	NM_026993	NM_026993	Mm.234247	69219	ENSMUST00000127193
A_51_P387220	Ddx1	DEAD (Asp-Glu-Ala-Asp) box polypeptide 1	1,01	2,02	1,49E-05	4,62E-04	NM_134040	NM_134040	NM_134040	Mm.251255	104721	ENSMUST00000071103
A_51_P402994	Ddx3y	DEAD (Asp-Glu-Ala-Asp) box polypeptide 3, Y-linked	0,96	1,95	5,60E-06	2,61E-04	NM_012008	NM_012008	NM_012008	Mm.486436	26900	ENSMUST00000091190
A_51_P491470	Ddx47	DEAD (Asp-Glu-Ala-Asp) box polypeptide 47	0,81	1,75	1,25E-04	1,75E-03	NM_026360	NM_026360	NM_026360	Mm.166524	67755	ENSMUST00000155022
A_51_P141164	Ddx50	DEAD (Asp-Glu-Ala-Asp) box polypeptide 50	0,73	1,66	3,41E-05	7,53E-04	NM_053183	NM_053183	NM_053183	Mm.114116	94213	ENSMUST00000020270
A_51_P497463	Dedd	death effector domain-containing	0,75	1,68	5,93E-05	1,08E-03	NM_011615	NM_011615	NM_011615	Mm.270139	21945	ENSMUST00000097467
A_51_P155085	Dennd2a	DENN/MADD domain containing 2A	1,27	2,41	2,04E-07	4,69E-05	NM_172477	NM_172477	NM_172477	Mm.440021	209773	ENSMUST00000036877
A_52_P474902	Denr	density-regulated protein	0,60	1,51	6,79E-05	1,17E-03	NM_026603	NM_026603	NM_026603	Mm.28549	68184	ENSMUST00000166233
A_55_P1984391	Depdc5	DEP domain containing 5	0,97	1,95	2,67E-05	6,53E-04	NM_177786	NM_177786	NM_177786	Mm.101524	277854	ENSMUST00000118698
A_51_P217236	Derl1	Der1-like domain family, member 1	0,76	1,70	7,75E-05	1,27E-03	NM_024207	NM_024207	NM_024207	Mm.289387	67819	ENSMUST00000022993
A_52_P550843	Desi2	desumoylating isopeptidase 2	0,52	1,44	6,51E-05	1,14E-03	NM_024282	NM_024282	NM_024282	Mm.440132	78825	ENSMUST00000027783
A_52_P456134	Dgat1	diacylglycerol O-acyltransferase 1	0,73	1,66	2,65E-05	6,50E-04	NM_010046	NM_010046	NM_010046	Mm.22633	13350	ENSMUST00000162354
A_55_P2172935	Dgkb	diacylglycerol kinase, beta	1,02	2,02	1,76E-05	5,10E-04	AK038544	0	AK038544	Mm.126525	217480	0
A_55_P2022337	Dhcr7	7-dehydrocholesterol reductase	0,67	1,59	6,70E-05	1,16E-03	NM_007856	NM_007856	NM_007856	Mm.249342	13360	ENSMUST00000073878
A_51_P293901	Dhrs1	dehydrogenase/reductase (SDR family) member 1	0,70	1,63	1,23E-04	1,74E-03	NM_026819	NM_026819	NM_026819	Mm.21623	52585	ENSMUST00000002403
A_51_P312437	Dhrs7	dehydrogenase/reductase (SDR family) member 7	0,68	1,60	5,55E-05	1,03E-03	NM_025522	NM_025522	NM_025522	Mm.289653	66375	ENSMUST00000021512
A_55_P2040888	Dhx15	DEAH (Asp-Glu-Ala-His) box polypeptide 15	1,16	2,23	7,41E-05	1,24E-03	NM_00104262	NM_00104262	NM_00104262	Mm.993	13204	ENSMUST00000031061
A_66_P117838	Dhx29	DEAH (Asp-Glu-Ala-His) box polypeptide 29	0,89	1,85	2,73E-06	1,68E-04	NM_172594	NM_172594	NM_172594	Mm.35094	218629	ENSMUST00000038574
A_55_P2036803	Dhx30	DEAH (Asp-Glu-Ala-His) box polypeptide 30	0,79	1,73	4,11E-05	8,46E-04	NM_00125268	NM_00125268	NM_00125268	Mm.276305	72831	ENSMUST00000111991
A_55_P2351505	Dhx9	DEAH (Asp-Glu-Ala-His) box polypeptide 9	1,71	3,27	4,61E-07	7,09E-05	NM_007842	NM_007842	NM_007842	Mm.20000	13211	ENSMUST00000042141
A_51_P427768	Diap1	diaphanous homolog 1 (Drosophila)	0,69	1,62	5,14E-05	9,82E-04	NM_007858	NM_007858	NM_007858	Mm.195916	13367	ENSMUST00000080033
A_51_P192162	Diap2	diaphanous homolog 2 (Drosophila)	0,77	1,71	7,40E-05	1,24E-03	NM_172493	NM_172493	NM_172493	Mm.10763	54004	ENSMUST00000167619
A_51_P336385	Dido1	death inducer-obliterator 1	1,43	2,69	6,16E-05	1,10E-03	NM_175551	NM_175551	NM_175551	Mm.253836	23856	ENSMUST00000087517
A_55_P1999361	Dip2a	DIP2 disco-interacting protein 2 homolog A (Drosophila)	1,13	2,19	7,46E-06	3,10E-04	NM_00108141	NM_00108141	NM_00108141	Mm.33183	64451	ENSMUST00000105417
A_52_P430169	Dip2b	DIP2 disco-interacting protein 2 homolog B (Drosophila)	1,41	2,65	3,60E-06	2,00E-04	NM_00115936	NM_00115936	NM_00115936	Mm.243658	239667	ENSMUST00000108971
A_55_P2137376	Dip2c	DIP2 disco-interacting protein 2 homolog C (Drosophila)	0,69	1,62	3,34E-05	7,43E-04	NM_00108142	NM_00108142	NM_00108142	Mm.217326	208440	ENSMUST00000174552
A_52_P627068	Disp2	dispatched homolog 2 (Drosophila)	1,64	3,11	4,47E-06	2,27E-04	NM_170593	NM_170593	NM_170593	Mm.221499	214240	ENSMUST00000037547
A_55_P2120807	Dld	dihydrolipoamide dehydrogenase	0,98	1,97	1,37E-05	4,41E-04	NM_007861	NM_007861	NM_007861	Mm.3131	13382	ENSMUST00000110857
A_51_P101858	Dlg1	discs, large homolog 1 (Drosophila)	1,13	2,19	2,52E-06	1,61E-04	NM_007862	NM_007862	NM_007862	Mm.382	13383	ENSMUST00000115201
A_55_P2072238	Dlg2	discs, large homolog 2 (Drosophila)	1,47	2,77	3,38E-06	1,93E-04	NM_011807	NM_011807	NM_011807	Mm.257035	23859	ENSMUST00000074273
A_55_P1999947	Dlgap2	discs, large (Drosophila) homolog-associated protein 2	0,80	1,74	1,39E-05	4,44E-04	NM_172910	NM_172910	NM_172910	Mm.404697	244310	ENSMUST00000133298
A_52_P411003	Dlgap5	discs, large (Drosophila) homolog-associated protein 5	0,78	1,71	3,83E-05	8,09E-04	NM_144553	NM_144553	NM_144553	Mm.240543	218977	ENSMUST00000043296
A_52_P306697	Dmd	dystrophin, muscular dystrophy	0,97	1,96	1,14E-05	3,93E-04	NM_007868	NM_007868	NM_007868	Mm.275608	13405	ENSMUST00000149433
A_55_P2179939	Dmxl2	Dmx-like 2	1,51	2,84	1,74E-06	1,31E-04	NM_172771	NM_172771	NM_172771	Mm.93636	235380	ENSMUST00000118600
A_55_P1964076	Dnajb6	DnaJ (Hsp40) homolog, subfamily B, member 6	0,64	1,56	4,23E-05	8,65E-04	NM_00103794	NM_00103794	NM_00103794	Mm.290110	23950	ENSMUST00000012734
A_66_P133450	Dnajb9	DnaJ (Hsp40) homolog, subfamily B, member 9	1,01	2,02	1,41E-05	4,48E-04	NM_013760	NM_013760	NM_013760	Mm.27432	27362	ENSMUST00000015049
A_52_P250590	Dnajc13	DnaJ (Hsp40) homolog, subfamily C, member 13	1,00	1,99	5,53E-06	2,59E-04	NM_00116302	NM_00116302	NM_00116302	Mm.217256	235567	ENSMUST00000035170
A_55_P2146304	Dnajc6	DnaJ (Hsp40) homolog, subfamily C, member 6	1,30	2,47	6,45E-06	2,85E-04	NM_00116458	NM_00116458	NM_00116458	Mm.76494	72685	ENSMUST00000106929
A_55_P2079175	Dnm1	dynamin 1	0,67	1,59	2,76E-05	6,65E-04	NM_010065	NM_010065	NM_010065	Mm.44736	13429	ENSMUST00000156866
A_55_P2100679	Dnm2	dynamin 2	0,85	1,80	1,29E-04	1,80E-03	NM_00125389	NM_00125389	NM_00125389	Mm.433257	13430	ENSMUST00000173299
A_52_P164286	Dnm3	dynamin 3	0,96	1,94	1,06E-04	1,58E-03	NM_00103861	NM_00103861	NM_00103861	Mm.441620	103967	ENSMUST00000086074
A_51_P195573	Dnmt1	DNA methyltransferase (cytosine-5) 1	1,15	2,21	8,09E-07	9,21E-05	NM_010066	NM_010066	NM_010066	Mm.128580	13433	ENSMUST00000178110
A_55_P2012849	Dock10	dedicator of cytokinesis 10	1,33	2,51	3,04E-07	5,93E-05	NM_175291	NM_175291	NM_175291	Mm.133473	210293	ENSMUST00000077946
A_51_P173735	Dock11	dedicator of cytokinesis 11	1,27	2,41	1,08E-05	3,83E-04	NM_00100994	NM_00100994	NM_00100994	Mm.32873	75974	ENSMUST00000115266
A_51_P114917	Dock2	dedicator of cyto-kinesis 2	0,78	1,71	7,37E-05	1,24E-03	NM_033374	NM_033374	NM_033374	Mm.380679	94176	ENSMUST00000093193
A_55_P2170413	Dock3	dedicator of cyto-kinesis 3	1,45	2,74	7,31E-07	8,77E-05	NM_153413	NM_153413	NM_153413	Mm.150259	208869	ENSMUST00000044532
A_52_P280114	Dock4	dedicator of cytokinesis 4	0,73	1,66	2,39E-05	6,10E-04	NM_172803	NM_172803	NM_172803	Mm.341423	238130	ENSMUST00000037488
A_52_P35961	Dohh	deoxyhypusine hydroxylase/monooxygenase	0,58	1,49	7,28E-05	1,23E-03	ENSMUST000	0	AK080664	Mm.485349	102115	ENSMUST00000144647
A_51_P284966	Dolk	dolichol kinase	0,90	1,86	1,20E-04	1,71E-03	NM_177648	NM_177648	NM_177648	Mm.26690	227697	ENSMUST00000100219
A_55_P1981739	Donson	downstream neighbor of SON	0,74	1,67	1,20E-05	4,04E-04	NM_021720	NM_021720	NM_021720	Mm.490365	60364	ENSMUST00000123893
A_52_P260787	Dopey1	dopey family member 1	1,32	2,50	7,18E-06	3,03E-04	NM_177208	NM_177208	NM_177208	Mm.245500	320615	ENSMUST00000034987
A_51_P481238	Dopey2	dopey family member 2	0,95	1,93	9,25E-06	3,52E-04	NM_027293	NM_027293	NM_027293	Mm.23230	70028	ENSMUST00000045004
A_55_P2041529	Doxl2	diamine oxidase-like protein 2	0,52	1,43	1,41E-04	1,90E-03	NM_00102998	NM_00102998	NM_00102998	Mm.244112	243376	ENSMUST00000090063
A_51_P125434	Dph1	DPH1 homolog (S. cerevisiae)	0,66	1,58	9,33E-05	1,44E-03	NM_144491	NM_144491	NM_144491	Mm.41496	116905	ENSMUST00000044949
A_55_P1953884	Dpm1	dolichol-phosphate (beta-D) mannosyltransferase 1	0,75	1,68	7,30E-05	1,23E-03	NM_010072	NM_010072	NM_010072	Mm.422657	13480	ENSMUST00000136582
A_55_P1968078	Dpp10	dipeptidylpeptidase 10	0,68	1,60	8,68E-05	1,38E-03	NM_199021	NM_199021	NM_199021	Mm.483255	269109	ENSMUST00000112606
A_52_P99807	Dpy19l3	dpy-19-like 3 (C. elegans)	0,92	1,89	4,46E-05	8,96E-04	NM_178704	NM_178704	NM_178704	Mm.257808	233115	ENSMUST00000051377
A_66_P105965	Drp2	dystrophin related protein 2	0,90	1,86	6,47E-06	2,85E-04	NM_010078	NM_010078	NM_010078	Mm.121662	13497	ENSMUST00000000228
A_52_P660693	Dsel	dermatan sulfate epimerase-like	0,68	1,60	1,01E-04	1,53E-03	NM_00108131	NM_00108131	NM_00108131	Mm.103468	319901	ENSMUST00000035462
A_51_P101460	Dsp	desmoplakin	1,01	2,02	1,18E-06	1,09E-04	NM_023842	NM_023842	NM_023842	Mm.355327	109620	ENSMUST00000124830
A_52_P445253	Dst	dystonin	2,28	4,86	4,81E-07	7,22E-05	NM_133833	NM_133833	NM_133833	Mm.336625	13518	ENSMUST00000097786
A_51_P184300	Dtna	dystrobrevin alpha	0,80	1,74	7,25E-06	3,04E-04	NM_010087	NM_010087	NM_010087	Mm.94371	13527	ENSMUST00000047954
A_55_P2103206	Dusp8	dual specificity phosphatase 8	0,63	1,54	1,24E-04	1,74E-03	NM_008748	NM_008748	NM_008748	Mm.39725	18218	ENSMUST00000039926
A_55_P2055864	Dvl2	dishevelled 2, dsh homolog (Drosophila)	0,89	1,85	2,60E-05	6,43E-04	ENSMUST000	0	BC092396	0	13543	ENSMUST00000102575
A_51_P354698	Dync1h1	dynein cytoplasmic 1 heavy chain 1	3,31	9,95	1,91E-09	5,69E-06	NM_030238	NM_030238	NM_030238	Mm.181430	13424	ENSMUST00000018851
A_52_P102783	Dync2h1	dynein cytoplasmic 2 heavy chain 1	2,15	4,45	1,93E-09	5,69E-06	NM_029851	NM_029851	NM_029851	Mm.237596	110350	ENSMUST00000147193
A_51_P472481	Dync2li1	dynein cytoplasmic 2 light intermediate chain 1	0,70	1,63	3,77E-05	7,99E-04	NM_172256	NM_172256	NM_172256	Mm.246119	213575	ENSMUST00000025101
A_55_P2329825	Dyrk2	dual-specificity tyrosine-(Y)-phosphorylation regulated kinase	0,65	1,57	8,56E-05	1,36E-03	AK149371	0	AK149371	Mm.458174	69181	0
A_51_P519108	Dzank1	double zinc ribbon and ankyrin repeat domains 1	1,43	2,69	3,28E-07	6,07E-05	NM_172859	NM_172859	NM_172859	Mm.442156	241688	ENSMUST00000081982
A_55_P2002226	Dzip1	DAZ interacting protein 1	0,85	1,81	8,18E-06	3,28E-04	NM_025943	NM_025943	NM_025943	Mm.87456	66573	ENSMUST00000047208
A_65_P08998	Dzip3	DAZ interacting protein 3, zinc finger	0,78	1,72	7,84E-05	1,28E-03	NM_00111001	NM_00111001	NM_00111001	Mm.275138	224170	ENSMUST00000114516
A_55_P2065513	Ebf1	early B cell factor 1	0,50	1,41	6,38E-05	1,13E-03	NM_007897	NM_007897	NM_007897	Mm.439662	13591	ENSMUST00000101326
A_52_P522754	Ebna1bp2	EBNA1 binding protein 2	0,66	1,58	4,47E-05	8,98E-04	NM_026932	NM_026932	NM_026932	Mm.29906	69072	ENSMUST00000030501
A_52_P472799	Ecd	ecdysoneless homolog (Drosophila)	1,09	2,12	1,58E-05	4,79E-04	NM_027475	NM_027475	NM_027475	Mm.35105	70601	ENSMUST00000022344
A_52_P325527	Edil3	EGF-like repeats and discoidin I-like domains 3	0,81	1,76	4,94E-06	2,40E-04	NM_010103	NM_010103	NM_010103	Mm.125580	13612	ENSMUST00000081769
A_52_P156190	Ednra	endothelin receptor type A	0,93	1,91	3,04E-05	7,05E-04	NM_010332	NM_010332	NM_010332	Mm.283168	13617	ENSMUST00000034029
A_55_P2089710	Ednrb	endothelin receptor type B	1,77	3,42	2,46E-07	5,22E-05	NM_007904	NM_007904	NM_007904	Mm.229532	13618	ENSMUST00000172237
A_66_P106191	Eef2	eukaryotic translation elongation factor 2	1,26	2,40	2,01E-07	4,69E-05	NM_007907	NM_007907	NM_007907	Mm.326799	13629	ENSMUST00000047864

A_55_P1972530	***Eftud1***	elongation factor Tu GTP binding domain containing 1	0,92	1,89	2,96E-05	6,91E-04	NM_175317	NM_175317	NM_175317	Mm.238020	101592	ENSMUST00000039881
A_55_P1956863	***Egfr***	epidermal growth factor receptor	1,89	3,72	3,62E-07	6,24E-05	NM_007912	NM_007912	NM_007912	Mm.8534	13649	ENSMUST00000125984
A_55_P2017845	***Ehbp1***	EH domain binding protein 1	1,09	2,13	3,36E-05	7,45E-04	NM_00125251	NM_00125251	NM_00125251	Mm.281732	216565	ENSMUST00000134293
A_51_P462918	***Ehhadh***	enoyl-Coenzyme A, hydratase/3-hydroxyacyl Coenzyme A del	0,78	1,71	4,54E-05	9,07E-04	NM_023737	NM_023737	NM_023737	Mm.28100	74147	ENSMUST00000023559
A_52_P620793	***Eif2a***	eukaryotic translation initiation factor 2A	0,88	1,85	9,83E-05	1,50E-03	NM_0010055(	NM_0010055(	NM_0010055(	Mm.21617	229317	ENSMUST00000135876
A_55_P2073945	***Eif2b3***	eukaryotic translation initiation factor 2B, subunit 3	1,06	2,09	7,80E-06	3,17E-04	NM_00111127	NM_00111127	NM_00111127	Mm.34612	108067	ENSMUST00000070610
A_51_P305532	***Eif2s3x***	eukaryotic translation initiation factor 2, subunit 3, structural g	0,77	1,71	2,26E-05	5,91E-04	NM_012010	NM_012010	NM_012010	Mm.218851	26905	ENSMUST00000050328
A_52_P524895	***Eif3a***	eukaryotic translation initiation factor 3, subunit A	1,90	3,73	3,57E-07	6,22E-05	NM_010123	NM_010123	NM_010123	Mm.2238	13669	ENSMUST00000025955
A_52_P455089	***Eif3c***	eukaryotic translation initiation factor 3, subunit C	0,73	1,66	8,82E-05	1,39E-03	ENSMUST000	0	0	0	56347	ENSMUST00000032992
A_51_P341130	***Eif3k***	eukaryotic translation initiation factor 3, subunit K	0,95	1,93	5,31E-06	2,52E-04	NM_028659	NM_028659	NM_028659	Mm.29714	73830	ENSMUST00000066070
A_66_P103926	***Eif3m***	eukaryotic translation initiation factor 3, subunit M	1,04	2,06	1,05E-05	3,77E-04	NM_145380	NM_145380	NM_145380	Mm.379278	98221	ENSMUST00000111110
A_55_P2046378	***Eif4g1***	eukaryotic translation initiation factor 4, gamma 1	1,11	2,16	2,21E-05	5,85E-04	NM_145941	NM_145941	NM_145941	Mm.260256	208643	ENSMUST00000115460
A_55_P2034372	***Eif4g3***	eukaryotic translation initiation factor 4 gamma, 3	1,17	2,25	7,45E-06	3,10E-04	NM_00125619	NM_00125619	NM_00125619	Mm.268903	230861	ENSMUST00000084215
A_55_P2181743	***Elmod1***	ELMO/CED-12 domain containing 1	1,09	2,13	2,34E-05	6,03E-04	NM_177769	NM_177769	NM_177769	Mm.259791	270162	ENSMUST00000048409
A_55_P1997407	***Elmsan1***	ELM2 and Myb/SANT-like domain containing 1	0,92	1,90	2,44E-06	1,58E-04	NM_00116350	NM_00116350	NM_00116350	Mm.31256	238317	ENSMUST00000046266
A_52_P609972	***Eln***	elastin	0,94	1,91	4,60E-05	9,14E-04	NM_007925	NM_007925	NM_007925	Mm.275320	13717	0
A_51_P463440	***Elovl6***	ELOVL family member 6, elongation of long chain fatty acids (	0,71	1,64	9,14E-05	1,42E-03	NM_130450	NM_130450	NM_130450	Mm.314113	170439	ENSMUST00000071402
A_55_P2073647	***Elp2***	elongator acetyltransferase complex subunit 2	0,69	1,61	3,84E-05	8,10E-04	NM_021448	NM_021448	NM_021448	Mm.25298	58523	ENSMUST00000025120
A_55_P2036285	***Elp4***	elongation protein 4 homolog (S. cerevisiae)	0,93	1,91	5,84E-05	1,07E-03	NM_023876	NM_023876	NM_023876	Mm.33870	77766	ENSMUST00000028588
A_52_P306537	***Emc1***	ER membrane protein complex subunit 1	0,96	1,95	8,71E-05	1,38E-03	NM_146157	NM_146157	NM_146157	Mm.394288	230866	ENSMUST00000082262
A_55_P2113673	***Eml1***	echinoderm microtubule associated protein like 1	0,56	1,47	1,18E-04	1,69E-03	NM_00104333	NM_00104333	NM_00104333	Mm.236645	68519	ENSMUST00000155544
A_51_P392776	***Eml6***	echinoderm microtubule associated protein like 6	0,84	1,79	6,76E-06	2,93E-04	NM_146016	NM_146016	NM_146016	Mm.445271	237711	ENSMUST00000058902
A_55_P2144386	***Emr1***	EGF-like module containing, mucin-like, hormone receptor-like	0,96	1,94	8,00E-05	1,30E-03	NM_010130	NM_010130	NM_010130	Mm.2254	13733	ENSMUST00000086763
A_51_P126437	***Enc1***	ectodermal-neural cortex 1	1,23	2,35	7,82E-06	3,17E-04	NM_007930	NM_007930	NM_007930	Mm.241073	13803	ENSMUST00000041623
A_55_P2055597	***Enpp2***	ectonucleotide pyrophosphatase/phosphodiesterase 2	1,09	2,13	2,42E-06	1,58E-04	NM_00113607	NM_00113607	NM_00113607	Mm.250256	18606	ENSMUST00000167541
A_52_P411601	***Ep300***	E1A binding protein p300	1,23	2,35	1,02E-06	1,01E-04	NM_177821	NM_177821	NM_177821	Mm.258397	328572	ENSMUST00000068387
A_55_P1987449	***Ep400***	E1A binding protein p400	1,46	2,75	7,22E-06	3,03E-04	NM_029337	NM_029337	NM_029337	Mm.270487	75560	ENSMUST00000112436
A_51_P250058	***Epas1***	endothelial PAS domain protein 1	1,30	2,47	7,18E-07	8,68E-05	NM_010137	NM_010137	NM_010137	Mm.1415	13819	ENSMUST00000024954
A_55_P2023762	***Epha4***	Eph receptor A4	1,32	2,50	1,62E-07	4,34E-05	NM_007936	NM_007936	NM_007936	Mm.400747	13838	ENSMUST00000027451
A_55_P2100425	***Epha5***	Eph receptor A5	0,58	1,50	9,88E-05	1,51E-03	NM_007937	NM_007937	NM_007937	Mm.137991	13839	0
A_66_P132657	***Epha6***	Eph receptor A6	1,13	2,19	3,07E-05	7,09E-04	NM_007938	NM_007938	NM_007938	Mm.455790	13840	ENSMUST00000068860
A_52_P306236	***Epha7***	Eph receptor A7	0,86	1,82	6,34E-05	1,12E-03	NM_00112288	NM_00112288	NM_00112288	Mm.257266	13841	ENSMUST00000080934
A_66_P130777	***Ephb4***	Eph receptor B4	0,99	1,98	8,29E-05	1,33E-03	NM_00115957	NM_00115957	NM_00115957	Mm.34533	13846	ENSMUST00000144296
A_55_P2124676	***Epm2aip1***	EPM2A (laforin) interacting protein 1	1,01	2,01	4,58E-06	2,30E-04	NM_175266	NM_175266	NM_175266	Mm.209005	77781	ENSMUST00000060711
A_55_P2007640	***Eprs***	glutamyl-prolyl-tRNA synthetase	1,56	2,96	1,32E-06	1,14E-04	NM_029735	NM_029735	NM_029735	Mm.154511	107508	ENSMUST00000046514
A_55_P1982454	***Eps8***	epidermal growth factor receptor pathway substrate 8	1,15	2,22	1,72E-06	1,30E-04	NM_007945	NM_007945	NM_007945	Mm.235346	13860	ENSMUST00000100841
A_51_P104891	***Ept1***	ethanolaminephosphotransferase 1 (CDP-ethanolamine-spec	1,31	2,48	9,80E-07	9,96E-05	NM_027652	NM_027652	NM_027652	Mm.168854	28042	0
A_52_P598912	***Erc1***	ELKS/RAB6-interacting/CAST family member 1	1,46	2,75	3,88E-06	2,10E-04	NM_178085	NM_178085	NM_178085	Mm.288860	111173	ENSMUST00000079582
A_55_P2026530	***Erc2***	ELKS/RAB6-interacting/CAST family member 2	1,65	3,14	5,60E-07	7,79E-05	NM_177814	NM_177814	NM_177814	Mm.318004	238988	ENSMUST00000090302
A_52_P26953	***Ergic2***	ERGIC and golgi 2	0,75	1,68	1,46E-04	1,94E-03	NM_026355	NM_026355	NM_026355	Mm.59812	67456	ENSMUST00000140311
A_52_P354202	***Eri2***	exoribonuclease 2	0,70	1,63	9,11E-05	1,42E-03	NM_027698	NM_027698	NM_027698	Mm.274160	71151	ENSMUST00000133926
A_52_P508920	***Erlec1***	endoplasmic reticulum lectin 1	0,72	1,65	8,71E-05	1,38E-03	NM_025745	NM_025745	NM_025745	Mm.294641	66753	0
A_52_P618745	***Ermp1***	endoplasmic reticulum metallopeptidase 1	1,51	2,84	2,53E-05	6,31E-04	NM_00108121	NM_00108121	NM_00108121	Mm.267131	226090	ENSMUST00000054083
A_55_P2016105	***Errfi1***	ERBB receptor feedback inhibitor 1	0,74	1,67	1,07E-04	1,58E-03	NM_133753	NM_133753	NM_133753	Mm.318841	74155	ENSMUST00000073600
A_55_P2101806	***Esf1***	ESF1, nucleolar pre-rRNA processing protein, homolog (S. ce	1,00	2,00	5,25E-05	9,95E-04	NM_00108109	NM_00108109	NM_00108109	Mm.21228	66580	ENSMUST00000046030
A_65_P19089	***Esrrg***	estrogen-related receptor gamma	0,94	1,92	9,52E-05	1,47E-03	NM_00124379	NM_00124379	NM_00124379	Mm.89989	26381	ENSMUST00000027906
A_52_P658122	***Ets2***	E26 avian leukemia oncogene 2, 3' domain	0,74	1,67	1,69E-05	4,96E-04	NM_011809	NM_011809	NM_011809	Mm.290207	23872	ENSMUST00000023612
A_55_P1971498	***Exoc1***	exocyst complex component 1	1,03	2,04	2,57E-06	1,63E-04	NM_027270	NM_027270	NM_027270	Mm.403233	69940	ENSMUST00000087133
A_55_P2037101	***Exoc2***	exocyst complex component 2	0,97	1,96	6,90E-06	2,95E-04	NM_025588	NM_025588	NM_025588	Mm.293510	66482	ENSMUST00000102946
A_52_P44030	***Exoc3***	exocyst complex component 3	1,03	2,05	1,50E-05	4,65E-04	NM_177333	NM_177333	NM_177333	Mm.489612	211446	ENSMUST00000035934
A_55_P2087518	***Exoc8***	exocyst complex component 8	0,53	1,44	1,27E-04	1,78E-03	NM_198103	NM_198103	NM_198103	Mm.347360	102058	ENSMUST00000098312
A_51_P391825	***Exosc10***	exosome component 10	0,61	1,53	2,02E-05	5,54E-04	NM_016699	NM_016699	NM_016699	Mm.266635	50912	ENSMUST00000173154
A_52_P496260	***Exosc8***	exosome component 8	0,73	1,66	1,90E-05	5,34E-04	NM_027148	NM_027148	NM_027148	Mm.29253	69639	ENSMUST00000134892
A_55_P2186125	***F13a1***	coagulation factor XIII, A1 subunit	0,73	1,66	6,18E-05	1,10E-03	NM_028784	NM_028784	NM_028784	Mm.235105	74145	ENSMUST00000037491
A_52_P678183	***Faf1***	Fas-associated factor 1	0,76	1,70	6,64E-05	1,15E-03	NM_007983	NM_007983	NM_007983	Mm.318259	14084	ENSMUST00000102724
A_51_P255193	***Faf2***	Fas associated factor family member 2	0,70	1,62	1,48E-04	1,95E-03	NM_178397	NM_178397	NM_178397	Mm.194459	76577	ENSMUST00000126071
A_51_P157982	***Fam115a***	family with sequence similarity 115, member A	1,56	2,94	2,35E-05	6,03E-04	NM_029930	NM_029930	NM_029930	Mm.24652	77574	ENSMUST00000121083
A_55_P2079142	***Fam124b***	family with sequence similarity 124, member B	1,13	2,19	1,15E-05	3,97E-04	NM_173425	NM_173425	NM_173425	Mm.145511	241128	ENSMUST00000058748
A_55_P2083489	***Fam129a***	family with sequence similarity 129, member A	0,84	1,80	3,52E-06	1,98E-04	NM_022018	NM_022018	NM_022018	Mm.482559	63913	ENSMUST00000097541
A_51_P428578	***Fam134b***	family with sequence similarity 134, member B	0,86	1,82	1,16E-04	1,67E-03	NM_025459	NM_025459	NM_025459	Mm.25311	66270	ENSMUST00000110438
A_51_P329966	***Fam13a***	family with sequence similarity 13, member A	0,89	1,85	2,35E-05	6,03E-04	NM_153574	NM_153574	NM_153574	Mm.28813	58909	ENSMUST00000089860
A_52_P561377	***Fam160b1***	family with sequence similarity 160, member B1	0,69	1,61	1,18E-04	1,69E-03	NM_145505	NM_145505	NM_145505	Mm.358870	226252	ENSMUST00000036407
A_51_P103594	***Fam162b***	family with sequence similarity 162, member B	0,62	1,54	1,07E-04	1,58E-03	NM_029894	NM_029894	NM_029894	Mm.30588	77296	ENSMUST00000020064
A_55_P2067091	***Fam179b***	family with sequence similarity 179, member B	0,73	1,66	3,24E-05	7,30E-04	NM_177805	NM_177805	NM_177805	Mm.138465	328108	ENSMUST00000066296
A_52_P572473	***Fam184b***	family with sequence similarity 184, member B	1,34	2,53	1,27E-07	3,94E-05	NM_021416	NM_021416	NM_021416	Mm.266466	58227	ENSMUST00000016023
A_55_P1953931	***Fam193a***	family with sequence similarity 193, member A	1,03	2,04	4,10E-05	8,45E-04	NM_00124312	NM_00124312	NM_00124312	Mm.286885	231128	ENSMUST00000180376
A_52_P592101	***Fam21***	family with sequence similarity 21	1,34	2,53	1,52E-06	1,22E-04	NM_026585	NM_026585	NM_026585	Mm.28524	28006	ENSMUST00000036759
A_52_P29953	***Fam212b***	family with sequence similarity 212, member B	0,47	1,38	8,25E-05	1,33E-03	NM_175398	NM_175398	NM_175398	Mm.212591	109050	ENSMUST00000066610
A_55_P2029846	***Fam214a***	family with sequence similarity 214, member A	0,61	1,53	8,22E-05	1,32E-03	NM_00111328	NM_00111328	NM_00111328	Mm.208955	235493	ENSMUST00000170846
A_55_P2150343	***Fam38a***	family with sequence similarity 38, member A	0,69	1,62	3,40E-05	7,52E-04	NM_00103729	NM_00103729	NM_00103729	Mm.490358	234839	ENSMUST00000123671
A_51_P447976	***Fam46c***	family with sequence similarity 46, member C	1,31	2,47	1,73E-07	4,42E-05	NM_00114295	NM_00114295	NM_00114295	Mm.226900	74645	ENSMUST00000061455
A_51_P253883	***Fam49a***	family with sequence similarity 49, member A	0,76	1,70	6,49E-05	1,14E-03	NM_029758	NM_029758	NM_029758	Mm.440802	76820	ENSMUST00000069066
A_55_P2144526	***Fam65b***	family with sequence similarity 65, member B	1,30	2,46	4,75E-06	2,35E-04	NM_00108038	NM_00108038	NM_00108038	Mm.217319	193385	ENSMUST00000134370
A_52_P243516	***Fam82a1***	family with sequence similarity 82, member A1	0,88	1,84	2,16E-05	5,79E-04	NM_201361	NM_201361	NM_201361	Mm.293063	381110	ENSMUST00000040368
A_55_P2071426	***Fam86***	family with sequence similarity 86	0,69	1,62	6,87E-06	2,94E-04	NM_027446	NM_027446	NM_027446	Mm.45780	70511	ENSMUST00000064635
A_55_P2044297	***Fam96a***	family with sequence similarity 96, member A	0,59	1,51	1,40E-04	1,89E-03	NM_026635	NM_026635	NM_026635	Mm.27107	68250	ENSMUST00000034945
A_51_P353173	***Fam98a***	family with sequence similarity 98, member A	0,60	1,52	1,11E-04	1,62E-03	NM_133747	NM_133747	NM_133747	Mm.490372	72722	ENSMUST00000112507
A_55_P2025790	***Fanci***	Fanconi anemia, complementation group I	0,68	1,61	8,10E-05	1,31E-03	NM_145946	NM_145946	NM_145946	Mm.349493	208836	ENSMUST00000036865
A_66_P109175	***Farsb***	phenylalanyl-tRNA synthetase, beta subunit	0,69	1,61	5,44E-05	1,02E-03	NM_011811	NM_011811	NM_011811	Mm.28922	23874	ENSMUST00000170217
A_52_P100252	***Fasn***	fatty acid synthase	1,13	2,18	6,48E-06	2,85E-04	NM_007988	NM_007988	NM_007988	Mm.236443	14104	ENSMUST00000055655
A_55_P2107502	***Fat1***	FAT tumor suppressor homolog 1 (Drosophila)	1,33	2,52	3,75E-07	6,40E-05	NM_0010812(	NM_0010812(	NM_0010812(	Mm.27365	14107	ENSMUST00000098796
A_52_P362828	***Fat3***	FAT tumor suppressor homolog 3 (Drosophila)	1,26	2,39	9,15E-06	3,50E-04	NM_00108081	NM_00108081	NM_00108081	Mm.195010	270120	ENSMUST00000082170
A_52_P675530	***Fat4***	FAT tumor suppressor homolog 4 (Drosophila)	1,17	2,25	7,46E-05	1,24E-03	NM_183221	NM_183221	NM_183221	Mm.316210	329628	ENSMUST00000061260
A_55_P2088601	***Fbln1***	fibulin 1	0,79	1,73	2,80E-05	6,71E-04	NM_010180	NM_010180	NM_010180	Mm.297992	14114	ENSMUST00000057410
A_52_P590535	***Fbln2***	fibulin 2	1,02	2,03	7,65E-06	3,13E-04	NM_007992	NM_007992	NM_007992	Mm.249146	14115	ENSMUST00000137029
A_51_P467224	***Fbn1***	fibrillin 1	1,44	2,71	1,54E-06	1,23E-04	NM_007993	NM_007993	NM_007993	Mm.271644	14118	ENSMUST00000103234
A_51_P466685	***Fbxl22***	F-box and leucine-rich repeat protein 22	1,71	3,27	1,35E-07	4,05E-05	NM_175206	NM_175206	NM_175206	Mm.297998	74165	ENSMUST00000056890
A_51_P429276	***Fbxl6***	F-box and leucine-rich repeat protein 6	0,52	1,43	1,42E-04	1,91E-03	NM_013909	NM_013909	NM_013909	Mm.275279	30840	ENSMUST00000023219
A_52_P412529	***Fbxo3***	F-box protein 3	0,90	1,87	5,67E-05	1,04E-03	NM_020593	NM_020593	NM_020593	Mm.143768	57443	ENSMUST00000102565
A_66_P123804	***Fbxo30***	F-box protein 30	0,77	1,70	1,00E-04	1,52E-03	NM_027968	NM_027968	NM_027968	Mm.276229	71865	ENSMUST00000070300
A_55_P1991406	***Fbxo34***	F-box protein 34	3,88	14,68	1,65E-08	1,39E-05	NM_00114608	NM_00114608	NM_00114608	Mm.11935	78938	ENSMUST00000095941
A_51_P362881	***Fbxo38***	F-box protein 38	0,68	1,60	6,62E-05	1,15E-03	NM_134136	NM_134136	NM_134136	Mm.21295	107035	ENSMUST00000048688
A_55_P2031966	***Fbxo39***	F-box protein 39	0,47	1,38	1,29E-04	1,80E-03	NM_0010996(	NM_0010996(	NM_0010996(	Mm.390365	628100	ENSMUST00000108504
A_51_P357877	***Fbxo46***	F-box protein 46	1,76	3,39	1,44E-07	4,10E-05	NM_175530	NM_175530	NM_175530	Mm.230830	243867	ENSMUST00000053109
A_55_P1961894	***Fbxw11***	F-box and WD-40 domain protein 11	0,77	1,71	1,35E-04	1,85E-03	NM_134015	NM_134015	NM_134015	Mm.28017	103583	ENSMUST00000109366
A_55_P2066399	***Fcho2***	FCH domain only 2	0,66	1,58	1,32E-04	1,82E-03	NM_172591	NM_172591	NM_172591	Mm.23928	218503	ENSMUST00000109403
A_52_P231075	***Fcrls***	Fc receptor-like S, scavenger receptor	1,04	2,06	2,87E-05	6,79E-04	NM_030707	NM_030707	NM_030707	Mm.45173	80891	ENSMUST00000090986
A_55_P1966209	***Fez2***	fasciculation and elongation protein zeta 2 (zygin II)	0,73	1,66	1,86E-05	5,27E-04	NM_199448	NM_199448	NM_199448	Mm.236835	225020	ENSMUST00000112487
A_52_P639229	***Fgd4***	FYVE, RhoGEF and PH domain containing 4	1,77	3,40	7,99E-07	9,17E-05	NM_139232	NM_139232	NM_139232	Mm.256131	224014	ENSMUST00000161861
A_55_P1952638	***Fgd6***	FYVE, RhoGEF and PH domain containing 6	0,54	1,45	6,32E-05	1,12E-03	NM_053072	NM_053072	NM_053072	Mm.269596	13998	ENSMUST00000020208
A_51_P359333	***Fh1***	fumarate hydratase 1	1,28	2,42	3,65E-05	7,84E-04	BC006048	0	BC006048	Mm.41502	14194	0
A_55_P1985693	***Fhdc1***	FH2 domain containing 1	0,70	1,63	6,98E-05	1,19E-03	NM_00120535	NM_00120535	NM_00120535	Mm.331107	229474	ENSMUST00000091002
A_55_P2088018	***Fhod3***	formin homology 2 domain containing 3	1,12	2,18	5,88E-06	2,70E-04	NM_175276	NM_175276	NM_175276	Mm.329322	225288	ENSMUST00000037097
A_52_P172441	***Fitm2***	fat storage-inducing transmembrane protein 2	0,62	1,53	8,22E-05	1,32E-03	NM_173397	NM_173397	NM_173397	Mm.242917	228859	ENSMUST00000109418
A_52_P156578	***Fkbp15***	FK506 binding protein 15	1,12	2,17	3,32E-05	7,41E-04	NM_00104552	NM_00104552	NM_00104552	Mm.357792	338355	ENSMUST00000107461
A_55_P1967648	***Flcn***	folliculin	0,60	1,51	1,06E-04	1,57E-03	NM_146018	NM_146018	NM_146018	Mm.339640	216805	ENSMUST00000091246
A_55_P2019068	***Flii***	flightless I homolog (Drosophila)	1,18	2,26	7,69E-07	8,95E-05	NM_022009	NM_022009	NM_022009	Mm.339755	14248	ENSMUST00000002889
A_55_P2001920	***Flna***	filamin, alpha	1,28	2,42	5,58E-06	2,61E-04	NM_010227	NM_010227	NM_010227	Mm.295533	192176	ENSMUST00000101454
A_65_P05152	***Flrt2***	fibronectin leucine rich transmembrane protein 2	0,83	1,78	1,02E-04	1,54E-03	NM_201518	NM_201518	NM_201518	Mm.341948	399558	ENSMUST00000057324
A_55_P2144556	***Flrt3***	fibronectin leucine rich transmembrane protein 3	0,91	1,88	2,80E-06	1,73E-04	NM_00117216	NM_00117216	NM_00117216	Mm.426781	71436	ENSMUST00000110057

A_51_P398723	*Flt1*	FMS-like tyrosine kinase 1	1,10	2,15	1,05E-05	3,79E-04	NM_010228	NM_010228	NM_010228	Mm.389712	14254	ENSMUST00000031653
A_55_P2005426	*Fmn2*	formin 2	1,67	3,18	9,28E-07	9,75E-05	NM_019445	NM_019445	NM_019445	Mm.330620	54418	ENSMUST00000030039
A_55_P2153783	*Fmo1*	flavin containing monooxygenase 1	0,91	1,88	4,97E-05	9,60E-04	NM_010231	NM_010231	NM_010231	Mm.976	14261	ENSMUST00000131058
A_55_P2183110	*Fmr1*	fragile X mental retardation syndrome 1	0,92	1,90	3,83E-05	8,09E-04	NM_008031	NM_008031	NM_008031	Mm.3451	14265	ENSMUST00000142584
A_55_P2130178	*Fn1*	fibronectin 1	2,52	5,73	1,06E-08	1,18E-05	NM_010233	NM_010233	NM_010233	Mm.193099	14268	ENSMUST00000055226
A_55_P1989519	*Fndc1*	fibronectin type III domain containing 1	1,31	2,48	1,44E-04	1,93E-03	NM_00108141	NM_00108141	NM_00108141	Mm.379161	68655	ENSMUST00000097425
A_52_P33800	*Fndc3a*	fibronectin type III domain containing 3A	0,49	1,40	1,29E-04	1,79E-03	NM_207636	NM_207636	NM_207636	Mm.205421	319448	ENSMUST00000089017
A_55_P2091836	*Fnip1*	folliculin interacting protein 1	0,93	1,91	2,12E-05	5,71E-04	NM_173753	NM_173753	NM_173753	Mm.34087	216742	ENSMUST00000046835
A_52_P235479	*Fntb*	farnesyltransferase, CAAX box, beta	0,72	1,65	9,08E-05	1,42E-03	NM_145927	NM_145927	NM_145927	Mm.151174	110606	ENSMUST00000041008
A_55_P2081349	*Focad*	focadhesin	0,65	1,57	1,20E-04	1,71E-03	NM_00108118	NM_00108118	NM_00108118	Mm.287210	230393	ENSMUST00000097992
A_51_P229886	*Fpgt*	fucose-1-phosphate guanylyltransferase	0,71	1,64	1,26E-04	1,76E-03	NM_029330	NM_029330	NM_029330	Mm.35218	75540	ENSMUST00000066568
A_51_P383270	*Fras1*	Fraser syndrome 1 homolog (human)	1,08	2,12	1,14E-06	1,06E-04	NM_175473	NM_175473	NM_175473	Mm.291120	231470	ENSMUST00000036019
A_55_P2097869	*Frem2*	Fras1 related extracellular matrix protein 2	0,83	1,78	4,20E-05	8,60E-04	NM_172862	NM_172862	NM_172862	Mm.38378	242022	ENSMUST00000091137
A_55_P2030383	*Frmd4a*	FERM domain containing 4A	0,83	1,78	1,41E-04	1,90E-03	NM_00117784	NM_00117784	NM_00117784	Mm.37932	209630	ENSMUST00000075767
A_51_P463401	*Fry*	furry homolog (Drosophila)	1,90	3,74	6,24E-06	2,80E-04	NM_172887	NM_172887	NM_172887	Mm.216590	320365	ENSMUST00000087204
A_55_P2289819	*Fryl*	furry homolog-like (Drosophila)	0,88	1,84	9,94E-05	1,51E-03	NM_028194	NM_028194	NM_028194	Mm.490279	72313	ENSMUST00000094700
A_52_P568235	*Fstl5*	follistatin-like 5	1,11	2,15	5,83E-07	7,93E-05	NM_178673	NM_178673	NM_178673	Mm.490527	213262	ENSMUST00000038364
A_55_P2043837	*Ftsjd2*	FtsJ methyltransferase domain containing 2	0,76	1,70	4,33E-05	8,79E-04	NM_028791	NM_028791	NM_028791	Mm.475060	74157	ENSMUST00000130871
A_52_P513842	*Fubp1*	far upstream element (FUSE) binding protein 1	0,77	1,70	1,24E-04	1,74E-03	NM_057172	NM_057172	NM_057172	Mm.278922	51886	ENSMUST00000106121
A_55_P2017183	*Fuk*	fucokinase	0,78	1,72	2,86E-05	6,77E-04	NM_172283	NM_172283	NM_172283	Mm.102591	234730	ENSMUST00000041382
A_66_P124755	*Fut8*	fucosyltransferase 8	0,55	1,46	1,45E-04	1,93E-03	NM_00125261	NM_00125261	NM_00125261	Mm.35628	53618	ENSMUST00000177595
A_51_P421223	*Fv1*	Friend virus susceptibility 1	0,58	1,50	1,28E-04	1,78E-03	NM_010244	NM_010244	NM_010244	Mm.422607	14349	ENSMUST00000094481
A_55_P2069415	*Fxr1*	fragile X mental retardation gene 1, autosomal homolog	0,92	1,89	2,70E-05	6,56E-04	NM_008053	NM_008053	NM_008053	Mm.259021	14359	ENSMUST00000167354
A_51_P142107	*Fzd7*	frizzled homolog 7 (Drosophila)	0,86	1,81	2,37E-05	6,05E-04	NM_008057	NM_008057	NM_008057	Mm.297906	14369	ENSMUST00000114246
A_55_P1963309	*G3bp1*	Ras-GTPase-activating protein SH3-domain binding protein 1	0,88	1,84	6,86E-06	2,94E-04	NM_013716	NM_013716	NM_013716	Mm.380129	27041	ENSMUST00000018727
A_52_P556281	*G3bp2*	GTPase activating protein (SH3 domain) binding protein 2	1,08	2,12	1,32E-05	4,30E-04	NM_00108079	NM_00108079	NM_00108079	Mm.290530	23881	ENSMUST00000113127
A_55_P2024439	*Gaa*	glucosidase, alpha, acid	1,41	2,65	8,25E-06	3,29E-04	NM_008064	NM_008064	NM_008064	Mm.4793	14387	ENSMUST00000128753
A_55_P2082135	*Gabbr1*	gamma-aminobutyric acid (GABA) B receptor, 1	0,91	1,88	6,22E-05	1,11E-03	NM_019439	NM_019439	NM_019439	Mm.32191	54393	ENSMUST00000173823
A_66_P118170	*Gabrg1*	gamma-aminobutyric acid (GABA) A receptor, subunit gamma	0,98	1,98	1,13E-04	1,64E-03	NM_010252	NM_010252	NM_010252	Mm.255292	14405	ENSMUST00000031119
A_55_P1965694	*Gabrg2*	gamma-aminobutyric acid (GABA) A receptor, subunit gamma	0,83	1,78	1,47E-04	1,95E-03	NM_008073	NM_008073	NM_008073	Mm.5309	14406	ENSMUST00000109292
A_52_P144310	*Gad1*	glutamate decarboxylase 1	1,24	2,37	3,20E-06	1,87E-04	NM_008077	NM_008077	NM_008077	Mm.272120	14415	ENSMUST00000140478
A_51_P495462	*Gad2*	glutamic acid decarboxylase 2	1,04	2,06	4,14E-06	2,17E-04	NM_008078	NM_008078	NM_008078	Mm.4784	14417	ENSMUST00000028123
A_55_P2050083	*Gak*	cyclin G associated kinase	0,75	1,68	7,41E-05	1,24E-03	NM_153569	NM_153569	NM_153569	Mm.276647	231580	ENSMUST00000145467
A_55_P2048210	*Ganab*	alpha glucosidase 2 alpha neutral subunit	0,91	1,88	6,23E-05	1,11E-03	NM_008060	NM_008060	NM_008060	Mm.3196	14376	ENSMUST00000096246
A_55_P2037425	*Gapdh*	glyceraldehyde-3-phosphate dehydrogenase	0,63	1,54	4,98E-05	9,61E-04	NM_008084	NM_008084	NM_008084	Mm.343110	14433	ENSMUST00000118875
A_51_P326393	*Gapvd1*	GTPase activating protein and VPS9 domains 1	1,40	2,65	2,03E-07	4,69E-05	NM_025709	NM_025709	NM_025709	Mm.156452	66691	ENSMUST00000113099
A_51_P436201	*Gart*	phosphoribosylglycinamide formyltransferase	1,63	3,09	2,12E-07	4,78E-05	NM_010256	NM_010256	NM_010256	Mm.4505	14450	ENSMUST00000156713
A_55_P1990483	*Gas2l1*	growth arrest-specific 2 like 1	0,93	1,90	6,40E-06	2,84E-04	NM_144560	NM_144560	NM_144560	Mm.44591	78926	ENSMUST00000037146
A_55_P2064676	*Gas2l3*	growth arrest-specific 2 like 3	1,34	2,53	1,53E-06	1,22E-04	AK171905	0	AK171905	Mm.11982	237436	0
A_55_P2377527	*Gbf1*	golgi-specific brefeldin A-resistance factor 1	1,59	3,01	1,50E-06	1,21E-04	NM_178930	NM_178930	NM_178930	Mm.271620	107338	ENSMUST00000177406
A_51_P463860	*Gbp7*	guanylate binding protein 7	0,76	1,69	3,31E-05	7,40E-04	NM_145545	NM_145545	NM_145545	Mm.45740	229900	ENSMUST00000045097
A_52_P351669	*Gcc2*	GRIP and coiled-coil domain containing 2	1,16	2,24	1,07E-05	3,81E-04	NM_027375	NM_027375	NM_027375	Mm.25531	70297	ENSMUST00000162965
A_55_P2169714	*Gcn1l1*	GCN1 general control of amino-acid synthesis 1-like 1 (yeast)	1,23	2,34	4,56E-06	2,29E-04	NM_172719	NM_172719	NM_172719	Mm.153014	231659	ENSMUST00000064454
A_55_P2154242	*Gfpt1*	glutamine fructose-6-phosphate transaminase 1	0,75	1,68	1,00E-04	1,51E-03	NM_013528	NM_013528	NM_013528	Mm.19893	14583	0
A_55_P1997465	*Ggnbp1*	gametogenetin binding protein 1	0,98	1,97	2,70E-06	1,68E-04	NM_027544	NM_027544	NM_027544	Mm.440437	70772	ENSMUST00000133257
A_55_P2061381	*Ghitm*	growth hormone inducible transmembrane protein	0,87	1,82	1,32E-04	1,82E-03	NM_00119912	NM_00119912	NM_00119912	Mm.182912	66092	ENSMUST00000042564
A_55_P2039712	*Gigyf2*	GRB10 interacting GYF protein 2	1,01	2,01	8,69E-06	3,39E-04	NM_146112	NM_146112	NM_146112	Mm.23065	227331	ENSMUST00000027475
A_52_P174915	*Gja1*	gap junction protein, alpha 1	1,33	2,52	2,76E-05	6,64E-04	NM_010288	NM_010288	NM_010288	Mm.378921	14609	ENSMUST00000068581
A_66_P105564	*Gjc2*	gap junction protein, gamma 2	1,12	2,17	7,03E-06	2,99E-04	NM_175452	NM_175452	NM_175452	Mm.40016	118454	ENSMUST00000108793
A_51_P337543	*Glce*	glucuronyl C5-epimerase	0,69	1,62	3,95E-05	8,26E-04	NM_033320	NM_033320	NM_033320	Mm.24411	93683	ENSMUST00000034785
A_51_P260548	*Glg1*	golgi apparatus protein 1	0,72	1,65	1,07E-04	1,58E-03	NM_009149	NM_009149	NM_009149	Mm.276271	20340	ENSMUST00000164283
A_52_P357829	*Gli2*	GLI-Kruppel family member GLI2	1,17	2,24	2,07E-05	5,61E-04	NM_00108112	NM_00108112	NM_00108112	Mm.273292	14633	ENSMUST00000062483
A_66_P133520	*Glmn*	glomulin, FKBP associated protein	0,77	1,71	4,75E-05	9,39E-04	NM_133248	NM_133248	NM_133248	Mm.41417	170823	ENSMUST00000124546
A_51_P445532	*Glra1*	glycine receptor, alpha 1 subunit	0,63	1,55	8,49E-05	1,36E-03	NM_020492	NM_020492	NM_020492	Mm.89320	14654	ENSMUST00000102716
A_55_P2108171	*Glud1*	glutamate dehydrogenase 1	0,78	1,71	1,51E-05	4,66E-04	NM_008133	NM_008133	NM_008133	Mm.10600	14661	ENSMUST00000022322
A_51_P133060	*Glyr1*	glyoxylate reductase 1 homolog (Arabidopsis)	0,88	1,84	2,75E-05	6,63E-04	NM_00107981	NM_00107981	NM_00107981	Mm.21652	74022	ENSMUST00000115844
A_51_P276939	*Gmip*	Gem-interacting protein	0,82	1,77	5,18E-05	9,86E-04	NM_198101	NM_198101	NM_198101	Mm.324305	78816	ENSMUST00000036074
A_51_P430327	*Gmpr2*	guanosine monophosphate reductase 2	0,99	1,98	3,09E-05	7,10E-04	NM_177992	NM_177992	NM_177992	Mm.390685	105446	ENSMUST00000002397
A_52_P47255	*Gmps*	guanine monophosphate synthetase	1,09	2,14	1,68E-05	4,94E-04	NM_00103330	NM_00103330	NM_00103330	Mm.331051	229363	ENSMUST00000029405
A_55_P2041784	*Gna13*	guanine nucleotide binding protein, alpha 13	0,78	1,71	5,05E-06	2,44E-04	NM_010303	NM_010303	NM_010303	Mm.193925	14674	ENSMUST00000020930
A_52_P304858	*Gnb1*	guanine nucleotide binding protein (G protein), beta 1	0,90	1,87	9,89E-05	1,51E-03	NM_008142	NM_008142	NM_008142	Mm.2344	14688	ENSMUST00000176637
A_55_P1964053	*Golga4*	golgi autoantigen, golgin subfamily a, 4	1,28	2,42	1,48E-07	4,18E-05	NM_018748	NM_018748	NM_018748	Mm.10409	54214	ENSMUST00000084820
A_55_P2051859	*Golgb1*	golgi autoantigen, golgin subfamily b, macrogolgin 1	1,13	2,18	3,28E-06	1,90E-04	NM_030035	NM_030035	NM_030035	Mm.244815	224139	ENSMUST00000039855
A_55_P2138041	*Gon4l*	gon-4-like (C.elegans)	0,91	1,87	1,63E-05	4,87E-04	NM_00124237	NM_00124237	NM_00124237	Mm.126870	76022	ENSMUST00000090942
A_55_P2012296	*Gopc*	golgi associated PDZ and coiled-coil motif containing	0,72	1,65	4,91E-06	2,40E-04	NM_00119927	NM_00119927	NM_00119927	Mm.390258	94221	ENSMUST00000105475
A_51_P167374	*Gpatch1*	G patch domain containing 1	0,59	1,51	1,41E-04	1,90E-03	NM_026181	NM_026181	NM_026181	Mm.358705	67471	ENSMUST00000131213
A_55_P2025403	*Gphn*	gephyrin	0,97	1,96	1,82E-05	5,20E-04	NM_145965	NM_145965	NM_145965	Mm.341742	268566	ENSMUST00000110388
A_52_P566681	*Gpm6a*	glycoprotein m6a	0,95	1,93	3,83E-06	2,08E-04	NM_153581	NM_153581	NM_153581	Mm.241700	234267	ENSMUST00000033915
A_55_P2011600	*Gpr101*	G protein-coupled receptor 101	0,81	1,76	2,25E-05	5,90E-04	NM_00103336	NM_00103336	NM_00103336	Mm.336070	245424	ENSMUST00000057645
A_52_P589763	*Gpr137c*	G protein-coupled receptor 137C	0,66	1,58	3,87E-05	8,14E-04	NM_027518	NM_027518	NM_027518	Mm.343793	70713	ENSMUST00000151749
A_51_P240703	*Gpr151*	G protein-coupled receptor 151	0,98	1,97	5,17E-06	2,47E-04	NM_181543	NM_181543	NM_181543	Mm.186779	240239	ENSMUST00000054738
A_52_P558988	*Gpr155*	G protein-coupled receptor 155	0,90	1,86	1,45E-04	1,94E-03	NM_00108070	NM_00108070	NM_00108070	Mm.465902	68526	ENSMUST00000048504
A_52_P141687	*Gpr37l1*	G protein-coupled receptor 37-like 1	1,00	2,00	1,15E-04	1,66E-03	NM_134438	NM_134438	NM_134438	Mm.272290	171469	ENSMUST00000027682
A_52_P317393	*Gpr56*	G protein-coupled receptor 56	0,65	1,57	3,17E-05	7,21E-04	NM_018882	NM_018882	NM_018882	Mm.290834	14766	ENSMUST00000179619
A_51_P309744	*Gpr89*	G protein-coupled receptor 89	1,01	2,01	6,10E-06	2,76E-04	NM_026229	NM_026229	NM_026229	Mm.46722	67549	ENSMUST00000029738
A_55_P2132902	*Gprasp1*	G protein-coupled receptor associated sorting protein 1	1,00	2,00	5,51E-06	2,59E-04	NM_026081	NM_026081	NM_026081	Mm.271980	67298	ENSMUST00000166554
A_55_P2004213	*Gprasp2*	G protein-coupled receptor associated sorting protein 2	1,15	2,22	4,88E-06	2,39E-04	NM_00116301	NM_00116301	NM_00116301	Mm.489763	245607	ENSMUST00000113136
A_51_P173709	*Gprc5b*	G protein-coupled receptor, family C, group 5, member B	0,80	1,74	1,00E-05	3,68E-04	NM_022420	NM_022420	NM_022420	Mm.103439	64297	ENSMUST00000008878
A_52_P179309	*Gria3*	glutamate receptor, ionotropic, AMPA3 (alpha 3)	1,37	2,58	2,15E-06	1,47E-04	NM_016886	NM_016886	NM_016886	Mm.327681	53623	ENSMUST00000165288
A_52_P68221	*Gria3*	glutamate receptor, ionotropic, AMPA3 (alpha 3)	0,86	1,82	3,91E-05	8,19E-04	NM_016886	NM_016886	NM_016886	Mm.327681	53623	ENSMUST00000139965
A_51_P477458	*Grid2*	glutamate receptor, ionotropic, delta 2	1,49	2,81	3,10E-07	5,98E-05	NM_008167	NM_008167	NM_008167	Mm.439651	14804	ENSMUST00000095852
A_51_P192800	*Grn*	granulin	1,17	2,26	4,85E-05	9,48E-04	NM_008175	NM_008175	NM_008175	Mm.1568	14824	ENSMUST00000049460
A_51_P172231	*Gsdmd*	gasdermin D	0,60	1,51	5,57E-05	1,03E-03	NM_026960	NM_026960	NM_026960	Mm.27385	69146	ENSMUST00000023238
A_65_P17476	*Gse1*	genetic suppressor element 1	1,16	2,23	1,90E-05	5,34E-04	NM_198671	NM_198671	NM_198671	Mm.334856	382034	ENSMUST00000118136
A_51_P514412	*Gsk3b*	glycogen synthase kinase 3 beta	0,80	1,74	8,82E-05	1,39E-03	NM_019827	NM_019827	NM_019827	Mm.394930	56637	ENSMUST00000134870
A_55_P2055638	*Gsn*	gelsolin	2,50	5,66	7,46E-09	9,49E-06	NM_146120	NM_146120	NM_146120	Mm.21109	227753	0
A_55_P2040907	*Gtf2i*	general transcription factor II I	1,40	2,65	2,51E-05	6,27E-04	NM_010365	NM_010365	NM_010365	Mm.261570	14886	ENSMUST00000173887
A_55_P2288232	*Gucy1a2*	guanylate cyclase 1, soluble, alpha 2	1,03	2,04	1,32E-06	1,14E-04	NM_00103332	NM_00103332	NM_00103332	Mm.91240	234889	0
A_55_P1992049	*Gucy1a3*	guanylate cyclase 1, soluble, alpha 3	0,67	1,60	1,22E-04	1,73E-03	NM_021896	NM_021896	NM_021896	Mm.143831	60596	ENSMUST00000048976
A_55_P2461067	*Guf1*	GUF1 GTPase homolog (S. cerevisiae)	0,90	1,87	2,45E-05	6,19E-04	NM_172711	NM_172711	NM_172711	Mm.21105	231279	ENSMUST00000087228
A_55_P2035667	*Gyk*	glycerol kinase	0,94	1,92	9,84E-05	1,50E-03	NM_008194	NM_008194	NM_008194	Mm.246682	14933	ENSMUST00000152221
A_55_P1997105	*Gyltl1b*	glycosyltransferase-like 1B	1,22	2,33	8,01E-06	3,23E-04	NM_172670	NM_172670	NM_172670	Mm.31158	228366	ENSMUST00000090582
A_55_P2026315	*Gys1*	glycogen synthase 1, muscle	0,93	1,91	2,13E-06	1,46E-04	NM_030678	NM_030678	NM_030678	Mm.275654	14936	ENSMUST00000003964
A_55_P2049647	*H2-K1*	histocompatibility 2, K1, K region	0,64	1,56	6,10E-05	1,09E-03	NM_00100189	NM_00100189	NM_00100189	Mm.422886	14972	ENSMUST00000173317
A_55_P2003996	*H2-T24*	histocompatibility 2, T region locus 24	0,70	1,63	5,67E-05	1,04E-03	NM_008207	NM_008207	NM_008207	Mm.439654	15042	ENSMUST00000113760
A_55_P2058726	*Hap1*	huntingtin-associated protein 1	1,00	2,00	4,19E-05	8,59E-04	NM_010404	NM_010404	NM_010404	Mm.281700	15114	ENSMUST00000103124
A_55_P2020876	*Haus6*	HAUS augmin-like complex, subunit 6	0,81	1,76	1,07E-04	1,58E-03	NM_173400	NM_173400	NM_173400	Mm.74730	230376	ENSMUST00000128381
A_55_P2083093	*Haus7*	HAUS augmin-like complex, subunit 7	0,77	1,70	1,02E-04	1,53E-03	NM_207104	NM_207104	NM_207104	Mm.264899	73738	ENSMUST00000077243
A_52_P39247	*Hbp1*	high mobility group box transcription factor 1	0,67	1,59	6,34E-05	1,12E-03	NM_153198	NM_153198	NM_153198	Mm.390461	73389	ENSMUST00000172314
A_55_P1965629	*Hcfc1*	host cell factor C1	1,24	2,36	1,40E-05	4,45E-04	NM_008224	NM_008224	NM_008224	Mm.248353	15161	ENSMUST00000033761
A_52_P581326	*Hcfc2*	host cell factor C2	0,72	1,64	1,51E-05	4,65E-04	NM_00108121	NM_00108121	NM_00108121	Mm.127405	67933	ENSMUST00000020478
A_51_P254656	*Hdc*	histidine decarboxylase	0,70	1,62	3,36E-05	7,45E-04	NM_008230	NM_008230	NM_008230	Mm.18603	15186	ENSMUST00000028838
A_66_P108019	*Heatr1*	HEAT repeat containing 1	1,37	2,59	1,48E-06	1,21E-04	NM_144835	NM_144835	NM_144835	Mm.171186	217995	0
A_52_P297075	*Heatr5a*	HEAT repeat containing 5A	0,76	1,69	1,37E-04	1,86E-03	NM_177171	NM_177171	NM_177171	Mm.131941	320487	ENSMUST00000040583
A_55_P1970126	*Heatr5b*	HEAT repeat containing 5B	1,77	3,40	5,02E-07	7,31E-05	NM_00108117	NM_00108117	NM_00108117	Mm.210489	320473	ENSMUST00000097281
A_51_P416191	*Hectd1*	HECT domain containing 1	2,15	4,44	8,22E-07	9,30E-05	NM_144788	NM_144788	NM_144788	Mm.249391	207304	ENSMUST00000179265
A_55_P2144716	*Hecw2*	HECT, C2 and WW domain containing E3 ubiquitin protein liga	1,01	2,02	2,18E-06	1,49E-04	NM_00100188	NM_00100188	NM_00100188	Mm.132150	329152	ENSMUST00000120904
A_51_P167426	*Herc1*	hect (homologous to the E6-AP (UBE3A) carboxyl terminus) c	2,04	4,11	7,08E-08	2,82E-05	NM_145617	NM_145617	NM_145617	Mm.244179	235439	ENSMUST00000144280
A_55_P2106429	*Herc2*	hect (homologous to the E6-AP (UBE3A) carboxyl terminus) c	1,20	2,29	6,43E-05	1,13E-03	NM_010418	NM_010418	NM_010418	Mm.20929	15204	ENSMUST00000164095
A_51_P453111	*Hexb*	hexosaminidase B	0,78	1,71	3,70E-05	7,91E-04	NM_010422	NM_010422	NM_010422	Mm.27816	15212	ENSMUST00000022169

A_51_P484998	**Hgf**	hepatocyte growth factor	0,90	1,87	2,05E-05	5,59E-04	NM_010427	NM_010427	NM_010427	Mm.267078	15234	ENSMUST00000030683
A_55_P1969276	**Hhip**	Hedgehog-interacting protein	1,17	2,25	1,19E-05	4,02E-04	NM_020259	NM_020259	NM_020259	Mm.254493	15245	ENSMUST00000079038
A_55_P2137001	**Hhipl1**	hedgehog interacting protein-like 1	1,25	2,38	5,21E-07	7,45E-05	NM_00104438	NM_00104438	NM_00104438	Mm.36423	214305	ENSMUST00000021685
A_51_P387608	**Hif1a**	hypoxia inducible factor 1, alpha subunit	1,23	2,34	3,19E-05	7,25E-04	NM_010431	NM_010431	NM_010431	Mm.3879	15251	ENSMUST00000021530
A_51_P166023	**Hip1**	huntingtin interacting protein 1	0,90	1,86	3,18E-06	1,86E-04	NM_146001	NM_146001	NM_146001	Mm.280805	215114	ENSMUST00000060311
A_51_P440460	**Hip1r**	huntingtin interacting protein 1 related	1,12	2,18	4,90E-07	7,25E-05	NM_145070	NM_145070	NM_145070	Mm.149954	29816	ENSMUST00000000939
A_55_P2091861	**Hipk2**	homeodomain interacting protein kinase 2	0,87	1,82	1,13E-04	1,64E-03	NM_00113606	NM_00113606	NM_00113606	Mm.23790	15258	ENSMUST00000114855
A_55_P2118843	**Hk1**	hexokinase 1	1,36	2,56	2,10E-05	5,69E-04	NM_00114610	NM_00114610	NM_00114610	Mm.196605	15275	ENSMUST00000072357
A_51_P204080	**Hk2**	hexokinase 2	0,57	1,49	4,37E-05	8,84E-04	NM_013820	NM_013820	NM_013820	Mm.255848	15277	ENSMUST00000168725
A_52_P532033	**Hltf**	helicase-like transcription factor	1,08	2,11	9,13E-07	9,69E-05	NM_009210	NM_009210	NM_009210	Mm.209650	20585	ENSMUST00000145853
A_52_P137371	**Hmgcr**	3-hydroxy-3-methylglutaryl-Coenzyme A reductase	1,59	3,00	2,35E-06	1,56E-04	NM_008255	NM_008255	NM_008255	Mm.316652	15357	ENSMUST00000022176
A_65_P08022	**Hnrnpk**	heterogeneous nuclear ribonucleoprotein K	1,03	2,05	2,21E-05	5,85E-04	ENSMUST000	0	AK207611	Mm.142872	15387	ENSMUST00000176359
A_51_P176387	**Hook3**	hook homolog 3 (Drosophila)	0,95	1,93	5,52E-05	1,03E-03	NM_207659	NM_207659	NM_207659	Mm.334464	320191	ENSMUST00000037182
A_52_P243599	**Hsd17b12**	hydroxysteroid (17-beta) dehydrogenase 12	1,27	2,42	5,04E-06	2,44E-04	NM_019657	NM_019657	NM_019657	Mm.489651	56348	ENSMUST00000146580
A_51_P445662	**Hsd17b4**	hydroxysteroid (17-beta) dehydrogenase 4	1,61	3,05	7,12E-08	2,82E-05	NM_008292	NM_008292	NM_008292	Mm.277857	15488	ENSMUST00000025385
A_51_P153557	**Hspa12a**	heat shock protein 12A	1,18	2,26	8,69E-05	1,38E-03	NM_175199	NM_175199	NM_175199	Mm.39739	73442	ENSMUST00000066285
A_55_P2080151	**Hspa2**	heat shock protein 2	0,99	1,99	1,12E-05	3,91E-04	NM_008301	NM_008301	NM_008301	Mm.296181	15512	ENSMUST00000080449
A_55_P2054157	**Hspa4**	heat shock protein 4	0,96	1,94	4,11E-06	2,16E-04	NM_008300	NM_008300	NM_008300	Mm.239865	15525	ENSMUST00000020630
A_55_P2173737	**Hspa4l**	heat shock protein 4 like	0,73	1,66	1,22E-04	1,73E-03	NM_011020	NM_011020	NM_011020	Mm.39330	18415	ENSMUST00000077083
A_55_P1959648	**Hspg2**	perlecan (heparan sulfate proteoglycan 2)	1,54	2,90	4,07E-06	2,15E-04	NM_008305	NM_008305	NM_008305	Mm.273662	15530	ENSMUST00000155648
A_55_P2003513	**Hsph1**	heat shock 105kDa/110kDa protein 1	1,75	3,37	6,41E-08	2,71E-05	NM_013559	NM_013559	NM_013559	Mm.270681	15505	ENSMUST00000076410
A_66_P120995	**Htr2a**	5-hydroxytryptamine (serotonin) receptor 2A	0,82	1,76	7,26E-05	1,22E-03	NM_172812	NM_172812	NM_172812	Mm.214351	15558	ENSMUST00000036653
A_55_P1977503	**Htr2c**	5-hydroxytryptamine (serotonin) receptor 2C	1,20	2,30	6,98E-07	8,61E-05	NM_008312	NM_008312	NM_008312	Mm.439670	15560	ENSMUST00000036303
A_52_P581594	**Htr4**	5 hydroxytryptamine (serotonin) receptor 4	1,10	2,15	5,95E-06	2,72E-04	NM_008313	NM_008313	NM_008313	Mm.20440	15562	ENSMUST00000027560
A_51_P322115	**Htr5b**	5-hydroxytryptamine (serotonin) receptor 5B	0,82	1,76	3,21E-05	7,27E-04	NM_010483	NM_010483	NM_010483	Mm.4833	15564	ENSMUST00000055884
A_51_P225224	**Htra1**	HtrA serine peptidase 1	0,70	1,62	3,91E-05	8,19E-04	NM_019564	NM_019564	NM_019564	Mm.30156	56213	ENSMUST00000006367
A_55_P2088530	**Htt**	huntingtin	1,10	2,15	1,96E-06	1,40E-04	NM_010414	NM_010414	NM_010414	Mm.209071	15194	ENSMUST00000080036
A_52_P200617	**Iars**	isoleucine-tRNA synthetase	1,29	2,44	2,66E-06	1,66E-04	NM_172015	NM_172015	NM_172015	Mm.21118	105148	ENSMUST00000165316
A_52_P613241	**Icam1**	intercellular adhesion molecule 1	0,70	1,63	2,02E-05	5,55E-04	NM_010493	NM_010493	NM_010493	Mm.435508	15894	ENSMUST00000086399
A_51_P132978	**Idh1**	isocitrate dehydrogenase 1 (NADP+), soluble	1,16	2,23	1,87E-05	5,28E-04	NM_010497	NM_010497	NM_010497	Mm.9925	15926	ENSMUST00000169032
A_51_P130110	**Idh3b**	isocitrate dehydrogenase 3 (NAD+) beta	0,64	1,56	1,07E-04	1,58E-03	NM_130884	NM_130884	NM_130884	Mm.29590	170718	ENSMUST00000028892
A_55_P2121856	**Ier5l**	immediate early response 5-like	0,56	1,48	1,08E-04	1,59E-03	NM_030244	NM_030244	NM_030244	Mm.295464	72500	0
A_55_P2009187	**Iffo1**	intermediate filament family orphan 1	0,65	1,57	1,35E-05	4,36E-04	NM_00103966	NM_00103966	NM_00103966	Mm.129415	320678	ENSMUST00000117675
A_51_P490305	**Ifi30**	interferon gamma inducible protein 30	0,80	1,74	2,20E-05	5,84E-04	NM_023065	NM_023065	NM_023065	Mm.30241	65972	ENSMUST00000034299
A_52_P190405	**Ifnar2**	interferon (alpha and beta) receptor 2	0,88	1,84	1,51E-04	1,98E-03	NM_010509	NM_010509	NM_010509	Mm.6834	15976	ENSMUST00000117836
A_51_P456857	**Ift122**	intraflagellar transport 122	0,76	1,69	8,98E-05	1,41E-03	NM_031177	NM_031177	NM_031177	Mm.333335	81896	ENSMUST00000038234
A_51_P330144	**Ift140**	intraflagellar transport 140	1,19	2,27	1,47E-05	4,60E-04	NM_134126	NM_134126	NM_134126	Mm.32802	106633	ENSMUST00000137386
A_52_P509906	**Ift172**	intraflagellar transport 172	1,97	3,93	1,56E-08	1,37E-05	NM_026298	NM_026298	NM_026298	Mm.293023	67661	ENSMUST00000041565
A_52_P459929	**Ift81**	intraflagellar transport 81	0,71	1,63	4,20E-05	8,59E-04	NM_009879	NM_009879	NM_009879	Mm.10125	12589	ENSMUST00000135956
A_51_P125467	**Igf2r**	insulin-like growth factor 2 receptor	1,00	2,00	1,54E-05	4,72E-04	NM_010515	NM_010515	NM_010515	Mm.26553	16004	ENSMUST00000024599
A_55_P2018611	**Igfbp7**	insulin-like growth factor binding protein 7	0,92	1,89	1,00E-05	3,68E-04	NM_00115951	NM_00115951	NM_00115951	Mm.233470	29817	ENSMUST00000163898
A_55_P1975396	**Ighmbp2**	immunoglobulin mu binding protein 2	0,67	1,59	6,71E-05	1,16E-03	NM_009212	NM_009212	NM_009212	Mm.3179	20589	ENSMUST00000025751
A_55_P2059864	**Igsf1**	immunoglobulin superfamily, member 1	1,25	2,38	1,71E-06	1,30E-04	NM_177591	NM_177591	NM_177591	Mm.110505	209268	ENSMUST00000033442
A_55_P2157902	**Igsf10**	immunoglobulin superfamily, member 10	0,97	1,96	2,11E-05	5,70E-04	NM_00116288	NM_00116288	NM_00116288	Mm.228086	242050	ENSMUST00000039419
A_51_P327632	**Igsf3**	immunoglobulin superfamily, member 3	0,65	1,57	1,14E-04	1,66E-03	NM_207205	NM_207205	NM_207205	Mm.489667	78908	ENSMUST00000043983
A_51_P231687	**Ik**	IK cytokine	0,78	1,72	2,79E-05	6,69E-04	NM_011879	NM_011879	NM_011879	Mm.30234	24010	ENSMUST00000007042
A_51_P114878	**Ikbkap**	inhibitor of kappa light polypeptide enhancer in B cells, kinase	1,02	2,02	5,49E-06	2,58E-04	NM_026079	NM_026079	NM_026079	Mm.282743	230233	ENSMUST00000150002
A_55_P1970489	**Il12rb2**	interleukin 12 receptor, beta 2	1,29	2,44	1,69E-06	1,30E-04	NM_008354	NM_008354	NM_008354	Mm.188337	16162	ENSMUST00000018485
A_55_P2035052	**Il1f10**	interleukin 1 family, member 10	0,73	1,66	6,89E-06	2,95E-04	NM_153077	NM_153077	NM_153077	Mm.218750	215274	ENSMUST00000058056
A_66_P113749	**Il2rg**	interleukin 2 receptor, gamma chain	0,69	1,62	7,91E-05	1,29E-03	NM_013563	NM_013563	NM_013563	Mm.2923	16186	ENSMUST00000033664
A_51_P473888	**Il6st**	interleukin 6 signal transducer	0,76	1,70	6,21E-05	1,11E-03	NM_010560	NM_010560	NM_010560	Mm.4364	16195	ENSMUST00000070731
A_51_P117109	**Ilvbl**	ilvB (bacterial acetolactate synthase)-like	0,96	1,95	1,59E-05	4,81E-04	NM_173751	NM_173751	NM_173751	Mm.2644	216136	ENSMUST00000105384
A_51_P458852	**Ina**	internexin neuronal intermediate filament protein, alpha	0,92	1,90	7,43E-05	1,24E-03	ENSMUST000		BU053129	Mm.414453	226180	ENSMUST00000037636
A_55_P2028054	**Incenp**	inner centromere protein	0,94	1,92	3,66E-05	7,85E-04	NM_016692	NM_016692	NM_016692	Mm.29755	16319	ENSMUST00000025562
A_51_P239750	**Inhba**	inhibin beta-A	0,86	1,82	4,30E-05	8,74E-04	NM_008380	NM_008380	NM_008380	Mm.8042	16323	ENSMUST00000042603
A_66_P104554	**Inpp1**	inositol polyphosphate-1-phosphatase	0,61	1,53	1,16E-04	1,67E-03	NM_008384	NM_008384	NM_008384	Mm.917	16329	ENSMUST00000177279
A_55_P2129826	**Inpp5f**	inositol polyphosphate-5-phosphatase F	0,70	1,62	1,09E-04	1,60E-03	NM_178641	NM_178641	NM_178641	Mm.490344	101490	ENSMUST00000129085
A_51_P439452	**Insig2**	insulin induced gene 2	1,06	2,08	9,38E-06	3,55E-04	NM_133748	NM_133748	NM_133748	Mm.27136	72999	ENSMUST00000003818
A_55_P2172566	**Insr**	insulin receptor	1,30	2,46	3,94E-07	6,55E-05	NM_010568	NM_010568	NM_010568	Mm.268003	16337	ENSMUST00000091291
A_51_P295708	**Ints1**	integrator complex subunit 1	1,31	2,48	4,78E-06	2,36E-04	NM_026748	NM_026748	NM_026748	Mm.292942	68510	ENSMUST00000072607
A_51_P284793	**Ints12**	integrator complex subunit 12	1,43	2,69	2,05E-06	1,44E-04	NM_027927	NM_027927	NM_027927	Mm.246726	71793	ENSMUST00000029650
A_55_P2254276	**Ints2**	integrator complex subunit 2	1,41	2,66	1,21E-06	1,10E-04	NM_027421	NM_027421	NM_027421	Mm.440906	70422	ENSMUST00000134883
A_51_P121252	**Ints4**	integrator complex subunit 4	1,75	3,36	5,21E-08	2,43E-05	NM_027256	NM_027256	NM_027256	Mm.27383	101861	ENSMUST00000026126
A_51_P103718	**Ip6k1**	inositol hexaphosphate kinase 1	0,65	1,57	1,37E-04	1,86E-03	NM_013785	NM_013785	NM_013785	Mm.276155	27399	ENSMUST00000035214
A_52_P588881	**Iqgap3**	IQ motif containing GTPase activating protein 3	1,42	2,68	3,13E-07	6,03E-05	NM_00103348	NM_00103348	NM_00103348	Mm.331133	404710	ENSMUST00000071812
A_55_P2052416	**Iqsec1**	IQ motif and Sec7 domain 1	0,76	1,70	1,44E-04	1,93E-03	NM_00113438	NM_00113438	NM_00113438	Mm.196943	232227	ENSMUST00000101151
A_52_P175242	**Irs1**	insulin receptor substrate 1	0,77	1,70	1,82E-05	5,21E-04	NM_010570	NM_010570	NM_010570	Mm.4952	16367	ENSMUST00000069799
A_55_P1976805	**Isca2**	iron-sulfur cluster assembly 2 homolog (S. cerevisiae)	0,83	1,78	4,81E-05	9,43E-04	NM_028863	NM_028863	NM_028863	Mm.25719	74316	ENSMUST00000021667
A_55_P2103706	**Isg15**	ISG15 ubiquitin-like modifier	0,62	1,54	4,97E-05	9,60E-04	NM_015783	NM_015783	NM_015783	Mm.4950	100038882	ENSMUST00000085425
A_55_P2013740	**Islr2**	immunoglobulin superfamily containing leucine-rich repeat 2	0,91	1,89	9,30E-05	1,44E-03	NM_00116153	NM_00116153	NM_00116153	Mm.186499	320563	ENSMUST00000163897
A_55_P2097913	**Itga10**	integrin, alpha 10	0,50	1,42	1,29E-04	1,82E-03	NM_00108105	NM_00108105	NM_00108105	Mm.32741	213119	ENSMUST00000029744
A_55_P1977926	**Itgam**	integrin alpha M	0,65	1,57	5,08E-05	9,74E-04	NM_008401	NM_008401	NM_008401	Mm.262106	16409	ENSMUST00000156593
A_51_P382484	**Itgav**	integrin alpha V	0,85	1,80	7,83E-05	1,28E-03	NM_008402	NM_008402	NM_008402	Mm.227	16410	ENSMUST00000028499
A_66_P113059	**Itgb1**	integrin beta 1 (fibronectin receptor beta)	1,26	2,40	3,22E-05	7,28E-04	NM_010578	NM_010578	NM_010578	Mm.263396	16412	ENSMUST00000124826
A_51_P262208	**Itgb2**	integrin beta 2	0,88	1,84	2,38E-05	6,07E-04	NM_008404	NM_008404	NM_008404	Mm.1137	16414	ENSMUST00000156644
A_55_P1998299	**Itgb4**	integrin beta 4	1,26	2,40	1,57E-06	8,68E-05	NM_00100560	NM_00100560	NM_00100560	Mm.213873	192897	ENSMUST00000106461
A_55_P2077048	**Itih5**	inter-alpha (globulin) inhibitor H5	0,94	1,91	5,72E-05	1,05E-03	NM_172471	NM_172471	NM_172471	Mm.313876	209378	ENSMUST00000026886
A_52_P99411	**Itm2b**	integral membrane protein 2B	0,75	1,68	2,58E-05	6,40E-04	NM_008410	NM_008410	NM_008410	Mm.4266	16432	ENSMUST00000022704
A_51_P273609	**Itpka**	inositol 1,4,5-trisphosphate 3-kinase A	2,77	6,82	6,10E-09	8,90E-06	NM_146125	NM_146125	NM_146125	Mm.65337	228550	ENSMUST00000028758
A_55_P2070069	**Itpkb**	inositol 1,4,5-trisphosphate 3-kinase B	2,13	4,38	6,53E-08	2,74E-05	NM_00108117	NM_00108117	NM_00108117	Mm.132535	320404	ENSMUST00000070181
A_55_P2008066	**Itpr1**	inositol 1,4,5-trisphosphate receptor 1	1,26	2,39	1,02E-06	1,01E-04	NM_010585	NM_010585	NM_010585	Mm.227912	16438	ENSMUST00000032192
A_55_P2008061	**Itpr2**	inositol 1,4,5-triphosphate receptor 2	1,05	2,07	5,96E-06	2,72E-04	NM_019923	NM_019923	NM_019923	Mm.7800	16439	ENSMUST00000053273
A_55_P2431118	**Itsn1**	intersectin 1 (SH3 domain protein 1A)	0,79	1,73	9,33E-06	3,54E-04	NM_00111027	NM_00111027	NM_00111027	Mm.40546	16443	ENSMUST00000138117
A_52_P634090	**Jag1**	jagged 1	0,98	1,97	4,47E-06	2,27E-04	NM_013822	NM_013822	NM_013822	Mm.22398	16449	ENSMUST00000028735
A_51_P451338	**Jag2**	jagged 2	0,87	1,83	1,03E-05	3,74E-04	NM_010588	NM_010588	NM_010588	Mm.186146	16450	ENSMUST00000075827
A_52_P106463	**Jak1**	Janus kinase 1	0,96	1,94	2,12E-05	5,70E-04	NM_146145	NM_146145	NM_146145	Mm.289657	16451	ENSMUST00000102781
A_55_P2003123	**Jkamp**	JNK1/MAPK8-associated membrane protein	0,79	1,72	2,25E-05	5,90E-04	NM_024205	NM_024205	NM_024205	Mm.258935	104771	ENSMUST00000117449
A_55_P1968043	**Jmjd1c**	jumonji domain containing 1C	0,75	1,68	7,04E-05	1,20E-03	NM_00124239	NM_00124239	NM_00124239	Mm.23846	108829	ENSMUST00000173689
A_65_P13547	**Jup**	junction plakoglobin	1,12	2,18	1,02E-06	1,01E-04	NM_010593	NM_010593	NM_010593	Mm.299774	16480	ENSMUST00000107403
A_55_P2160623	**Kalrn**	kalirin, RhoGEF kinase	1,62	3,07	3,54E-07	6,21E-05	NM_177357	NM_177357	NM_177357	Mm.82274	545156	ENSMUST00000132002
A_55_P2014304	**Kank1**	KN motif and ankyrin repeat domains 1	1,26	2,39	1,75E-07	4,42E-05	NM_181404	NM_181404	NM_181404	Mm.260722	107351	ENSMUST00000049400
A_55_P2071691	**Kank3**	KN motif and ankyrin repeat domains 3	0,57	1,48	7,06E-05	1,20E-03	NM_030697	NM_030697	NM_030697	Mm.196330	80880	ENSMUST00000048560
A_55_P2146404	**Kansl1**	KAT8 regulatory NSL complex subunit 1	0,92	1,89	3,27E-05	7,33E-04	NM_00108104	NM_00108104	NM_00108104	Mm.136418	76719	ENSMUST00000018556
A_55_P1965709	**Kbtbd3**	kelch repeat and BTB (POZ) domain containing 3	0,74	1,67	5,06E-05	9,71E-04	NM_026962	NM_026962	NM_026962	Mm.25946	69149	ENSMUST00000049648
A_55_P2123283	**Kcna6**	potassium voltage-gated channel, shaker-related, subfamily, r	0,78	1,72	1,04E-04	1,55E-03	NM_013568	NM_013568	NM_013568	Mm.62535	16494	ENSMUST00000112242
A_52_P867058	**Kcnc1**	potassium voltage gated channel, Shaw-related subfamily, me	0,63	1,54	8,02E-05	1,30E-03	NM_00111273	NM_00111273	NM_00111273	Mm.249386	16502	0
A_55_P2027083	**Kcnj10**	potassium inwardly-rectifying channel, subfamily J, member 1	0,85	1,80	4,25E-06	2,20E-04	NM_00103948	NM_00103948	NM_00103948	Mm.254563	16513	ENSMUST00000056136
A_52_P191086	**Kcnj6**	potassium inwardly-rectifying channel, subfamily J, member 6	0,98	1,98	2,31E-05	5,97E-04	NM_00102558	NM_00102558	NM_00102558	Mm.328720	16522	ENSMUST00000095873
A_52_P191086	**Kcnj6**	potassium inwardly-rectifying channel, subfamily J, member 6	0,76	1,69	5,56E-05	1,03E-03	NM_00102558	NM_00102558	NM_00102558	Mm.328720	16522	ENSMUST00000095873
A_55_P1985544	**Kcnk10**	potassium channel, subfamily K, member 10	0,70	1,63	1,78E-05	5,13E-04	NM_029911	NM_029911	NM_029911	Mm.196158	72258	ENSMUST00000110113
A_55_P1961730	**Kcnt2**	potassium channel, subfamily T, member 2	0,83	1,78	5,44E-05	1,02E-03	NM_00108102	NM_00108102	NM_00108102	Mm.483845	240776	ENSMUST00000060201
A_55_P2074499	**Kcp**	kielin/chordin-like protein	0,62	1,54	3,66E-05	7,85E-04	NM_00102998	NM_00102998	NM_00102998	Mm.332961	333088	ENSMUST00000101614
A_51_P338397	**Kctd10**	potassium channel tetramerisation domain containing 10	0,90	1,86	2,78E-05	6,68E-04	NM_026145	NM_026145	NM_026145	Mm.490304	330171	ENSMUST00000102581
A_55_P1992762	**Kdelr1**	KDEL (Lys-Asp-Glu-Leu) endoplasmic reticulum protein retent	1,50	2,83	2,99E-06	1,80E-04	NM_133950	NM_133950	NM_133950	Mm.298433	68137	ENSMUST00000002855
A_55_P1982578	**Kdm3a**	lysine (K)-specific demethylase 3A	0,91	1,87	3,21E-06	1,87E-04	NM_173001	NM_173001	NM_173001	Mm.260479	104263	ENSMUST00000167220
A_55_P1970665	**Kdm3b**	KDM3B lysine (K)-specific demethylase 3B	0,93	1,90	2,25E-05	5,90E-04	NM_00108125	NM_00108125	NM_00108125	Mm.277906	277250	ENSMUST00000043775
A_52_P281209	**Kdm4a**	lysine (K)-specific demethylase 4A	1,12	2,17	4,17E-07	6,79E-05	NM_00116182	NM_00116182	NM_00116182	Mm.234234	230674	ENSMUST00000106403
A_55_P2028064	**Kdm4b**	lysine (K)-specific demethylase 4B	0,79	1,72	1,93E-05	5,39E-04	NM_172132	NM_172132	NM_172132	Mm.45047	193796	ENSMUST00000086835
A_55_P2065449	**Kdm5b**	lysine (K)-specific demethylase 5B	1,69	3,24	2,02E-07	4,69E-05	NM_152895	NM_152895	NM_152895	Mm.28995	75605	ENSMUST00000047714
A_52_P551707	**Kdm6a**	lysine (K)-specific demethylase 6A	1,00	2,00	7,52E-05	1,25E-03	NM_009483	NM_009483	NM_009483	Mm.257498	22289	ENSMUST00000155161

A_51_P316553	***Kdr***	kinase insert domain protein receptor	1,15	2,22	6,44E-06	2,85E-04	NM_010612	NM_010612	NM_010612	Mm.285	16542	ENSMUST00000113516
A_52_P641849	***Khnyn***	KH and NYN domain containing	0,80	1,74	4,91E-05	9,55E-04	NM_027143	NM_027143	NM_027143	Mm.490360	219094	ENSMUST0000022831
A_55_P2082478	***Kidins220***	kinase D-interacting substrate 220	1,26	2,40	1,36E-06	1,17E-04	NM_00108137	NM_00108137	NM_00108137	Mm.250641	77480	ENSMUST00000066652
A_55_P2032449	***Kif1a***	kinesin family member 1A	1,22	2,33	2,27E-06	1,52E-04	NM_008440	NM_008440	NM_008440	Mm.276408	16560	ENSMUST00000112958
A_55_P2058933	***Kif1b***	kinesin family member 1B	1,16	2,24	8,66E-05	1,38E-03	NM_207682	NM_207682	NM_207682	Mm.402393	16561	ENSMUST00000060537
A_51_P324287	***Kif23***	kinesin family member 23	1,17	2,26	9,76E-07	9,95E-05	NM_024245	NM_024245	NM_024245	Mm.259374	71819	ENSMUST00000034815
A_55_P2085556	***Kif26a***	kinesin family member 26A	0,75	1,68	3,09E-05	7,10E-04	NM_00109762	NM_00109762	NM_00109762	Mm.313104	668303	ENSMUST00000128402
A_51_P179258	***Kif26b***	kinesin family member 26B	0,92	1,90	6,70E-06	2,91E-04	NM_00116166	NM_00116166	NM_00116166	Mm.138659	269152	ENSMUST00000161017
A_51_P254805	***Kif4***	kinesin family member 4	0,81	1,75	1,37E-04	1,86E-03	NM_008446	NM_008446	NM_008446	Mm.383091	16571	ENSMUST00000048962
A_51_P107020	***Kif5a***	kinesin family member 5A	1,94	3,83	1,08E-06	1,03E-04	NM_00103900	NM_00103900	NM_00103900	Mm.30355	16572	ENSMUST00000099172
A_55_P2037478	***Kifap3***	kinesin-associated protein 3	0,90	1,86	1,79E-05	5,15E-04	NM_010629	NM_010629	NM_010629	Mm.4651	16579	ENSMUST00000077642
A_51_P114297	***Kifc3***	kinesin family member C3	0,75	1,69	4,08E-06	2,16E-04	NM_010631	NM_010631	NM_010631	Mm.378951	16582	ENSMUST00000169748
A_66_P128434	***Kit***	kit oncogene	0,82	1,76	9,76E-06	3,63E-04	NM_00112273	NM_00112273	NM_00112273	Mm.247073	16590	ENSMUST00000144270
A_52_P420663	***Klhl11***	kelch-like 11	0,90	1,87	6,67E-06	2,90E-04	NM_172565	NM_172565	NM_172565	Mm.183320	217194	ENSMUST00000056665
A_55_P2455725	***Klhl20***	kelch-like 20	1,11	2,15	8,32E-07	9,30E-05	NM_00103948	NM_00103948	NM_00103948	Mm.255165	226541	ENSMUST00000117467
A_55_P2005285	***Klhl22***	kelch-like 22	0,84	1,80	5,98E-06	2,73E-04	NM_145479	NM_145479	NM_145479	Mm.282807	224023	ENSMUST00000117192
A_55_P1962284	***Klhl24***	kelch-like 24	1,09	2,13	4,58E-06	2,30E-04	NM_029436	NM_029436	NM_029436	Mm.485857	75785	ENSMUST00000023509
A_66_P111385	***Klhl28***	kelch-like 28	0,88	1,84	1,81E-05	5,19E-04	NM_025707	NM_025707	NM_025707	Mm.248678	66689	ENSMUST00000021331
A_55_P1981949	***Klhl5***	kelch-like 5	0,62	1,53	4,04E-05	8,38E-04	NM_175174	NM_175174	NM_175174	Mm.10281	71778	ENSMUST00000126692
A_51_P423008	***Klhl7***	kelch-like 7	0,96	1,94	1,19E-04	1,69E-03	NM_026448	NM_026448	NM_026448	Mm.273768	52323	ENSMUST00000030841
A_55_P2154714	***Klhl9***	kelch-like 9	0,95	1,94	1,36E-04	1,86E-03	NM_172871	NM_172871	NM_172871	Mm.260601	242521	ENSMUST00000094993
A_51_P176474	***Kndc1***	kinase non-catalytic C-lobe domain (KIND) containing 1	1,25	2,38	1,03E-05	3,73E-04	NM_177261	NM_177261	NM_177261	Mm.44442	76484	ENSMUST00000053445
A_55_P2078459	***Kpna2***	karyopherin (importin) alpha 2	1,02	2,03	4,76E-05	9,39E-04	NM_010655	NM_010655	NM_010655	Mm.12508	16647	ENSMUST00000086423
A_55_P2067895	***Krba1***	KRAB-A domain containing 1	0,72	1,65	8,05E-06	3,24E-04	NM_133922	NM_133922	NM_133922	Mm.107646	77827	ENSMUST00000114572
A_55_P2070940	***Krtap20-2***	keratin associated protein 20-2	0,80	1,74	9,39E-05	1,45E-03	NM_00116361	NM_00116361	NM_00116361	Mm.459837	622935	ENSMUST00000169954
A_66_P135018	***Krtap5-1***	keratin associated protein 5-1	0,59	1,51	1,25E-04	1,75E-03	NM_015808	NM_015808	NM_015808	Mm.389993	50774	ENSMUST00000106074
A_55_P2065199	***Krtap9-5***	keratin associated protein 9-5	0,63	1,55	2,50E-05	6,26E-04	NM_00108552	NM_00108552	NM_00108552	Mm.457964	435286	ENSMUST00000105052
A_55_P2102857	***Krtcap3***	keratinocyte associated protein 3	1,20	2,30	4,13E-06	2,16E-04	NM_027221	NM_027221	NM_027221	Mm.389609	69815	ENSMUST00000054829
A_55_P2008681	***Ktn1***	kinectin 1	1,46	2,75	4,34E-08	2,20E-05	NM_008477	NM_008477	NM_008477	Mm.3110	16709	ENSMUST00000022391
A_55_P1967659	***Lactb***	lactamase, beta	0,80	1,74	6,43E-06	2,85E-04	NM_030717	NM_030717	NM_030717	Mm.157882	80907	ENSMUST00000034929
A_51_P418908	***Larp1***	La ribonucleoprotein domain family, member 1	0,75	1,69	7,00E-05	1,19E-03	NM_028451	NM_028451	NM_028451	Mm.248843	73158	ENSMUST00000071487
A_52_P239726	***Larp4***	La ribonucleoprotein domain family, member 4	1,00	2,00	2,26E-05	5,90E-04	NM_00108094	NM_00108094	NM_00108094	Mm.28811	207214	ENSMUST00000057632
A_52_P500488	***Lars***	leucyl-tRNA synthetase	0,98	1,97	4,97E-06	2,42E-04	NM_134137	NM_134137	NM_134137	Mm.312170	107045	ENSMUST00000097590
A_55_P1957227	***Las1l***	LAS1-like (S. cerevisiae)	0,90	1,87	8,41E-06	3,32E-04	NM_152822	NM_152822	NM_152822	Mm.274318	76130	ENSMUST00000079987
A_52_P667287	***Lass6***	LAG1 homolog, ceramide synthase 6	1,16	2,24	3,57E-05	7,74E-04	NM_172856	NM_172856	NM_172856	Mm.222222	241447	ENSMUST00000028426
A_51_P201137	***Lbr***	lamin B receptor	1,00	1,99	1,02E-05	3,71E-04	NM_133815	NM_133815	NM_133815	Mm.4538	98386	ENSMUST00000005003
A_52_P299358	***Lclat1***	lysocardiolipin acyltransferase 1	0,94	1,92	6,26E-06	2,81E-04	NM_00108107	NM_00108107	NM_00108107	Mm.329194	225010	ENSMUST00000067545
A_52_P67983	***Lcmt2***	leucine carboxyl methyltransferase 2	0,69	1,61	8,31E-05	1,34E-03	NM_177846	NM_177846	NM_177846	Mm.219449	329504	ENSMUST00000099486
A_52_P56751	***Lcp1***	lymphocyte cytosolic protein 1	1,56	2,94	7,29E-07	8,76E-05	NM_008879	NM_008879	NM_008879	Mm.153911	18826	ENSMUST00000131802
A_55_P2141876	***Ldha***	lactate dehydrogenase A	0,76	1,70	3,28E-05	7,36E-04	NM_00113606	NM_00113606	NM_00113606	Mm.29324	16828	ENSMUST00000125862
A_51_P444645	***Lect1***	leukocyte cell derived chemotaxin 1	1,26	2,40	1,24E-05	4,12E-04	NM_010701	NM_010701	NM_010701	Mm.46561	16840	ENSMUST00000022603
A_55_P2177910	***Lepr***	leptin receptor	0,96	1,94	4,13E-05	8,50E-04	NM_010704	NM_010704	NM_010704	Mm.259282	16847	ENSMUST00000156402
A_52_P609695	***Lgi1***	leucine-rich repeat LGI family, member 1	1,40	2,63	2,94E-06	1,78E-04	NM_020278	NM_020278	NM_020278	Mm.298251	56839	ENSMUST00000087252
A_55_P2179453	***Lig3***	ligase III, DNA, ATP-dependent	0,78	1,71	5,05E-05	9,71E-04	NM_010716	NM_010716	NM_010716	Mm.277136	16882	ENSMUST00000080461
A_65_P01834	***Lima1***	LIM domain and actin binding 1	2,78	6,89	3,92E-09	7,48E-06	NM_00111354	NM_00111354	NM_00111354	Mm.33207	65970	ENSMUST00000109024
A_55_P2023727	***Limch1***	LIM and calponin homology domains 1	3,98	15,81	1,53E-10	3,00E-06	NM_00100198	NM_00100198	NM_00100198	Mm.481371	77569	ENSMUST00000038188
A_55_P2085641	***Lmbr1l***	limb region 1 like	0,56	1,48	8,18E-05	1,32E-03	NM_029098	NM_029098	NM_029098	Mm.385020	74775	ENSMUST00000109127
A_55_P2123942	***Lmbrd1***	LMBR1 domain containing 1	0,55	1,47	8,57E-05	1,36E-03	NM_026719	NM_026719	NM_026719	Mm.336563	68421	ENSMUST00000095062
A_55_P1985273	***Lmcd1***	LIM and cysteine-rich domains 1	0,86	1,81	1,52E-04	1,99E-03	NM_144799	NM_144799	NM_144799	Mm.234441	30937	ENSMUST00000032376
A_55_P2177988	***Lmf1***	lipase maturation factor 1	0,80	1,74	7,65E-06	3,13E-04	NM_029624	NM_029624	NM_029624	Mm.12787	76483	ENSMUST00000156868
A_52_P54261	***Lmf2***	lipase maturation factor 2	0,48	1,40	1,42E-04	1,91E-03	NM_178919	NM_178919	NM_178919	Mm.34511	105847	ENSMUST00000023283
A_55_P2110512	***Lmo7***	LIM domain only 7	2,95	7,71	2,35E-09	5,69E-06	NM_201529	NM_201529	NM_201529	Mm.486662	380928	ENSMUST00000160038
A_52_P445474	***Lmtk2***	lemur tyrosine kinase 2	2,00	4,01	1,66E-05	4,91E-04	NM_00108110	NM_00108110	NM_00108110	Mm.288726	231876	ENSMUST00000041804
A_55_P1976200	***Lmtk3***	lemur tyrosine kinase 3	1,51	2,85	1,96E-07	4,65E-05	NM_00100551	NM_00100551	NM_00100551	Mm.44928	381983	ENSMUST00000120005
A_55_P1995233	***Lonp2***	lon peptidase 2, peroxisomal	1,57	2,96	4,52E-07	7,01E-05	NM_025827	NM_025827	NM_025827	Mm.324550	66887	ENSMUST00000034141
A_55_P2115008	***Lonrf2***	LON peptidase N-terminal domain and ring finger 2	1,10	2,15	2,18E-06	1,49E-04	NM_00102987	NM_00102987	NM_00102987	Mm.487137	381338	ENSMUST00000039612
A_55_P2013019	***Lpar1***	lysophosphatidic acid receptor 1	0,92	1,89	2,19E-06	1,49E-04	NM_172989	NM_172989	NM_172989	Mm.4772	14745	ENSMUST00000107571
A_51_P145415	***Lpcat3***	lysophosphatidylcholine acyltransferase 3	1,20	2,29	3,06E-05	7,08E-04	NM_145130	NM_145130	NM_145130	Mm.273915	14792	ENSMUST00000130020
A_51_P453351	***Lpgat1***	lysophosphatidylglycerol acyltransferase 1	0,89	1,85	4,49E-06	2,27E-04	NM_172266	NM_172266	NM_172266	Mm.277958	226856	ENSMUST00000110855
A_55_P2040200	***Lrba***	LPS-responsive beige-like anchor	0,74	1,67	3,89E-06	2,10E-04	NM_030695	NM_030695	NM_030695	Mm.439825	80877	ENSMUST00000107635
A_55_P2400322	***Lrch2***	leucine-rich repeats and calponin homology (CH) domain cont	0,75	1,68	4,39E-05	8,86E-04	NM_00108117	NM_00108117	NM_00108117	Mm.30506	210297	ENSMUST00000112819
A_51_P295237	***Lrp11***	low density lipoprotein receptor-related protein 11	0,74	1,67	1,74E-05	5,06E-04	NM_172784	NM_172784	NM_172784	Mm.206759	237253	ENSMUST00000019931
A_51_P476428	***Lrp1b***	low density lipoprotein-related protein 1B (deleted in tumors)	0,95	1,94	7,22E-06	3,03E-04	NM_053011	NM_053011	NM_053011	Mm.441398	94217	ENSMUST00000129974
A_55_P1967168	***Lrp4***	low density lipoprotein receptor-related protein 4	1,16	2,24	1,63E-05	4,87E-04	NM_172668	NM_172668	NM_172668	Mm.275149	228357	ENSMUST00000028689
A_52_P279687	***Lrp8***	low density lipoprotein receptor-related protein 8, apolipoprote	0,66	1,58	2,46E-05	6,21E-04	NM_00108092	NM_00108092	NM_00108092	Mm.442134	16975	ENSMUST00000123140
A_55_P1969845	***Lrpprc***	leucine-rich PPR-motif containing	1,03	2,04	3,99E-06	2,13E-04	NM_028233	NM_028233	NM_028233	Mm.217027	72416	ENSMUST00000112308
A_52_P11402	***Lrrc24***	leucine rich repeat containing 24	0,80	1,74	5,51E-05	1,03E-03	NM_198119	NM_198119	NM_198119	Mm.289666	378937	ENSMUST00000049956
A_51_P362089	***Lrrc3b***	leucine rich repeat containing 3B	0,69	1,61	9,93E-05	1,51E-03	NM_146052	NM_146052	NM_146052	Mm.138039	218763	ENSMUST00000163937
A_52_P365660	***Lrrc4c***	leucine rich repeat containing 4C	0,59	1,51	6,59E-05	1,15E-03	NM_178725	NM_178725	NM_178725	Mm.241682	241568	ENSMUST00000162807
A_55_P2125564	***Lrrc58***	leucine rich repeat containing 58	0,82	1,77	5,99E-05	1,08E-03	NM_177093	NM_177093	NM_177093	Mm.390882	320184	ENSMUST00000078717
A_51_P485472	***Lrrc71***	leucine rich repeat containing 71	1,94	3,84	2,26E-08	1,66E-05	NM_028971	NM_028971	NM_028971	Mm.474494	74485	ENSMUST00000023846
A_55_P1984406	***Lrrk2***	leucine-rich repeat kinase 2	0,52	1,44	1,44E-04	1,92E-03	NM_025730	NM_025730	NM_025730	Mm.37558	66725	ENSMUST00000137657
A_55_P1973033	***Ltbp1***	latent transforming growth factor beta binding protein 1	1,45	2,73	2,44E-05	6,17E-04	NM_019919	NM_019919	NM_019919	Mm.269747	268977	ENSMUST00000112516
A_55_P2033500	***Ltbp4***	latent transforming growth factor beta binding protein 4	1,19	2,28	1,71E-06	1,30E-04	NM_175641	NM_175641	NM_175641	Mm.272251	108075	ENSMUST00000108369
A_52_P427240	***Ltn1***	listerin E3 ubiquitin protein ligase 1	1,22	2,33	6,49E-06	2,85E-04	NM_00108106	NM_00108106	NM_00108106	Mm.249005	78913	ENSMUST00000039449
A_55_P2011610	***Lyst***	lysosomal trafficking regulator	1,11	2,16	3,87E-06	2,10E-04	NM_010748	NM_010748	NM_010748	Mm.342337	17101	ENSMUST00000110559
A_55_P2017684	***Macf1***	microtubule-actin crosslinking factor 1	1,39	2,62	2,19E-05	5,83E-04	NM_00119913	NM_00119913	NM_00119913	Mm.402299	11426	ENSMUST00000106224
A_55_P2009091	***Mad1l1***	MAD1 mitotic arrest deficient 1-like 1	0,53	1,45	4,97E-05	9,60E-04	NM_010752	NM_010752	NM_010752	Mm.27250	17120	ENSMUST00000031534
A_55_P2062757	***Mag***	myelin-associated glycoprotein	0,85	1,80	1,17E-04	1,68E-03	NM_010758	NM_010758	NM_010758	Mm.241355	17136	ENSMUST00000040548
A_51_P104430	***Magee1***	melanoma antigen, family E, 1	2,09	4,24	4,58E-07	7,06E-05	NM_053201	NM_053201	NM_053201	Mm.24341	107528	ENSMUST00000033578
A_55_P2003251	***Magel2***	melanoma antigen, family L, 2	0,82	1,76	4,03E-05	8,38E-04	NM_013779	NM_013779	NM_013779	Mm.45207	27385	ENSMUST00000080403
A_55_P2062642	***Magt1***	magnesium transporter 1	0,70	1,62	1,41E-04	1,89E-03	NM_025952	NM_025952	NM_025952	Mm.275943	67075	ENSMUST00000033583
A_51_P324923	***Man2a1***	mannosidase 2, alpha 1	0,65	1,57	3,40E-05	7,51E-04	NM_008549	NM_008549	NM_008549	Mm.2433	17158	ENSMUST00000086723
A_51_P336161	***Man2b1***	mannosidase 2, alpha B1	1,28	2,42	1,41E-04	1,90E-03	NM_010764	NM_010764	NM_010764	Mm.4219	17159	ENSMUST00000034121
A_51_P418168	***Manf***	mesencephalic astrocyte-derived neurotrophic factor	0,71	1,63	6,24E-05	1,11E-03	NM_029103	NM_029103	NM_029103	Mm.29778	74840	ENSMUST00000159283
A_66_P140452	***Map1a***	microtubule-associated protein 1 A	1,27	2,41	8,07E-05	1,31E-03	NM_032393	NM_032393	NM_032393	Mm.36501	17754	ENSMUST00000094639
A_55_P1964193	***Map1b***	microtubule-associated protein 1B	2,02	4,04	6,57E-06	2,88E-04	NM_008634	NM_008634	NM_008634	Mm.4173	17755	ENSMUST00000064762
A_55_P2059312	***Map2***	microtubule-associated protein 2	0,94	1,92	1,04E-04	1,55E-03	ENSMUST000	0	M21041	Mm.487809	17756	ENSMUST00000114013
A_51_P402193	***Map3k1***	mitogen-activated protein kinase kinase kinase 1	0,99	1,99	1,23E-05	4,11E-04	NM_011945	NM_011945	NM_011945	Mm.15918	26401	ENSMUST00000109267
A_51_P439876	***Map4k4***	mitogen-activated protein kinase kinase kinase kinase 4	0,60	1,52	1,08E-04	1,59E-03	NM_0012522	NM_0012522	NM_0012522	Mm.19073	26921	ENSMUST00000163854
A_55_P2074836	***Mapk8ip1***	mitogen-activated protein kinase 8 interacting protein 1	0,92	1,89	3,00E-05	7,00E-04	NM_00120244	NM_00120244	NM_00120244	Mm.2720	19099	ENSMUST00000111279
A_52_P370392	***Mapk8ip2***	mitogen-activated protein kinase 8 interacting protein 2	2,89	7,39	2,26E-08	1,66E-05	NM_021921	NM_021921	NM_021921	Mm.482169	60597	ENSMUST00000023291
A_55_P2085751	***Mapk8ip3***	mitogen-activated protein kinase 8 interacting protein 3	1,27	2,41	5,27E-05	9,97E-04	NM_00116344	NM_00116344	NM_00116344	Mm.43081	30957	ENSMUST00000119115
A_55_P2038504	***March6***	membrane-associated ring finger (C3HC4) 6	1,40	2,63	4,01E-06	2,14E-04	NM_172606	NM_172606	NM_172606	Mm.272185	223455	ENSMUST00000090227
A_65_P19138	***March7***	membrane-associated ring finger (C3HC4) 7	0,76	1,69	2,78E-05	6,68E-04	ENSMUST000	0	AK155072	Mm.260635	57438	ENSMUST00000102748
A_55_P2076303	***March8***	membrane-associated ring finger (C3HC4) 8	0,62	1,54	9,93E-05	1,51E-03	NM_027920	NM_027920	NM_027920	Mm.27064	71779	ENSMUST00000101032
A_55_P2133337	***March9***	membrane-associated ring finger (C3HC4) 9	1,19	2,28	8,44E-07	9,35E-05	ENSMUST000	0	0	0	216438	ENSMUST00000040307
A_51_P479902	***Mars***	methionine-tRNA synthetase	1,04	2,05	8,51E-06	3,35E-04	NM_00100391	NM_00100391	NM_00100391	Mm.28173	216443	ENSMUST00000139683
A_55_P2177659	***Mast1***	microtubule associated serine/threonine kinase 1	1,03	2,04	1,28E-05	4,21E-04	NM_019945	NM_019945	NM_019945	Mm.154614	56527	ENSMUST00000109741
A_55_P2058403	***Mast3***	microtubule associated serine/threonine kinase 3	0,79	1,73	8,69E-05	1,38E-03	NM_199308	NM_199308	NM_199308	Mm.250731	546071	ENSMUST00000166004
A_51_P168695	***Mast4***	microtubule associated serine/threonine kinase family membe	0,71	1,63	1,32E-04	1,83E-03	NM_175171	NM_175171	NM_175171	Mm.202606	328329	ENSMUST00000167058
A_55_P2095047	***Mat2a***	methionine adenosyltransferase II, alpha	1,07	2,10	1,04E-05	3,75E-04	NM_145569	NM_145569	NM_145569	Mm.485037	232087	ENSMUST00000059472
A_55_P2151751	***Matr3***	matrin 3	1,82	3,53	3,17E-07	6,03E-05	NM_010771	NM_010771	NM_010771	Mm.215034	17184	ENSMUST00000166793
A_55_P1963585	***Mbd5***	methyl-CpG binding domain protein 5	0,57	1,48	8,98E-05	1,41E-03	NM_029924	NM_029924	NM_029924	Mm.440436	109241	ENSMUST00000112754
A_51_P144868	***Mboat7***	membrane bound O-acyltransferase domain containing 7	0,88	1,84	5,02E-05	9,66E-04	NM_029934	NM_029934	NM_029934	Mm.490287	77582	ENSMUST00000127106
A_55_P2185910	***Mbtps2***	membrane-bound transcription factor peptidase, site 2	0,57	1,49	9,93E-05	1,51E-03	NM_172307	NM_172307	NM_172307	Mm.37577	270669	ENSMUST00000065857
A_55_P1986551	***Mcam***	melanoma cell adhesion molecule	0,80	1,74	6,70E-05	1,16E-03	NM_023061	NM_023061	NM_023061	Mm.275003	84004	ENSMUST00000034650
A_55_P1953899	***Mccc1***	methylcrotonoyl-Coenzyme A carboxylase 1 (alpha)	0,86	1,82	2,41E-06	1,57E-04	NM_023644	NM_023644	NM_023644	Mm.249016	72039	ENSMUST00000029259
A_55_P2036176	***Mcf2l***	mcf.2 transforming sequence-like	1,42	2,67	1,24E-06	1,10E-04	NM_178076	NM_178076	NM_178076	Mm.334413	17207	ENSMUST00000110867
A_52_P93933	***Mcl1***	myeloid cell leukemia sequence 1	0,78	1,72	1,09E-04	1,61E-03	NM_008562	NM_008562	NM_008562	Mm.1639	17210	ENSMUST00000037947

A_55_P2182134	Mctp1	multiple C2 domains, transmembrane 1	0,95	1,93	9,76E-06	3,63E-04	NM_030174	NM_030174	NM_030174	Mm.316249	78771	ENSMUST00000109583
A_52_P407796	Mdh1	malate dehydrogenase 1, NAD (soluble)	0,93	1,90	8,07E-05	1,31E-03	NM_008618	NM_008618	NM_008618	Mm.212703	17449	ENSMUST00000102874
A_55_P2016842	Me1	malic enzyme 1, NADP(+)-dependent, cytosolic	0,97	1,95	6,97E-05	1,19E-03	NM_00119893	NM_00119893	NM_00119893	Mm.148155	17436	ENSMUST00000034989
A_51_P324082	Med1	mediator complex subunit 1	1,01	2,01	1,31E-04	1,81E-03	NM_013634	NM_013634	NM_013634	Mm.12926	19014	ENSMUST00000018304
A_66_P136955	Med14	mediator complex subunit 14	1,33	2,51	5,77E-07	7,91E-05	NM_012005	NM_012005	NM_012005	Mm.17616	26896	ENSMUST00000124053
A_55_P1964568	Megf10	multiple EGF-like-domains 10	1,38	2,61	3,31E-07	6,08E-05	NM_00100197	NM_00100197	NM_00100197	Mm.297863	70417	ENSMUST00000075770
A_51_P124535	Mest	mesoderm specific transcript	1,29	2,44	4,33E-06	2,22E-04	NM_008590	NM_008590	NM_008590	Mm.335639	17294	ENSMUST00000163949
A_51_P102503	Metap1	methionyl aminopeptidase 1	0,78	1,71	3,11E-05	7,11E-04	NM_175224	NM_175224	NM_175224	Mm.26833	75624	ENSMUST00000029804
A_51_P345046	Metap2	methionine aminopeptidase 2	0,93	1,90	1,67E-05	4,93E-04	NM_019648	NM_019648	NM_019648	Mm.289329	56307	ENSMUST00000180840
A_66_P134412	Mettl25	methyltransferase like 25	0,86	1,82	6,05E-05	1,09E-03	NM_207522	NM_207522	NM_207522	Mm.250875	216292	ENSMUST00000176924
A_55_P1961140	Mettl3	methyltransferase like 3	0,73	1,65	3,88E-05	8,15E-04	NM_019721	NM_019721	NM_019721	Mm.271759	56335	ENSMUST00000173546
A_66_P133270	Mfsd1	major facilitator superfamily domain containing 1	0,83	1,78	1,22E-05	4,10E-04	NM_025813	NM_025813	NM_025813	Mm.271975	66868	ENSMUST00000029344
A_55_P2452384	Mga	MAX gene associated	1,20	2,30	7,29E-06	3,05E-04	NM_013720	NM_013720	NM_013720	Mm.87532	29808	ENSMUST00000046717
A_55_P2130388	Mical1	microtubule associated monoxygenase, calponin and LIM dor	2,41	5,30	2,07E-08	1,63E-05	NM_138315	NM_138315	NM_138315	Mm.290431	171580	ENSMUST00000019967
A_51_P519648	Mical3	microtubule associated monoxygenase, calponin and LIM dor	2,07	4,20	1,53E-07	4,22E-05	NM_153396	NM_153396	NM_153396	Mm.122399	194401	ENSMUST00000077159
A_52_P642207	Mkrn1	makorin, ring finger protein, 1	0,63	1,55	6,69E-05	1,16E-03	NM_018810	NM_018810	NM_018810	Mm.270484	54484	ENSMUST00000031985
A_51_P427828	Mlip	muscular LMNA-interacting protein	0,89	1,85	1,17E-05	4,00E-04	AK009836	0	AK009836	Mm.35008	69642	0
A_55_P2185990	Mll1	myeloid/lymphoid or mixed-lineage leukemia 1	1,58	2,98	2,31E-06	1,54E-04	NM_00108104	NM_00108104	NM_00108104	Mm.2389	214162	ENSMUST00000152241
A_55_P2136526	Mll2	myeloid/lymphoid or mixed-lineage leukemia 2	1,29	2,44	4,24E-07	6,84E-05	NM_00103327	NM_00103327	NM_00103327	Mm.264889	381022	ENSMUST00000023741
A_55_P1984123	Mll3	myeloid/lymphoid or mixed-lineage leukemia 3	1,23	2,35	1,80E-07	4,47E-05	NM_00108138	NM_00108138	NM_00108138	Mm.332268	231051	ENSMUST00000174734
A_52_P304128	Mmp14	matrix metallopeptidase 14 (membrane-inserted)	0,96	1,94	6,74E-05	1,17E-03	NM_008608	NM_008608	NM_008608	Mm.280175	17387	ENSMUST00000089688
A_51_P195775	Mmp16	matrix metallopeptidase 16	1,36	2,56	3,82E-07	6,44E-05	NM_019724	NM_019724	NM_019724	Mm.187315	17389	ENSMUST00000029881
A_51_P341736	Mmp2	matrix metallopeptidase 2	0,84	1,79	1,99E-05	5,48E-04	NM_008610	NM_008610	NM_008610	Mm.29564	17390	ENSMUST00000034187
A_51_P414396	Mmrn2	multimerin 2	0,67	1,59	1,16E-04	1,67E-03	NM_153127	NM_153127	NM_153127	Mm.272673	105450	ENSMUST00000111908
A_55_P2352896	Mon2	MON2 homolog (yeast)	1,40	2,64	7,11E-06	3,02E-04	NM_153395	NM_153395	NM_153395	Mm.169924	67074	ENSMUST00000037557
A_55_P2137887	Mpdz	multiple PDZ domain protein	1,22	2,33	1,17E-06	1,08E-04	NM_010820	NM_010820	NM_010820	Mm.153039	17475	ENSMUST00000134726
A_55_P2115127	Mphosph10	M-phase phosphoprotein 10 (U3 small nucleolar ribonucleopro	1,44	2,71	6,32E-07	8,24E-05	NM_026483	NM_026483	NM_026483	Mm.26973	67973	ENSMUST00000032735
A_55_P2077746	Mphosph8	M-phase phosphoprotein 8	0,71	1,64	3,29E-05	7,36E-04	NM_023773	NM_023773	NM_023773	Mm.152466	75339	ENSMUST00000116468
A_55_P2079520	Mpp1	membrane protein, palmitoylated	0,84	1,78	4,55E-05	9,09E-04	NM_008621	NM_008621	NM_008621	Mm.391267	17524	ENSMUST00000033775
A_52_P17098	Mpp7	membrane protein, palmitoylated 7 (MAGUK p55 subfamily m	0,87	1,83	1,14E-04	1,65E-03	NM_00116162	NM_00116162	NM_00116162	Mm.479293	75739	ENSMUST00000115869
A_55_P1957433	Mprip	myosin phosphatase Rho interacting protein	3,13	8,78	1,60E-08	1,38E-05	NM_201245	NM_201245	NM_201245	Mm.2402	26936	ENSMUST00000066330
A_55_P2097038	Mrc2	mannose receptor, C type 2	1,20	2,30	1,39E-06	1,17E-04	NM_008626	NM_008626	NM_008626	Mm.235616	17534	ENSMUST00000126931
A_52_P368057	Mri1	methylthioribose-1-phosphate isomerase homolog (S. cerevis	0,82	1,77	3,79E-06	2,07E-04	NM_026423	NM_026423	NM_026423	Mm.278085	67873	ENSMUST00000126435
A_55_P2087885	Mroh1	maestro heat-like repeat family member 1	0,61	1,52	1,35E-04	1,84E-03	NM_00116248	NM_00116248	NM_00116248	Mm.123240	223658	ENSMUST00000161683
A_51_P405565	Msh6	mutS homolog 6 (E. coli)	1,27	2,41	3,69E-07	6,32E-05	NM_010830	NM_010830	NM_010830	Mm.18210	17688	ENSMUST00000005503
A_55_P2022094	Mta3	metastasis associated 3	0,82	1,76	3,14E-06	1,85E-04	NM_00117105	NM_00117105	NM_00117105	Mm.277668	116871	ENSMUST00000112350
A_55_P2000280	Mthfd1l	methylenetetrahydrofolate dehydrogenase (NADP+ depender	0,92	1,89	8,21E-06	3,28E-04	NM_00117078	NM_00117078	NM_00117078	Mm.184752	270685	ENSMUST00000120585
A_55_P2117028	Mtmr3	myotubularin related protein 3	0,50	1,41	6,84E-05	1,18E-03	NM_028860	NM_028860	NM_028860	Mm.425669	74302	ENSMUST00000123506
A_52_P450188	Mtmr4	myotubularin related protein 4	0,83	1,78	9,96E-06	3,68E-04	NM_133215	NM_133215	NM_133215	Mm.399376	170749	ENSMUST00000119628
A_55_P2084378	Mtmr7	myotubularin related protein 7	1,26	2,40	2,15E-06	1,47E-04	NM_0010406	NM_0010406	NM_0010406	Mm.294871	54384	ENSMUST00000048898
A_51_P282179	Mtor	mechanistic target of rapamycin (serine/threonine kinase)	1,71	3,27	1,55E-06	1,23E-04	NM_020009	NM_020009	NM_020009	Mm.21158	56717	ENSMUST00000103221
A_55_P2152926	Mybbp1a	MYB binding protein (P160) 1a	0,78	1,72	4,10E-06	2,16E-04	NM_016776	NM_016776	NM_016776	Mm.147946	18432	ENSMUST00000152894
A_52_P108346	Myc	myelocytomatosis oncogene	1,07	2,10	8,29E-07	9,30E-05	NM_010849	NM_010849	NM_010849	Mm.2444	17869	ENSMUST00000167731
A_51_P473919	Mycbp2	MYC binding protein 2	2,15	4,43	7,23E-09	9,49E-06	NM_207215	NM_207215	NM_207215	Mm.6478	105689	ENSMUST00000159855
A_52_P536494	Mycn	v-myc myelocytomatosis viral related oncogene, neuroblaston	1,24	2,37	4,35E-05	8,81E-04	NM_008709	NM_008709	NM_008709	Mm.16469	18109	ENSMUST00000043396
A_55_P2088525	Myh10	myosin, heavy polypeptide 10, non-muscle	2,90	7,48	4,29E-08	2,20E-05	NM_175260	NM_175260	NM_175260	Mm.218233	77579	ENSMUST00000102611
A_55_P2090070	Myh14	myosin, heavy polypeptide 14	2,10	4,30	1,12E-08	1,19E-05	NM_00127153	NM_00127153	NM_00127153	Mm.158289	71960	ENSMUST00000107900
A_55_P2028600	Myh9	myosin, heavy polypeptide 9, non-muscle	4,00	15,98	8,80E-10	4,68E-06	NM_022410	NM_022410	NM_022410	Mm.29677	17886	ENSMUST00000016771
A_55_P1993744	Myo10	myosin X	1,92	3,78	4,14E-08	2,20E-05	NM_019472	NM_019472	NM_019472	Mm.60590	17909	ENSMUST00000110457
A_55_P2181306	Myo16	myosin XVI	0,97	1,96	2,61E-05	6,45E-04	NM_00108139	NM_00108139	NM_00108139	Mm.422761	244281	ENSMUST00000042103
A_55_P2154054	Myo18a	myosin XVIIIA	3,08	8,45	3,11E-09	6,76E-06	NM_011586	NM_011586	NM_011586	Mm.341248	360013	ENSMUST00000108375
A_51_P434171	Myo19	myosin XIX	1,46	2,75	6,65E-06	2,90E-04	NM_025414	NM_025414	NM_025414	Mm.288689	66196	ENSMUST00000141705
A_55_P1955039	Myo1b	myosin IB	1,24	2,36	4,94E-07	7,25E-05	NM_00116181	NM_00116181	NM_00116181	Mm.3390	17912	ENSMUST00000018561
A_52_P650855	Myo1d	myosin ID	1,16	2,23	1,24E-06	1,10E-04	NM_177390	NM_177390	NM_177390	Mm.151948	338367	ENSMUST00000041065
A_51_P406583	Myo1e	myosin IE	2,79	6,91	8,28E-10	4,68E-06	NM_181072	NM_181072	NM_181072	Mm.249311	71602	ENSMUST00000034745
A_66_P112305	Myo1f	myosin IF	0,71	1,63	4,76E-05	9,39E-04	NM_053214	NM_053214	NM_053214	Mm.42019	17916	ENSMUST00000173372
A_55_P2006250	Myo5a	myosin VA	2,24	4,74	3,35E-08	1,92E-05	NM_010864	NM_010864	NM_010864	Mm.3645	17918	ENSMUST00000123128
A_55_P2096081	Myo5b	myosin VB	1,38	2,60	3,44E-07	6,10E-05	NM_201600	NM_201600	NM_201600	Mm.260098	17919	ENSMUST00000121875
A_55_P2024669	Myo6	myosin VI	3,60	12,11	1,01E-09	4,68E-06	NM_00103954	NM_00103954	NM_00103954	Mm.4040	17920	ENSMUST00000127779
A_52_P201531	Myo9a	myosin IXa	1,09	2,12	3,18E-06	1,86E-04	NM_173018	NM_173018	NM_173018	Mm.249545	270163	ENSMUST00000136740
A_51_P375558	Myoc	myocilin	1,33	2,52	5,42E-06	2,55E-04	NM_010865	NM_010865	NM_010865	Mm.10694	17926	ENSMUST00000028020
A_52_P173703	Myrip	myosin VIIA and Rab interacting protein	0,92	1,89	3,49E-05	7,65E-04	NM_144557	NM_144557	NM_144557	Mm.100936	245049	ENSMUST00000048121
A_55_P2056120	Myt1l	myelin transcription factor 1-like	1,40	2,64	1,75E-07	4,42E-05	NM_00109377	NM_00109377	NM_00109377	Mm.253067	17933	ENSMUST00000049784
A_55_P2452914	N4bp2	NEDD4 binding protein 2	1,68	3,21	1,47E-08	1,34E-05	NM_00102491	NM_00102491	NM_00102491	Mm.332398	333789	ENSMUST00000138239
A_51_P414448	Naa15	N(alpha)-acetyltransferase 15, NatA auxiliary subunit	0,76	1,69	2,50E-05	6,26E-04	NM_053089	NM_053089	NM_053089	Mm.275281	74838	ENSMUST00000029303
A_52_P443167	Naa35	N(alpha)-acetyltransferase 35, NatC auxiliary subunit	0,88	1,84	1,43E-04	1,92E-03	NM_030153	NM_030153	NM_030153	Mm.253902	78689	ENSMUST00000172118
A_52_P111866	Naa50	N(alpha)-acetyltransferase 50, NatE catalytic subunit	0,88	1,84	3,40E-05	7,51E-04	NM_028108	NM_028108	NM_028108	Mm.278726	72117	ENSMUST00000063520
A_55_P2005853	Nacc2	nucleus accumbens associated 2, BEN and BTB (POZ) doma	0,87	1,83	4,78E-05	9,41E-04	NM_0010370	NM_0010370	NM_0010370	Mm.131074	67991	ENSMUST00000114159
A_51_P387235	Nampt	nicotinamide phosphoribosyltransferase	0,70	1,62	1,85E-05	5,26E-04	NM_021524	NM_021524	NM_021524	Mm.489597	59027	ENSMUST00000020886
A_51_P366207	Nap1l2	nucleosome assembly protein 1-like 2	0,79	1,72	3,89E-05	8,16E-04	NM_008671	NM_008671	NM_008671	Mm.388694	17954	ENSMUST00000121720
A_51_P213928	Nap1l3	nucleosome assembly protein 1-like 3	0,90	1,86	4,46E-06	2,27E-04	NM_138742	NM_138742	NM_138742	Mm.490573	54561	ENSMUST00000079490
A_52_P201551	Nav1	neuron navigator 1	1,81	3,49	1,05E-06	1,02E-04	NM_173437	NM_173437	NM_173437	Mm.34977	215690	ENSMUST00000040599
A_65_P03874	Nav2	neuron navigator 2	1,79	3,46	1,90E-07	4,58E-05	NM_175272	NM_175272	NM_175272	Mm.333881	78286	ENSMUST00000064395
A_55_P2126615	Nav3	neuron navigator 3	1,29	2,45	6,00E-06	2,74E-04	AK035945	0	AK035945	Mm.225050	260315	0
A_52_P80567	Nbas	neuroblastoma amplified sequence	1,77	3,40	4,52E-07	7,01E-05	NM_027706	NM_027706	NM_027706	Mm.177520	71169	ENSMUST00000042953
A_55_P2250391	Nbea	neurobeachin	1,33	2,51	7,62E-06	3,13E-04	NM_030595	NM_030595	NM_030595	Mm.384353	26422	ENSMUST00000029374
A_55_P2133524	Nbr1	neighbor of Brca1 gene 1	1,50	2,82	1,16E-07	3,76E-05	NM_008676	NM_008676	NM_008676	Mm.784	17966	ENSMUST00000141170
A_55_P2050602	Ncam1	neural cell adhesion molecule 1	0,91	1,87	1,50E-05	4,63E-04	NM_010875	NM_010875	NM_010875	Mm.4974	17967	0
A_52_P209119	Ncam2	neural cell adhesion molecule 2	1,56	2,96	3,28E-06	1,90E-04	NM_010954	NM_010954	NM_010954	Mm.433941	17968	ENSMUST00000067602
A_55_P2154228	Ncapd3	non-SMC condensin II complex, subunit D3	1,17	2,25	1,48E-06	1,21E-04	NM_178113	NM_178113	NM_178113	Mm.251777	78658	ENSMUST00000086198
A_55_P1967291	Ncaph	non-SMC condensin I complex, subunit H	0,91	1,87	1,10E-04	1,61E-03	NM_144818	NM_144818	NM_144818	Mm.29786	215387	ENSMUST00000110387
A_55_P1998545	Ncdn	neurochondrin	0,53	1,44	1,45E-04	1,94E-03	NM_011986	NM_011986	NM_011986	Mm.456184	26562	ENSMUST00000030637
A_55_P2128695	Nckap1	NCK-associated protein 1	1,13	2,19	5,61E-06	2,61E-04	NM_016965	NM_016965	NM_016965	Mm.25203	50884	ENSMUST00000111760
A_51_P349281	Nckap5l	NCK-associated protein 5-like	0,84	1,78	2,79E-05	6,69E-04	NM_0010018	NM_0010018	NM_0010018	Mm.22915	380969	ENSMUST00000023747
A_51_P134007	Ncl	nucleolin	0,93	1,91	6,61E-06	2,89E-04	NM_010880	NM_010880	NM_010880	Mm.154378	17975	ENSMUST00000027438
A_55_P2165974	Ncor1	nuclear receptor co-repressor 1	1,07	2,10	6,74E-05	1,17E-03	ENSMUST000	0	0	0	20185	ENSMUST00000101068
A_51_P396643	Ncstn	nicastrin	0,81	1,75	1,00E-04	1,51E-03	NM_021607	NM_021607	NM_021607	Mm.218203	59287	ENSMUST00000122986
A_55_P2116973	Neb	nebulin	1,10	2,15	3,98E-05	8,30E-04	NM_010889	NM_010889	NM_010889	Mm.389247	17996	ENSMUST00000139672
A_52_P648424	Nedd4	neural precursor cell expressed, developmentally down-regula	1,43	2,69	1,61E-05	4,83E-04	NM_010890	NM_010890	NM_010890	Mm.279923	17999	ENSMUST00000034740
A_55_P2030752	Nedd4l	neural precursor cell expressed, developmentally down-regula	1,27	2,41	4,16E-06	2,17E-04	NM_031881	NM_031881	NM_031881	Mm.98668	83814	ENSMUST00000080418
A_55_P2053181	Nefh	neurofilament, heavy polypeptide	1,86	3,62	5,50E-09	8,90E-06	NM_010904	NM_010904	NM_010904	Mm.298283	380684	ENSMUST00000093369
A_51_P339934	Nefl	neurofilament, light polypeptide	1,24	2,37	7,79E-06	3,17E-04	NM_010910	NM_010910	NM_010910	Mm.1956	18039	ENSMUST00000022639
A_51_P145220	Nefm	neurofilament, medium polypeptide	1,29	2,45	4,41E-07	6,97E-05	NM_008691	NM_008691	NM_008691	Mm.390700	18040	ENSMUST00000022638
A_55_P1967500	Nell1	NEL-like 1	1,01	2,02	3,11E-05	7,11E-04	NM_0010379	NM_0010379	NM_0010379	Mm.383528	338352	ENSMUST00000081872
A_55_P2316300	Neo1	neogenin	0,66	1,58	4,99E-05	9,62E-04	NM_008684	NM_008684	NM_008684	Mm.42249	18007	ENSMUST00000068664
A_52_P367520	Nexn	nexilin	2,03	4,09	1,40E-07	4,10E-05	NM_199465	NM_199465	NM_199465	Mm.200188	68810	ENSMUST00000046045
A_55_P2075941	Nf1	neurofibromatosis 1	1,27	2,41	6,05E-05	1,09E-03	NM_010897	NM_010897	NM_010897	Mm.255596	18015	ENSMUST00000071325
A_55_P2173011	Nfasc	neurofascin	0,71	1,64	8,84E-06	3,43E-04	NM_182716	NM_182716	NM_182716	Mm.326702	269116	ENSMUST00000094569
A_51_P270184	Nhlrc2	NHL repeat containing 2	0,71	1,64	1,05E-04	1,56E-03	NM_025811	NM_025811	NM_025811	Mm.46346	66866	ENSMUST00000071423
A_55_P2425190	Nhsl2	NHS-like 2	1,34	2,52	9,86E-07	9,97E-05	NM_00116361	NM_00116361	NM_00116361	Mm.232	100042480	ENSMUST00000101339
A_55_P2084308	Nid1	nidogen 1	1,64	3,11	1,76E-07	4,42E-05	NM_010917	NM_010917	NM_010917	Mm.4691	18073	ENSMUST00000005532
A_51_P315666	Nid2	nidogen 2	0,96	1,95	3,15E-05	7,18E-04	NM_008695	NM_008695	NM_008695	Mm.20348	18074	ENSMUST00000022340
A_51_P479043	Nif3l1	Ngg1 interacting factor 3-like 1 (S. pombe)	0,67	1,59	5,25E-05	9,95E-04	NM_022988	NM_022988	NM_022988	Mm.340248	65102	ENSMUST00000171597
A_51_P122035	Nipa1	non imprinted in Prader-Willi/Angelman syndrome 1 homolog	0,79	1,73	5,51E-05	1,03E-03	NM_153578	NM_153578	NM_153578	Mm.389901	233280	ENSMUST00000052204
A_52_P650180	Nipal2	NIPA-like domain containing 2	0,86	1,82	4,34E-05	8,79E-04	NM_145469	NM_145469	NM_145469	Mm.258402	223473	ENSMUST00000040791
A_55_P2181176	Nipal3	NIPA-like domain containing 3	0,73	1,66	3,43E-05	7,54E-04	NM_028995	NM_028995	NM_028995	Mm.26548	74552	ENSMUST00000105856
A_55_P2121231	Nipbl	Nipped-B homolog (Drosophila)	1,42	2,67	2,86E-06	1,75E-04	NM_201232	NM_201232	NM_201232	Mm.240329	71175	ENSMUST00000052965
A_66_P105947	Nkain3	Na+/K+ transporting ATPase interacting 3	0,88	1,84	1,33E-06	1,15E-04	NM_172987	NM_172987	NM_172987	Mm.489728	269513	ENSMUST00000102998
A_52_P602898	Nkrf	NF-kappaB repressing factor	0,77	1,70	1,34E-04	1,84E-03	NM_029891	NM_029891	NM_029891	Mm.21649	77286	ENSMUST00000057093
A_55_P2012794	Nmbr	neuromedin B receptor	0,79	1,73	3,54E-05	7,69E-04	NM_008703	NM_008703	NM_008703	Mm.487342	18101	ENSMUST00000020015

A_52_P464629	***Nme7***	NME/NM23 family member 7	0,97	1,96	9,13E-06	3,50E-04	NM_138314	NM_138314	NM_138314	Mm.219428	171567	ENSMUST00000086028
A_52_P489119	***Nol6***	nucleolar protein family 6 (RNA-associated)	0,58	1,50	1,16E-04	1,67E-03	NM_139236	NM_139236	NM_139236	Mm.285797	230082	ENSMUST00000030138
A_51_P246705	***Nop14***	NOP14 nucleolar protein	0,67	1,59	1,09E-04	1,60E-03	NM_029278	NM_029278	NM_029278	Mm.40292	75416	ENSMUST00000041364
A_55_P2034033	***Nos3***	nitric oxide synthase 3, endothelial cell	0,71	1,63	3,08E-05	7,09E-04	NM_008713	NM_008713	NM_008713	Mm.258415	18127	ENSMUST00000030834
A_55_P2047168	***Notch1***	notch 1	0,87	1,83	4,10E-05	8,45E-04	NM_008714	NM_008714	NM_008714	Mm.290610	18128	ENSMUST00000028288
A_52_P289213	***Notch2***	notch 2	1,00	2,00	3,32E-06	1,91E-04	NM_010928	NM_010928	NM_010928	Mm.485843	18129	ENSMUST00000079812
A_51_P220162	***Notch3***	notch 3	1,05	2,08	6,22E-06	2,80E-04	NM_008716	NM_008716	NM_008716	Mm.439741	18131	ENSMUST00000087723
A_51_P349341	***Npc1***	Niemann Pick type C1	1,50	2,83	6,09E-07	8,14E-05	NM_008720	NM_008720	NM_008720	Mm.3484	18145	ENSMUST00000025279
A_55_P2111745	***Npcd***	neuronal pentraxin chromo domain	0,52	1,43	1,22E-04	1,73E-03	NM_00101336	NM_00101336	NM_00101336	Mm.38438	504193	ENSMUST00000089299
A_52_P479500	***Npepps***	aminopeptidase puromycin sensitive	1,15	2,21	1,81E-06	1,34E-04	NM_008942	NM_008942	NM_008942	Mm.29824	19155	ENSMUST00000171320
A_55_P1993549	***Npnt***	nephronectin	0,58	1,49	3,73E-05	7,95E-04	NM_033525	NM_033525	NM_033525	Mm.279310	114249	ENSMUST00000042729
A_55_P2076832	***Nrd1***	nardilysin, N-arginine dibasic convertase, NRD convertase 1	0,72	1,65	4,48E-06	2,27E-04	NM_146150	NM_146150	NM_146150	Mm.274950	230598	ENSMUST00000065977
A_51_P213592	***Nrde2***	nrde-2 necessary for RNA interference, domain containing	0,85	1,81	1,35E-04	1,84E-03	NM_183155	NM_183155	NM_183155	Mm.224076	217827	ENSMUST00000021596
A_51_P469285	***Nrp1***	neuropilin 1	0,74	1,67	7,40E-05	1,24E-03	NM_008737	NM_008737	NM_008737	Mm.271745	18186	ENSMUST00000026917
A_52_P93910	***Nrp2***	neuropilin 2	0,91	1,88	9,39E-06	3,55E-04	NM_0010774C	NM_0010774C	NM_0010774C	Mm.266341	18187	ENSMUST00000075144
A_55_P2054628	***Nrxn1***	neurexin I	0,85	1,80	7,14E-06	3,02E-04	NM_020252	NM_020252	NM_020252	Mm.312068	18189	ENSMUST00000176174
A_55_P2054640	***Nrxn3***	neurexin III	1,11	2,15	9,27E-07	9,75E-05	NM_172544	NM_172544	NM_172544	Mm.425766	18191	ENSMUST00000167887
A_66_P137462	***Nsdhl***	NAD(P) dependent steroid dehydrogenase-like	0,66	1,58	8,79E-05	1,39E-03	NM_010941	NM_010941	NM_010941	Mm.38792	18194	ENSMUST00000033715
A_51_P389386	***Nsmce2***	non-SMC element 2 homolog (MMS21, S. cerevisiae)	0,75	1,68	4,25E-05	8,68E-04	NM_026746	NM_026746	NM_026746	Mm.27762	68501	ENSMUST00000079703
A_55_P2143688	***Ntm***	neurotrimin	0,87	1,82	5,37E-05	1,01E-03	NM_172290	NM_172290	NM_172290	Mm.283138	235106	ENSMUST00000115237
A_51_P497661	***Ntn4***	netrin 4	0,72	1,64	2,96E-05	6,91E-04	NM_021320	NM_021320	NM_021320	Mm.483868	57764	ENSMUST00000020204
A_51_P393454	***Ntrk2***	neurotrophic tyrosine kinase, receptor, type 2	1,02	2,03	2,26E-06	1,51E-04	NM_00102507	NM_00102507	NM_00102507	Mm.130054	18212	ENSMUST00000079828
A_55_P2056533	***Ntrk3***	neurotrophic tyrosine kinase, receptor, type 3	1,00	2,00	2,56E-05	6,36E-04	NM_008746	NM_008746	NM_008746	Mm.33496	18213	ENSMUST00000039431
A_55_P2068172	***Nufip2***	nuclear fragile X mental retardation protein interacting protein	0,90	1,87	4,10E-05	8,45E-04	NM_0010242C	NM_0010242C	NM_0010242C	Mm.428996	68564	0
A_66_P128918	***Numbl***	numb-like	0,67	1,59	1,08E-04	1,60E-03	NM_010950	NM_010950	NM_010950	Mm.458153	18223	ENSMUST00000079258
A_52_P286002	***Obsl1***	obscurin-like 1	0,60	1,52	3,51E-05	7,66E-04	NM_178884	NM_178884	NM_178884	Mm.236454	98733	ENSMUST00000127507
A_51_P261107	***Ogt***	O-linked N-acetylglucosamine (GlcNAc) transferase (UDP-N-a	1,43	2,70	2,31E-07	4,92E-05	NM_139144	NM_139144	NM_139144	Mm.259191	108155	ENSMUST00000044475
A_52_P454183	***Olfml2b***	olfactomedin-like 2B	0,84	1,79	3,85E-05	8,11E-04	NM_177068	NM_177068	NM_177068	Mm.287131	320078	ENSMUST00000046792
A_52_P454295	***Olfml2b***	olfactomedin-like 2B	0,48	1,40	1,34E-04	1,84E-03	NM_177068	NM_177068	NM_177068	Mm.287131	320078	ENSMUST00000046792
A_52_P632359	***Olfr1316***	olfactory receptor 1316	1,03	2,04	2,53E-05	6,31E-04	NM_146742	NM_146742	NM_146742	Mm.377665	258737	ENSMUST00000099596
A_51_P499223	***Olfr1354***	olfactory receptor 1354	1,15	2,22	3,12E-06	1,84E-04	NM_00119984	NM_00119984	NM_00119984	Mm.419145	259163	ENSMUST00000075859
A_51_P491856	***Olfr1358***	olfactory receptor 1358	2,06	4,16	8,94E-08	3,18E-05	XM_622784	XM_622784	XM_622784	Mm.380597	258224	0
A_52_P91019	***Olfr1386***	olfactory receptor 1386	2,14	4,41	2,46E-08	1,69E-05	NM_00101174	NM_00101174	NM_00101174	Mm.377286	257888	ENSMUST00000071905
A_51_P476798	***Olfr2***	olfactory receptor 2	0,80	1,74	3,21E-05	7,27E-04	NM_010983	NM_010983	NM_010983	Mm.246525	18317	ENSMUST00000094109
A_52_P242724	***Opa1***	optic atrophy 1	0,80	1,74	7,66E-05	1,27E-03	NM_00119917	NM_00119917	NM_00119917	Mm.274285	74143	ENSMUST00000160597
A_51_P378550	***Oplah***	5-oxoprolinase (ATP-hydrolysing)	0,80	1,74	1,47E-05	4,60E-04	NM_153122	NM_153122	NM_153122	Mm.322738	75475	ENSMUST00000023222
A_55_P2066598	***Oprl1***	opioid receptor-like 1	1,04	2,05	7,66E-05	1,27E-03	NM_011012	NM_011012	NM_011012	Mm.285075	18389	ENSMUST00000108767
A_55_P2066593	***Oprl1***	opioid receptor-like 1	0,74	1,67	9,84E-06	3,65E-04	NM_011012	NM_011012	NM_011012	Mm.285075	18389	ENSMUST00000135535
A_51_P100309	***Oprm1***	opioid receptor, mu 1	0,96	1,95	3,76E-05	7,99E-04	NM_00103965	NM_00103965	NM_00103965	Mm.439715	18390	ENSMUST00000092734
A_51_P109171	***Os9***	amplified in osteosarcoma	0,95	1,93	9,88E-05	1,51E-03	NM_177614	NM_177614	NM_177614	Mm.295246	216440	ENSMUST00000164259
A_55_P2029503	***Osbpl1a***	oxysterol binding protein-like 1A	0,65	1,57	5,81E-05	1,06E-03	NM_207530	NM_207530	NM_207530	Mm.259470	64291	ENSMUST00000132594
A_52_P593015	***Ostc***	oligosaccharyltransferase complex subunit	0,79	1,73	7,44E-05	1,24E-03	NM_025509	NM_025509	NM_025509	Mm.300697	66357	ENSMUST00000043937
A_51_P506733	***P2rx7***	purinergic receptor P2X, ligand-gated ion channel, 7	0,80	1,74	1,02E-04	1,54E-03	NM_00103884	NM_00103884	NM_00103884	Mm.42026	18439	ENSMUST00000031425
A_55_P2117146	***Pa2g4***	proliferation-associated 2G4	0,86	1,82	1,24E-05	4,13E-04	NM_011119	NM_011119	NM_011119	Mm.4742	18813	ENSMUST00000026425
A_66_P115406	***Pabpc1***	poly(A) binding protein, cytoplasmic 1	1,24	2,37	7,85E-07	9,08E-05	NM_008774	NM_008774	NM_008774	Mm.371570	18458	ENSMUST00000146577
A_65_P08881	***Pabpc1l***	poly(A) binding protein, cytoplasmic 1-like	1,48	2,79	2,18E-06	1,49E-04	NM_00111407	NM_00111407	NM_00111407	Mm.82301	381404	ENSMUST00000067715
A_55_P2020607	***Pacsin1***	protein kinase C and casein kinase substrate in neurons 1	0,61	1,52	1,30E-04	1,80E-03	NM_011861	NM_011861	NM_011861	Mm.4926	23969	ENSMUST00000045896
A_55_P1992769	***Pafah1b1***	platelet-activating factor acetylhydrolase, isoform 1b, subunit '	0,67	1,59	1,47E-04	1,95E-03	NM_013625	NM_013625	NM_013625	Mm.397111	18472	ENSMUST00000021091
A_51_P172323	***Pak2***	p21 protein (Cdc42/Rac)-activated kinase 2	0,99	1,98	1,69E-06	1,30E-04	NM_177326	NM_177326	NM_177326	Mm.234204	224105	ENSMUST00000023467
A_55_P1977653	***Palld***	palladin, cytoskeletal associated protein	0,80	1,75	4,79E-05	9,42E-04	NM_0010813S	NM_0010813S	NM_0010813S	Mm.29933	72333	ENSMUST00000034057
A_51_P196127	***Papss1***	3'-phosphoadenosine 5'-phosphosulfate synthase 1	1,15	2,21	8,34E-06	3,31E-04	NM_011863	NM_011863	NM_011863	Mm.244912	23971	ENSMUST00000029666
A_51_P430973	***Paqr7***	progestin and adipoQ receptor family member VII	0,53	1,45	1,00E-04	1,52E-03	NM_027995	NM_027995	NM_027995	Mm.142343	71904	ENSMUST00000081525
A_52_P51548	***Pard3***	par-3 (partitioning defective 3) homolog (C. elegans)	0,85	1,81	1,52E-04	1,99E-03	NM_033620	NM_033620	NM_033620	Mm.299254	93742	ENSMUST00000162309
A_52_P247943	***Parl***	presenilin associated, rhomboid-like	0,55	1,46	1,44E-04	1,93E-03	NM_0010057€	NM_0010057€	NM_0010057€	Mm.371737	381038	ENSMUST00000152887
A_51_P314277	***Parp1***	poly (ADP-ribose) polymerase family, member 1	0,81	1,75	1,95E-05	5,42E-04	NM_007415	NM_007415	NM_007415	Mm.277779	11545	ENSMUST00000027777
A_51_P343913	***Parp4***	poly (ADP-ribose) polymerase family, member 4	0,82	1,76	1,31E-05	4,29E-04	NM_00114597	NM_00114597	NM_00114597	Mm.379357	328417	ENSMUST00000161553
A_55_P2042272	***Pbrm1***	polybromo 1	1,08	2,11	8,70E-05	1,38E-03	AK166588	0	AK166588	Mm.27913	66923	0
A_55_P2051696	***Pbx1***	pre B cell leukemia homeobox 1	0,91	1,88	3,41E-05	7,52E-04	NM_008783	NM_008783	NM_008783	Mm.43358	18514	ENSMUST00000072863
A_51_P484254	***Pcca***	propionyl-Coenzyme A carboxylase, alpha polypeptide	0,90	1,87	6,48E-06	2,85E-04	NM_144844	NM_144844	NM_144844	Mm.23876	110821	ENSMUST00000038374
A_66_P130887	***Pcdh18***	protocadherin 18	0,83	1,77	1,50E-04	1,98E-03	NM_130448	NM_130448	NM_130448	Mm.87246	73173	ENSMUST00000035931
A_52_P432969	***Pcdh19***	protocadherin 19	0,67	1,59	6,80E-05	1,17E-03	NM_00110524	NM_00110524	NM_00110524	Mm.39738	279653	ENSMUST00000149154
A_55_P2035167	***Pcdh7***	protocadherin 7	1,29	2,45	8,90E-06	3,44E-04	NM_00112275	NM_00112275	NM_00112275	Mm.332387	54216	0
A_51_P274488	***Pcdh8***	protocadherin 8	1,60	3,04	1,84E-07	4,50E-05	NM_021543	NM_021543	NM_021543	Mm.390715	18530	ENSMUST00000039568
A_51_P476654	***Pcdha11***	protocadherin alpha 11	1,10	2,15	4,68E-06	2,33E-04	NM_009960	NM_009960	NM_009960	Mm.308500	12942	ENSMUST00000115657
A_55_P2006632	***Pcdha12***	protocadherin alpha 12	0,91	1,88	1,57E-06	1,24E-04	NM_138663	NM_138663	NM_138663	Mm.308500	192164	ENSMUST00000047614
A_51_P388048	***Pcdha4***	protocadherin alpha 4	0,87	1,83	1,05E-05	3,77E-04	NM_007766	NM_007766	NM_007766	Mm.308500	12936	ENSMUST00000115661
A_55_P2106584	***Pcdha8***	protocadherin alpha 8	1,20	2,29	2,69E-06	1,67E-04	NM_201243	NM_201243	NM_201243	Mm.308500	353235	ENSMUST00000115661
A_55_P2023306	***Pcdha9***	protocadherin alpha 9	0,83	1,78	4,87E-05	9,49E-04	NM_138661	NM_138661	NM_138661	Mm.308500	192161	ENSMUST00000115659
A_55_P2006630	***Pcdhac1***	protocadherin alpha subfamily C, 1	0,55	1,46	1,33E-04	1,83E-03	NM_00100367	NM_00100367	NM_00100367	Mm.308500	353236	ENSMUST00000007584
A_55_P2471752	***Pcdhac2***	protocadherin alpha subfamily C, 2	0,86	1,82	1,05E-04	1,56E-03	NM_00100367	NM_00100367	NM_00100367	Mm.308500	353237	ENSMUST00000047479
A_55_P2008634	***Pcdhb10***	protocadherin beta 10	0,87	1,83	2,03E-05	5,56E-04	NM_053135	NM_053135	NM_053135	Mm.115996	93881	ENSMUST00000051126
A_51_P240421	***Pcdhb14***	protocadherin beta 14	0,99	1,99	1,67E-05	4,93E-04	NM_053139	NM_053139	NM_053139	Mm.196699	93885	ENSMUST00000052387
A_51_P159833	***Pcdhb15***	protocadherin beta 15	0,97	1,96	1,74E-05	5,06E-04	NM_053140	NM_053140	NM_053140	Mm.283857	93886	ENSMUST00000050034
A_55_P2072522	***Pcdhb16***	protocadherin beta 16	1,20	2,30	2,15E-06	1,47E-04	NM_053141	NM_053141	NM_053141	Mm.197441	93887	ENSMUST00000051442
A_51_P317181	***Pcdhb21***	protocadherin beta 21	0,94	1,92	4,39E-06	2,24E-04	NM_053146	NM_053146	NM_053146	Mm.348053	93892	ENSMUST00000061405
A_51_P109295	***Pcdhb3***	protocadherin beta 3	0,68	1,60	1,03E-04	1,54E-03	NM_053128	NM_053128	NM_053128	Mm.82308	93874	ENSMUST00000051754
A_51_P403413	***Pcdhb6***	protocadherin beta 6	0,83	1,77	1,88E-05	5,30E-04	NM_053131	NM_053131	NM_053131	Mm.450193	93877	ENSMUST00000061717
A_51_P302206	***Pcdhb8***	protocadherin beta 8	1,24	2,36	1,82E-06	1,35E-04	NM_053133	NM_053133	NM_053133	Mm.348052	93879	ENSMUST00000051163
A_55_P2441059	***Pcdhga1***	protocadherin gamma subfamily A, 1	1,07	2,10	3,30E-06	1,90E-04	NM_033584	NM_033584	NM_033584	Mm.247203	93709	0
A_55_P2094792	***Pcdhga10***	protocadherin gamma subfamily A, 10	0,96	1,95	1,11E-05	3,88E-04	NM_033593	NM_033593	NM_033593	Mm.247203	93722	0
A_55_P2447154	***Pcdhga5***	protocadherin gamma subfamily A, 5	1,22	2,33	1,17E-06	1,08E-04	NM_033588	NM_033588	NM_033588	Mm.247203	93713	0
A_55_P2067719	***Pcdhgb1***	protocadherin gamma subfamily B, 1	0,87	1,83	3,72E-05	7,94E-04	NM_033574	NM_033574	NM_033574	Mm.247203	93699	0
A_55_P2250164	***Pcdhgc5***	protocadherin gamma subfamily C, 5	0,72	1,64	1,74E-05	5,05E-04	NM_033583	NM_033583	NM_033583	Mm.247203	93708	ENSMUST00000055935
A_55_P2014750	***Pclo***	piccolo (presynaptic cytomatrix protein)	2,31	4,97	2,05E-08	1,63E-05	NM_011995	NM_011995	NM_011995	Mm.146275	26875	ENSMUST00000030691
A_52_P89425	***Pcnt***	pericentrin (kendrin)	1,20	2,30	3,77E-06	2,07E-04	NM_008787	NM_008787	NM_008787	Mm.251794	18541	ENSMUST00000001179
A_55_P2000384	***Pcnxl3***	pecanex-like 3 (Drosophila)	0,50	1,42	1,36E-04	1,86E-03	NM_144868	NM_144868	NM_144868	Mm.329650	104401	ENSMUST00000068169
A_52_P500979	***Pcsk1***	proprotein convertase subtilisin/kexin type 1	1,14	2,21	8,26E-07	9,30E-05	NM_013628	NM_013628	NM_013628	Mm.1333	18548	ENSMUST00000022075
A_51_P229613	***Pcsk2***	proprotein convertase subtilisin/kexin type 2	1,17	2,25	3,72E-06	2,05E-04	NM_008792	NM_008792	NM_008792	Mm.294493	18549	ENSMUST00000028905
A_52_P544043	***Pcsk5***	proprotein convertase subtilisin/kexin type 5	1,16	2,24	1,76E-06	1,32E-04	NM_00116314	NM_00116314	NM_00116314	Mm.3401	18552	ENSMUST00000050715
A_51_P397673	***Pcsk9***	proprotein convertase subtilisin/kexin type 9	0,77	1,70	1,08E-04	1,60E-03	NM_153565	NM_153565	NM_153565	Mm.133268	100102	ENSMUST00000049507
A_51_P407594	***Pdcd11***	programmed cell death 11	1,32	2,50	1,93E-06	1,39E-04	NM_011053	NM_011053	NM_011053	Mm.41166	18572	ENSMUST00000072141
A_51_P419300	***Pdcd2l***	programmed cell death 2-like	0,65	1,56	6,04E-05	1,09E-03	NM_026549	NM_026549	NM_026549	Mm.38173	68079	ENSMUST00000002710
A_52_P452787	***Pdcd6ip***	programmed cell death 6 interacting protein	1,16	2,24	6,05E-06	2,75E-04	NM_011052	NM_011052	NM_011052	Mm.29816	18571	ENSMUST00000137104
A_55_P2071402	***Pde3a***	phosphodiesterase 3A, cGMP inhibited	0,76	1,69	1,13E-04	1,64E-03	NM_018779	NM_018779	NM_018779	Mm.485878	54611	ENSMUST00000043259
A_65_P12530	***Pde4d***	phosphodiesterase 4D, cAMP specific	0,75	1,68	4,01E-05	8,35E-04	NM_011056	NM_011056	NM_011056	Mm.434429	238871	ENSMUST00000099179
A_55_P1984173	***Pde4dip***	phosphodiesterase 4D interacting protein (myomegalin)	1,04	2,05	8,42E-06	3,32E-04	NM_178080	NM_178080	NM_178080	Mm.129840	83679	ENSMUST00000045243
A_51_P345649	***Pdgfra***	platelet derived growth factor receptor, alpha polypeptide	1,30	2,47	7,03E-06	2,99E-04	NM_011058	NM_011058	NM_011058	Mm.221403	18595	ENSMUST00000168162
A_55_P1969776	***Pdhb***	pyruvate dehydrogenase (lipoamide) beta	0,66	1,58	1,30E-04	1,81E-03	NM_024221	NM_024221	NM_024221	Mm.301527	68263	ENSMUST00000022268
A_51_P328300	***Pdia4***	protein disulfide isomerase associated 4	1,16	2,23	9,23E-06	3,52E-04	NM_009787	NM_009787	NM_009787	Mm.2442	12304	ENSMUST00000077290
A_51_P472726	***Pdlim2***	PDZ and LIM domain 2	1,64	3,11	2,69E-06	1,67E-04	NM_145978	NM_145978	NM_145978	Mm.283968	213019	ENSMUST00000129174
A_55_P2162394	***Pdp1***	pyruvate dehyrogenase phosphatase catalytic subunit 1	0,83	1,78	9,75E-05	1,49E-03	NM_00109823	NM_00109823	NM_00109823	Mm.331489	381511	ENSMUST00000056050
A_66_P111285	***Pdp2***	pyruvate dehyrogenase phosphatase catalytic subunit 2	0,91	1,88	7,16E-06	3,03E-04	NM_0010246C	NM_0010246C	NM_0010246C	Mm.290750	382051	ENSMUST00000059588
A_66_P123898	***Pdyn***	prodynorphin	0,67	1,59	4,03E-05	8,38E-04	NM_018863	NM_018863	NM_018863	Mm.6239	18610	ENSMUST00000028883
A_55_P2062549	***Pdzrn3***	PDZ domain containing RING finger 3	0,58	1,49	1,38E-04	1,87E-03	NM_018884	NM_018884	NM_018884	Mm.321654	55983	ENSMUST00000075994
A_52_P987201	***Pdzrn4***	PDZ domain containing RING finger 4	1,22	2,34	2,92E-07	5,83E-05	NM_00116459	NM_00116459	NM_00116459	Mm.309826	239618	ENSMUST00000169942
A_52_P377723	***Peg10***	paternally expressed 10	2,28	4,84	1,93E-07	4,60E-05	NM_00104061	NM_00104061	NM_00104061	Mm.320575	170676	ENSMUST00000166678
A_55_P2163319	***Peg3***	paternally expressed 3	1,84	3,59	8,89E-07	9,65E-05	NM_008817	NM_008817	NM_008817	Mm.389800	18616	ENSMUST00000051209
A_52_P546552	***Pelp1***	proline, glutamic acid and leucine rich protein 1	0,88	1,84	1,18E-05	4,01E-04	NM_029231	NM_029231	NM_029231	Mm.340601	75273	ENSMUST00000019065
A_51_P282760	***Per2***	period circadian clock 2	1,02	2,02	2,21E-05	5,85E-04	NM_011066	NM_011066	NM_011066	Mm.482463	18627	ENSMUST00000069620
A_55_P2023094	***Pes1***	pescadillo homolog 1, containing BRCT domain (zebrafish)	0,88	1,84	3,92E-05	8,21E-04	NM_022889	NM_022889	NM_022889	Mm.28659	64934	ENSMUST00000020705

A_52_P599624	Pfas	phosphoribosylformylglycinamidine synthase (FGAR amidotra	1,03	2,05	3,78E-06	2,07E-04	NM_00115951	NM_00115951	NM_00115951	Mm.340288	237823	ENSMUST00000172915
A_55_P1963184	Pgap1	post-GPI attachment to proteins 1	1,13	2,19	8,92E-06	3,45E-04	NM_00116331	NM_00116331	NM_00116331	Mm.103539	241062	ENSMUST00000097739
A_66_P111747	Pgk1	phosphoglycerate kinase 1	0,80	1,74	1,44E-04	1,93E-03	ENSMUST000	0	0	0	18655		ENSMUST00000085735
A_51_P213476	Pgr	progesterone receptor	0,79	1,73	4,07E-05	8,42E-04	NM_008829	NM_008829	NM_008829	Mm.12798	18667	ENSMUST00000070463
A_55_P2127238	Phc3	polyhomeotic-like 3 (Drosophila)	1,15	2,22	4,73E-06	2,34E-04	ENSMUST000	0	AK166296	Mm.233173	241915	ENSMUST00000099163
A_55_P2081560	Phf20	PHD finger protein 20	0,72	1,65	8,20E-06	3,28E-04	NM_172674	NM_172674	NM_172674	Mm.427078	228829	ENSMUST00000152617
A_52_P587648	Phf3	PHD finger protein 3	0,74	1,67	6,65E-05	1,16E-03	NM_00108108	NM_00108108	NM_00108108	Mm.194486	213109	ENSMUST00000088310
A_51_P258698	Phf8	PHD finger protein 8	1,11	2,15	3,21E-06	1,87E-04	NM_00111335	NM_00111335	NM_00111335	Mm.17156	320595	ENSMUST00000168501
A_55_P2092310	Phgdh	3-phosphoglycerate dehydrogenase	0,51	1,42	9,12E-05	1,42E-03	AK195427	0	AK195427	Mm.371997	236539	0
A_55_P1972192	Phka1	phosphorylase kinase alpha 1	0,87	1,82	9,68E-07	9,88E-05	NM_008832	NM_008832	NM_008832	Mm.212889	18679	ENSMUST00000113611
A_52_P187217	Phka2	phosphorylase kinase alpha 2	1,15	2,22	1,37E-05	4,41E-04	NM_172783	NM_172783	NM_172783	Mm.350712	110094	ENSMUST00000033652
A_52_P513347	Phkb	phosphorylase kinase beta	0,83	1,78	3,41E-05	7,53E-04	NM_199446	NM_199446	NM_199446	Mm.237296	102093	ENSMUST00000162305
A_55_P1981155	Phldb1	pleckstrin homology-like domain, family B, member 1	1,07	2,10	1,08E-06	1,03E-04	NM_153537	NM_153537	NM_153537	Mm.28639	102693	ENSMUST00000138356
A_55_P2090055	Phrf1	PHD and ring finger domains 1	0,73	1,66	2,80E-05	6,71E-04	NM_00108111	NM_00108111	NM_00108111	Mm.248656	101471	ENSMUST00000106027
A_52_P454994	Pi4ka	phosphatidylinositol 4-kinase, catalytic, alpha polypeptide	1,32	2,50	9,31E-07	9,77E-05	NM_00100198	NM_00100198	NM_00100198	Mm.5718	224020	ENSMUST00000148110
A_52_P607195	Pigh	phosphatidylinositol glycan anchor biosynthesis, class H	0,65	1,57	6,72E-05	1,16E-03	NM_029988	NM_029988	NM_029988	Mm.268317	110417	ENSMUST00000072154
A_51_P197175	Pigk	phosphatidylinositol glycan anchor biosynthesis, class K	0,87	1,83	7,69E-06	3,14E-04	NM_178016	NM_178016	NM_178016	Mm.331447	329777	ENSMUST00000051510
A_55_P2006911	Pik3c3	phosphoinositide-3-kinase, class 3	1,10	2,14	1,44E-06	1,19E-04	NM_181414	NM_181414	NM_181414	Mm.194127	225326	ENSMUST00000131405
A_51_P110035	Pik3r4	phosphatidylinositol 3 kinase, regulatory subunit, polypeptide	1,22	2,33	1,19E-06	1,09E-04	NM_00108130	NM_00108130	NM_00108130	Mm.274830	75669	ENSMUST00000065778
A_51_P393355	Pikfyve	phosphoinositide kinase, FYVE finger containing	0,90	1,86	2,64E-05	6,49E-04	NM_011086	NM_011086	NM_011086	Mm.38370	18711	ENSMUST00000097707
A_55_P2037156	Pja1	praja1, RING-H2 motif containing	0,77	1,71	1,17E-04	1,68E-03	NM_00108311	NM_00108311	NM_00108311	Mm.8211	18744	ENSMUST00000113797
A_52_P420712	Pja2	praja 2, RING-H2 motif containing	1,96	3,90	9,55E-07	9,82E-05	NM_00102530	NM_00102530	NM_00102530	Mm.489681	224938	ENSMUST00000172733
A_52_P234729	Pkd2	polycystic kidney disease 2	0,56	1,48	1,23E-04	1,74E-03	NM_008861	NM_008861	NM_008861	Mm.6442	18764	ENSMUST00000086831
A_51_P228892	Pkd2l2	polycystic kidney disease 2-like 2	0,75	1,68	1,15E-04	1,66E-03	NM_016927	NM_016927	NM_016927	Mm.445792	53871	ENSMUST00000014647
A_52_P516091	Pla2g15	phospholipase A2, group XV	0,78	1,72	3,92E-06	3,67E-04	NM_133792	NM_133792	NM_133792	Mm.284770	192654	ENSMUST00000034377
A_66_P114784	Pla2g7	phospholipase A2, group VII (platelet-activating factor acetylh	1,36	2,56	1,03E-06	1,02E-04	NM_013737	NM_013737	NM_013737	Mm.9277	27226	ENSMUST00000167418
A_55_P2173333	Plcb4	phospholipase C, beta 4	0,58	1,49	8,96E-05	1,41E-03	ENSMUST000	0	BC051068	Mm.38009	18798	ENSMUST00000110109
A_52_P327588	Plcd4	phospholipase C, delta 4	1,30	2,46	6,94E-07	8,60E-05	NM_148937	NM_148937	NM_148937	Mm.290731	18802	ENSMUST00000152707
A_55_P2015860	Plcg1	phospholipase C, gamma 1	1,15	2,23	9,75E-06	3,63E-04	NM_021280	NM_021280	NM_021280	Mm.44463	18803	ENSMUST00000017077
A_51_P279163	Plcg2	phospholipase C, gamma 2	0,95	1,93	1,69E-06	1,30E-04	NM_172285	NM_172285	NM_172285	Mm.192699	234779	ENSMUST00000081232
A_55_P2139027	Plec	plectin	1,22	2,33	7,41E-07	8,79E-05	NM_00116354	NM_00116354	NM_00116354	Mm.234912	18810	ENSMUST00000073418
A_55_P1968718	Plekha6	pleckstrin homology domain containing, family A member 6	1,05	2,07	1,17E-05	3,99E-04	NM_182930	NM_182930	NM_182930	Mm.253559	240753	ENSMUST00000038295
A_55_P2165560	Plekhg5	pleckstrin homology domain containing, family G (with RhoGe	0,62	1,54	1,23E-04	1,73E-03	NM_00100415	NM_00100415	NM_00100415	Mm.486442	269608	ENSMUST00000105661
A_55_P1968340	Plekhh1	pleckstrin homology domain containing, family H (with MyTH4	0,74	1,67	2,45E-05	6,19E-04	NM_181073	NM_181073	NM_181073	Mm.478321	211945	ENSMUST00000039928
A_55_P2169784	Plod1	procollagen-lysine, 2-oxoglutarate 5-dioxygenase 1	1,38	2,60	6,80E-07	8,57E-05	NM_011122	NM_011122	NM_011122	Mm.37371	18822	ENSMUST00000019199
A_51_P396570	Plod2	procollagen lysine, 2-oxoglutarate 5-dioxygenase 2	0,56	1,48	6,49E-05	1,14E-03	NM_011961	NM_011961	NM_011961	Mm.79983	26432	ENSMUST00000070522
A_55_P2168098	Plrg1	pleiotropic regulator 1, PRL1 homolog (Arabidopsis)	0,50	1,42	6,55E-05	1,15E-03	NM_016784	NM_016784	NM_016784	Mm.286349	53317	ENSMUST00000029628
A_55_P2141851	Pls3	plastin 3 (T-isoform)	1,00	2,00	4,03E-06	2,14E-04	NM_145629	NM_145629	NM_145629	Mm.28777	102866	ENSMUST00000033547
A_51_P232748	Plxnb3	plexin B3	1,17	2,25	1,43E-06	1,19E-04	NM_019587	NM_019587	NM_019587	Mm.275600	140571	ENSMUST00000149478
A_52_P642836	Pnmal2	PNMA-like 2	1,10	2,15	4,03E-06	2,14E-04	NM_00109963	NM_00109963	NM_00109963	Mm.483018	434128	ENSMUST00000094807
A_66_P125831	Pnn	pinin	1,06	2,08	9,16E-07	9,70E-05	NM_008891	NM_008891	NM_008891	Mm.22347	18949	ENSMUST00000021381
A_55_P2018841	Pnp	purine-nucleoside phosphorylase	0,73	1,65	6,19E-05	1,10E-03	NM_013632	NM_013632	NM_013632	Mm.17932	18950	ENSMUST00000048615
A_55_P1985219	Pnpla6	patatin-like phospholipase domain containing 6	0,80	1,74	1,12E-05	3,91E-04	NM_00112281	NM_00112281	NM_00112281	Mm.23085	50767	ENSMUST00000111070
A_55_P2114347	Pnpla8	patatin-like phospholipase domain containing 8	0,59	1,51	1,05E-04	1,56E-03	NM_026164	NM_026164	NM_026164	Mm.54126	67452	0
A_55_P1952235	Pnpo	pyridoxine 5'-phosphate oxidase	0,53	1,45	1,22E-04	1,73E-03	NM_134021	NM_134021	NM_134021	Mm.254704	103711	ENSMUST00000153896
A_55_P1975110	Pnpt1	polyribonucleotide nucleotidyltransferase 1	1,11	2,16	9,82E-07	9,96E-05	NM_027869	NM_027869	NM_027869	Mm.211131	71701	ENSMUST00000154924
A_51_P513449	Pogz	pogo transposable element with ZNF domain	0,99	1,99	1,22E-06	1,10E-04	NM_172683	NM_172683	NM_172683	Mm.274787	229584	ENSMUST00000107270
A_51_P483261	Pomt1	protein-O-mannosyltransferase 1	0,97	1,96	3,43E-06	1,94E-04	NM_145145	NM_145145	NM_145145	Mm.31192	99011	ENSMUST00000129163
A_52_P126055	Pon2	paraoxonase 2	0,85	1,81	1,35E-05	4,35E-04	NM_183308	NM_183308	NM_183308	Mm.126984	330260	ENSMUST00000057792
A_55_P1954086	Postn	periostin, osteoblast specific factor	0,97	1,96	1,24E-06	1,10E-04	NM_00119876	NM_00119876	NM_00119876	Mm.236067	50706	ENSMUST00000107985
A_66_P139785	Ppap2b	phosphatidic acid phosphatase type 2B	0,82	1,77	2,91E-05	6,85E-04	NM_080555	NM_080555	NM_080555	Mm.348326	67916	ENSMUST00000064139
A_52_P659258	Ppat	phosphoribosyl pyrophosphate amidotransferase	1,04	2,06	1,34E-05	4,33E-04	NM_172146	NM_172146	NM_172146	Mm.202337	231327	ENSMUST00000031158
A_51_P392377	Ppfia2	protein tyrosine phosphatase, receptor type, f polypeptide (PT	0,99	1,99	9,64E-06	3,61E-04	NM_177373	NM_177373	NM_177373	Mm.391424	327814	ENSMUST00000029404
A_55_P1998374	Ppfibp1	PTPRF interacting protein, binding protein 1 (liprin beta 1)	1,64	3,12	2,27E-07	4,92E-05	NM_00117043	NM_00117043	NM_00117043	Mm.103382	67533	ENSMUST00000016631
A_55_P2016249	Ppid	peptidylprolyl isomerase D (cyclophilin D)	1,46	2,75	4,33E-05	8,79E-04	NM_026352	NM_026352	NM_026352	Mm.295252	67738	ENSMUST00000161460
A_52_P607683	Ppm1e	protein phosphatase 1E (PP2C domain containing)	0,97	1,96	1,73E-05	5,04E-04	NM_177167	NM_177167	NM_177167	Mm.341988	320472	ENSMUST00000055438
A_51_P484832	Ppm1g	protein phosphatase 1G (formerly 2C), magnesium-dependen	1,44	2,71	1,20E-06	1,09E-04	NM_008014	NM_008014	NM_008014	Mm.14501	14208	ENSMUST00000031032
A_51_P265219	Ppm1k	protein phosphatase 1K (PP2C domain containing)	0,95	1,93	2,68E-05	6,72E-04	NM_175523	NM_175523	NM_175523	Mm.489618	243382	ENSMUST00000042766
A_52_P540434	Ppp1cc	protein phosphatase 1, catalytic subunit, gamma isoform	0,91	1,88	3,29E-06	1,90E-04	NM_013636	NM_013636	NM_013636	Mm.280784	19047	ENSMUST00000134719
A_52_P116372	Ppp1r15b	protein phosphatase 1, regulatory (inhibitor) subunit 15b	0,76	1,69	1,95E-05	5,43E-04	NM_133819	NM_133819	NM_133819	Mm.293628	108954	ENSMUST00000052529
A_52_P239536	Ppp1r9a	protein phosphatase 1, regulatory (inhibitor) subunit 9A	4,15	17,75	5,08E-10	4,08E-06	NM_181595	NM_181595	NM_181595	Mm.332901	243725	ENSMUST00000035813
A_55_P1967315	Ppp1r9b	protein phosphatase 1, regulatory subunit 9B	2,53	5,78	5,72E-09	8,90E-06	NM_172261	NM_172261	NM_172261	Mm.490322	217124	ENSMUST00000038696
A_55_P2053783	Ppp2r3a	protein phosphatase 2, regulatory subunit B'', alpha	1,09	2,13	5,92E-05	1,08E-03	NM_00116136	NM_00116136	NM_00116136	Mm.335386	235542	ENSMUST00000075941
A_52_P487362	Ppp4r4	protein phosphatase 4, regulatory subunit 4	1,02	2,03	2,48E-05	6,23E-04	NM_028980	NM_028980	NM_028980	Mm.248619	74521	ENSMUST00000021631
A_55_P2141415	Ppp6r3	protein phosphatase 6, regulatory subunit 3	1,12	2,18	6,32E-06	2,82E-04	AK018652	0	AK018652	Mm.284686	52036	0
A_55_P1991693	Pprc1	peroxisome proliferative activated receptor, gamma, coactivat	0,91	1,88	6,41E-06	2,84E-04	NM_00108121	NM_00108121	NM_00108121	Mm.2415	226169	0
A_55_P2082361	Ppt1	palmitoyl-protein thioesterase 1	1,00	2,00	3,98E-06	2,13E-04	NM_008917	NM_008917	NM_008917	Mm.277719	19063	ENSMUST00000030412
A_51_P123604	Ppwd1	peptidylprolyl isomerase domain and WD repeat containing 1	0,75	1,68	3,86E-05	8,13E-04	NM_172807	NM_172807	NM_172807	Mm.98910	238831	ENSMUST00000022226
A_55_P1988083	Prc1	protein regulator of cytokinesis 1	0,98	1,98	1,05E-04	1,56E-03	NM_145150	NM_145150	NM_145150	Mm.227274	233406	ENSMUST00000163812
A_55_P1979929	Prcp	prolylcarboxypeptidase (angiotensinase C)	0,68	1,61	5,94E-05	1,08E-03	NM_028243	NM_028243	NM_028243	Mm.389969	72461	ENSMUST00000076052
A_55_P1979457	Prdx4	peroxiredoxin 4	0,59	1,50	1,39E-04	1,88E-03	NM_016764	NM_016764	NM_016764	Mm.247542	53381	ENSMUST00000026328
A_52_P90124	Prep	prolyl endopeptidase	0,69	1,62	6,68E-05	1,16E-03	NM_011156	NM_011156	NM_011156	Mm.37294	19072	ENSMUST00000099858
A_55_P2072661	Prex1	phosphatidylinositol-3,4,5-trisphosphate-dependent Rac exch	0,68	1,60	4,26E-05	8,68E-04	NM_177782	NM_177782	NM_177782	Mm.489669	277360	ENSMUST00000036719
A_55_P1954758	Prickle1	prickle homolog 1 (Drosophila)	0,80	1,74	2,68E-05	6,53E-04	NM_00103321	NM_00103321	NM_00103321	Mm.150314	106042	ENSMUST00000109255
A_55_P2020786	Prkar1a	protein kinase, cAMP dependent regulatory, type I, alpha	1,47	2,77	2,15E-05	5,77E-04	NM_021880	NM_021880	NM_021880	Mm.30039	19084	ENSMUST00000106677
A_55_P1961436	Prkca	protein kinase C, alpha	0,94	1,92	3,08E-05	7,09E-04	NM_011101	NM_011101	NM_011101	Mm.222178	18750	ENSMUST00000100302
A_55_P1976282	Prkcg	protein kinase C, gamma	1,01	2,01	2,18E-05	5,82E-04	NM_011102	NM_011102	NM_011102	Mm.7980	18752	ENSMUST00000100301
A_51_P106397	Prkd3	protein kinase D3	1,01	2,01	2,87E-05	6,79E-04	NM_029239	NM_029239	NM_029239	Mm.252776	75292	ENSMUST00000168887
A_51_P241667	Prkdc	protein kinase, DNA activated, catalytic polypeptide	1,73	3,31	3,07E-07	5,95E-05	NM_011159	NM_011159	NM_011159	Mm.71	19090	ENSMUST00000023352
A_52_P405193	Prkrir	protein-kinase, interferon-inducible double stranded RNA depe	0,86	1,82	9,59E-06	3,59E-04	NM_028410	NM_028410	NM_028410	Mm.4428	72981	ENSMUST00000033009
A_51_P389539	Prmt1	protein arginine N-methyltransferase 1	0,77	1,70	9,52E-06	3,58E-04	NM_019830	NM_019830	NM_019830	Mm.27545	15469	ENSMUST00000153576
A_55_P1996374	Prmt10	protein arginine methyltransferase 10 (putative)	0,95	1,93	6,12E-05	1,10E-03	NM_00108124	NM_00108124	NM_00108124	Mm.197630	102182	ENSMUST00000118622
A_51_P483280	Prnp	prion protein	0,75	1,69	9,77E-06	3,63E-04	NM_011170	NM_011170	NM_011170	Mm.648	19122	ENSMUST00000091288
A_55_P2076219	Prokr2	prokineticin receptor 2	1,01	2,02	1,96E-05	5,44E-04	NM_144944	NM_144944	NM_144944	Mm.283777	246313	ENSMUST00000049997
A_51_P393426	Pros1	protein S (alpha)	0,80	1,74	1,97E-05	5,45E-04	NM_011173	NM_011173	NM_011173	Mm.127156	19128	ENSMUST00000023629
A_55_P2148753	Proser1	proline and serine rich 1	1,25	2,37	6,27E-07	8,24E-05	NM_173382	NM_173382	NM_173382	Mm.218697	212127	ENSMUST00000141418
A_55_P2048751	Prox1	prospero-related homeobox 1	1,31	2,48	2,29E-05	5,94E-04	NM_008937	NM_008937	NM_008937	Mm.132579	19130	ENSMUST00000175916
A_52_P672453	Prpf3	PRP3 pre-mRNA processing factor 3 homolog (yeast)	0,84	1,80	1,12E-05	3,91E-04	NM_027541	NM_027541	NM_027541	Mm.279872	70767	ENSMUST00000161476
A_55_P2132472	Prpf4b	PRP4 pre-mRNA processing factor 4 homolog B (yeast)	0,90	1,87	1,63E-05	4,31E-04	NM_013830	NM_013830	NM_013830	Mm.10027	19134	ENSMUST00000077853
A_55_P1997751	Prpf8	pre-mRNA processing factor 8	2,77	6,83	1,32E-09	5,15E-06	NM_138659	NM_138659	NM_138659	Mm.3757	192159	ENSMUST00000018449
A_52_P257472	Prr12	proline rich 12	1,44	2,72	8,97E-07	9,66E-05	NM_175022	NM_175022	NM_175022	Mm.219137	233210	ENSMUST00000057293
A_55_P2432388	Prr14l	proline rich 14-like	1,03	2,05	2,26E-05	5,91E-04	NM_194340	NM_194340	NM_194340	Mm.248721	215476	ENSMUST00000120129
A_66_P126552	Prr14l	proline rich 14-like	0,69	1,61	2,16E-05	5,79E-04	NM_194340	NM_194340	NM_194340	Mm.248721	215476	ENSMUST00000120129
A_51_P485651	Prrc2a	proline-rich coiled-coil 2A	0,74	1,67	1,27E-04	1,78E-03	NM_020027	NM_020027	NM_020027	Mm.272462	53761	ENSMUST00000173558
A_55_P1994477	Prrc2c	proline-rich coiled-coil 2C	1,29	2,44	9,75E-06	3,63E-04	NM_00108129	NM_00108129	NM_00108129	Mm.245446	226562	0
A_51_P363338	Prss41	protease, serine, 41	0,60	1,51	8,95E-05	1,41E-03	NM_027644	NM_027644	NM_027644	Mm.328839	71003	ENSMUST00000024926
A_55_P2042101	Prune2	prune homolog 2 (Drosophila)	1,56	2,96	2,10E-08	1,63E-05	NM_181348	NM_181348	NM_181348	Mm.489758	353211	ENSMUST00000087689
A_66_P121495	Psat1	phosphoserine aminotransferase 1	0,92	1,89	1,19E-05	4,02E-04	NM_00120533	NM_00120533	NM_00120533	Mm.289936	107272	ENSMUST00000025542
A_55_P2138177	Psd3	pleckstrin and Sec7 domain containing 3	0,92	1,89	1,12E-04	1,63E-03	NM_030263	NM_030263	NM_030263	Mm.32525	234353	ENSMUST00000038959
A_55_P1952127	Psma3	proteasome (prosome, macropain) subunit, alpha type 3	0,76	1,69	8,08E-05	1,31E-03	NM_011184	NM_011184	NM_011184	Mm.296338	19167	ENSMUST00000162851
A_52_P81468	Psma4	proteasome (prosome, macropain) subunit, alpha type 4	0,95	1,93	2,87E-06	1,75E-04	NM_011966	NM_011966	NM_011966	Mm.30270	26441	ENSMUST00000171900
A_55_P2176345	Psma5	proteasome (prosome, macropain) subunit, alpha type 5	0,73	1,66	7,04E-06	2,99E-04	NM_011967	NM_011967	NM_011967	Mm.208883	26442	ENSMUST00000079684
A_51_P428364	Psma6	proteasome (prosome, macropain) subunit, alpha type 6	0,97	1,96	3,91E-06	2,11E-04	NM_011968	NM_011968	NM_011968	Mm.30210	26443	ENSMUST00000162972
A_51_P367162	Psmb1	proteasome (prosome, macropain) subunit, beta type 1	0,99	1,99	1,01E-06	1,01E-04	NM_011185	NM_011185	NM_011185	Mm.32912	19170	ENSMUST00000014913
A_51_P468329	Psmb7	proteasome (prosome, macropain) subunit, beta type 7	1,16	2,23	1,30E-06	1,13E-04	NM_011187	NM_011187	NM_011187	Mm.389251	19177	ENSMUST00000151981
A_55_P2009064	Psmc1	protease (prosome, macropain) 26S subunit, ATPase 1	0,84	1,79	1,04E-05	3,76E-04	NM_008947	NM_008947	NM_008947	Mm.157105	19179	ENSMUST00000021595
A_55_P2103422	Psmc2	proteasome (prosome, macropain) 26S subunit, ATPase 2	0,90	1,86	6,38E-06	2,83E-04	NM_011188	NM_011188	NM_011188	Mm.2462	19181	ENSMUST00000030769
A_55_P2121961	Psmc3	proteasome (prosome, macropain) 26S subunit, ATPase 3	0,69	1,61	5,99E-05	1,08E-03	NM_008948	NM_008948	NM_008948	Mm.289832	19182	ENSMUST00000067663
A_52_P10254	Psmc4	proteasome (prosome, macropain) 26S subunit, ATPase, 4	0,63	1,55	1,34E-04	1,84E-03	NM_011874	NM_011874	NM_011874	Mm.29582	23996	ENSMUST00000135482
A_55_P2061591	Psmd1	proteasome (prosome, macropain) 26S subunit, non-ATPase,	0,98	1,98	2,65E-06	1,66E-04	NM_027357	NM_027357	NM_027357	Mm.280013	70247	0

A_52_P652212	***Psmd14***	proteasome (prosome, macropain) 26S subunit, non-ATPase,	0,97	1,96	1,77E-05	5,10E-04	NM_021526	NM_021526	NM_021526	Mm.218198	59029	ENSMUST00000146051
A_55_P1976007	***Psme4***	proteasome (prosome, macropain) activator subunit 4	0,57	1,48	4,55E-05	9,09E-04	NM_134013	NM_134013	NM_134013	Mm.481953	103554	ENSMUST00000129824
A_66_P139876	***Psph***	phosphoserine phosphatase	0,66	1,58	1,22E-04	1,73E-03	NM_133900	NM_133900	NM_133900	Mm.271784	100678	ENSMUST00000031399
A_55_P2021545	***Ptcd1***	pentatricopeptide repeat domain 1	0,87	1,82	7,39E-05	1,24E-03	AK172986	0	AK172986	Mm.332840	71799	0
A_55_P2040227	***Ptchd4***	patched domain containing 4	0,79	1,73	1,22E-04	1,72E-03	NM_028474	NM_028474	NM_028474	Mm.390999	627626	ENSMUST00000048691
A_51_P442053	***Ptdss1***	phosphatidylserine synthase 1	1,30	2,47	1,15E-05	3,96E-04	NM_008959	NM_008959	NM_008959	Mm.281464	19210	ENSMUST00000021990
A_51_P185713	***Ptdss2***	phosphatidylserine synthase 2	0,69	1,61	2,50E-05	6,27E-04	NM_013782	NM_013782	NM_013782	Mm.293591	27388	ENSMUST00000026568
A_51_P349008	***Ptges2***	prostaglandin E synthase 2	0,88	1,84	4,19E-05	8,59E-04	NM_133783	NM_133783	NM_133783	Mm.28048	96979	ENSMUST00000028162
A_55_P2349265	***Ptk2***	PTK2 protein tyrosine kinase 2	0,68	1,60	1,11E-04	1,62E-03	NM_00113040	NM_00113040	NM_00113040	Mm.254494	14083	ENSMUST00000170939
A_51_P311904	***Ptk2b***	PTK2 protein tyrosine kinase 2 beta	0,87	1,82	1,02E-05	3,72E-04	NM_172498	NM_172498	NM_172498	Mm.21613	19229	ENSMUST00000136216
A_52_P585652	***Ptp4a2***	protein tyrosine phosphatase 4a2	0,69	1,61	1,96E-05	5,44E-04	NM_008974	NM_008974	NM_008974	Mm.193688	19244	ENSMUST00000030578
A_55_P2069485	***Ptpn13***	protein tyrosine phosphatase, non-receptor type 13	1,11	2,15	3,43E-06	1,94E-04	NM_011204	NM_011204	NM_011204	Mm.3414	19249	ENSMUST00000048957
A_55_P2172989	***Ptpn23***	protein tyrosine phosphatase, non-receptor type 23	1,05	2,07	1,29E-06	1,13E-04	NM_00108104	NM_00108104	NM_00108104	Mm.335477	104831	ENSMUST00000040021
A_55_P1987694	***Ptprd***	protein tyrosine phosphatase, receptor type, D	1,18	2,26	1,05E-06	1,02E-04	NM_011211	NM_011211	NM_011211	Mm.184021	19266	ENSMUST00000084467
A_55_P1992079	***Ptprf***	protein tyrosine phosphatase, receptor type, F	0,98	1,98	5,15E-06	2,47E-04	NM_011213	NM_011213	NM_011213	Mm.29855	19268	ENSMUST00000124758
A_55_P2006327	***Ptprg***	protein tyrosine phosphatase, receptor type, G	0,75	1,68	2,75E-05	6,63E-04	NM_008981	NM_008981	NM_008981	Mm.431266	19270	ENSMUST00000142917
A_52_P246165	***Ptprk***	protein tyrosine phosphatase, receptor type, K	0,74	1,67	8,77E-05	1,39E-03	NM_008983	NM_008983	NM_008983	Mm.332303	19272	ENSMUST00000166468
A_55_P2028591	***Ptprm***	protein tyrosine phosphatase, receptor type, M	1,01	2,02	7,86E-05	1,29E-03	NM_008984	NM_008984	NM_008984	Mm.311809	19274	ENSMUST00000037974
A_52_P15490	***Pvrl3***	poliovirus receptor-related 3	0,82	1,76	3,70E-05	7,91E-04	NM_021496	NM_021496	NM_021496	Mm.328072	58998	ENSMUST00000096052
A_55_P2104327	***Pxdn***	peroxidasin homolog (Drosophila)	0,72	1,65	1,18E-04	1,69E-03	NM_181395	NM_181395	NM_181395	Mm.251774	69675	ENSMUST00000122328
A_55_P2011545	***Pyroxd1***	pyridine nucleotide-disulphide oxidoreductase domain 1	1,11	2,15	8,68E-06	3,39E-04	NM_183165	NM_183165	NM_183165	Mm.490474	232491	ENSMUST00000041852
A_51_P287232	***Qars***	glutaminyl-tRNA synthetase	0,75	1,68	1,03E-04	1,54E-03	NM_133794	NM_133794	NM_133794	Mm.272427	97541	ENSMUST00000136293
A_52_P543792	***Qrich1***	glutamine-rich 1	1,02	2,03	3,12E-05	7,13E-04	NM_175143	NM_175143	NM_175143	Mm.485781	69232	ENSMUST00000006851
A_52_P138895	***Qsox2***	quiescin Q6 sulfhydryl oxidase 2	0,60	1,52	2,03E-05	5,57E-04	NM_153559	NM_153559	NM_153559	Mm.116769	227638	ENSMUST00000036187
A_52_P444162	***Rab11a***	RAB11a, member RAS oncogene family	0,68	1,61	9,18E-05	1,43E-03	NM_017382	NM_017382	NM_017382	Mm.1387	53869	ENSMUST00000172298
A_55_P2082156	***Rab11fip3***	RAB11 family interacting protein 3 (class II)	1,30	2,46	6,06E-06	2,75E-04	NM_00116286	NM_00116286	NM_00116286	Mm.41191	215445	ENSMUST00000120691
A_52_P529360	***Rab11fip5***	RAB11 family interacting protein 5 (class I)	0,96	1,94	9,74E-05	1,49E-03	NM_00100395	NM_00100395	NM_00100395	Mm.220334	52055	ENSMUST00000060837
A_51_P419117	***Rab15***	RAB15, member RAS oncogene family	0,51	1,43	9,04E-05	1,42E-03	NM_134050	NM_134050	NM_134050	Mm.172847	104886	ENSMUST00000021459
A_51_P209280	***Rab31***	RAB31, member RAS oncogene family	0,71	1,63	1,78E-05	5,13E-04	NM_133685	NM_133685	NM_133685	Mm.29274	106572	ENSMUST00000070673
A_52_P558939	***Rab3gap1***	RAB3 GTPase activating protein subunit 1	0,87	1,82	2,82E-05	6,72E-04	NM_178690	NM_178690	NM_178690	Mm.489713	226407	ENSMUST00000037649
A_51_P244558	***Rab3gap2***	RAB3 GTPase activating protein subunit 2	0,89	1,86	8,39E-06	3,32E-04	NM_00116375	NM_00116375	NM_00116375	Mm.275841	98732	ENSMUST00000069652
A_51_P117604	***Rab5a***	RAB5A, member RAS oncogene family	1,09	2,12	2,06E-06	1,44E-04	NM_025887	NM_025887	NM_025887	Mm.329123	271457	ENSMUST00000017975
A_55_P2060097	***Rabep1***	rabaptin, RAB GTPase binding effector protein 1	0,61	1,53	1,09E-04	1,60E-03	ENSMUST000	0	BC003921	Mm.7087	54189	ENSMUST00000100928
A_55_P2167818	***Rabgef1***	RAB guanine nucleotide exchange factor (GEF) 1	0,94	1,92	3,54E-06	1,99E-04	NM_019983	NM_019983	NM_019983	Mm.288639	56715	ENSMUST00000125937
A_51_P384091	***Rad21***	RAD21 homolog (S. pombe)	0,81	1,75	1,14E-05	3,93E-04	NM_009009	NM_009009	NM_009009	Mm.182628	19357	ENSMUST00000022927
A_55_P2042958	***Rad50***	RAD50 homolog (S. cerevisiae)	1,36	2,56	1,66E-07	4,37E-05	NM_009012	NM_009012	NM_009012	Mm.4888	19360	ENSMUST00000020649
A_55_P2142371	***Rai1***	retinoic acid induced 1	1,34	2,52	1,36E-07	4,05E-05	NM_009021	NM_009021	NM_009021	Mm.296366	19377	ENSMUST00000064190
A_52_P51029	***Ralgapa1***	Ral GTPase activating protein, alpha subunit 1	1,05	2,07	1,09E-04	1,60E-03	NM_019994	NM_019994	NM_019994	Mm.292180	56784	ENSMUST00000110687
A_51_P511375	***Ranbp2***	RAN binding protein 2	2,12	4,36	4,15E-08	2,20E-05	NM_011240	NM_011240	NM_011240	Mm.431695	19386	ENSMUST00000003310
A_52_P450123	***Ranbp6***	RAN binding protein 6	1,27	2,41	1,90E-06	1,38E-04	NM_177721	NM_177721	NM_177721	Mm.125503	240614	ENSMUST00000099525
A_55_P2064004	***Rapgef2***	Rap guanine nucleotide exchange factor (GEF) 2	0,89	1,85	1,10E-05	3,86E-04	NM_00109962	NM_00109962	NM_00109962	Mm.31220	76089	ENSMUST00000118340
A_51_P285916	***Rapgef4***	Rap guanine nucleotide exchange factor (GEF) 4	0,95	1,93	3,37E-05	7,47E-04	NM_019688	NM_019688	NM_019688	Mm.196153	56508	ENSMUST00000102698
A_55_P2113683	***Raph1***	Ras association (RalGDS/AF-6) and pleckstrin homology dom	0,97	1,96	1,10E-04	1,57E-03	NM_00104551	NM_00104551	NM_00104551	Mm.440325	77300	0
A_52_P288614	***Rars***	arginyl-tRNA synthetase	1,02	2,03	2,35E-05	6,03E-04	NM_025936	NM_025936	NM_025936	Mm.284906	104458	ENSMUST00000165817
A_55_P1968789	***Rasa3***	RAS p21 protein activator 3	0,66	1,59	6,90E-05	1,18E-03	NM_009025	NM_009025	NM_009025	Mm.18517	19414	ENSMUST00000117551
A_52_P16873	***Rasal3***	RAS protein activator like 3	0,96	1,95	2,88E-05	6,80E-04	NM_178785	NM_178785	NM_178785	Mm.122284	320484	ENSMUST00000137458
A_52_P671676	***Rasgrf1***	RAS protein-specific guanine nucleotide-releasing factor 1	1,04	2,06	2,25E-06	1,51E-04	NM_011245	NM_011245	NM_011245	Mm.44561	19417	ENSMUST00000034912
A_55_P2096768	***Rasgrf2***	RAS protein-specific guanine nucleotide-releasing factor 2	1,19	2,28	6,09E-06	2,76E-04	NM_009027	NM_009027	NM_009027	Mm.248630	19418	ENSMUST00000099326
A_55_P1969650	***Rasgrp1***	RAS guanyl releasing protein 1	0,63	1,55	1,01E-04	1,52E-03	NM_011246	NM_011246	NM_011246	Mm.42150	19419	ENSMUST00000178884
A_55_P2143233	***Rasgrp2***	RAS, guanyl releasing protein 2	1,03	2,04	5,43E-05	1,02E-03	NM_011242	NM_011242	NM_011242	Mm.77017	19395	ENSMUST00000113468
A_55_P2119030	***Rasl2-9***	RAS-like, family 2, locus 9	0,63	1,55	7,46E-05	1,24E-03	NM_009028	NM_009028	NM_009028	Mm.322186	19428	ENSMUST00000147835
A_52_P542172	***Rb1cc1***	RB1-inducible coiled-coil 1	0,90	1,86	6,92E-05	1,18E-03	NM_009826	NM_009826	NM_009826	Mm.293811	12421	ENSMUST00000159656
A_51_P516850	***Rbbp5***	retinoblastoma binding protein 5	0,66	1,59	1,36E-04	1,85E-03	NM_172517	NM_172517	NM_172517	Mm.132868	213464	ENSMUST00000027700
A_55_P2080542	***Rbbp7***	retinoblastoma binding protein 7	1,05	2,07	2,31E-06	1,54E-04	NM_009031	NM_009031	NM_009031	Mm.270186	245688	ENSMUST00000033720
A_55_P2016739	***Rbm12b2***	RNA binding motif protein 12 B2	0,74	1,67	6,00E-05	1,08E-03	NM_198957	NM_198957	NM_198957	Mm.474129	77604	ENSMUST00000063839
A_55_P1996742	***Rbm14***	RNA binding motif protein 14	0,95	1,93	3,45E-05	7,58E-04	NM_019869	NM_019869	NM_019869	Mm.276338	56275	ENSMUST00000006625
A_55_P1968143	***Rbm25***	RNA binding motif protein 25	0,73	1,65	1,90E-05	5,34E-04	NM_027349	NM_027349	NM_027349	Mm.46005	67039	ENSMUST00000048155
A_52_P574993	***Rbpjl***	recombination signal binding protein for immunoglobulin kapp	0,61	1,53	1,03E-04	1,61E-03	NM_009036	NM_009036	NM_009036	Mm.473794	19668	ENSMUST00000137427
A_66_P117389	***Rcn2***	reticulocalbin 2	1,49	2,81	5,06E-05	9,72E-04	NM_011992	NM_011992	NM_011992	Mm.1782	26611	ENSMUST00000114276
A_55_P2183172	***Rdx***	radixin	0,83	1,77	2,65E-06	1,66E-04	NM_00110461	NM_00110461	NM_00110461	Mm.245746	19684	ENSMUST00000061352
A_51_P297963	***Recql***	RecQ protein-like	0,75	1,68	1,38E-05	4,42E-04	NM_023042	NM_023042	NM_023042	Mm.27407	19691	ENSMUST00000111803
A_55_P2035742	***Reg3d***	regenerating islet-derived 3 delta	1,18	2,27	3,05E-05	7,07E-04	NM_013893	NM_013893	NM_013893	Mm.33691	30053	ENSMUST00000089667
A_66_P108810	***Reln***	reelin	1,36	2,56	9,38E-07	9,77E-05	NM_011261	NM_011261	NM_011261	Mm.425236	19699	ENSMUST00000159768
A_55_P2013357	***Renbp***	renin binding protein	0,62	1,54	1,19E-04	1,70E-03	NM_023132	NM_023132	NM_023132	Mm.236969	19703	ENSMUST00000135711
A_55_P1968698	***Rere***	arginine glutamic acid dipeptide (RE) repeats	0,86	1,81	5,33E-05	1,01E-03	NM_00108549	NM_00108549	NM_00108549	Mm.291274	68703	ENSMUST00000105682
A_51_P271665	***Rev3l***	REV3-like, catalytic subunit of DNA polymerase zeta RAD54 l	0,96	1,95	5,11E-06	2,46E-04	NM_011264	NM_011264	NM_011264	Mm.439723	19714	ENSMUST00000131186
A_55_P2122444	***Rgag4***	retrotransposon gag domain containing 4	1,24	2,36	1,20E-05	4,03E-04	NM_183318	NM_183318	NM_183318	Mm.23297	331474	ENSMUST00000119076
A_55_P1982563	***Rhbdf1***	rhomboid family 1 (Drosophila)	0,60	1,52	8,46E-05	1,35E-03	NM_010117	NM_010117	NM_010117	Mm.11545	13650	ENSMUST00000146179
A_52_P665295	***Ric3***	resistance to inhibitors of cholinesterase 3 homolog (C. elegai	0,82	1,76	6,57E-05	1,15E-03	NM_00103862	NM_00103862	NM_00103862	Mm.71007	320360	ENSMUST00000055993
A_52_P145412	***Rictor***	RPTOR independent companion of MTOR, complex 2	0,88	1,85	9,25E-05	1,44E-03	NM_030168	NM_030168	NM_030168	Mm.275811	78757	ENSMUST00000061656
A_55_P2015964	***Rif1***	Rap1 interacting factor 1 homolog (yeast)	1,21	2,31	1,35E-05	7,60E-04	NM_175238	NM_175238	NM_175238	Mm.254530	51869	ENSMUST00000125322
A_66_P125741	***Ripply2***	ripply2 homolog (zebrafish)	1,03	2,05	1,13E-04	1,64E-03	NM_00103790	NM_00103790	NM_00103790	Mm.92244	382089	ENSMUST00000058846
A_55_P2054897	***Rnd1***	Rho family GTPase 1	0,71	1,64	1,41E-04	1,90E-03	NM_172612	NM_172612	NM_172612	Mm.274010	223881	ENSMUST00000120997
A_55_P2184009	***Rnd2***	Rho family GTPase 2	0,64	1,56	2,53E-05	6,32E-04	NM_009708	NM_009708	NM_009708	Mm.378883	11858	ENSMUST00000153185
A_52_P255250	***Rnf103***	ring finger protein 103	0,87	1,83	2,35E-05	6,03E-04	NM_009543	NM_009543	NM_009543	Mm.422969	22644	ENSMUST00000150069
A_65_P02958	***Rnf13***	ring finger protein 13	0,85	1,81	9,75E-05	1,49E-03	NM_00111341	NM_00111341	NM_00111341	Mm.274360	24017	ENSMUST00000041826
A_52_P228964	***Rnf150***	ring finger protein 150	0,74	1,67	6,06E-05	1,09E-03	NM_177378	NM_177378	NM_177378	Mm.66737	330812	ENSMUST00000078525
A_66_P124724	***Rnf19b***	ring finger protein 19B	0,77	1,71	2,73E-05	6,61E-04	ENSMUST000	0	BC117807	Mm.259672	75234	ENSMUST00000030584
A_51_P159503	***Rnf213***	ring finger protein 213	1,82	3,52	9,12E-07	9,69E-05	ENSMUST000	XM_00147665	XM_00147665	Mm.133342	672511	ENSMUST00000093902
A_51_P135939	***Rnf219***	ring finger protein 219	1,46	2,74	2,30E-05	5,96E-04	NM_026047	NM_026047	NM_026047	Mm.291811	72486	ENSMUST00000022716
A_55_P2043330	***Rnf26***	ring finger protein 26	1,60	3,03	1,66E-08	1,39E-05	NM_153762	NM_153762	NM_153762	Mm.392032	213211	ENSMUST00000097218
A_51_P236013	***Rnf40***	ring finger protein 40	0,81	1,75	6,13E-05	1,10E-03	NM_172281	NM_172281	NM_172281	Mm.41122	233900	ENSMUST00000033088
A_52_P466799	***Rngtt***	RNA guanylyltransferase and 5'-phosphatase	1,13	2,18	1,61E-05	4,83E-04	NM_011884	NM_011884	NM_011884	Mm.240024	24018	ENSMUST00000029942
A_55_P2118734	***Rnmt***	RNA (guanine-7-) methyltransferase	0,86	1,81	2,83E-06	1,74E-04	NM_026440	NM_026440	NM_026440	Mm.27544	67897	ENSMUST00000009679
A_55_P1985070	***Robo1***	roundabout homolog 1 (Drosophila)	1,23	2,34	3,88E-05	8,15E-04	NM_019413	NM_019413	NM_019413	Mm.310772	19876	ENSMUST00000023600
A_55_P2083929	***Robo2***	roundabout homolog 2 (Drosophila)	1,02	2,03	5,54E-05	1,03E-03	NM_175549	NM_175549	NM_175549	Mm.171736	268902	ENSMUST00000149114
A_55_P2036026	***Robo4***	roundabout homolog 4 (Drosophila)	0,53	1,45	1,17E-04	1,68E-03	NM_028783	NM_028783	NM_028783	Mm.485070	74144	ENSMUST00000102895
A_52_P119672	***Rock1***	Rho-associated coiled-coil containing protein kinase 1	0,89	1,85	8,96E-05	1,41E-03	NM_009071	NM_009071	NM_009071	Mm.6710	19877	ENSMUST00000067947
A_51_P320444	***Rock2***	Rho-associated coiled-coil containing protein kinase 2	0,90	1,87	2,43E-05	6,16E-04	NM_009072	NM_009072	NM_009072	Mm.276024	19878	ENSMUST00000020904
A_55_P2078123	***Rora***	RAR-related orphan receptor alpha	0,62	1,54	2,18E-05	5,82E-04	NM_013646	NM_013646	NM_013646	Mm.427266	19883	ENSMUST00000113624
A_55_P2135556	***Rpgrip1***	retinitis pigmentosa GTPase regulator interacting protein 1	0,61	1,53	1,22E-04	1,73E-03	NM_023879	NM_023879	NM_023879	Mm.21662	77945	ENSMUST00000111603
A_52_P152219	***Rpgrip1l***	Rpgrip1-like	1,28	2,43	1,54E-06	1,23E-04	NM_173431	NM_173431	NM_173431	Mm.324592	244585	ENSMUST00000047483
A_55_P2039205	***Rprd2***	regulation of nuclear pre-mRNA domain containing 2	1,12	2,18	9,24E-06	3,52E-04	NM_00108129	NM_00108129	NM_00108129	Mm.196275	75137	ENSMUST00000090791
A_55_P2109222	***Rps3***	ribosomal protein S3	0,50	1,42	8,39E-05	1,34E-03	AK146165	0	AK146165	Mm.236868	27050	0
A_51_P292357	***Rps3a***	ribosomal protein S3A	0,63	1,54	1,38E-04	1,88E-03	NM_016959	NM_016959	NM_016959	Mm.399829	20091	ENSMUST00000074680
A_55_P2078115	***Rps6kc1***	ribosomal protein S6 kinase polypeptide 1	0,78	1,72	2,08E-05	5,64E-04	NM_178775	NM_178775	NM_178775	Mm.220912	320119	ENSMUST00000162692
A_51_P502082	***Rrm1***	ribonucleotide reductase M1	1,12	2,17	4,24E-06	2,20E-04	NM_009103	NM_009103	NM_009103	Mm.197486	20133	ENSMUST00000033283
A_55_P2132805	***Rsf1***	remodeling and spacing factor 1	1,31	2,47	1,20E-05	4,04E-04	NM_00108126	NM_00108126	NM_00108126	Mm.43977	233532	ENSMUST00000042399
A_52_P191975	***Rtn3***	reticulon 3	1,59	3,01	7,47E-08	2,84E-05	NM_00100393	NM_00100393	NM_00100393	Mm.246990	20168	ENSMUST00000088169
A_52_P379337	***Rtn4***	reticulon 4	0,84	1,79	4,62E-05	9,17E-04	NM_194054	NM_194054	NM_194054	Mm.192580	68585	ENSMUST00000170731
A_55_P2267736	***Rufy2***	RUN and FYVE domain-containing 2	1,41	2,65	2,25E-05	5,90E-04	NM_027425	NM_027425	NM_027425	Mm.234587	70432	0
A_51_P519756	***Rusc1***	RUN and SH3 domain containing 1	0,72	1,65	1,93E-05	5,39E-04	NM_028188	NM_028188	NM_028188	Mm.27687	72296	ENSMUST00000090929
A_55_P2124153	***Rusc2***	RUN and SH3 domain containing 2	0,81	1,75	7,79E-06	3,17E-04	NM_00103770	NM_00103770	NM_00103770	Mm.235730	100213	ENSMUST00000098106
A_52_P440709	***Ryr2***	ryanodine receptor 2, cardiac	1,72	3,29	4,63E-08	2,24E-05	NM_023868	NM_023868	NM_023868	Mm.239871	20191	ENSMUST00000021750
A_55_P2009622	***S100pbp***	S100P binding protein	1,14	2,20	6,49E-07	8,29E-05	NM_029036	NM_029036	NM_029036	Mm.172064	74648	ENSMUST00000033283
A_52_P578043	***Sacs***	sacsin	2,33	5,04	2,09E-09	5,69E-06	ENSMUST000	0	AK173011	Mm.440703	50720	ENSMUST00000119943
A_51_P137094	***Sall2***	sal-like 2 (Drosophila)	1,03	2,04	4,52E-06	2,28E-04	NM_015772	NM_015772	NM_015772	Mm.39487	50524	ENSMUST00000058326
A_52_P478256	***Samm50***	sorting and assembly machinery component 50 homolog (S. c	0,63	1,55	5,15E-05	9,82E-04	NM_178614	NM_178614	NM_178614	Mm.290725	68653	ENSMUST00000023071
A_52_P649224	***Sash1***	SAM and SH3 domain containing 1	1,65	3,15	3,12E-06	1,84E-04	NM_175155	NM_175155	NM_175155	Mm.338275	70097	ENSMUST00000015449
A_51_P324228	***Satb1***	special AT-rich sequence binding protein 1	0,83	1,78	8,55E-05	1,36E-03	NM_009122	NM_009122	NM_009122	Mm.311655	20230	ENSMUST00000152830

A_55_P1960626	***Satb2***	special AT-rich sequence binding protein 2	1,35	2,55	1,58E-06	1,25E-04	NM_139146	NM_139146	NM_139146	Mm.145599	212712	ENSMUST00000042857
A_51_P123805	***Sbno1***	sno, strawberry notch homolog 1 (Drosophila)	0,91	1,88	3,13E-05	7,15E-04	NM_0010812C	NM_0010812C	NM_0010812C	Mm.23879	243272	ENSMUST00000065263
A_55_P2055477	***Scaf11***	SR-related CTD-associated factor 11	1,57	2,98	2,44E-06	1,58E-04	NM_028148	NM_028148	NM_028148	Mm.324474	72193	ENSMUST00000047835
A_51_P298666	***Scaf4***	SR-related CTD-associated factor 4	0,72	1,65	1,21E-04	1,72E-03	NM_178923	NM_178923	NM_178923	Mm.439811	224432	ENSMUST00000039280
A_51_P494006	***Scaf8***	SR-related CTD-associated factor 8	0,51	1,43	1,18E-04	1,68E-03	NM_134123	NM_134123	NM_134123	Mm.124373	106583	ENSMUST00000076734
A_55_P2426272	***Scaper***	S phase cyclin A-associated protein in the ER	1,73	3,31	5,10E-07	7,35E-05	NM_00108134	NM_00108134	NM_00108134	Mm.86588	244891	ENSMUST00000037408
A_52_P682382	***Scd1***	stearoyl-Coenzyme A desaturase 1	1,15	2,22	3,33E-05	7,41E-04	NM_009127	NM_009127	NM_009127	Mm.267377	20249	ENSMUST00000041331
A_51_P129464	***Scd2***	stearoyl-Coenzyme A desaturase 2	1,26	2,39	5,92E-06	2,71E-04	NM_009128	NM_009128	NM_009128	Mm.487021	20250	ENSMUST00000026221
A_55_P2083949	***Scfd1***	Sec1 family domain containing 1	1,55	2,93	3,52E-07	6,19E-05	AK129238	0	AK129238	Mm.216511	76983	0
A_55_P2067905	***Scfd2***	Sec1 family domain containing 2	0,65	1,57	3,64E-05	7,83E-04	NM_00111466	NM_00111466	NM_00111466	Mm.134147	212986	ENSMUST00000113542
A_55_P2106514	***Scn2a1***	sodium channel, voltage-gated, type II, alpha 1	1,05	2,07	7,67E-06	3,14E-04	NM_00109929	NM_00109929	NM_00109929	Mm.220329	110876	ENSMUST00000144254
A_66_P106808	***Scn3a***	sodium channel, voltage-gated, type III, alpha	0,96	1,95	2,69E-05	6,55E-04	NM_018732	NM_018732	NM_018732	Mm.330256	20269	ENSMUST00000100069
A_55_P2072333	***Scn8a***	sodium channel, voltage-gated, type VIII, alpha	1,27	2,41	5,15E-07	7,41E-05	NM_00107749	NM_00107749	NM_00107749	Mm.385012	20273	ENSMUST00000108909
A_55_P2040748	***Scrib***	scribbled homolog (Drosophila)	0,78	1,72	2,82E-05	6,72E-04	NM_134089	NM_134089	NM_134089	Mm.25568	105782	ENSMUST00000109946
A_55_P2002562	***Sec11a***	SEC11 homolog A (S. cerevisiae)	1,04	2,05	2,22E-06	1,50E-04	NM_019951	NM_019951	NM_019951	Mm.43760	56529	ENSMUST00000147813
A_55_P1962736	***Sec23b***	SEC23B (S. cerevisiae)	0,81	1,76	2,25E-05	5,90E-04	NM_019787	NM_019787	NM_019787	Mm.248492	27054	ENSMUST00000028916
A_51_P477440	***Sec23ip***	Sec23 interacting protein	0,77	1,71	4,06E-05	8,41E-04	NM_00102998	NM_00102998	NM_00102998	Mm.297105	207352	ENSMUST00000042942
A_55_P2024575	***Sec24c***	Sec24 related gene family, member C (S. cerevisiae)	0,48	1,39	9,18E-05	1,43E-03	NM_172596	NM_172596	NM_172596	Mm.173119	218811	ENSMUST00000048657
A_51_P129100	***Sec63***	SEC63-like (S. cerevisiae)	0,79	1,73	9,03E-06	3,47E-04	NM_153055	NM_153055	NM_153055	Mm.214344	140740	ENSMUST00000124613
A_55_P2054013	***Sema3a***	sema domain, immunoglobulin domain (Ig), short basic domai	1,67	3,19	1,43E-07	4,10E-05	NM_009152	NM_009152	NM_009152	Mm.372039	20346	ENSMUST00000095012
A_55_P2103026	***Sema3d***	sema domain, immunoglobulin domain (Ig), short basic domai	0,98	1,97	2,64E-06	1,65E-04	NM_028882	NM_028882	NM_028882	Mm.89313	108151	ENSMUST00000030868
A_51_P462516	***Senp7***	SUMO1/sentrin specific peptidase 7	0,92	1,89	9,31E-06	3,54E-04	NM_00100397	NM_00100397	NM_00100397	Mm.255784	66315	ENSMUST00000049128
A_51_P214343	***Sephs1***	selenophosphate synthetase 1	0,57	1,49	4,89E-05	9,53E-04	NM_175400	NM_175400	NM_175400	Mm.34329	109079	ENSMUST00000115019
A_52_P153327	***Sept2***	septin 2	0,97	1,96	1,03E-06	1,02E-04	NM_010891	NM_010891	NM_010891	Mm.428652	18000	ENSMUST00000131175
A_55_P2109554	***Serinc3***	serine incorporator 3	0,83	1,78	3,86E-05	8,13E-04	NM_012032	NM_012032	NM_012032	Mm.218473	26943	ENSMUST00000017851
A_51_P128336	***Serinc5***	serine incorporator 5	0,86	1,81	1,99E-05	5,48E-04	NM_172588	NM_172588	NM_172588	Mm.297970	218442	ENSMUST00000049488
A_51_P268094	***Serpine2***	serine (or cysteine) peptidase inhibitor, clade E, member 2	0,86	1,81	8,86E-05	1,40E-03	NM_009255	NM_009255	NM_009255	Mm.3093	20720	ENSMUST00000027467
A_66_P100165	***Serpini1***	serine (or cysteine) peptidase inhibitor, clade I, member 1	1,23	2,34	1,47E-05	4,60E-04	NM_009250	NM_009250	NM_009250	Mm.41560	20713	ENSMUST00000161776
A_52_P230167	***Setbp1***	SET binding protein 1	0,91	1,88	3,06E-05	7,08E-04	NM_053099	NM_053099	NM_053099	Mm.312871	240427	ENSMUST00000025430
A_52_P468033	***Setd1b***	SET domain containing 1B	0,87	1,83	1,70E-05	4,98E-04	NM_00104039	NM_00104039	NM_00104039	Mm.250391	208043	ENSMUST00000174836
A_51_P110395	***Setd2***	SET domain containing 2	1,23	2,35	1,64E-06	1,26E-04	NM_00108134	NM_00108134	NM_00108134	Mm.288949	235626	ENSMUST00000173373
A_55_P1965635	***Setdb1***	SET domain, bifurcated 1	2,10	4,28	1,23E-06	1,10E-04	NM_00116364	NM_00116364	NM_00116364	Mm.490259	84505	ENSMUST00000124638
A_51_P470448	***Setx***	senataxin	1,11	2,16	9,27E-06	3,53E-04	NM_198033	NM_198033	NM_198033	Mm.41867	269254	ENSMUST00000061578
A_55_P2118744	***Sez6l***	seizure related 6 homolog like	1,24	2,36	2,10E-07	4,78E-05	NM_00125391	NM_00125391	NM_00125391	Mm.143742	56747	ENSMUST00000079491
A_51_P504442	***Sf3a1***	splicing factor 3a, subunit 1	0,67	1,59	6,44E-05	1,13E-03	NM_026175	NM_026175	NM_026175	Mm.156914	67465	ENSMUST00000002198
A_66_P108345	***Sgcb***	sarcoglycan, beta (dystrophin-associated glycoprotein)	0,91	1,88	1,53E-05	4,70E-04	NM_011890	NM_011890	NM_011890	Mm.89310	24051	ENSMUST00000081170
A_55_P1984730	***Sgpl1***	sphingosine phosphate lyase 1	0,76	1,69	7,86E-05	1,29E-03	NM_009163	NM_009163	NM_009163	Mm.412319	20397	ENSMUST00000122259
A_55_P2088705	***Sgsm1***	small G protein signaling modulator 1	0,85	1,80	1,90E-05	5,35E-04	NM_00125473	NM_00125473	NM_00125473	Mm.200203	52850	ENSMUST00000145708
A_55_P2130249	***Sh3gl2***	SH3-domain GRB2-like 2	0,79	1,73	9,48E-05	1,46E-03	NM_019535	NM_019535	NM_019535	Mm.143603	20404	ENSMUST00000107188
A_55_P2062181	***Shank1***	SH3/ankyrin domain gene 1	1,05	2,07	4,86E-05	9,48E-04	ENSMUST000C	0	AK078290	Mm.360368	243961	ENSMUST00000154776
A_55_P2051656	***Shank2***	SH3/ankyrin domain gene 2	0,88	1,85	1,34E-05	4,33E-04	NM_00111337	NM_00111337	NM_00111337	Mm.483624	210274	ENSMUST00000097929
A_55_P2154749	***Shisa6***	shisa homolog 6 (Xenopus laevis)	0,72	1,65	2,78E-05	6,68E-04	NM_00103487	NM_00103487	NM_00103487	Mm.351764	380702	ENSMUST00000123454
A_55_P2153541	***Sik3***	SIK family kinase 3	0,74	1,68	3,52E-05	7,68E-04	NM_027498	NM_027498	NM_027498	Mm.219459	70661	ENSMUST00000126865
A_55_P2112693	***Sipa1l1***	signal-induced proliferation-associated 1 like 1	2,33	5,02	7,71E-08	2,87E-05	NM_00116798	NM_00116798	NM_00116798	Mm.261333	217692	ENSMUST00000166429
A_55_P2098782	***Sipa1l2***	signal-induced proliferation-associated 1 like 2	1,05	2,08	1,95E-05	5,42E-04	AK122501	0	AK122501	Mm.271668	244668	0
A_55_P1975732	***Sipa1l3***	signal-induced proliferation-associated 1 like 3	0,62	1,54	1,28E-04	1,78E-03	NM_00108102	NM_00108102	NM_00108102	Mm.375176	74206	ENSMUST00000085809
A_51_P243914	***Skap2***	src family associated phosphoprotein 2	0,85	1,80	1,61E-05	4,83E-04	NM_018773	NM_018773	NM_018773	Mm.221479	54353	ENSMUST00000078214
A_51_P118132	***Skil***	SKI-like	0,75	1,68	8,13E-06	3,27E-04	NM_011386	NM_011386	NM_011386	Mm.15406	20482	ENSMUST00000029194
A_52_P614960	***Skiv2l***	superkiller viralicidic activity 2-like (S. cerevisiae)	1,26	2,39	2,07E-06	1,44E-04	NM_021337	NM_021337	NM_021337	Mm.18845	108077	ENSMUST00000174363
A_51_P476711	***Skiv2l2***	superkiller viralicidic activity 2-like 2 (S. cerevisiae)	1,22	2,32	7,32E-06	3,06E-04	NM_028151	NM_028151	NM_028151	Mm.291029	72198	ENSMUST00000022281
A_55_P1953874	***Skor2***	SKI family transcriptional corepressor 2	0,99	1,99	1,18E-05	4,01E-04	NM_00110974	NM_00110974	NM_00110974	Mm.329543	664805	ENSMUST00000166956
A_55_P2087544	***Slc12a5***	solute carrier family 12, member 5	1,55	2,92	9,28E-06	3,53E-04	NM_020333	NM_020333	NM_020333	Mm.252987	57138	ENSMUST00000099092
A_55_P1972252	***Slc12a6***	solute carrier family 12, member 6	1,15	2,21	1,58E-05	4,79E-04	NM_133649	NM_133649	NM_133649	Mm.46449	107723	ENSMUST00000110991
A_52_P307752	***Slc12a9***	solute carrier family 12 (potassium/chloride transporters), mer	1,05	2,06	8,18E-07	9,27E-05	NM_031406	NM_031406	NM_031406	Mm.235625	83704	ENSMUST00000039991
A_55_P2044242	***Slc13a5***	solute carrier family 13 (sodium-dependent citrate transporter)	0,56	1,48	7,42E-05	1,24E-03	NM_00100414	NM_00100414	NM_00100414	Mm.340778	237831	ENSMUST00000021161
A_51_P312336	***Slc14a1***	solute carrier family 14 (urea transporter), member 1	0,85	1,80	7,43E-05	1,24E-03	NM_028122	NM_028122	NM_028122	Mm.33832	108052	ENSMUST00000160639
A_51_P267278	***Slc15a2***	solute carrier family 15 (H+/peptide transporter), member 2	0,96	1,95	1,55E-05	4,74E-04	NM_021301	NM_021301	NM_021301	Mm.281804	57738	ENSMUST00000023616
A_52_P566129	***Slc16a14***	solute carrier family 16 (monocarboxylic acid transporters), me	0,96	1,95	9,67E-06	3,61E-04	NM_027921	NM_027921	NM_027921	Mm.158754	71781	ENSMUST00000027422
A_52_P408736	***Slc16a7***	solute carrier family 16 (monocarboxylic acid transporters), me	0,92	1,89	1,02E-05	3,72E-04	ENSMUST000C	0	AK085398	Mm.29161	20503	ENSMUST00000063318
A_51_P493117	***Slc16a9***	solute carrier family 16 (monocarboxylic acid transporters), me	1,22	2,33	6,90E-07	8,59E-05	NM_025807	NM_025807	NM_025807	Mm.19325	66859	ENSMUST00000046807
A_52_P8197	***Slc17a6***	solute carrier family 17 (sodium-dependent inorganic phospha	1,07	2,11	3,70E-06	2,04E-04	NM_080853	NM_080853	NM_080853	Mm.256618	140919	ENSMUST00000032710
A_55_P2000489	***Slc18a2***	solute carrier family 18 (vesicular monoamine), member 2	0,99	1,98	9,42E-05	1,45E-03	NM_172523	NM_172523	NM_172523	Mm.361919	214084	ENSMUST00000026084
A_51_P456826	***Slc19a3***	solute carrier family 19, member 3	1,10	2,15	4,67E-06	2,33E-04	NM_030556	NM_030556	NM_030556	Mm.261542	80721	ENSMUST00000164473
A_55_P1955412	***Slc1a2***	solute carrier family 1 (glial high affinity glutamate transporter)	1,04	2,05	8,40E-05	1,34E-03	NM_011393	NM_011393	NM_011393	Mm.267547	20511	ENSMUST00000154446
A_51_P268697	***Slc1a3***	solute carrier family 1 (glial high affinity glutamate transporter)	1,12	2,17	3,35E-06	1,92E-04	NM_148938	NM_148938	NM_148938	Mm.204834	20512	ENSMUST00000005493
A_51_P453475	***Slc1a5***	solute carrier family 1 (neutral amino acid transporter), membe	1,00	2,00	1,67E-05	4,93E-04	NM_009201	NM_009201	NM_009201	Mm.1056	20514	ENSMUST00000108496
A_55_P1977245	***Slc22a13***	solute carrier family 22 (organic cation transporter), member 1	1,24	2,35	1,39E-06	1,17E-04	NM_133980	NM_133980	NM_133980	Mm.38775	102570	ENSMUST00000084797
A_52_P286520	***Slc23a2***	solute carrier family 23 (nucleobase transporters), member 2	1,01	2,01	1,55E-05	4,73E-04	NM_018824	NM_018824	NM_018824	Mm.103581	54338	ENSMUST00000028815
A_55_P2161026	***Slc24a2***	solute carrier family 24 (sodium/potassium/calcium exchanger	1,04	2,06	2,21E-06	1,50E-04	NM_00111024	NM_00111024	NM_00111024	Mm.490487	76376	ENSMUST00000155361
A_52_P169901	***Slc25a51***	solute carrier family 25, member 51	0,62	1,54	6,88E-05	1,18E-03	NM_00100994	NM_00100994	NM_00100994	Mm.260210	230125	ENSMUST00000116341
A_51_P113403	***Slc26a11***	solute carrier family 26, member 11	1,05	2,07	7,47E-06	3,10E-04	NM_178743	NM_178743	NM_178743	Mm.31869	268512	ENSMUST00000151612
A_52_P354744	***Slc2a3***	solute carrier family 2 (facilitated glucose transporter), membe	1,24	2,36	1,20E-06	1,09E-04	NM_011401	NM_011401	NM_011401	Mm.395108	20527	ENSMUST00000168704
A_51_P300572	***Slc30a1***	solute carrier family 30 (zinc transporter), member 1	0,81	1,75	5,05E-05	9,71E-04	NM_009579	NM_009579	NM_009579	Mm.9024	22782	ENSMUST00000044954
A_51_P420859	***Slc30a3***	solute carrier family 30 (zinc transporter), member 3	0,65	1,57	7,41E-05	1,24E-03	NM_011773	NM_011773	NM_011773	Mm.1396	22784	ENSMUST00000031037
A_55_P2054491	***Slc31a2***	solute carrier family 31, member 2	0,60	1,51	5,97E-05	1,08E-03	NM_025286	NM_025286	NM_025286	Mm.292539	20530	ENSMUST00000107467
A_55_P1985286	***Slc32a1***	solute carrier family 32 (GABA vesicular transporter), member	1,58	2,98	2,86E-06	1,75E-04	NM_009508	NM_009508	NM_009508	Mm.413854	22348	ENSMUST00000045738
A_55_P2176300	***Slc33a1***	solute carrier family 33 (acetyl-CoA transporter), member 1	1,44	2,71	7,55E-06	3,11E-04	NM_00127203	NM_00127203	NM_00127203	Mm.135619	11416	ENSMUST00000160883
A_55_P2110595	***Slc35a2***	solute carrier family 35 (UDP-galactose transporter), member	1,10	2,15	1,41E-05	4,47E-04	NM_078484	NM_078484	NM_078484	Mm.214976	22232	ENSMUST00000115660
A_52_P618379	***Slc35a5***	solute carrier family 35, member A5	1,20	2,29	1,65E-05	4,91E-04	NM_028756	NM_028756	NM_028756	Mm.264984	74102	ENSMUST00000180636
A_51_P209319	***Slc35b1***	solute carrier family 35, member B1	0,89	1,86	4,80E-06	2,36E-04	NM_016752	NM_016752	NM_016752	Mm.4593	110172	ENSMUST00000021243
A_51_P501773	***Slc35f3***	solute carrier family 35, member F3	0,52	1,44	1,52E-04	1,99E-03	NM_175434	NM_175434	NM_175434	Mm.459322	210027	ENSMUST00000108759
A_55_P2089020	***Slc35g2***	solute carrier family 35, member G2	1,27	2,41	7,47E-06	3,10E-04	NM_00110148	NM_00110148	NM_00110148	Mm.236289	245020	ENSMUST00000093792
A_55_P2136743	***Slc36a4***	solute carrier family 36 (proton/amino acid symporter), membe	0,79	1,73	1,37E-04	1,87E-03	NM_172289	NM_172289	NM_172289	Mm.138815	234967	ENSMUST00000061568
A_51_P122740	***Slc37a3***	solute carrier family 37 (glycerol-3-phosphate transporter), me	0,75	1,68	7,96E-05	1,29E-03	NM_028123	NM_028123	NM_028123	Mm.482167	72144	ENSMUST00000090243
A_51_P385598	***Slc37a4***	solute carrier family 37 (glucose-6-phosphate transporter), me	0,57	1,49	9,41E-05	1,45E-03	NM_008063	NM_008063	NM_008063	Mm.30087	14385	ENSMUST00000165839
A_55_P2161640	***Slc38a1***	solute carrier family 38, member 1	1,39	2,63	5,07E-07	7,34E-05	NM_134086	NM_134086	NM_134086	Mm.103568	105727	ENSMUST00000100262
A_51_P458384	***Slc38a2***	solute carrier family 38, member 2	1,15	2,21	3,86E-05	8,13E-04	NM_175121	NM_175121	NM_175121	Mm.46754	67760	ENSMUST00000023099
A_55_P2153797	***Slc38a6***	solute carrier family 38, member 6	1,39	2,62	2,57E-07	5,41E-05	ENSMUST000C	0	BC048389	Mm.37829	625098	ENSMUST00000058139
A_51_P223498	***Slc39a10***	solute carrier family 39 (zinc transporter), member 10	0,77	1,71	2,85E-05	6,76E-04	NM_172653	NM_172653	NM_172653	Mm.233889	227059	ENSMUST00000027131
A_55_P2036394	***Slc39a12***	solute carrier family 39 (zinc transporter), member 12	1,14	2,20	6,82E-06	2,94E-04	NM_0010123C	NM_0010123C	NM_0010123C	Mm.44662	277468	ENSMUST00000082290
A_51_P259214	***Slc39a6***	solute carrier family 39 (metal ion transporter), member 6	1,26	2,39	4,04E-06	2,14E-04	NM_139143	NM_139143	NM_139143	Mm.21688	106957	ENSMUST00000070726
A_55_P1976102	***Slc39a9***	solute carrier family 39 (zinc transporter), member 9	0,99	1,99	3,63E-05	7,82E-04	NM_026244	NM_026244	NM_026244	Mm.238279	328133	ENSMUST00000085245
A_52_P578562	***Slc41a1***	solute carrier family 41, member 1	0,82	1,77	1,58E-05	4,79E-04	NM_173865	NM_173865	NM_173865	Mm.389181	98396	ENSMUST00000086559
A_51_P219505	***Slc41a2***	solute carrier family 41, member 2	0,78	1,72	5,46E-05	1,02E-03	NM_177388	NM_177388	NM_177388	Mm.67073	338365	ENSMUST00000039956
A_55_P1967776	***Slc4a4***	solute carrier family 4 (anion exchanger), member 4	0,84	1,79	9,93E-06	3,67E-04	NM_018760	NM_018760	NM_018760	Mm.41044	54403	ENSMUST00000068864
A_55_P2026818	***Slc4a7***	solute carrier family 4, sodium bicarbonate cotransporter, mer	0,98	1,97	2,10E-06	1,45E-04	NM_00103327	NM_00103327	NM_00103327	Mm.258893	218756	ENSMUST00000068865
A_51_P112762	***Slc5a3***	solute carrier family 5 (inositol transporters), member 3	0,82	1,76	6,18E-05	1,10E-03	NM_017391	NM_017391	NM_017391	Mm.217354	53881	ENSMUST00000068866
A_55_P2000182	***Slc5a6***	solute carrier family 5 (sodium-dependent vitamin transporter)	0,89	1,85	2,56E-05	6,36E-04	NM_00117762	NM_00117762	NM_00117762	Mm.205463	330064	ENSMUST00000068870
A_55_P2000613	***Slc6a1***	solute carrier family 6 (neurotransmitter transporter, GABA), m	1,49	2,82	2,33E-05	6,02E-04	NM_178703	NM_178703	NM_178703	Mm.5260	232333	ENSMUST00000068871
A_55_P1956553	***Slc6a5***	solute carrier family 6 (neurotransmitter transporter, glycine), r	1,21	2,32	1,40E-07	4,10E-05	ENSMUST000C	0	AY147186	Mm.207053	104245	ENSMUST00000068874
A_66_P106661	***Slc7a1***	solute carrier family 7 (cationic amino acid transporter, y+ syst	1,06	2,08	2,06E-05	5,61E-04	NM_007513	NM_007513	NM_007513	Mm.275489	11987	ENSMUST00000068875
A_51_P268193	***Slc7a10***	solute carrier family 7 (cationic amino acid transporter, y+ syst	0,48	1,39	1,10E-04	1,61E-03	NM_017394	NM_017394	NM_017394	Mm.35567	53896	ENSMUST00000068876
A_55_P2055127	***Slc7a14***	solute carrier family 7 (cationic amino acid transporter, y+ syst	0,96	1,95	1,91E-06	1,38E-04	NM_172861	NM_172861	NM_172861	Mm.285366	241919	ENSMUST00000068877
A_52_P77764	***Slc7a2***	solute carrier family 7 (cationic amino acid transporter, y+ syst	1,29	2,45	2,03E-06	1,43E-04	NM_007514	NM_007514	NM_007514	Mm.4676	11988	ENSMUST00000068878
A_55_P1988384	***Slc7a3***	solute carrier family 7 (cationic amino acid transporter, y+ syst	1,34	2,54	6,95E-07	8,60E-05	NM_007515	NM_007515	NM_007515	Mm.5140	11989	ENSMUST00000068879
A_55_P1999775	***Slc7a4***	solute carrier family 7 (cationic amino acid transporter, y+ syst	0,75	1,68	6,16E-05	1,10E-03	NM_144852	NM_144852	NM_144852	Mm.298878	224022	ENSMUST00000068880
A_55_P2017972	***Slc7a5***	solute carrier family 7 (cationic amino acid transporter, y+ syst	0,96	1,94	8,10E-06	3,26E-04	NM_011404	NM_011404	NM_011404	Mm.27943	20539	ENSMUST00000068882
A_51_P112734	***Slc7a8***	solute carrier family 7 (cationic amino acid transporter, y+ syst	0,75	1,68	2,90E-05	6,83E-04	NM_016972	NM_016972	NM_016972	Mm.276831	50934	ENSMUST00000068883
A_55_P2049999	***Slc9a1***	solute carrier family 9 (sodium/hydrogen exchanger), member	0,59	1,50	4,07E-05	8,42E-04	NM_016981	NM_016981	NM_016981	Mm.4312	20544	ENSMUST00000068890
A_51_P192149	***Slc9a6***	solute carrier family 9 (sodium/hydrogen exchanger), member	0,72	1,64	9,89E-05	1,51E-03	NM_172780	NM_172780	NM_172780	Mm.17815	236794	ENSMUST00000068891

A_52_P85791	**Slc9a7**	solute carrier family 9 (sodium/hydrogen exchanger), member	1,74	3,34	5,50E-07	7,71E-05	NM_177353	NM_177353	NM_177353	Mm.132983	236727	ENSMUST00000068892
A_52_P296996	**Slc9a8**	solute carrier family 9 (sodium/hydrogen exchanger), member	0,76	1,69	2,66E-06	1,66E-04	NM_148929	NM_148929	NM_148929	Mm.278924	77031	ENSMUST00000068893
A_52_P375323	**Slc9a9**	solute carrier family 9 (sodium/hydrogen exchanger), member	0,84	1,79	7,58E-05	1,25E-03	NM_177909	NM_177909	NM_177909	Mm.326125	331004	ENSMUST00000068894
A_55_P2185832	**Slco1c1**	solute carrier organic anion transporter family, member 1c1	1,75	3,36	1,11E-07	3,65E-05	NM_021471	NM_021471	NM_021471	Mm.284495	58807	ENSMUST00000068895
A_55_P1989703	**Slco2a1**	solute carrier organic anion transporter family, member 2a1	0,53	1,44	1,08E-04	1,59E-03	NM_033314	NM_033314	NM_033314	Mm.207106	24059	ENSMUST00000068897
A_55_P1958394	**Slit2**	slit homolog 2 (Drosophila)	0,77	1,71	7,00E-06	2,98E-04	NM_178804	NM_178804	NM_178804	Mm.289739	20563	ENSMUST00000068898
A_51_P301809	**Slit3**	slit homolog 3 (Drosophila)	1,18	2,26	5,10E-07	7,35E-05	NM_011412	NM_011412	NM_011412	Mm.478604	20564	ENSMUST00000068899
A_51_P141012	**Slitrk1**	SLIT and NTRK-like family, member 1	0,74	1,67	1,04E-04	1,55E-03	NM_199065	NM_199065	NM_199065	Mm.257268	76965	ENSMUST00000068900
A_52_P462125	**Smarca5-ps**	SWI/SNF related, matrix associated, actin depenent regulator	1,14	2,21	1,42E-05	4,50E-04	NR_002888	NR_002888	NR_002888	Mm.389595	545700	ENSMUST00000068901
A_66_P116804	**Smchd1**	SMC hinge domain containing 1	1,47	2,77	2,63E-07	5,52E-05	NM_028887	NM_028887	NM_028887	Mm.194450	74355	ENSMUST00000068902
A_52_P171791	**Smg1**	SMG1 homolog, phosphatidylinositol 3-kinase-related kinase	0,83	1,78	9,78E-05	1,50E-03	NM_00103181	NM_00103181	NM_00103181	Mm.309053	233789	ENSMUST00000068904
A_66_P137654	**Smpd2**	sphingomyelin phosphodiesterase 2, neutral	0,91	1,87	4,49E-05	9,00E-04	NM_009213	NM_009213	NM_009213	Mm.953	20598	ENSMUST00000068905
A_51_P479786	**Smtn**	smoothelin	1,26	2,39	8,63E-08	3,10E-05	NM_013870	NM_013870	NM_013870	Mm.188516	29856	ENSMUST00000068906
A_55_P2007525	**Smu1**	smu-1 suppressor of mec-8 and unc-52 homolog (C. elegans)	0,85	1,80	5,32E-05	1,01E-03	NM_021535	NM_021535	NM_021535	Mm.289929	74255	ENSMUST00000068907
A_52_P600030	**Smyd3**	SET and MYND domain containing 3	0,91	1,87	5,66E-05	1,04E-03	NM_027188	NM_027188	NM_027188	Mm.222338	69726	ENSMUST00000068908
A_55_P2029151	**Snap47**	synaptosomal-associated protein, 47	1,34	2,53	1,93E-06	1,39E-04	NM_144521	NM_144521	NM_144521	Mm.26680	67826	ENSMUST00000068909
A_52_P467140	**Snd1**	staphylococcal nuclease and tudor domain containing 1	1,22	2,33	1,90E-05	5,34E-04	NM_019776	NM_019776	NM_019776	Mm.439987	56463	ENSMUST00000068910
A_55_P2086677	**Snrk**	SNF related kinase	0,61	1,52	3,83E-05	8,09E-04	ENSMUST000	0	AK082185	Mm.257989	20623	ENSMUST00000068911
A_55_P2120682	**Snrpa1**	small nuclear ribonucleoprotein polypeptide A'	0,72	1,65	5,47E-05	1,02E-03	NM_021336	NM_021336	NM_021336	Mm.22362	68981	ENSMUST00000068912
A_66_P111877	**Snx13**	sorting nexin 13	0,91	1,87	4,84E-05	9,48E-04	NM_00101497	NM_00101497	NM_00101497	Mm.213991	217463	ENSMUST00000068913
A_52_P274555	**Snx14**	sorting nexin 14	0,72	1,64	2,29E-05	5,96E-04	NM_172926	NM_172926	NM_172926	Mm.439873	244962	ENSMUST00000068915
A_55_P1975695	**Snx18**	sorting nexin 18	0,73	1,66	8,50E-06	3,35E-04	NM_130796	NM_130796	NM_130796	Mm.33721	170625	ENSMUST00000068916
A_55_P1957389	**Snx25**	sorting nexin 25	0,52	1,44	7,36E-05	1,23E-03	NM_207213	NM_207213	NM_207213	Mm.267258	102141	ENSMUST00000068917
A_55_P2065726	**Snx29**	sorting nexin 29	1,35	2,56	8,16E-07	9,25E-05	ENSMUST000	0	AK086865	Mm.325831	74478	ENSMUST00000068918
A_55_P1952788	**Son**	Son DNA binding protein	1,44	2,71	6,55E-07	8,34E-05	ENSMUST000	0	AB546195	Mm.46401	20658	ENSMUST00000068920
A_55_P1960857	**Sorbs2**	sorbin and SH3 domain containing 2	1,79	3,47	9,72E-05	1,49E-03	NM_00120521	NM_00120521	NM_00120521	Mm.211096	234214	ENSMUST00000068921
A_55_P2044587	**Sorcs1**	VPS10 domain receptor protein SORCS 1	0,66	1,58	5,45E-05	1,02E-03	NM_0012525	NM_0012525	NM_0012525	Mm.313672	58178	ENSMUST00000068922
A_55_P2042151	**Sorcs2**	sortilin-related VPS10 domain containing receptor 2	1,44	2,71	9,92E-07	1,00E-04	NM_030889	NM_030889	NM_030889	Mm.34113	81840	ENSMUST00000068923
A_66_P123824	**Sorl1**	sortilin-related receptor, LDLR class A repeats-containing	1,49	2,81	4,72E-07	7,16E-05	ENSMUST000	0	AK162665	Mm.121920	20660	ENSMUST00000068925
A_51_P517145	**Sort1**	sortilin 1	0,93	1,90	6,41E-05	1,13E-03	NM_019972	NM_019972	NM_019972	Mm.157119	20661	ENSMUST00000068927
A_52_P475356	**Sox6**	SRY-box containing gene 6	1,08	2,11	9,90E-05	1,51E-03	NM_011445	NM_011445	NM_011445	Mm.323365	20679	ENSMUST00000068928
A_51_P513530	**Spag5**	sperm associated antigen 5	1,01	2,01	2,64E-05	6,49E-04	NM_017407	NM_017407	NM_017407	Mm.24250	54141	ENSMUST00000068929
A_55_P2046807	**Sparc**	secreted acidic cysteine rich glycoprotein	0,83	1,78	3,74E-05	7,96E-04	NM_009242	NM_009242	NM_009242	Mm.291442	20692	ENSMUST00000068930
A_66_P122828	**Spata31**	spermatogenesis associated 31	0,65	1,57	2,21E-05	5,85E-04	NM_030047	NM_030047	NM_030047	Mm.342895	78124	ENSMUST00000068931
A_55_P1955279	**Specc1**	sperm antigen with calponin homology and coiled-coil domain	1,26	2,39	1,25E-06	1,11E-04	NM_00102993	NM_00102993	NM_00102993	Mm.458025	432572	ENSMUST00000068932
A_55_P2087162	**Specc1l**	sperm antigen with calponin homology and coiled-coil domain	1,39	2,63	3,20E-06	1,87E-04	NM_00114582	NM_00114582	NM_00114582	Mm.27716	74392	ENSMUST00000068934
A_55_P2009943	**Spen**	SPEN homolog, transcriptional regulator (Drosophila)	1,23	2,35	1,27E-07	3,94E-05	NM_019763	NM_019763	NM_019763	Mm.299906	56381	ENSMUST00000068935
A_55_P2033849	**Sphkap**	SPHK1 interactor, AKAP domain containing	1,39	2,63	3,36E-06	1,92E-04	NM_172430	NM_172430	NM_172430	Mm.154303	77629	ENSMUST00000068936
A_55_P2082837	**Spin1**	spindlin 1	0,73	1,65	4,81E-05	9,44E-04	NM_146043	NM_146043	NM_146043	Mm.188432	20729	ENSMUST00000068938
A_55_P2081253	**Spock3**	sparc/osteonectin, cwcv and kazal-like domains proteoglycan	0,68	1,60	8,16E-05	1,32E-03	NM_023689	NM_023689	NM_023689	Mm.334552	72902	ENSMUST00000068940
A_51_P151902	**Spon1**	spondin 1, (f-spondin) extracellular matrix protein	1,40	2,63	1,16E-05	3,98E-04	NM_145584	NM_145584	NM_145584	Mm.334160	233744	ENSMUST00000068941
A_55_P2085142	**Spp1**	secreted phosphoprotein 1	0,81	1,75	1,20E-04	1,71E-03	NM_00120423	NM_00120423	NM_00120423	Mm.288474	20750	ENSMUST00000068942
A_55_P2072985	**Sptan1**	spectrin alpha, non-erythrocytic 1	2,62	6,15	5,83E-09	8,90E-06	NM_00107655	NM_00107655	NM_00107655	Mm.204969	20740	ENSMUST00000068943
A_51_P489138	**Sptb**	spectrin beta, erythrocytic	1,56	2,94	4,43E-07	6,97E-05	NM_013675	NM_013675	NM_013675	Mm.32881	20741	ENSMUST00000068945
A_55_P2081223	**Sptbn1**	spectrin beta, non-erythrocytic 1	3,08	8,48	5,18E-10	4,08E-06	NM_175836	NM_175836	NM_175836	Mm.123110	20742	ENSMUST00000068946
A_51_P403799	**Sptbn2**	spectrin beta, non-erythrocytic 2	2,80	6,97	2,84E-09	6,40E-06	NM_021287	NM_021287	NM_021287	Mm.329668	20743	ENSMUST00000068948
A_55_P2075966	**Sptbn4**	spectrin beta, non-erythrocytic 4	1,33	2,51	7,50E-07	8,85E-05	NM_032610	NM_032610	NM_032610	Mm.459123	80297	ENSMUST00000068949
A_51_P345593	**Sptlc1**	serine palmitoyltransferase, long chain base subunit 1	1,03	2,04	2,24E-05	5,90E-04	NM_009269	NM_009269	NM_009269	Mm.240336	268656	ENSMUST00000068950
A_51_P321331	**Sptlc2**	serine palmitoyltransferase, long chain base subunit 2	0,63	1,55	4,57E-05	9,11E-04	NM_011479	NM_011479	NM_011479	Mm.565	20773	ENSMUST00000068951
A_52_P25420	**Srcin1**	SRC kinase signaling inhibitor 1	0,64	1,56	2,75E-05	6,63E-04	NM_018873	NM_018873	NM_018873	Mm.342665	56013	ENSMUST00000068952
A_55_P2042510	**Srgap1**	SLIT-ROBO Rho GTPase activating protein 1	0,95	1,93	3,13E-05	7,15E-04	NM_00108103	NM_00108103	NM_00108103	Mm.288698	117600	ENSMUST00000068953
A_52_P112178	**Srgap2**	SLIT-ROBO Rho GTPase activating protein 2	1,45	2,73	4,22E-06	2,19E-04	NM_00108101	NM_00108101	NM_00108101	Mm.276259	14270	ENSMUST00000068956
A_55_P2137736	**Srgap3**	SLIT-ROBO Rho GTPase activating protein 3	0,85	1,80	9,56E-05	1,47E-03	NM_080448	NM_080448	NM_080448	Mm.236401	259302	ENSMUST00000068957
A_52_P279184	**Srp54a**	signal recognition particle 54A	1,11	2,16	3,92E-05	8,20E-04	NM_011899	NM_011899	NM_011899	Mm.471968	24067	ENSMUST00000068959
A_55_P2166592	**Srrm2**	serine/arginine repetitive matrix 2	1,04	2,06	1,74E-06	1,31E-04	NM_175229	NM_175229	NM_175229	Mm.5222	75956	ENSMUST00000068960
A_51_P420236	**Srrt**	serrate RNA effector molecule homolog (Arabidopsis)	0,66	1,58	9,74E-05	1,49E-03	NM_031405	NM_031405	NM_031405	Mm.387734	83701	ENSMUST00000068962
A_66_P125071	**Ssb**	Sjogren syndrome antigen B	0,86	1,82	4,34E-05	8,80E-04	NM_009278	NM_009278	NM_009278	Mm.10508	20823	ENSMUST00000068963
A_55_P2403135	**Ssh2**	slingshot homolog 2 (Drosophila)	2,10	4,29	1,21E-07	4,80E-05	NM_177710	NM_177710	NM_177710	Mm.440381	237860	ENSMUST00000068964
A_51_P314264	**Ssr2**	signal sequence receptor, beta	1,02	2,03	1,18E-05	4,00E-04	NM_025448	NM_025448	NM_025448	Mm.7091	66256	ENSMUST00000068966
A_52_P312398	**Ssr3**	signal sequence receptor, gamma	0,69	1,62	8,53E-06	3,35E-04	NM_026155	NM_026155	NM_026155	Mm.232728	67437	ENSMUST00000068967
A_52_P546090	**Ssr4**	signal sequence receptor, delta	0,81	1,76	9,52E-06	3,58E-04	NM_009279	NM_009279	NM_009279	Mm.831	20832	ENSMUST00000068968
A_51_P432950	**Sstr1**	somatostatin receptor 1	0,98	1,98	4,82E-07	7,22E-05	NM_009216	NM_009216	NM_009216	Mm.278336	20605	ENSMUST00000068969
A_51_P129546	**Sstr4**	somatostatin receptor 4	1,13	2,19	2,39E-06	1,57E-04	NM_009219	NM_009219	NM_009219	Mm.35324	20608	ENSMUST00000068971
A_51_P352594	**St5**	suppression of tumorigenicity 5	1,60	3,04	2,30E-08	1,66E-05	NM_00100132	NM_00100132	NM_00100132	Mm.252009	76954	ENSMUST00000068972
A_51_P101375	**St6gal1**	beta galactoside alpha 2,6 sialyltransferase 1	0,58	1,49	3,22E-05	7,27E-04	NM_145933	NM_145933	NM_145933	Mm.149029	20440	ENSMUST00000068973
A_51_P232371	**Stab1**	stabilin 1	0,99	1,99	2,96E-06	1,79E-04	NM_138672	NM_138672	NM_138672	Mm.220821	192187	ENSMUST00000068974
A_52_P491861	**Stag1**	stromal antigen 1	0,79	1,73	6,53E-05	1,14E-03	NM_009282	NM_009282	NM_009282	Mm.42135	20842	ENSMUST00000068975
A_55_P2000638	**Stag2**	stromal antigen 2	0,86	1,81	3,40E-06	1,93E-04	NM_00107771	NM_00107771	NM_00107771	Mm.290422	20843	ENSMUST00000068977
A_52_P640204	**Stam**	signal transducing adaptor molecule (SH3 domain and ITAM r	0,85	1,81	7,96E-05	1,29E-03	NM_011484	NM_011484	NM_011484	Mm.273174	20844	ENSMUST00000068978
A_51_P251129	**Stard8**	START domain containing 8	0,79	1,72	3,07E-05	7,09E-04	NM_199018	NM_199018	NM_199018	Mm.236928	236920	ENSMUST00000068979
A_55_P2059606	**Stat2**	signal transducer and activator of transcription 2	0,77	1,71	9,08E-06	3,49E-04	NM_019963	NM_019963	NM_019963	Mm.293120	20847	ENSMUST00000068980
A_55_P1991219	**Stat3**	signal transducer and activator of transcription 3	1,20	2,31	8,92E-07	9,66E-05	NM_011486	NM_011486	NM_011486	Mm.249934	20848	ENSMUST00000068981
A_51_P233334	**Stc1**	stanniocalcin 1	0,76	1,70	7,80E-05	1,28E-03	NM_009285	NM_009285	NM_009285	Mm.20911	20855	ENSMUST00000068988
A_51_P192130	**Stk10**	serine/threonine kinase 10	0,75	1,69	4,24E-05	8,66E-04	NM_009288	NM_009288	NM_009288	Mm.8235	20868	ENSMUST00000068989
A_55_P2056393	**Stk11ip**	serine/threonine kinase 11 interacting protein	0,81	1,75	6,62E-06	2,89E-04	NM_027886	NM_027886	NM_027886	Mm.312650	71728	ENSMUST00000068990
A_55_P2036723	**Stk36**	serine/threonine kinase 36	1,03	2,04	1,31E-06	1,14E-04	NM_175031	NM_175031	NM_175031	Mm.310974	269209	ENSMUST00000068991
A_66_P130366	**Stk38l**	serine/threonine kinase 38 like	0,65	1,57	9,38E-05	1,45E-03	NM_172734	NM_172734	NM_172734	Mm.322121	232533	ENSMUST00000068992
A_55_P1988804	**Stom**	stomatin	0,64	1,56	9,04E-05	1,42E-03	NM_013515	NM_013515	NM_013515	Mm.295284	13830	ENSMUST00000068993
A_52_P639064	**Strbp**	spermatid perinuclear RNA binding protein	1,23	2,34	2,95E-05	6,90E-04	NM_009261	NM_009261	NM_009261	Mm.237095	20744	ENSMUST00000068994
A_51_P515242	**Strip2**	striatin interacting protein 2	0,81	1,75	5,56E-05	1,03E-03	NM_177204	NM_177204	NM_177204	Mm.56097	320609	ENSMUST00000068995
A_52_P505234	**Stt3a**	STT3, subunit of the oligosaccharyltransferase complex, hom	1,86	3,64	9,40E-08	3,22E-05	NM_008408	NM_008408	NM_008408	Mm.2863	16430	ENSMUST00000068996
A_51_P149621	**Stt3b**	STT3, subunit of the oligosaccharyltransferase complex, hom	1,73	3,33	2,44E-06	1,58E-04	NM_024222	NM_024222	NM_024222	Mm.296158	68292	ENSMUST00000068997
A_55_P2009910	**Stxbp3a**	syntaxin binding protein 3A	1,15	2,22	2,99E-05	6,97E-04	NM_011504	NM_011504	NM_011504	Mm.316894	20912	ENSMUST00000068999
A_55_P2105988	**Stxbp5l**	syntaxin binding protein 5-like	1,24	2,36	1,63E-06	1,26E-04	ENSMUST000	0	AK161955	Mm.80170	207227	ENSMUST00000069001
A_51_P142744	**Sulf1**	sulfatase 1	0,95	1,93	1,38E-05	4,42E-04	NM_172294	NM_172294	NM_172294	Mm.45563	240725	ENSMUST00000069003
A_52_P193925	**Sulf2**	sulfatase 2	1,02	2,03	7,25E-05	1,22E-03	NM_028072	NM_028072	NM_028072	Mm.1011	72043	ENSMUST00000069005
A_51_P249360	**Suox**	sulfite oxidase	0,65	1,57	1,93E-05	5,40E-04	NM_173733	NM_173733	NM_173733	Mm.23352	211389	ENSMUST00000069006
A_51_P280532	**Supt16**	suppressor of Ty 16	0,72	1,65	1,40E-04	1,89E-03	NM_033618	NM_033618	NM_033618	Mm.489682	114741	ENSMUST00000069007
A_51_P510663	**Supt3**	suppressor of Ty 3	1,04	2,05	1,58E-06	1,25E-04	NM_178652	NM_178652	NM_178652	Mm.431327	109115	ENSMUST00000069008
A_55_P2085195	**Supt6**	suppressor of Ty 6	1,13	2,18	8,07E-06	3,25E-04	NM_009297	NM_009297	NM_009297	Mm.20755	20926	ENSMUST00000069009
A_55_P2131168	**Sv2c**	synaptic vesicle glycoprotein 2c	1,17	2,25	6,83E-06	2,94E-04	NM_029210	NM_029210	NM_029210	Mm.40436	75209	ENSMUST00000069010
A_51_P354077	**Svil**	supervillin	2,56	5,91	2,41E-08	1,69E-05	NM_153153	NM_153153	NM_153153	Mm.136791	225115	ENSMUST00000069012
A_51_P210143	**Syne2**	spectrin repeat containing, nuclear envelope 2	2,08	4,23	3,39E-09	7,12E-06	NM_00100551	NM_00100551	NM_00100551	Mm.26652	319565	ENSMUST00000069014
A_55_P1955517	**Synj1**	synaptojanin 1	0,81	1,75	2,47E-05	6,21E-04	ENSMUST000	0		0	104015	ENSMUST00000069016
A_55_P2142103	**Synj2**	synaptojanin 2	1,09	2,13	5,54E-06	2,59E-04	NM_00111335	NM_00111335	NM_00111335	Mm.236068	20975	ENSMUST00000069017
A_55_P2052696	**Synm**	synemin, intermediate filament protein	1,58	2,98	1,44E-07	4,10E-05	NM_201639	NM_201639	NM_201639	Mm.60526	233335	ENSMUST00000069018
A_52_P223127	**Synpo2**	synaptopodin 2	1,09	2,12	7,56E-07	8,87E-05	NM_080451	NM_080451	NM_080451	Mm.34359	118449	ENSMUST00000069023
A_55_P2236291	**Syt16**	synaptotagmin XVI	0,88	1,84	5,21E-06	2,48E-04	NM_172804	NM_172804	NM_172804	Mm.311393	238266	ENSMUST00000069024
A_55_P2017894	**Syt2**	synaptotagmin II	1,15	2,22	2,20E-06	1,49E-04	NM_009307	NM_009307	NM_009307	Mm.5102	20980	ENSMUST00000069025
A_52_P628915	**Syt4**	synaptotagmin IV	1,33	2,51	3,27E-06	1,90E-04	NM_009308	NM_009308	NM_009308	Mm.490509	20983	ENSMUST00000069026
A_51_P290921	**Sytl2**	synaptotagmin-like 2	1,05	2,07	3,74E-05	7,96E-04	NM_00104008	NM_00104008	NM_00104008	Mm.26751	83671	ENSMUST00000069027
A_55_P2015734	**Syvn1**	synovial apoptosis inhibitor 1, synoviolin	0,93	1,91	9,41E-05	1,45E-03	NM_028769	NM_028769	NM_028769	Mm.149870	74126	ENSMUST00000069028
A_66_P138567	**Szt2**	seizure threshold 2	1,30	2,46	1,27E-06	1,11E-04	NM_198170	NM_198170	NM_198170	Mm.415218	230676	ENSMUST00000069029
A_55_P2036472	**T2**	brachyury 2	1,11	2,16	6,23E-06	2,80E-04	NM_00116183	NM_00116183	NM_00116183	Mm.482744	21331	ENSMUST00000069031
A_55_P2004797	**Tacc2**	transforming, acidic coiled-coil containing protein 2	0,83	1,78	9,34E-06	3,54E-04	NM_00100446	NM_00100446	NM_00100446	Mm.86322	57752	ENSMUST00000069032
A_55_P2462940	**Taf1**	TAF1 RNA polymerase II, TATA box binding protein (TBP)-ass	1,46	2,75	3,12E-06	1,84E-04	NM_0010810	NM_0010810	NM_0010810	Mm.261750	270627	ENSMUST00000069033
A_52_P261322	**Tanc1**	tetratricopeptide repeat, ankyrin repeat and coiled-coil contain	1,14	2,20	7,61E-06	3,13E-04	NM_198294	NM_198294	NM_198294	Mm.27917	66860	ENSMUST00000069034
A_55_P2294295	**Tanc2**	tetratricopeptide repeat, ankyrin repeat and coiled-coil contain	1,06	2,09	2,06E-06	1,44E-04	NM_181071	NM_181071	NM_181071	Mm.22501	77097	ENSMUST00000069035
A_55_P2028004	**Tas2r124**	taste receptor, type 2, member 124	1,08	2,11	7,72E-06	3,15E-04	NM_207026	NM_207026	NM_207026	Mm.377912	387351	ENSMUST00000069036
A_52_P381430	**Tbc1d4**	TBC1 domain family, member 4	0,98	1,97	5,31E-06	2,52E-04	NM_00108127	NM_00108127	NM_00108127	Mm.320639	210789	ENSMUST00000069037

A_55_P2051293	***Tbcd***	tubulin-specific chaperone d	0,91	1,88	3,32E-05	7,41E-04	NM_029878	NM_029878	NM_029878	Mm.23686	108903	ENSMUST00000069039
A_55_P2405129	***Tbr1***	T-box brain gene 1	0,88	1,84	8,41E-06	3,32E-04	NM_009322	NM_009322	NM_009322	Mm.308525	21375	ENSMUST00000069041
A_52_P621567	***Tcf20***	transcription factor 20	1,15	2,23	7,06E-07	8,63E-05	NM_00111414	NM_00111414	NM_00111414	Mm.252156	21411	ENSMUST00000069042
A_55_P2098061	***Tchp***	trichoplein, keratin filament binding	0,76	1,70	2,07E-05	5,61E-04	NM_029992	NM_029992	NM_029992	Mm.239698	77832	ENSMUST00000069043
A_51_P241861	***Tcirg1***	T cell, immune regulator 1, ATPase, H+ transporting, lysosom	0,99	1,99	4,17E-06	2,18E-04	NM_016921	NM_016921	NM_016921	Mm.271689	27060	ENSMUST00000069044
A_51_P164030	***Tcp1***	t-complex protein 1	1,64	3,11	4,32E-08	2,20E-05	NM_013686	NM_013686	NM_013686	Mm.229342	21454	ENSMUST00000069045
A_55_P1999834	***Tenm1***	teneurin transmembrane protein 1	1,94	3,84	1,42E-07	4,10E-05	ENSMUST000	0	0	0	23963	ENSMUST00000069046
A_55_P2011290	***Tenm2***	teneurin transmembrane protein 2	1,52	2,88	4,28E-06	2,21E-04	NM_011856	NM_011856	NM_011856	Mm.439889	23964	ENSMUST00000069048
A_55_P2028971	***Tenm3***	teneurin transmembrane protein 3	1,20	2,29	9,38E-06	3,55E-04	NM_011857	NM_011857	NM_011857	Mm.42191	23965	ENSMUST00000069049
A_55_P2141088	***Tenm4***	teneurin transmembrane protein 4	1,65	3,13	4,51E-08	2,22E-05	ENSMUST000	0	0	0	23966	ENSMUST00000069051
A_51_P506755	***Tep1***	telomerase associated protein 1	1,02	2,03	1,00E-04	1,52E-03	NM_009351	NM_009351	NM_009351	Mm.318736	21745	ENSMUST00000069053
A_55_P1961320	***Tes***	testis derived transcript	2,32	5,01	6,83E-08	2,81E-05	NM_207176	NM_207176	NM_207176	Mm.436548	21753	ENSMUST00000069054
A_52_P394539	***Tex2***	testis expressed gene 2	0,63	1,55	6,10E-05	1,09E-03	NM_198292	NM_198292	NM_198292	Mm.102407	21763	ENSMUST00000069055
A_55_P1970097	***Tex28***	testis expressed 28	0,96	1,94	3,36E-06	1,92E-04	NM_00112648	NM_00112648	NM_00112648	Mm.475616	385380	ENSMUST00000069056
A_51_P502348	***Tfip11***	tuftelin interacting protein 11	0,91	1,88	2,95E-05	6,91E-04	NM_018783	NM_018783	NM_018783	Mm.172947	54723	ENSMUST00000069057
A_51_P167876	***Tfrc***	transferrin receptor	1,39	2,62	6,47E-06	2,85E-04	NM_011638	NM_011638	NM_011638	Mm.28683	22042	ENSMUST00000069058
A_51_P212754	***Tgfbi***	transforming growth factor, beta induced	0,73	1,66	2,51E-05	6,27E-04	NM_009369	NM_009369	NM_009369	Mm.14455	21810	ENSMUST00000069066
A_65_P16059	***Tgfbr3***	transforming growth factor, beta receptor III	0,75	1,68	1,47E-04	1,95E-03	NM_011578	NM_011578	NM_011578	Mm.200775	21814	ENSMUST00000069067
A_55_P2118575	***Tgs1***	trimethylguanosine synthase homolog (S. cerevisiae)	1,01	2,02	5,96E-06	2,72E-04	NM_054089	NM_054089	NM_054089	Mm.171323	116940	ENSMUST00000069068
A_55_P2275402	***Th***	tyrosine hydroxylase	0,59	1,50	7,75E-05	1,27E-03	NM_009377	NM_009377	NM_009377	Mm.1292	21823	ENSMUST00000069069
A_51_P461877	***Thbs2***	thrombospondin 2	1,30	2,46	3,41E-06	1,94E-04	NM_011581	NM_011581	NM_011581	Mm.26688	21826	ENSMUST00000069070
A_52_P401504	***Thbs4***	thrombospondin 4	0,95	1,94	1,60E-05	4,82E-04	NM_011582	NM_011582	NM_011582	Mm.20865	21828	ENSMUST00000069071
A_51_P263844	***Thnsl1***	threonine synthase-like 1 (bacterial)	0,74	1,67	1,14E-05	3,95E-04	NM_177588	NM_177588	NM_177588	Mm.268841	208967	ENSMUST00000069072
A_55_P2091201	***Thoc2***	THO complex 2	1,19	2,29	2,94E-06	1,79E-04	NM_00103342	NM_00103342	NM_00103342	Mm.259498	331401	ENSMUST00000069073
A_55_P2161219	***Thsd7a***	thrombospondin, type I, domain containing 7A	0,98	1,97	1,59E-05	4,81E-04	NM_00116480	NM_00116480	NM_00116480	Mm.11989	330267	ENSMUST00000069074
A_51_P256066	***Tiam2***	T cell lymphoma invasion and metastasis 2	0,97	1,95	8,99E-06	3,47E-04	NM_011878	NM_011878	NM_011878	Mm.137134	24001	ENSMUST00000069077
A_52_P679101	***Tjp2***	tight junction protein 2	0,63	1,54	1,44E-04	1,93E-03	NM_011597	NM_011597	NM_011597	Mm.104744	21873	ENSMUST00000069078
A_55_P2108599	***Tlcd1***	TLC domain containing 1	1,02	2,02	6,45E-06	2,85E-04	NM_026708	NM_026708	NM_026708	Mm.390375	68385	ENSMUST00000069079
A_52_P451888	***Tlk2***	tousled-like kinase 2 (Arabidopsis)	0,80	1,74	1,52E-04	1,99E-03	NM_011903	NM_011903	NM_011903	Mm.126976	24086	ENSMUST00000069080
A_55_P1956973	***Tln1***	talin 1	1,18	2,27	1,62E-06	1,25E-04	NM_011602	NM_011602	NM_011602	Mm.208601	21894	ENSMUST00000069081
A_55_P2071834	***Tln2***	talin 2	1,27	2,41	9,59E-08	3,25E-05	NM_00108124	NM_00108124	NM_00108124	Mm.33645	70549	ENSMUST00000069083
A_51_P504588	***Tm2d2***	TM2 domain containing 2	0,82	1,77	3,09E-05	7,10E-04	NM_027194	NM_027194	NM_027194	Mm.28626	69742	ENSMUST00000069085
A_55_P2032222	***Tm9sf2***	transmembrane 9 superfamily member 2	1,41	2,65	1,40E-05	4,46E-04	NM_080556	NM_080556	NM_080556	Mm.275191	68059	ENSMUST00000069086
A_51_P368713	***Tmbim4***	transmembrane BAX inhibitor motif containing 4	1,11	2,16	4,55E-06	2,29E-04	NM_026617	NM_026617	NM_026617	Mm.157426	68212	ENSMUST00000069088
A_51_P454993	***Tmcc2***	transmembrane and coiled-coil domains 2	0,59	1,50	1,05E-04	1,57E-03	NM_178874	NM_178874	NM_178874	Mm.273785	68875	ENSMUST00000069089
A_55_P2038117	***Tmem104***	transmembrane protein 104	0,56	1,47	2,81E-05	6,72E-04	NM_0010333	NM_0010333	NM_0010333	Mm.260587	320534	ENSMUST00000069090
A_52_P429749	***Tmem115***	transmembrane protein 115	0,67	1,59	1,03E-04	1,54E-03	NM_019704	NM_019704	NM_019704	Mm.260153	56395	ENSMUST00000069091
A_51_P375693	***Tmem135***	transmembrane protein 135	0,92	1,89	7,35E-05	1,23E-03	NM_028343	NM_028343	NM_028343	Mm.208477	72759	ENSMUST00000069092
A_52_P515094	***Tmem176a***	transmembrane protein 176A	1,12	2,17	2,89E-06	1,76E-04	NM_025326	NM_025326	NM_025326	Mm.27061	66058	ENSMUST00000069093
A_66_P111322	***Tmem178b***	transmembrane protein 178B	0,93	1,90	1,04E-04	1,55E-03	NM_0010041	NM_0010041	NM_0010041	Mm.130209	434008	ENSMUST00000069096
A_51_P392090	***Tmem19***	transmembrane protein 19	0,62	1,53	6,37E-05	1,13E-03	NM_133683	NM_133683	NM_133683	Mm.29647	67226	ENSMUST00000069097
A_55_P2183622	***Tmem201***	transmembrane protein 201	0,59	1,51	1,44E-04	1,93E-03	NM_177672	NM_177672	NM_177672	Mm.266704	230917	ENSMUST00000069098
A_55_P2444965	***Tmem245***	transmembrane protein 245	0,88	1,84	4,19E-05	8,59E-04	NM_175518	NM_175518	NM_175518	Mm.296632	242474	ENSMUST00000069099
A_52_P518270	***Tmem255a***	transmembrane protein 255A	1,06	2,09	2,76E-06	1,70E-04	NM_172930	NM_172930	NM_172930	Mm.72979	245386	ENSMUST00000069101
A_52_P263201	***Tmem33***	transmembrane protein 33	0,88	1,84	1,46E-05	4,59E-04	NM_030108	NM_030108	NM_030108	Mm.23217	67878	ENSMUST00000069102
A_51_P407606	***Tmem48***	transmembrane protein 48	0,89	1,85	3,89E-05	8,16E-04	NM_028355	NM_028355	NM_028355	Mm.28478	72787	ENSMUST00000069103
A_51_P265545	***Tmem57***	transmembrane protein 57	0,58	1,49	1,04E-04	1,55E-03	NM_025382	NM_025382	NM_025382	Mm.99793	66146	ENSMUST00000069104
A_55_P2045268	***Tmem59***	transmembrane protein 59	0,92	1,89	6,50E-05	1,14E-03	NM_029565	NM_029565	NM_029565	Mm.291192	56374	ENSMUST00000069105
A_51_P204387	***Tmem63c***	transmembrane protein 63c	0,92	1,89	2,00E-05	5,51E-04	NM_172583	NM_172583	NM_172583	Mm.110032	217733	ENSMUST00000069106
A_51_P484753	***Tmem66***	transmembrane protein 66	0,66	1,58	5,45E-05	1,02E-03	NM_026432	NM_026432	NM_026432	Mm.290353	67887	ENSMUST00000069107
A_55_P1970474	***Tmem67***	transmembrane protein 67	0,87	1,82	7,94E-05	1,29E-03	NM_177861	NM_177861	NM_177861	Mm.475125	329795	ENSMUST00000069108
A_55_P1969850	***Tmem68***	transmembrane protein 68	1,18	2,26	1,86E-05	5,27E-04	NM_028097	NM_028097	NM_028097	Mm.490391	72098	ENSMUST00000069109
A_55_P2216996	***Tmem87a***	transmembrane protein 87A	1,28	2,43	8,14E-08	3,00E-05	NM_00111049	NM_00111049	NM_00111049	Mm.260712	211499	ENSMUST00000069110
A_51_P212473	***Tmtc3***	transmembrane and tetratricopeptide repeat containing 3	1,34	2,54	5,17E-07	7,41E-05	NM_0010333	NM_0010333	NM_0010333	Mm.296805	237500	ENSMUST00000069111
A_55_P2023777	***Tmx2***	thioredoxin-related transmembrane protein 2	0,68	1,60	7,28E-05	1,23E-03	NM_025868	NM_025868	NM_025868	Mm.371621	66958	ENSMUST00000069112
A_51_P319379	***Tmx4***	thioredoxin-related transmembrane protein 4	1,26	2,40	2,03E-05	5,56E-04	NM_029148	NM_029148	NM_029148	Mm.264096	52837	ENSMUST00000069113
A_52_P355169	***Tnc***	tenascin C	2,48	5,59	2,84E-08	1,75E-05	NM_011607	NM_011607	NM_011607	Mm.454219	21923	ENSMUST00000069114
A_51_P385099	***Tnf***	tumor necrosis factor	0,80	1,74	3,36E-05	7,45E-04	NM_013693	NM_013693	NM_013693	Mm.1293	21926	ENSMUST00000069116
A_52_P663757	***Tnik***	TRAF2 and NCK interacting kinase	1,10	2,14	4,53E-06	2,28E-04	NM_026910	NM_026910	NM_026910	Mm.126193	665113	ENSMUST00000069119
A_52_P127024	***Tnks***	tankyrase, TRF1-interacting ankyrin-related ADP-ribose polym	1,30	2,47	1,13E-05	3,93E-04	NM_175091	NM_175091	NM_175091	Mm.88364	21951	ENSMUST00000069120
A_51_P221362	***Tnks2***	tankyrase, TRF1-interacting ankyrin-related ADP-ribose polym	0,57	1,49	6,27E-05	1,11E-03	NM_00116363	NM_00116363	NM_00116363	Mm.249310	74493	ENSMUST00000069122
A_52_P420308	***Tnpo1***	transportin 1	0,73	1,66	8,32E-05	1,34E-03	ENSMUST000	0	BC058670	Mm.173286	238799	ENSMUST00000069123
A_52_P480544	***Tnpo2***	transportin 2 (importin 3, karyopherin beta 2b)	0,89	1,85	8,71E-06	3,40E-04	NM_145390	NM_145390	NM_145390	Mm.220357	212999	ENSMUST00000069124
A_55_P1974957	***Tnr***	tenascin R	1,24	2,35	1,64E-07	4,37E-05	NM_022312	NM_022312	NM_022312	Mm.44701	21960	ENSMUST00000069125
A_55_P2030578	***Tns1***	tensin 1	0,70	1,62	1,47E-04	1,95E-03	NM_027884	NM_027884	NM_027884	Mm.309975	21961	ENSMUST00000069126
A_52_P403443	***Tns3***	tensin 3	1,29	2,44	1,98E-06	1,41E-04	NM_0010835	NM_0010835	NM_0010835	Mm.337820	319939	ENSMUST00000069127
A_55_P1989296	***Tnxb***	tenascin XB	1,77	3,42	7,35E-08	2,84E-05	NM_031176	NM_031176	NM_031176	Mm.290527	81877	ENSMUST00000069128
A_55_P2133047	***Tollip***	toll interacting protein	1,14	2,20	4,86E-06	2,38E-04	NM_023764	NM_023764	NM_023764	Mm.103551	54473	ENSMUST00000069129
A_55_P2162712	***Tph2***	tryptophan hydroxylase 2	1,66	3,17	8,82E-07	9,63E-05	NM_173391	NM_173391	NM_173391	Mm.31597	216343	ENSMUST00000069130
A_55_P2130219	***Tpr***	translocated promoter region	1,54	2,91	9,48E-07	9,80E-05	NM_133780	NM_133780	NM_133780	Mm.174256	108989	ENSMUST00000069131
A_52_P615401	***Tpra1***	transmembrane protein, adipocyte asscociated 1	0,90	1,86	5,18E-05	9,86E-04	NM_011906	NM_011906	NM_011906	Mm.284480	24100	ENSMUST00000069133
A_55_P2096777	***Tprn***	taperin	2,74	6,68	1,33E-09	5,15E-06	NM_175286	NM_175286	NM_175286	Mm.308539	97031	ENSMUST00000069134
A_51_P144957	***Tram1l1***	translocation associated membrane protein 1-like 1	0,80	1,74	5,45E-05	1,02E-03	NM_146140	NM_146140	NM_146140	Mm.165810	229801	ENSMUST00000069135
A_51_P402435	***Trap1***	TNF receptor-associated protein 1	1,02	2,03	3,58E-05	7,76E-04	NM_026508	NM_026508	NM_026508	Mm.123366	68015	ENSMUST00000069136
A_51_P356525	***Trappc11***	trafficking protein particle complex 11	1,21	2,32	1,18E-05	4,01E-04	NM_177240	NM_177240	NM_177240	Mm.39461	320714	ENSMUST00000069137
A_52_P251425	***Trappc8***	trafficking protein particle complex 8	0,83	1,78	9,62E-05	1,48E-03	NM_029491	NM_029491	NM_029491	Mm.273769	75964	ENSMUST00000069138
A_55_P1974627	***Trappc9***	trafficking protein particle complex 9	1,24	2,36	2,15E-06	1,47E-04	NM_00116464	NM_00116464	NM_00116464	Mm.158902	76510	ENSMUST00000069140
A_55_P2073099	***Trex1***	three prime repair exonuclease 1	1,86	3,63	5,91E-07	8,00E-05	NM_011637	NM_011637	NM_011637	Mm.439964	22040	ENSMUST00000069141
A_55_P2037585	***Trf***	transferrin	1,33	2,51	1,06E-06	1,02E-04	NM_133977	NM_133977	NM_133977	Mm.37214	22041	ENSMUST00000069142
A_66_P136097	***Trim13***	tripartite motif-containing 13	0,86	1,81	3,89E-05	8,16E-04	NM_00116422	NM_00116422	NM_00116422	Mm.23959	66597	ENSMUST00000069144
A_55_P2026863	***Trim32***	tripartite motif-containing 32	1,23	2,34	1,72E-06	1,30E-04	NM_053084	NM_053084	NM_053084	Mm.22786	69807	ENSMUST00000069145
A_55_P2030938	***Trim59***	tripartite motif-containing 59	0,72	1,65	1,06E-04	1,57E-03	NM_025863	NM_025863	NM_025863	Mm.490341	66949	ENSMUST00000069146
A_55_P2006861	***Trio***	triple functional domain (PTPRF interacting)	2,02	4,05	1,26E-07	3,94E-05	NM_0010813	NM_0010813	NM_0010813	Mm.485422	223435	ENSMUST00000069147
A_51_P176265	***Trip11***	thyroid hormone receptor interactor 11	1,80	3,49	5,07E-06	2,44E-04	ENSMUST000	0	BC025876	Mm.482284	109181	ENSMUST00000069150
A_55_P2050380	***Trip11***	thyroid hormone receptor interactor 11	1,54	2,91	2,61E-06	1,64E-04	NM_028446	NM_028446	NM_028446	Mm.208618	109181	ENSMUST00000069151
A_55_P2110235	***Trip12***	thyroid hormone receptor interactor 12	0,92	1,89	3,16E-06	1,86E-04	NM_133975	NM_133975	NM_133975	Mm.209265	14897	ENSMUST00000069153
A_55_P2298438	***Trmt10a***	tRNA methyltransferase 10A	1,13	2,18	4,13E-06	2,16E-04	ENSMUST000	0	AK145681	Mm.471692	108943	ENSMUST00000069154
A_52_P550218	***Trmt12***	tRNA methyltranferase 12	0,83	1,78	6,47E-06	2,85E-04	NM_026642	NM_026642	NM_026642	Mm.278805	68260	ENSMUST00000069155
A_52_P346033	***Trmt1l***	tRNA methyltransferase 1 like	0,96	1,94	9,59E-06	3,59E-04	NM_026876	NM_026876	NM_026876	Mm.289373	98685	ENSMUST00000069156
A_66_P116998	***Tro***	trophinin	1,27	2,40	2,22E-07	4,87E-05	NM_00100227	NM_00100227	NM_00100227	Mm.3597	56191	ENSMUST00000069157
A_55_P1978860	***Trp53bp2***	transformation related protein 53 binding protein 2	1,47	2,78	7,28E-07	8,76E-05	NM_173378	NM_173378	NM_173378	Mm.287450	209456	ENSMUST00000069159
A_55_P2043782	***Trpm1***	transient receptor potential cation channel, subfamily M, mem	0,71	1,64	3,10E-05	7,11E-04	NM_0010391	NM_0010391	NM_0010391	Mm.38875	17364	ENSMUST00000069160
A_55_P2075692	***Trpm3***	transient receptor potential cation channel, subfamily M, mem	1,15	2,22	8,49E-06	3,34E-04	NM_00103524	NM_00103524	NM_00103524	Mm.440339	226025	ENSMUST00000069161
A_55_P2024654	***Trrap***	transformation/transcription domain-associated protein	1,65	3,13	2,83E-08	1,75E-05	NM_0010813	NM_0010813	NM_0010813	Mm.273570	100683	ENSMUST00000069163
A_66_P102528	***Tspan5***	tetraspanin 5	1,00	2,01	1,23E-05	4,11E-04	NM_019571	NM_019571	NM_019571	Mm.31927	56224	ENSMUST00000069164
A_52_P270035	***Tspyl2***	TSPY-like 2	0,82	1,76	2,89E-05	6,80E-04	NM_029836	NM_029836	NM_029836	Mm.9997	52808	ENSMUST00000069165
A_66_P101903	***Tspyl3***	TSPY-like 3	0,68	1,60	6,36E-05	1,13E-03	NM_198617	NM_198617	NM_198617	Mm.277243	241732	ENSMUST00000069166
A_51_P257675	***Tspyl4***	TSPY-like 4	0,84	1,79	3,55E-05	7,70E-04	NM_030203	NM_030203	NM_030203	Mm.490255	72480	ENSMUST00000069167
A_52_P290511	***Tstd2***	thiosulfate sulfurtransferase (rhodanese)-like domain containi	0,73	1,66	5,44E-05	1,02E-03	NM_173033	NM_173033	NM_173033	Mm.331588	272027	ENSMUST00000069168
A_51_P292490	***Ttbk2***	tau tubulin kinase 2	0,81	1,75	1,50E-04	1,98E-03	NM_0010248	NM_0010248	NM_0010248	Mm.275698	140810	ENSMUST00000069169
A_51_P107808	***Ttc19***	tetratricopeptide repeat domain 19	0,93	1,91	1,52E-04	1,99E-03	NM_028360	NM_028360	NM_028360	Mm.489722	72795	ENSMUST00000069170
A_52_P528634	***Ttc27***	tetratricopeptide repeat domain 27	0,98	1,98	8,62E-06	3,38E-04	NM_152817	NM_152817	NM_152817	Mm.209503	74196	ENSMUST00000069176
A_51_P303089	***Ttc28***	tetratricopeptide repeat domain 28	1,18	2,26	9,43E-07	9,79E-05	NM_00126762	NM_00126762	NM_00126762	Mm.295660	209683	ENSMUST00000069177
A_66_P106065	***Ttc3***	tetratricopeptide repeat domain 3	0,98	1,98	2,24E-05	5,89E-04	NM_009441	NM_009441	NM_009441	Mm.213408	22129	ENSMUST00000069178
A_52_P131458	***Ttc39b***	tetratricopeptide repeat domain 39B	0,73	1,66	7,87E-05	1,29E-03	NM_027238	NM_027238	NM_027238	Mm.5540	69863	ENSMUST00000069179
A_51_P103209	***Ttc7***	tetratricopeptide repeat domain 7	0,99	1,98	2,91E-05	6,85E-04	NM_028639	NM_028639	NM_028639	Mm.77396	225049	ENSMUST00000069180
A_51_P262238	***Tub***	tubby candidate gene	0,58	1,50	1,32E-04	1,83E-03	NM_021885	NM_021885	NM_021885	Mm.439722	22141	ENSMUST00000069181
A_51_P315795	***Tubb4a***	tubulin, beta 4A class IVA	1,18	2,27	6,18E-06	2,78E-04	NM_009451	NM_009451	NM_009451	Mm.7420	22153	ENSMUST00000069182
A_55_P2108808	***Tubgcp4***	tubulin, gamma complex associated protein 4	0,72	1,65	1,36E-04	1,86E-03	NM_153387	NM_153387	NM_153387	Mm.383499	51885	ENSMUST00000069183
A_51_P507792	***Tubgcp6***	tubulin, gamma complex associated protein 6	1,00	2,00	2,43E-05	6,16E-04	NM_00116331	NM_00116331	NM_00116331	Mm.380934	328580	ENSMUST00000069184

A_52_P244496	***U2surp***	U2 snRNP-associated SURP domain containing	0,88	1,84	1,49E-05	4,62E-04	NM_00111497	NM_00111497	NM_00111497	Mm.292742	67958	ENSMUST00000069186
A_55_P1971966	***Uba1***	ubiquitin-like modifier activating enzyme 1	1,24	2,36	6,28E-07	8,24E-05	NM_00113608	NM_00113608	NM_00113608	Mm.1104	22201	ENSMUST00000069187
A_55_P2136020	***Uba2***	ubiquitin-like modifier activating enzyme 2	0,67	1,59	4,35E-05	8,81E-04	NM_016682	NM_016682	NM_016682	Mm.27560	50995	ENSMUST00000069188
A_52_P671062	***Uba6***	ubiquitin-like modifier activating enzyme 6	1,32	2,49	7,05E-07	8,63E-05	NM_172712	NM_172712	NM_172712	Mm.486425	231380	ENSMUST00000069189
A_55_P1996941	***Ube2c***	ubiquitin-conjugating enzyme E2C	1,13	2,19	1,05E-06	1,02E-04	NM_026785	NM_026785	NM_026785	Mm.89830	68612	ENSMUST00000069190
A_51_P499061	***Ube2o***	ubiquitin-conjugating enzyme E2O	1,31	2,48	2,22E-06	1,50E-04	NM_173755	NM_173755	NM_173755	Mm.243950	217342	ENSMUST00000069191
A_55_P2077994	***Ube2v2***	ubiquitin-conjugating enzyme E2 variant 2	0,95	1,93	3,66E-06	2,03E-04	NM_023585	NM_023585	NM_023585	Mm.235407	70620	ENSMUST00000069192
A_55_P2158627	***Ube4a***	ubiquitination factor E4A, UFD2 homolog (S. cerevisiae)	0,78	1,72	4,59E-05	9,14E-04	ENSMUST000	0	BC006649	Mm.215069	140630	ENSMUST00000069193
A_55_P2074908	***Ube4b***	ubiquitination factor E4B, UFD2 homolog (S. cerevisiae)	0,71	1,63	4,22E-05	8,62E-04	NM_022022	NM_022022	NM_022022	Mm.393991	63958	ENSMUST00000069194
A_51_P107938	***Ubr1***	ubiquitin protein ligase E3 component n-recognin 1	1,31	2,48	1,35E-05	4,35E-04	NM_009461	NM_009461	NM_009461	Mm.389330	22222	ENSMUST00000069195
A_55_P2008576	***Ubr2***	ubiquitin protein ligase E3 component n-recognin 2	1,34	2,54	4,65E-07	7,10E-05	NM_00117737	NM_00117737	NM_00117737	Mm.28234	224826	ENSMUST00000069196
A_52_P160418	***Ubr3***	ubiquitin protein ligase E3 component n-recognin 3	1,62	3,08	1,83E-07	4,50E-05	NM_177783	NM_177783	NM_177783	Mm.314576	68795	ENSMUST00000069198
A_52_P647607	***Ubr4***	ubiquitin protein ligase E3 component n-recognin 4	2,47	5,53	9,04E-09	1,03E-05	NM_00116031	NM_00116031	NM_00116031	Mm.271956	69116	ENSMUST00000069200
A_55_P1980651	***Ubr5***	ubiquitin protein ligase E3 component n-recognin 5	1,20	2,29	3,96E-06	2,12E-04	NM_00108135	NM_00108135	NM_00108135	Mm.476840	70790	ENSMUST00000069202
A_52_P24308	***Uchl1***	ubiquitin carboxy-terminal hydrolase L1	0,73	1,65	6,07E-05	1,09E-03	NM_011670	NM_011670	NM_011670	Mm.29807	22223	ENSMUST00000069203
A_51_P446417	***Ufl1***	UFM1 specific ligase 1	1,13	2,19	2,15E-06	1,47E-04	NM_026194	NM_026194	NM_026194	Mm.226295	67490	ENSMUST00000069204
A_55_P2016089	***Uggt1***	UDP-glucose glycoprotein glucosyltransferase 1	0,55	1,46	1,44E-04	1,93E-03	NM_198899	NM_198899	NM_198899	Mm.261022	320011	ENSMUST00000069205
A_52_P439263	***Ugt8a***	UDP galactosyltransferase 8A	0,99	1,99	6,57E-05	1,15E-03	NM_011674	NM_011674	NM_011674	Mm.306021	22239	ENSMUST00000069206
A_66_P120074	***Uhrf1***	ubiquitin-like, containing PHD and RING finger domains, 1	1,07	2,10	5,19E-05	9,87E-04	NM_00111107	NM_00111107	NM_00111107	Mm.42196	18140	ENSMUST00000069207
A_55_P1983070	***Uhrf1bp1***	UHRF1 (ICBP90) binding protein 1	0,73	1,66	8,48E-05	1,35E-03	NM_00108076	NM_00108076	NM_00108076	Mm.291291	224648	ENSMUST00000069208
A_51_P141772	***Uhrf2***	ubiquitin-like, containing PHD and RING finger domains 2	0,74	1,67	7,90E-06	3,20E-04	NM_144873	NM_144873	NM_144873	Mm.313364	109113	ENSMUST00000069209
A_55_P2427900	***Unc13c***	unc-13 homolog C (C. elegans)	1,40	2,65	4,06E-07	6,72E-05	NM_00108115	NM_00108115	NM_00108115	Mm.41035	208898	ENSMUST00000069210
A_55_P2026794	***Unc50***	unc-50 homolog (C. elegans)	0,99	1,99	8,62E-06	3,38E-04	NM_026123	NM_026123	NM_026123	Mm.27404	67387	ENSMUST00000069211
A_52_P380301	***Unc5c***	unc-5 homolog C (C. elegans)	1,19	2,28	1,01E-06	1,01E-04	NM_009472	NM_009472	NM_009472	Mm.24430	22253	ENSMUST00000069212
A_55_P2073313	***Unc79***	unc-79 homolog (C. elegans)	0,65	1,57	4,92E-05	9,56E-04	NM_00108101	NM_00108101	NM_00108101	Mm.387073	217843	ENSMUST00000069213
A_55_P2003586	***Unkl***	unkempt-like (Drosophila)	0,57	1,48	6,57E-05	1,15E-03	NM_00119702	NM_00119702	NM_00119702	Mm.267353	74154	ENSMUST00000069214
A_51_P183051	***Upb1***	ureidopropionase, beta	1,64	3,12	2,16E-06	1,47E-04	NM_133995	NM_133995	NM_133995	Mm.441195	103149	ENSMUST00000069215
A_51_P128648	***Uqcrc2***	ubiquinol cytochrome c reductase core protein 2	0,87	1,83	9,86E-05	1,50E-03	NM_025899	NM_025899	NM_025899	Mm.334206	67003	ENSMUST00000069216
A_52_P484118	***Urb2***	URB2 ribosome biogenesis 2 homolog (S. cerevisiae)	0,83	1,77	4,44E-05	8,93E-04	NM_00102987	NM_00102987	NM_00102987	Mm.334891	382038	ENSMUST00000069217
A_55_P2060722	***Uso1***	USO1 vesicle docking factor	0,96	1,95	3,85E-05	8,12E-04	NM_019490	NM_019490	NM_019490	Mm.15868	56041	ENSMUST00000069218
A_51_P315673	***Usp11***	ubiquitin specific peptidase 11	1,07	2,10	1,36E-05	4,37E-04	NM_145628	NM_145628	NM_145628	Mm.34489	236733	ENSMUST00000069219
A_51_P104077	***Usp13***	ubiquitin specific peptidase 13 (isopeptidase T-3)	0,57	1,48	3,66E-05	7,85E-04	NM_00101302	NM_00101302	NM_00101302	Mm.316153	72607	ENSMUST00000069220
A_65_P11632	***Usp14***	ubiquitin specific peptidase 14	0,97	1,96	5,54E-05	1,03E-03	NM_021522	NM_021522	NM_021522	Mm.329277	59025	ENSMUST00000069221
A_55_P2099785	***Usp15***	ubiquitin specific peptidase 15	0,88	1,84	1,09E-04	1,60E-03	NM_027604	NM_027604	NM_027604	Mm.244209	14479	ENSMUST00000069222
A_66_P138769	***Usp19***	ubiquitin specific peptidase 19	1,53	2,89	3,02E-07	5,92E-05	NM_00116837	NM_00116837	NM_00116837	Mm.289706	71472	ENSMUST00000069223
A_55_P1974238	***Usp20***	ubiquitin specific peptidase 20	0,95	1,93	1,57E-05	4,78E-04	NM_028846	NM_028846	NM_028846	Mm.346654	74270	ENSMUST00000069225
A_55_P2440032	***Usp24***	ubiquitin specific peptidase 24	1,65	3,13	4,40E-07	6,97E-05	NM_183225	NM_183225	NM_183225	Mm.234544	329908	ENSMUST00000069227
A_51_P257982	***Usp29***	ubiquitin specific peptidase 29	0,74	1,67	1,40E-04	1,89E-03	NM_021323	NM_021323	NM_021323	Mm.40752	57775	ENSMUST00000069229
A_52_P452019	***Usp31***	ubiquitin specific peptidase 31	0,64	1,56	1,15E-04	1,66E-03	NM_00103317	NM_00103317	NM_00103317	Mm.122538	76179	ENSMUST00000069230
A_55_P2179335	***Usp33***	ubiquitin specific peptidase 33	1,36	2,57	1,05E-06	1,05E-04	NM_133247	NM_133247	NM_133247	Mm.258320	170822	ENSMUST00000069231
A_52_P58059	***Usp34***	ubiquitin specific peptidase 34	1,73	3,31	1,41E-06	1,18E-04	NM_00119040	NM_00119040	NM_00119040	Mm.119155	17847	ENSMUST00000069232
A_65_P02321	***Usp36***	ubiquitin specific peptidase 36	0,82	1,77	7,75E-05	1,27E-03	NM_00103352	NM_00103352	NM_00103352	Mm.232293	72344	ENSMUST00000069233
A_51_P131216	***Usp54***	ubiquitin specific peptidase 54	0,85	1,80	9,65E-06	3,61E-04	NM_030180	NM_030180	NM_030180	Mm.301173	78787	ENSMUST00000069234
A_52_P622391	***Usp9x***	ubiquitin specific peptidase 9, X chromosome	1,77	3,40	1,13E-07	3,70E-05	NM_009481	NM_009481	NM_009481	Mm.242646	22284	ENSMUST00000069235
A_51_P347467	***Utp14a***	UTP14, U3 small nucleolar ribonucleoprotein, homolog A (yea	0,81	1,75	4,49E-05	8,99E-04	NM_028276	NM_028276	NM_028276	Mm.336045	72554	ENSMUST00000069236
A_55_P2212027	***Utrn***	utrophin	1,19	2,28	1,13E-05	3,92E-04	NM_011682	NM_011682	NM_011682	Mm.331784	22288	ENSMUST00000069237
A_51_P203501	***Vars***	valyl-tRNA synthetase	1,03	2,04	1,90E-06	1,37E-04	NM_011690	NM_011690	NM_011690	Mm.28420	22321	ENSMUST00000069238
A_51_P210956	***Vcam1***	vascular cell adhesion molecule 1	1,48	2,80	1,29E-05	4,25E-04	NM_011693	NM_011693	NM_011693	Mm.76649	22329	ENSMUST00000069239
A_55_P1960208	***Vcan***	versican	1,58	2,98	2,70E-05	6,56E-04	NM_172955	NM_172955	NM_172955	Mm.158700	13003	ENSMUST00000069241
A_66_P133244	***Vcl***	vinculin	1,56	2,95	3,00E-07	5,90E-05	NM_009502	NM_009502	NM_009502	Mm.279361	22330	ENSMUST00000069243
A_52_P638895	***Vegfa***	vascular endothelial growth factor A	0,71	1,64	9,15E-05	1,43E-03	NM_00102525	NM_00102525	NM_00102525	Mm.282184	22339	ENSMUST00000069245
A_52_P96552	***Vkorc1l1***	vitamin K epoxide reductase complex, subunit 1-like 1	0,81	1,76	8,88E-05	1,40E-03	NM_027121	NM_027121	NM_027121	Mm.288718	69568	ENSMUST00000069246
A_55_P2026790	***Vprbp***	Vpr (HIV-1) binding protein	1,00	2,00	5,56E-05	1,03E-03	NM_00101550	NM_00101550	NM_00101550	Mm.489783	321006	ENSMUST00000069247
A_51_P356822	***Vps13c***	vacuolar protein sorting 13C	1,20	2,30	1,82E-07	4,50E-05	AK048766	0	AK048766	Mm.241454	320528	ENSMUST00000069249
A_51_P175974	***Vps13d***	vacuolar protein sorting 13D	1,28	2,42	3,19E-07	6,03E-05	ENSMUST000	0	AK134463	Mm.476856	230895	ENSMUST00000069250
A_51_P109840	***Vtn***	vitronectin	1,21	2,32	1,83E-06	1,35E-04	NM_011707	NM_011707	NM_011707	Mm.3667	22370	ENSMUST00000069251
A_55_P2180121	***Vwa8***	von Willebrand factor A domain containing 8	1,26	2,40	1,85E-06	1,36E-04	NM_173758	NM_173758	NM_173758	Mm.159651	219189	ENSMUST00000069261
A_52_P597860	***Wasf2***	WAS protein family, member 2	0,89	1,86	1,25E-06	1,11E-04	NM_153423	NM_153423	NM_153423	Mm.23566	242687	ENSMUST00000069263
A_52_P96782	***Wasl***	Wiskott-Aldrich syndrome-like (human)	0,75	1,68	6,93E-06	2,96E-04	NM_028459	NM_028459	NM_028459	Mm.1574	73178	ENSMUST00000069264
A_55_P1977330	***Wbp11***	WW domain binding protein 11	0,90	1,87	1,04E-05	3,77E-04	NM_021714	NM_021714	NM_021714	Mm.141197	60321	ENSMUST00000069265
A_55_P1978875	***Wbp7***	WW domain binding protein 7	1,24	2,36	4,14E-07	6,75E-05	NM_029274	NM_029274	NM_029274	Mm.168688	75410	ENSMUST00000069266
A_55_P2457360	***Wdfy3***	WD repeat and FYVE domain containing 3	1,68	3,20	2,02E-07	4,69E-05	NM_172882	NM_172882	NM_172882	Mm.332522	72145	ENSMUST00000069267
A_52_P409675	***Wdr11***	WD repeat domain 11	0,81	1,76	7,52E-06	3,10E-04	NM_172255	NM_172255	NM_172255	Mm.229323	207425	ENSMUST00000069269
A_51_P112445	***Wdr3***	WD repeat domain 3	0,82	1,77	4,05E-06	2,15E-04	NM_175552	NM_175552	NM_175552	Mm.24591	269470	ENSMUST00000069270
A_52_P218002	***Wdr36***	WD repeat domain 36	1,23	2,34	1,43E-06	1,19E-04	NM_144863	NM_144863	NM_144863	Mm.38816	225348	ENSMUST00000069273
A_52_P434052	***Wdr37***	WD repeat domain 37	1,06	2,09	2,09E-06	1,45E-04	NM_00103938	NM_00103938	NM_00103938	Mm.284654	207615	ENSMUST00000069274
A_52_P293556	***Wdr41***	WD repeat domain 41	0,91	1,88	4,39E-05	8,86E-04	NM_172590	NM_172590	NM_172590	Mm.33856	218460	ENSMUST00000069275
A_55_P2178197	***Wdr45l***	Wdr45 like	0,66	1,58	9,98E-05	1,51E-03	NM_025793	NM_025793	NM_025793	Mm.103986	66840	ENSMUST00000069276
A_51_P211573	***Wdr47***	WD repeat domain 47	0,92	1,89	1,13E-04	1,64E-03	NM_181400	NM_181400	NM_181400	Mm.285968	99512	ENSMUST00000069277
A_51_P118650	***Wdr6***	WD repeat domain 6	1,47	2,77	2,13E-07	4,78E-05	NM_031392	NM_031392	NM_031392	Mm.335454	83669	ENSMUST00000069278
A_55_P2105794	***Wdr61***	WD repeat domain 61	0,65	1,57	8,33E-05	1,34E-03	NM_00102537	NM_00102537	NM_00102537	Mm.28437	66317	ENSMUST00000069288
A_55_P2238255	***Wdr7***	WD repeat domain 7	1,56	2,94	3,27E-07	6,07E-05	NM_00101498	NM_00101498	NM_00101498	Mm.30850	104082	ENSMUST00000069289
A_51_P125050	***Wdr74***	WD repeat domain 74	0,76	1,70	4,74E-05	9,37E-04	NM_134139	NM_134139	NM_134139	Mm.29627	107071	ENSMUST00000069290
A_51_P111455	***Wdr77***	WD repeat domain 77	0,95	1,93	1,15E-06	1,07E-04	NM_027432	NM_027432	NM_027432	Mm.5110	70465	ENSMUST00000069293
A_55_P1980421	***Wdr81***	WD repeat domain 81	0,91	1,88	1,99E-06	1,41E-04	NM_138950	NM_138950	NM_138950	Mm.340378	192652	ENSMUST00000069294
A_51_P299216	***Wdr90***	WD repeat domain 90	0,80	1,74	3,75E-05	7,96E-04	NM_00116376	NM_00116376	NM_00116376	Mm.35470	106618	ENSMUST00000069296
A_52_P165455	***Wwp1***	WW domain containing E3 ubiquitin protein ligase 1	0,85	1,80	4,57E-05	9,10E-04	NM_177327	NM_177327	NM_177327	Mm.78312	107568	ENSMUST00000069297
A_51_P386358	***Wwp2***	WW domain containing E3 ubiquitin protein ligase 2	0,45	1,37	1,36E-04	1,85E-03	NM_025830	NM_025830	NM_025830	Mm.390058	66894	ENSMUST00000069298
A_52_P678117	***Xpr1***	xenotropic and polytropic retrovirus receptor 1	0,87	1,83	2,56E-05	6,37E-04	NM_011273	NM_011273	NM_011273	Mm.266215	19775	ENSMUST00000069299
A_52_P403020	***Xrn1***	5'-3' exoribonuclease 1	1,10	2,14	7,96E-06	3,22E-04	NM_011916	NM_011916	NM_011916	Mm.291179	24127	ENSMUST00000069300
A_55_P2016316	***Xrn2***	5'-3' exoribonuclease 2	1,10	2,15	1,19E-06	1,09E-04	NM_011917	NM_011917	NM_011917	Mm.3065	24128	ENSMUST00000069301
A_51_P327904	***Yipf1***	Yip1 domain family, member 1	0,69	1,61	3,98E-05	8,30E-04	NM_145550	NM_145550	NM_145550	Mm.29118	230584	ENSMUST00000069302
A_55_P1975405	***Ylpm1***	YLP motif containing 1	0,80	1,75	3,17E-05	7,21E-04	NM_178363	NM_178363	NM_178363	Mm.153183	56531	ENSMUST00000069303
A_66_P131788	***Ythdf2***	YTH domain family 2	1,44	2,71	1,72E-06	1,31E-04	NM_145393	NM_145393	NM_145393	Mm.23998	213541	ENSMUST00000069305
A_55_P2019929	***Ythdf3***	YTH domain family 3	1,18	2,26	1,04E-06	1,02E-04	NM_172677	NM_172677	NM_172677	Mm.23834	229096	ENSMUST00000069307
A_52_P377299	***Zbtb11***	zinc finger and BTB domain containing 11	0,97	1,96	8,40E-06	3,32E-04	NM_173026	NM_173026	NM_173026	Mm.282814	271377	ENSMUST00000069309
A_55_P1974892	***Zbtb18***	zinc finger and BTB domain containing 18	0,63	1,55	2,73E-05	6,61E-04	NM_013915	NM_013915	NM_013915	Mm.330700	30928	ENSMUST00000069311
A_52_P916539	***Zbtb34***	zinc finger and BTB domain containing 34	0,87	1,83	1,06E-05	3,81E-04	NM_00108550	NM_00108550	NM_00108550	Mm.296512	241311	ENSMUST00000069312
A_55_P2084533	***Zbtb40***	zinc finger and BTB domain containing 40	0,99	1,99	1,46E-06	1,20E-04	NM_198248	NM_198248	NM_198248	Mm.31068	230848	ENSMUST00000069313
A_51_P464064	***Zc3h13***	zinc finger CCCH type containing 13	1,20	2,30	8,70E-07	9,54E-05	NM_026083	NM_026083	NM_026083	Mm.74704	67302	ENSMUST00000069314
A_51_P267796	***Zc3h15***	zinc finger CCCH-type containing 15	0,72	1,65	3,00E-05	7,00E-04	NM_026934	NM_026934	NM_026934	Mm.27804	69082	ENSMUST00000069316
A_52_P283501	***Zc3h7b***	zinc finger CCCH type containing 7B	1,72	3,28	4,27E-07	6,87E-05	NM_00108101	NM_00108101	NM_00108101	Mm.432075	20286	ENSMUST00000069317
A_55_P2072233	***Zcchc12***	zinc finger, CCHC domain containing 12	0,83	1,77	5,64E-05	1,04E-03	NM_028325	NM_028325	NM_028325	Mm.297698	72693	ENSMUST00000069319
A_55_P2146214	***Zcchc18***	zinc finger, CCHC domain containing 18	0,72	1,65	4,37E-05	8,83E-04	NM_00103551	NM_00103551	NM_00103551	Mm.440155	66995	ENSMUST00000069320
A_55_P2027259	***Zcchc2***	zinc finger, CCHC domain containing 2	0,66	1,58	2,44E-05	6,18E-04	NM_00112267	NM_00112267	NM_00112267	Mm.210188	227449	ENSMUST00000069321
A_51_P213725	***Zdbf2***	zinc finger, DBF-type containing 2	1,35	2,55	1,79E-05	5,14E-04	NM_028673	NM_028673	NM_028673	Mm.86986	73884	ENSMUST00000069322
A_55_P1958371	***Zdhhc17***	zinc finger, DHHC domain containing 17	1,32	2,49	7,05E-05	1,20E-03	NM_172554	NM_172554	NM_172554	Mm.339281	320150	ENSMUST00000069323
A_51_P416439	***Zdhhc6***	zinc finger, DHHC domain containing 6	0,98	1,97	2,51E-05	6,27E-04	NM_00103357	NM_00103357	NM_00103357	Mm.386789	66980	ENSMUST00000069324
A_52_P207635	***Zfc3h1***	zinc finger, C3H1-type containing	0,80	1,74	3,42E-05	7,54E-04	NM_00103326	NM_00103326	NM_00103326	Mm.207621	216345	ENSMUST00000069325
A_55_P2106459	***Zfhx3***	zinc finger homeobox 3	1,28	2,43	4,27E-06	2,20E-04	NM_007496	NM_007496	NM_007496	Mm.416972	11906	ENSMUST00000069326
A_55_P2123841	***Zfhx4***	zinc finger homeodomain 4	1,14	2,20	3,87E-07	6,49E-05	NM_030708	NM_030708	NM_030708	Mm.41522	80892	ENSMUST00000069327
A_55_P2021665	***Zfml***	zinc finger, matrin-like	1,24	2,36	1,51E-07	4,22E-05	NM_008717	NM_008717	NM_008717	Mm.132392	18139	ENSMUST00000069329
A_51_P330615	***Zfp119b***	zinc finger protein 119b	0,81	1,75	1,00E-05	3,68E-04	NM_146249	NM_146249	NM_146249	Mm.441018	240120	ENSMUST00000069330
A_51_P476261	***Zfp120***	zinc finger protein 120	1,07	2,10	6,10E-05	1,09E-03	NM_181266	NM_181266	NM_181266	Mm.485851	104348	ENSMUST00000069331
A_55_P2072080	***Zfp142***	zinc finger protein 142	1,32	2,49	8,29E-07	9,30E-05	NM_029888	NM_029888	NM_029888	Mm.207298	77264	ENSMUST00000069332
A_55_P2111668	***Zfp157***	zinc finger protein 157	0,96	1,95	1,25E-04	1,75E-03	NM_028130	NM_028130	NM_028130	Mm.20263	72154	ENSMUST00000069333
A_52_P196743	***Zfp169***	zinc finger protein 169	0,92	1,90	1,67E-05	4,93E-04	NM_026450	NM_026450	NM_026450	Mm.251375	67911	ENSMUST00000069334
A_65_P09778	***Zfp213***	zinc finger protein 213	0,71	1,64	5,34E-05	1,01E-03	NM_00103349	NM_00103349	NM_00103349	Mm.268582	449521	ENSMUST00000069339
A_55_P2174816	***Zfp266***	zinc finger protein 266	0,85	1,80	1,43E-05	4,53E-04	NM_00108248	NM_00108248	NM_00108248	Mm.431849	77519	ENSMUST00000069340
A_51_P438479	***Zfp281***	zinc finger protein 281	0,57	1,49	1,49E-04	1,97E-03	NM_177643	NM_177643	NM_177643	Mm.62521	226442	ENSMUST00000069342

A_55_P2029136	***Zfp287***	zinc finger protein 287	0,77	1,71	7,23E-06	3,04E-04	NM_133208	NM_133208	NM_133208	Mm.339982	170740	ENSMUST00000069343
A_51_P275591	***Zfp292***	zinc finger protein 292	0,89	1,85	1,62E-05	4,85E-04	NM_013889	NM_013889	NM_013889	Mm.38193	30046	ENSMUST00000069344
A_52_P509185	***Zfp318***	zinc finger protein 318	1,95	3,87	2,44E-08	1,69E-05	NM_207671	NM_207671	NM_207671	Mm.439916	57908	ENSMUST00000069345
A_52_P312371	***Zfp345***	zinc finger protein 345	0,67	1,59	5,47E-05	1,02E-03	NM_00103490	NM_00103490	NM_00103490	Mm.451002	545471	ENSMUST00000069347
A_51_P273705	***Zfp354c***	zinc finger protein 354C	1,02	2,03	1,67E-06	1,29E-04	NM_013922	NM_013922	NM_013922	Mm.103674	30944	ENSMUST00000069348
A_52_P61703	***Zfp369***	zinc finger protein 369	1,08	2,12	5,02E-05	9,67E-04	NM_178364	NM_178364	NM_178364	Mm.151546	170936	ENSMUST00000069349
A_55_P2153941	***Zfp386***	zinc finger protein 386 (Kruppel-like)	0,95	1,93	7,04E-06	2,99E-04	NM_00100406	NM_00100406	NM_00100406	Mm.254997	56220	ENSMUST00000069351
A_51_P372895	***Zfp433***	RIKEN cDNA 1700123A16 gene	0,78	1,72	3,70E-05	7,91E-04	NM_00124306	NM_00124306	NM_00124306	Mm.490466	73610	ENSMUST00000069353
A_51_P446583	***Zfp445***	zinc finger protein 445	0,87	1,83	1,48E-05	4,60E-04	NM_173364	NM_173364	NM_173364	Mm.326477	235682	ENSMUST00000069354
A_55_P2059085	***Zfp457***	zinc finger protein 457	1,10	2,14	9,16E-06	3,50E-04	NM_00100366	NM_00100366	NM_00100366	Mm.439456	431706	ENSMUST00000069355
A_51_P357207	***Zfp518b***	zinc finger protein 518B	0,88	1,84	8,56E-05	1,36E-03	NM_00108114	NM_00108114	NM_00108114	Mm.38277	100515	ENSMUST00000069356
A_55_P2159665	***Zfp534***	zinc finger protein 534	0,85	1,80	7,05E-05	1,20E-03	NM_00112718	NM_00112718	NM_00112718	Mm.327194	100503584	ENSMUST00000069357
A_51_P218953	***Zfp536***	zinc finger protein 536	0,98	1,98	9,45E-07	9,79E-05	NM_172385	NM_172385	NM_172385	Mm.378309	243937	ENSMUST00000069358
A_55_P1976529	***Zfp551***	zinc fingr protein 551	1,25	2,38	2,44E-06	1,58E-04	NM_00103382	NM_00103382	NM_00103382	Mm.361832	619331	ENSMUST00000069360
A_55_P2000903	***Zfp58***	zinc finger protein 58	1,27	2,41	8,64E-06	3,38E-04	NM_00100757	NM_00100757	NM_00100757	Mm.478914	238693	ENSMUST00000069362
A_52_P400857	***Zfp595***	zinc finger protein 595	1,28	2,43	4,47E-06	2,27E-04	NM_177622	NM_177622	NM_177622	Mm.289695	218314	ENSMUST00000069363
A_55_P2130032	***Zfp599***	zinc finger protein 599	0,85	1,81	1,49E-05	4,62E-04	NM_181419	NM_181419	NM_181419	Mm.292381	235048	ENSMUST00000069364
A_66_P124732	***Zfp606***	zinc finger protein 606	0,66	1,58	7,14E-05	1,21E-03	NM_026112	NM_026112	NM_026112	Mm.98929	67370	ENSMUST00000069365
A_55_P2173373	***Zfp62***	zinc finger protein 62	0,74	1,67	3,83E-06	2,08E-04	NM_00102484	NM_00102484	NM_00102484	Mm.16650	22720	ENSMUST00000069366
A_55_P2083997	***Zfp664***	zinc finger protein 664	0,66	1,58	5,70E-05	1,05E-03	NM_00108175	NM_00108175	NM_00108175	Mm.138617	269704	ENSMUST00000069367
A_55_P1982234	***Zfp708***	zinc finger protein 708	0,86	1,81	7,29E-05	1,23E-03	NM_00101232	NM_00101232	NM_00101232	Mm.458849	432769	ENSMUST00000069368
A_51_P472621	***Zfp719***	zinc finger protein 719	0,77	1,71	2,06E-05	5,61E-04	NM_172482	NM_172482	NM_172482	Mm.234422	210105	ENSMUST00000069369
A_51_P366227	***Zfp799***	zinc finger protein 799	0,97	1,96	4,01E-05	8,35E-04	NM_177359	NM_177359	NM_177359	Mm.476307	240064	ENSMUST00000069370
A_55_P2046478	***Zfp81***	zinc finger protein 81	0,88	1,84	4,10E-05	8,45E-04	NM_207541	NM_207541	NM_207541	Mm.170029	224694	ENSMUST00000069371
A_55_P1967301	***Zfp85-rs1***	zinc finger protein 85, related sequence 1	1,16	2,23	2,69E-06	1,67E-04	NM_00100113	NM_00100113	NM_00100113	Mm.288396	22746	ENSMUST00000069373
A_51_P366290	***Zfp865***	zinc finger protein 865	0,77	1,70	1,22E-04	1,72E-03	NM_00103338	NM_00103338	NM_00103338	Mm.86607	319748	ENSMUST00000069374
A_52_P493322	***Zfp866***	zinc finger protein 866	0,66	1,58	6,77E-05	1,17E-03	NM_177899	NM_177899	NM_177899	Mm.41364	330788	ENSMUST00000069375
A_51_P341664	***Zfp87***	zinc finger protein 87	1,05	2,07	1,07E-05	3,81E-04	NM_133228	NM_133228	NM_133228	Mm.138283	170763	ENSMUST00000069376
A_55_P1959076	***Zfp930***	zinc finger protein 930	0,74	1,67	2,67E-05	6,53E-04	NM_00101337	NM_00101337	NM_00101337	Mm.334577	234358	ENSMUST00000069377
A_55_P2117130	***Zfp937***	zinc finger protein 937	1,06	2,09	2,42E-06	1,58E-04	NM_00114241	NM_00114241	NM_00114241	Mm.378428	245174	ENSMUST00000069378
A_52_P239023	***Zfp955b***	zinc finger protein 955B	1,32	2,50	1,77E-06	1,32E-04	NM_00114295	NM_00114295	NM_00114295	Mm.276296	100043468	ENSMUST00000069379
A_51_P336509	***Zfpm2***	zinc finger protein, multitype 2	0,76	1,69	2,62E-05	6,45E-04	NM_011766	NM_011766	NM_011766	Mm.39496	22762	ENSMUST00000069380
A_51_P312149	***Zfr***	zinc finger RNA binding protein	0,70	1,62	1,30E-04	1,80E-03	NM_011767	NM_011767	NM_011767	Mm.273496	22763	ENSMUST00000069381
A_52_P566390	***Zfyve26***	zinc finger, FYVE domain containing 26	0,62	1,53	7,49E-05	1,24E-03	NM_00100855	NM_00100855	NM_00100855	Mm.297919	211978	ENSMUST00000069382
A_55_P1980416	***Zkscan1***	zinc finger with KRAB and SCAN domains 1	0,92	1,89	2,70E-05	6,57E-04	NM_133906	NM_133906	NM_133906	Mm.213114	74570	ENSMUST00000069383
A_55_P2059502	***Zkscan16***	zinc finger with KRAB and SCAN domains 16	1,03	2,04	1,15E-05	3,96E-04	NM_00109932	NM_00109932	NM_00109932	Mm.484169	100041581	ENSMUST00000069384
A_55_P2113071	***Zkscan5***	zinc finger with KRAB and SCAN domains 5	0,53	1,45	1,21E-04	1,72E-03	NM_016683	NM_016683	NM_016683	Mm.332842	22757	ENSMUST00000069385
A_55_P2034270	***Zkscan7***	zinc finger with KRAB and SCAN domains 7	0,73	1,66	1,18E-05	4,00E-04	NM_00117750	NM_00117750	NM_00117750	Mm.483265	382118	ENSMUST00000069386
A_52_P28582	***Zmym2***	zinc finger, MYM-type 2	0,98	1,97	1,24E-05	4,12E-04	NM_029498	NM_029498	NM_029498	Mm.31417	76007	ENSMUST00000069387
A_55_P2122688	***Zmym3***	zinc finger, MYM-type 3	1,13	2,20	1,22E-06	1,10E-04	NM_019831	NM_019831	NM_019831	Mm.23458	56364	ENSMUST00000069388
A_55_P2021991	***Zmym4***	zinc finger, MYM-type 4	0,82	1,77	2,94E-05	6,90E-04	NM_00111439	NM_00111439	NM_00111439	Mm.165401	67785	ENSMUST00000069389
A_55_P2076757	***Znfx1***	zinc finger, NFX1-type containing 1	1,35	2,55	1,67E-07	4,37E-05	NM_00103319	NM_00103319	NM_00103319	Mm.297074	98999	ENSMUST00000069390
A_52_P513345	***Zswim5***	zinc finger SWIM-type containing 5	0,93	1,91	1,23E-05	4,11E-04	NM_00102991	NM_00102991	NM_00102991	Mm.218946	74464	ENSMUST00000069391
A_51_P215995	***Zswim6***	zinc finger SWIM-type containing 6	0,53	1,45	9,90E-05	1,51E-03	NM_145456	NM_145456	NM_145456	Mm.490418	67263	ENSMUST00000069392
A_55_P2039180	***Zswim8***	zinc finger SWIM-type containing 8	0,92	1,89	1,22E-05	4,10E-04	NM_027996	NM_027996	NM_027996	Mm.275082	268721	ENSMUST00000069393
A_55_P2172430	***Zxdb***	zinc finger, X-linked, duplicated B	0,65	1,57	1,15E-04	1,66E-03	NM_00108147	NM_00108147	NM_00108147	Mm.426145	668166	ENSMUST00000069394
A_52_P32903	***Zzef1***	zinc finger, ZZ-type with EF hand domain 1	1,44	2,72	4,45E-07	6,99E-05	NM_00104553	NM_00104553	NM_00104553	Mm.272030	195018	ENSMUST00000069395
