# Supplementary material for: Tracking the Fragile X Mental Retardation Protein in a Highly Ordered Neuronal RiboNucleoParticles Population: A Link between Stalled Polyribosomes and RNA Granules
Source: PLoS Genet. 2016 Jul 27;12(7):e1006192. doi: 10.1371/journal.pgen.1006192 (PMC4963131; doi:10.1371/journal.pgen.1006192)
Supplement: S5 Table — Analysis was performed using DAVID bioinformatics resources with the list of transcripts with FC>1 and pval<0.002 (mRNA as abundant or more abundant in granules than polyribosomes, see S3 Table). GOTERM categories (biological processes, cellular components and molecular functions) with a significant Benjamini’s adjusted pvalue (p<0.05) are presented. (PDF) [file pgen.1006192.s010.pdf]

GOTERM category	Term	PValue	Fold Enrichment	Bonferroni	Benjamini	FDR
Biological process	GO:0007155~cell adhesion	5,07E-16	2,15	1,85E-12	1,85E-12	1,01E-12
	GO:0022610~biological adhesion	5,49E-16	2,15	1,85E-12	9,25E-13	1,01E-12
	GO:0030029~actin filament-based process	8,59E-12	2,89	2,87E-08	9,55E-09	1,57E-08
	GO:0030030~cell projection organization	9,76E-12	2,32	3,25E-08	8,14E-09	1,78E-08
	GO:0016337~cell-cell adhesion	2,95E-11	2,54	9,83E-08	1,97E-08	5,37E-08
	GO:0007010~cytoskeleton organization	7,74E-11	2,24	2,58E-07	4,30E-08	1,41E-07
	GO:0030036~actin cytoskeleton organization	1,29E-10	2,83	4,31E-07	6,16E-08	2,36E-07
	GO:0043062~extracellular structure organization	1,73E-10	2,93	5,76E-07	7,20E-08	3,15E-07
	GO:0000902~cell morphogenesis	3,26E-10	2,23	1,09E-06	1,21E-07	5,94E-07
	GO:0032989~cellular component morphogenesis	9,69E-10	2,11	3,23E-06	3,23E-07	1,77E-06
	GO:0030163~protein catabolic process	1,27E-09	1,84	4,24E-06	3,86E-07	2,32E-06
	GO:0016477~cell migration	4,40E-09	2,33	1,47E-05	1,22E-06	8,02E-06
	GO:0033043~regulation of organelle organization	6,42E-09	2,70	2,14E-05	1,65E-06	1,17E-05
	GO:0006928~cell motion	7,23E-09	2,02	2,41E-05	1,72E-06	1,32E-05
	GO:0044257~cellular protein catabolic process	7,89E-09	1,82	2,63E-05	1,75E-06	1,44E-05
	GO:0051493~regulation of cytoskeleton organization	1,11E-08	3,18	3,70E-05	2,31E-06	2,02E-05
	GO:0007156~homophilic cell adhesion	1,52E-08	2,95	5,06E-05	2,98E-06	2,77E-05
	GO:0009057~macromolecule catabolic process	1,82E-08	1,71	6,08E-05	3,38E-06	3,32E-05
	GO:0044265~cellular macromolecule catabolic process	2,12E-08	1,73	7,08E-05	3,73E-06	3,87E-05
	GO:0051603~proteolysis involved in cellular protein catabolic process	2,53E-08	1,79	8,43E-05	4,21E-06	4,61E-05
	GO:0031175~neuron projection development	6,48E-08	2,28	2,16E-04	1,03E-05	1,18E-04
	GO:0051056~regulation of small GTPase mediated signal transduction	1,05E-07	2,23	3,51E-04	1,60E-05	1,92E-04
	GO:0019941~modification-dependent protein catabolic process	1,57E-07	1,76	5,22E-04	2,27E-05	2,86E-04
	GO:0043632~modification-dependent macromolecule catabolic process	1,57E-07	1,76	5,22E-04	2,27E-05	2,86E-04
	GO:0048858~cell projection morphogenesis	3,14E-07	2,26	1,05E-03	4,36E-05	5,71E-04
	GO:0048666~neuron development	3,20E-07	2,02	1,07E-03	4,27E-05	5,83E-04
	GO:0019226~transmission of nerve impulse	5,27E-07	2,16	1,76E-03	6,76E-05	9,61E-04
	GO:0051129~negative regulation of cellular component organization	6,08E-07	2,95	2,02E-03	7,50E-05	1,11E-03
	GO:0044087~regulation of cellular component biogenesis	9,03E-07	2,97	3,01E-03	1,08E-04	1,65E-03
	GO:0007268~synaptic transmission	1,24E-06	2,28	4,14E-03	1,43E-04	2,27E-03
	GO:0032990~cell part morphogenesis	1,28E-06	2,16	4,25E-03	1,42E-04	2,33E-03
	GO:0048870~cell motility	1,53E-06	1,97	5,07E-03	1,64E-04	2,78E-03
	GO:0051674~localization of cell	1,53E-06	1,97	5,07E-03	1,64E-04	2,78E-03
	GO:0035023~regulation of Rho protein signal transduction	1,69E-06	2,95	5,62E-03	1,76E-04	3,08E-03
	GO:0043242~negative regulation of protein complex disassembly	2,37E-06	4,35	7,86E-03	2,39E-04	4,31E-03
	GO:0032956~regulation of actin cytoskeleton organization	2,58E-06	3,39	8,55E-03	2,53E-04	4,69E-03
	GO:0007267~cell-cell signaling	2,95E-06	1,93	9,80E-03	2,81E-04	5,38E-03
	GO:0000904~cell morphogenesis involved in differentiation	3,17E-06	2,11	1,05E-02	2,93E-04	5,77E-03
	GO:0016192~vesicle-mediated transport	3,33E-06	1,70	1,11E-02	3,00E-04	6,07E-03
	GO:0032970~regulation of actin filament-based process	3,40E-06	3,33	1,13E-02	2,98E-04	6,19E-03
	GO:0007015~actin filament organization	3,71E-06	3,45	1,23E-02	3,17E-04	6,75E-03
	GO:0048812~neuron projection morphogenesis	6,42E-06	2,19	2,12E-02	5,35E-04	1,17E-02
	GO:0030705~cytoskeleton-dependent intracellular transport	6,66E-06	4,31	2,20E-02	5,41E-04	1,21E-02
	GO:0007409~axonogenesis	6,93E-06	2,24	2,29E-02	5,50E-04	1,26E-02
	GO:0051494~negative regulation of cytoskeleton organization	7,54E-06	3,60	2,48E-02	5,84E-04	1,37E-02
	GO:0043254~regulation of protein complex assembly	9,62E-06	3,12	3,15E-02	7,28E-04	1,75E-02
	GO:0050808~synapse organization	1,02E-05	3,52	3,34E-02	7,54E-04	1,85E-02
	GO:0030198~extracellular matrix organization	1,09E-05	2,61	3,56E-02	7,88E-04	1,98E-02
	GO:0032535~regulation of cellular component size	1,36E-05	2,21	4,43E-02	9,63E-04	2,47E-02
	GO:0007610~behavior	1,38E-05	1,71	4,50E-02	9,59E-04	2,51E-02
	GO:0048667~cell morphogenesis involved in neuron differentiation	1,43E-05	2,12	4,66E-02	9,73E-04	2,60E-02
	GO:0007626~locomotory behavior	1,45E-05	1,95	4,71E-02	9,65E-04	2,64E-02
	GO:0030182~neuron differentiation	1,61E-05	1,71	5,21E-02	1,05E-03	2,92E-02
	GO:0042592~homeostatic process	1,70E-05	1,57	5,51E-02	1,09E-03	3,10E-02
	GO:0030832~regulation of actin filament length	1,80E-05	3,39	5,84E-02	1,13E-03	3,29E-02
	GO:0016044~membrane organization	2,11E-05	1,87	6,79E-02	1,30E-03	3,84E-02
	GO:0046578~regulation of Ras protein signal transduction	3,05E-05	2,08	9,67E-02	1,85E-03	5,55E-02
	GO:0007017~microtubule-based process	3,62E-05	1,97	1,14E-01	2,15E-03	6,59E-02
	GO:0006511~ubiquitin-dependent protein catabolic process	3,69E-05	2,23	1,16E-01	2,16E-03	6,72E-02
	GO:0043244~regulation of protein complex disassembly	3,76E-05	3,54	1,18E-01	2,16E-03	6,85E-02
	GO:0048878~chemical homeostasis	4,65E-05	1,70	1,44E-01	2,62E-03	8,46E-02
	GO:0008064~regulation of actin polymerization or depolymerization	5,89E-05	3,25	1,78E-01	3,27E-03	1,07E-01
	GO:0030833~regulation of actin filament polymerization	6,64E-05	3,39	1,99E-01	3,62E-03	1,21E-01
	GO:0010639~negative regulation of organelle organization	6,91E-05	2,95	2,06E-01	3,71E-03	1,26E-01
	GO:0006897~endocytosis	7,02E-05	2,00	2,09E-01	3,71E-03	1,28E-01
	GO:0010324~membrane invagination	7,02E-05	2,00	2,09E-01	3,71E-03	1,28E-01
	GO:0008283~cell proliferation	7,18E-05	1,85	2,13E-01	3,73E-03	1,31E-01
	GO:0032271~regulation of protein polymerization	8,25E-05	3,03	2,40E-01	4,22E-03	1,50E-01
	GO:0044271~nitrogen compound biosynthetic process	8,42E-05	1,75	2,45E-01	4,25E-03	1,53E-01
	GO:0006508~proteolysis	9,37E-05	1,37	2,68E-01	4,65E-03	1,71E-01
	GO:0055080~cation homeostasis	1,01E-04	1,99	2,86E-01	4,95E-03	1,84E-01
	GO:0030031~cell projection assembly	1,07E-04	2,76	2,99E-01	5,14E-03	1,94E-01
	GO:0000910~cytokinesis	1,45E-04	4,14	3,83E-01	6,87E-03	2,63E-01
	GO:0050801~ion homeostasis	1,47E-04	1,73	3,87E-01	6,88E-03	2,67E-01
	GO:0007411~axon guidance	1,72E-04	2,38	4,37E-01	7,94E-03	3,13E-01
	GO:0019725~cellular homeostasis	1,85E-04	1,66	4,60E-01	8,40E-03	3,36E-01
	GO:0030155~regulation of cell adhesion	2,57E-04	2,38	5,76E-01	1,15E-02	4,68E-01
	GO:0060249~anatomical structure homeostasis	3,58E-04	2,45	6,97E-01	1,58E-02	6,51E-01

	**GO:0022604~regulation of cell morphogenesis**	4,05E-04	2,30	7,41E-01	1,76E-02	7,36E-01
	**GO:0007507~heart development**	5,39E-04	1,78	8,34E-01	2,31E-02	9,77E-01
	**GO:0030835~negative regulation of actin filament depolymerization**	5,42E-04	4,35	8,36E-01	2,29E-02	9,83E-01
	**GO:0032940~secretion by cell**	5,91E-04	1,86	8,60E-01	2,46E-02	1,07E+00
	**GO:0008104~protein localization**	6,76E-04	1,38	8,95E-01	2,78E-02	1,22E+00
	**GO:0010769~regulation of cell morphogenesis involved in differentiation**	8,42E-04	2,84	9,40E-01	3,41E-02	1,52E+00
	**GO:0055082~cellular chemical homeostasis**	8,82E-04	1,67	9,47E-01	3,53E-02	1,60E+00
	**GO:0006793~phosphorus metabolic process**	9,49E-04	1,34	9,58E-01	3,74E-02	1,72E+00
	**GO:0006796~phosphate metabolic process**	9,49E-04	1,34	9,58E-01	3,74E-02	1,72E+00
	**GO:0031344~regulation of cell projection organization**	9,95E-04	2,67	9,64E-01	3,88E-02	1,80E+00
	**GO:0001764~neuron migration**	1,04E-03	2,47	9,69E-01	3,99E-02	1,87E+00
	**GO:0000226~microtubule cytoskeleton organization**	1,06E-03	2,10	9,71E-01	4,03E-02	1,92E+00
	**GO:0044057~regulation of system process**	1,17E-03	1,77	9,80E-01	4,40E-02	2,12E+00
	**GO:0010810~regulation of cell-substrate adhesion**	1,26E-03	3,05	9,85E-01	4,68E-02	2,28E+00
	**GO:0032319~regulation of Rho GTPase activity**	1,34E-03	5,08	9,89E-01	4,91E-02	2,42E+00
**Cellular component**	**GO:0042995~cell projection**	3,26E-27	2,55	1,59E-24	1,59E-24	4,67E-24
	**GO:0043005~neuron projection**	3,70E-24	3,41	1,80E-21	9,01E-22	5,31E-21
	**GO:0005856~cytoskeleton**	4,44E-21	1,89	2,16E-18	7,21E-19	6,37E-18
	**GO:0030424~axon**	1,53E-14	3,90	7,46E-12	1,87E-12	2,20E-11
	**GO:0015629~actin cytoskeleton**	6,76E-12	2,73	3,29E-09	6,58E-10	9,70E-09
	**GO:0043232~intracellular non-membrane-bounded organelle**	2,52E-11	1,44	1,23E-08	2,05E-09	3,62E-08
	**GO:0043228~non-membrane-bounded organelle**	2,52E-11	1,44	1,23E-08	2,05E-09	3,62E-08
	**GO:0045202~synapse**	5,95E-11	2,27	2,90E-08	4,14E-09	8,54E-08
	**GO:0044430~cytoskeletal part**	7,23E-11	1,75	3,52E-08	4,40E-09	1,04E-07
	**GO:0030425~dendrite**	1,34E-10	3,34	6,53E-08	7,26E-09	1,92E-07
	**GO:0031252~cell leading edge**	2,35E-10	3,28	1,14E-07	1,14E-08	3,37E-07
	**GO:0030054~cell junction**	2,61E-10	1,97	1,27E-07	1,16E-08	3,75E-07
	**GO:0044420~extracellular matrix part**	3,03E-10	3,54	1,48E-07	1,23E-08	4,35E-07
	**GO:0000267~cell fraction**	1,19E-09	1,81	5,80E-07	4,46E-08	1,71E-06
	**GO:0005604~basement membrane**	1,09E-08	3,63	5,29E-06	3,78E-07	1,56E-05
	**GO:0005626~insoluble fraction**	1,13E-08	1,81	5,48E-06	3,66E-07	1,62E-05
	**GO:0005624~membrane fraction**	1,80E-08	1,82	8,75E-06	5,47E-07	2,58E-05
	**GO:0044459~plasma membrane part**	1,89E-08	1,41	9,20E-06	5,41E-07	2,71E-05
	**GO:0031012~extracellular matrix**	6,89E-08	2,04	3,36E-05	1,86E-06	9,88E-05
	**GO:0030427~site of polarized growth**	1,36E-07	4,00	6,62E-05	3,48E-06	1,95E-04
	**GO:0030426~growth cone**	1,36E-07	4,00	6,62E-05	3,48E-06	1,95E-04
	**GO:0005886~plasma membrane**	1,56E-07	1,26	7,61E-05	3,80E-06	2,24E-04
	**GO:0005578~proteinaceous extracellular matrix**	2,14E-07	2,02	1,04E-04	4,96E-06	3,07E-04
	**GO:0030027~lamellipodium**	2,73E-07	3,56	1,33E-04	6,05E-06	3,92E-04
	**GO:0015630~microtubule cytoskeleton**	7,06E-07	1,77	3,44E-04	1,49E-05	1,01E-03
	**GO:0005912~adherens junction**	7,47E-07	2,79	3,63E-04	1,51E-05	1,07E-03
	**GO:0070161~anchoring junction**	1,90E-06	2,57	9,23E-04	3,69E-05	2,72E-03
	**GO:0043025~cell soma**	1,97E-06	2,61	9,61E-04	3,70E-05	2,83E-03
	**GO:0044456~synapse part**	6,87E-06	2,07	3,34E-03	1,24E-04	9,85E-03
	**GO:0030863~cortical cytoskeleton**	7,33E-06	3,79	3,57E-03	1,28E-04	1,05E-02
	**GO:0005938~cell cortex**	9,54E-06	2,43	4,63E-03	1,60E-04	1,37E-02
	**GO:0005913~cell-cell adherens junction**	1,80E-05	4,27	8,71E-03	2,91E-04	2,58E-02
	**GO:0005829~cytosol**	2,11E-05	1,58	1,02E-02	3,32E-04	3,03E-02
	**GO:0005581~collagen**	3,02E-05	5,36	1,46E-02	4,60E-04	4,34E-02
	**GO:0016323~basolateral plasma membrane**	3,42E-05	2,24	1,65E-02	5,04E-04	4,90E-02
	**GO:0031410~cytoplasmic vesicle**	3,68E-05	1,58	1,78E-02	5,27E-04	5,28E-02
	**GO:0044448~cell cortex part**	5,48E-05	2,79	2,64E-02	7,63E-04	7,87E-02
	**GO:0014069~postsynaptic density**	7,27E-05	3,20	3,48E-02	9,83E-04	1,04E-01
	**GO:0031982~vesicle**	7,70E-05	1,55	3,68E-02	1,01E-03	1,10E-01
	**GO:0016023~cytoplasmic membrane-bounded vesicle**	8,38E-05	1,62	4,00E-02	1,07E-03	1,20E-01
	**GO:0031988~membrane-bounded vesicle**	1,28E-04	1,60	6,06E-02	1,60E-03	1,84E-01
	**GO:0005911~cell-cell junction**	1,38E-04	2,00	6,52E-02	1,68E-03	1,98E-01
	**GO:0000151~ubiquitin ligase complex**	1,53E-04	2,89	7,20E-02	1,82E-03	2,20E-01
	**GO:0048770~pigment granule**	1,62E-04	2,52	7,58E-02	1,88E-03	2,32E-01
	**GO:0042470~melanosome**	1,62E-04	2,52	7,58E-02	1,88E-03	2,32E-01
	**GO:0016459~myosin complex**	1,89E-04	2,84	8,81E-02	2,14E-03	2,71E-01
	**GO:0044463~cell projection part**	2,24E-04	1,98	1,03E-01	2,47E-03	3,20E-01
	**GO:0033267~axon part**	3,88E-04	4,08	1,72E-01	4,19E-03	5,56E-01
	**GO:0060053~neurofilament cytoskeleton**	3,91E-04	7,64	1,73E-01	4,13E-03	5,59E-01
	**GO:0001726~ruffle**	4,25E-04	3,04	1,87E-01	4,40E-03	6,09E-01
	**GO:0030055~cell-substrate junction**	4,99E-04	2,62	2,16E-01	5,06E-03	7,14E-01
	**GO:0019717~synaptosome**	4,99E-04	2,62	2,16E-01	5,06E-03	7,14E-01
	**GO:0048471~perinuclear region of cytoplasm**	7,36E-04	1,83	3,01E-01	7,29E-03	1,05E+00
	**GO:0030864~cortical actin cytoskeleton**	7,60E-04	4,17	3,09E-01	7,38E-03	1,08E+00
	**GO:0000502~proteasome complex**	1,15E-03	2,64	4,30E-01	1,09E-02	1,64E+00
	**GO:0044449~contractile fiber part**	1,48E-03	2,25	5,13E-01	1,37E-02	2,10E+00
	**GO:0005783~endoplasmic reticulum**	1,61E-03	1,33	5,43E-01	1,47E-02	2,28E+00
	**GO:0005819~spindle**	1,88E-03	2,06	6,01E-01	1,69E-02	2,67E+00
	**GO:0005924~cell-substrate adherens junction**	1,94E-03	2,51	6,11E-01	1,70E-02	2,74E+00
	**GO:0043292~contractile fiber**	1,94E-03	2,15	6,11E-01	1,67E-02	2,75E+00
	**GO:0005874~microtubule**	2,03E-03	1,66	6,28E-01	1,72E-02	2,87E+00
	**GO:0030016~myofibril**	2,86E-03	2,13	7,52E-01	2,37E-02	4,02E+00
	**GO:0005925~focal adhesion**	2,94E-03	2,50	7,62E-01	2,40E-02	4,14E+00
	**GO:0012505~endomembrane system**	3,27E-03	1,39	7,97E-01	2,62E-02	4,59E+00

	**GO:0030175~filopodium**	3,89E-03	3,71	8,51E-01	3,07E-02	5,44E+00
	**GO:0031594~neuromuscular junction**	3,89E-03	3,71	8,51E-01	3,07E-02	5,44E+00
	**GO:0045211~postsynaptic membrane**	5,26E-03	1,86	9,23E-01	4,06E-02	7,29E+00
	**GO:0005815~microtubule organizing center**	5,48E-03	1,67	9,31E-01	4,16E-02	7,58E+00
**Molecular function**	**GO:0008092~cytoskeletal protein binding**	1,65E-18	2,52	1,81E-15	1,81E-15	2,63E-15
	**GO:0003779~actin binding**	8,84E-16	2,72	9,78E-13	4,89E-13	1,42E-12
	**GO:0046872~metal ion binding**	1,16E-12	1,30	1,28E-09	4,26E-10	1,86E-09
	**GO:0043167~ion binding**	3,65E-12	1,29	4,02E-09	1,01E-09	5,84E-09
	**GO:0043169~cation binding**	3,66E-12	1,29	4,03E-09	8,05E-10	5,85E-09
	**GO:0030695~GTPase regulator activity**	4,07E-10	2,14	4,48E-07	7,47E-08	6,51E-07
	**GO:0005509~calcium ion binding**	5,48E-10	1,69	6,04E-07	8,62E-08	8,77E-07
	**GO:0019899~enzyme binding**	7,73E-10	2,46	8,51E-07	1,06E-07	1,24E-06
	**GO:0060589~nucleoside-triphosphatase regulator activity**	8,79E-10	2,11	9,68E-07	1,08E-07	1,41E-06
	**GO:0032559~adenyl ribonucleotide binding**	1,36E-08	1,45	1,49E-05	1,49E-06	2,17E-05
	**GO:0005524~ATP binding**	1,45E-08	1,46	1,59E-05	1,45E-06	2,31E-05
	**GO:0030554~adenyl nucleotide binding**	1,06E-07	1,41	1,16E-04	9,70E-06	1,69E-04
	**GO:0005083~small GTPase regulator activity**	1,51E-07	2,25	1,66E-04	1,28E-05	2,42E-04
	**GO:0001883~purine nucleoside binding**	2,05E-07	1,40	2,25E-04	1,61E-05	3,28E-04
	**GO:0005085~guanyl-nucleotide exchange factor activity**	2,81E-07	2,56	3,09E-04	2,06E-05	4,50E-04
	**GO:0001882~nucleoside binding**	3,29E-07	1,39	3,63E-04	2,27E-05	5,27E-04
	**GO:0032553~ribonucleotide binding**	5,08E-07	1,35	5,59E-04	3,29E-05	8,12E-04
	**GO:0032555~purine ribonucleotide binding**	5,08E-07	1,35	5,59E-04	3,29E-05	8,12E-04
	**GO:0008270~zinc ion binding**	1,84E-06	1,30	2,03E-03	1,13E-04	2,95E-03
	**GO:0017076~purine nucleotide binding**	2,54E-06	1,32	2,79E-03	1,47E-04	4,06E-03
	**GO:0000166~nucleotide binding**	2,81E-06	1,29	3,09E-03	1,55E-04	4,49E-03
	**GO:0005089~Rho guanyl-nucleotide exchange factor activity**	6,82E-06	3,09	7,48E-03	3,58E-04	1,09E-02
	**GO:0051015~actin filament binding**	7,07E-06	3,62	7,76E-03	3,54E-04	1,13E-02
	**GO:0005088~Ras guanyl-nucleotide exchange factor activity**	7,16E-06	2,89	7,85E-03	3,43E-04	1,14E-02
	**GO:0003774~motor activity**	1,38E-05	2,35	1,51E-02	6,34E-04	2,21E-02
	**GO:0015171~amino acid transmembrane transporter activity**	4,24E-05	3,34	4,56E-02	1,86E-03	6,77E-02
	**GO:0015085~calcium ion transmembrane transporter activity**	4,93E-05	8,13	5,29E-02	2,09E-03	7,88E-02
	**GO:0048407~platelet-derived growth factor binding**	4,93E-05	8,13	5,29E-02	2,09E-03	7,88E-02
	**GO:0005388~calcium-transporting ATPase activity**	4,93E-05	8,13	5,29E-02	2,09E-03	7,88E-02
	**GO:0005201~extracellular matrix structural constituent**	5,77E-05	4,18	6,16E-02	2,35E-03	9,23E-02
	**GO:0046914~transition metal ion binding**	7,85E-05	1,21	8,28E-02	3,08E-03	1,25E-01
	**GO:0019900~kinase binding**	1,16E-04	2,58	1,20E-01	4,39E-03	1,85E-01
	**GO:0051020~GTPase binding**	1,75E-04	2,86	1,75E-01	6,40E-03	2,79E-01
	**GO:0004714~transmembrane receptor protein tyrosine kinase activity**	1,77E-04	2,98	1,77E-01	6,26E-03	2,82E-01
	**GO:0017016~Ras GTPase binding**	2,19E-04	2,93	2,14E-01	7,51E-03	3,50E-01
	**GO:0015179~L-amino acid transmembrane transporter activity**	2,26E-04	3,96	2,21E-01	7,53E-03	3,62E-01
	**GO:0019901~protein kinase binding**	2,95E-04	2,65	2,78E-01	9,52E-03	4,71E-01
	**GO:0050840~extracellular matrix binding**	3,23E-04	4,18	2,99E-01	1,01E-02	5,15E-01
	**GO:0031267~small GTPase binding**	3,30E-04	2,83	3,05E-01	1,00E-02	5,26E-01
	**GO:0016879~ligase activity, forming carbon-nitrogen bonds**	3,35E-04	1,89	3,08E-01	9,91E-03	5,34E-01
	**GO:0019838~growth factor binding**	3,53E-04	2,61	3,22E-01	1,02E-02	5,63E-01
	**GO:0005516~calmodulin binding**	4,28E-04	2,20	3,76E-01	1,20E-02	6,82E-01
	**GO:0015082~di-, tri-valent inorganic cation transmembrane transporter activ**	5,59E-04	3,59	4,60E-01	1,53E-02	8,90E-01
	**GO:0015293~symporter activity**	6,66E-04	2,06	5,20E-01	1,77E-02	1,06E+00
	**GO:0005275~amine transmembrane transporter activity**	7,02E-04	2,65	5,38E-01	1,82E-02	1,12E+00
	**GO:0004842~ubiquitin-protein ligase activity**	7,38E-04	2,21	5,57E-01	1,87E-02	1,17E+00
	**GO:0019992~diacylglycerol binding**	1,08E-03	2,66	6,97E-01	2,68E-02	1,72E+00
	**GO:0017048~Rho GTPase binding**	1,25E-03	3,92	7,46E-01	3,00E-02	1,97E+00
	**GO:0019894~kinesin binding**	1,33E-03	6,27	7,68E-01	3,13E-02	2,10E+00
